# Frequency and prognostic outcomes of emergency diagnosis in 13 non-neoplastic conditions in England: A population-based cohort study using linked electronic health records of 1.7 million patients

**DOI:** 10.1371/journal.pmed.1005182

**Published:** 2026-08-25

**Authors:** Emma Whitfield, Becky White, Matthew E. Barclay, Meena Rafiq, Nadine Zakkak, Marta Berglund, Hardeep Singh, Sean McPhail, Spiros Denaxas, Georgios Lyratzopoulos

**Affiliations:** 1 ECHO (Epidemiology of Cancer Healthcare & Outcomes), Department of Behavioural Science and Health, Institute of Epidemiology and Health Care, UCL (University College London), London, United Kingdom; 2 Department of General Practice and Primary Care, University of Melbourne, Melbourne, Australia; 3 Cancer Intelligence, Cancer Research UK, London, United Kingdom; 4 Safety, Quality & Well-Being Institute, Houston Methodist, Houston, Texas, United States of America; 5 National Disease Registration Service, NHS England, Leeds, United Kingdom; 6 Institute of Health Informatics, UCL, London, United Kingdom; 7 Interdisciplinary Transformation University, Linz, Austria; 8 Health Data Research UK, London, United Kingdom; 9 UCL Hospitals Biomedical Research Centre, London, United Kingdom; 10 British Heart Foundation Data Science Centre, London, United Kingdom; Ministry of Health: Gobierno de Chile Ministerio de Salud, CHILE

## Abstract

**Background:**

In patients with cancer, diagnosis through or soon after the use of emergency hospital care (‘emergency diagnosis’) is associated with advanced disease and poor prognosis. The varied and complex reasons for emergency diagnoses—including rapid disease progression, patient factors, and system delays—are unlikely to be unique to cancer. However, little is known about the frequency and prognostic outcomes of emergency diagnosis in other conditions. We aimed to document the frequency of emergency diagnoses across various non-neoplastic conditions and examine the association of emergency diagnosis with clinical outcomes.

**Methods and findings:**

We analysed records from linked primary care, secondary care, and death data (Clinical Practice Research Datalink (CPRD), Hospital Episode Statistics, Office for National Statistics) for 1,701,154 patients with one of 13 exemplar conditions (axial spondyloarthritis, coeliac disease, coronary/ischaemic heart disease, chronic obstructive pulmonary disease (COPD), inflammatory bowel disease (IBD), Lyme disease, multiple sclerosis (MS), Parkinson’s disease, polycystic ovary syndrome, rheumatoid arthritis, schizophrenia, subacute bacterial endocarditis, and tuberculosis) in England between 1999 and 2019. We examined the percentage of patients diagnosed as an emergency and compared their mortality and time in hospital in the year post-diagnosis with patients who were not diagnosed as an emergency. Emergency diagnosis occurred in at least 20% of patients for 9 out of 13 conditions in our sample, including more than 30% of patients with Parkinson’s disease and 35% of patients with COPD. For 9 out of 13 conditions, there was a substantial increase (at least +10% difference) in 1-year mortality in patients diagnosed as an emergency versus patients who were not diagnosed as an emergency. After adjusting for age and year of diagnosis, deprivation, comorbidity burden, and the healthcare setting in which the diagnosis was made, patients diagnosed as an emergency were typically much more likely to die within one year. This association held even in conditions with generally good prognosis, such as coeliac disease (adjusted odds ratio 7.53, 95% CI [5.64, 10.1], women) and IBD (adjusted odds ratio 5.99, 95% CI [5.30, 6.78], men). Similarly large differences were observed for time in hospital in the year post-diagnosis by emergency diagnosis status. For example, adjusted rate ratios of 8.76 (95% CI [6.52, 11.8]) for men with coeliac disease, and 5.59 (95%CI [2.57, 12.2]) for women diagnosed with Lyme disease. The findings were consistent across two subsets of CPRD data (Aurum, GOLD). Analyses of patients with five supplementary cancer sites (brain, colon, lung, pancreas, and ovary) produced findings concordant with prior literature, supporting the validity of the study methods. The main study limitation is the assumption of accurate recording of diagnoses in health records.

**Conclusions:**

Emergency diagnosis is common and associated with worse clinical outcomes in a wide range of conditions with diverse aetiology. Further research and investment to reduce the number of emergency diagnoses is needed, particularly for diseases where diagnosis in community settings should be expected (e.g., rheumatoid arthritis and COPD), and conditions such as coeliac disease, IBD, and MS, where emergency diagnosis is associated with particularly poor outcomes.

## Author summary

**Why was this study done?:**

- There is growing recognition that improvements are needed to enable patients to be diagnosed with many conditions earlier.
- Many patients with cancer are diagnosed as an emergency (the diagnosis is made after the patient receives emergency hospital care) and experience worse outcomes—consequently, substantial efforts have been made to reduce the number of patients diagnosed with cancer as an emergency.
- It is unclear if emergency diagnosis is a phenomenon specific to cancer or if it also occurs frequently in other conditions.

**What did the researchers do and find?:**

- We examined health records from ~1.7 million patients in England who were diagnosed with 13 conditions between 1999 and 2019.
- Emergency diagnoses were common, with more than one in five patients diagnosed after an emergency presentation in nine conditions.
- Patients diagnosed as an emergency were more likely to die and to spend longer in hospital in the year after diagnosis.

**What do these findings mean?:**

- It is unclear why emergency diagnoses happen—further research should consider both general and disease-specific drivers to examine why emergency diagnoses occur across diverse conditions, but at varying frequencies.
- Whilst patients diagnosed as an emergency are more likely to have worse outcomes, it is not yet known whether either preventing emergency diagnoses or improving the management of patients diagnosed as an emergency would lead to improved outcomes.
- Limitations include the assumption that recording of diagnoses and diagnosis dates are accurate, and that the definition of emergency diagnosis used may identify patients whose emergency care episode was unrelated to their as-yet-undiagnosed condition.

## Abstract 3

In a population-based cohort study, Emma Whitfield and colleagues investigate the frequency of emergency diagnoses across various non-neoplastic conditions and examine the association of emergency diagnosis with clinical outcomes in England.

## Introduction

Improving the timeliness and accuracy of medical diagnosis is a priority for contemporary healthcare [1]. Despite diagnosis being foundational to medicine, healthcare quality initiatives have traditionally focused on increasing treatment effectiveness and safety, rather than reducing the risks of delayed diagnosis.

System-level measures of the quality of the diagnostic process for patients with cancer have been developed or introduced in public health surveillance in some countries in recent years [2]. The diagnosis of cancer as an emergency (that is, through or soon after the use of emergency hospital care) is one such care quality measure [3,4]. Emergency diagnosis of cancer—occurring in around one in five patients with cancer in England—is strongly associated with worse prognosis even after adjustment for stage at diagnosis; international differences in cancer survival have been partly attributed to between-country variation in the proportion of patients diagnosed as emergencies [5–8]. While not all emergency diagnoses can be deemed avoidable, a key concern is that many emergency diagnoses arise from failures of the diagnostic process, within both general practice and secondary care diagnostic services [3,4,9,10].

While evidence on emergency diagnosis is concentrated on cancer, the challenges in achieving an accurate and timely diagnosis apply to all diseases. Disease-agnostic retrospective record review studies have documented diagnostic process failures in primary care, with 61% of safety incidents related to diagnostic errors, such as delayed referrals; absent, incorrect, or delayed investigations; and inaccurate documentation [11]. Furthermore, missed diagnostic opportunities occurred in ~4% of face-to-face consultations, for any complaint, in primary care [12]. More broadly, many of the hypothesised drivers of emergency diagnosis are unlikely to be unique to cancer [13,14].

In this study, we aimed to document the frequency of emergency diagnoses across various conditions and examine the association of emergency diagnosis with prognosis. Our broader aim was to provide evidence characterising key aspects of the phenomenon of emergency diagnosis beyond cancer, to enable future public health surveillance of diagnostic routes across disease areas, and to identify the potential for future interventions.

## Methods

This study is reported as per the REporting of studies Conducted using Observational Routinely-collected Data (RECORD) guideline (S1 Checklist) [15].

### Ethics statement

The protocol covering this study was approved by the Clinical Practice Research Datalink (CPRD) Research Data Governance Process of the UK Medicines and Healthcare Products Regulatory Agency (protocol number 22_002264), under Section 251 (National Health Service Act 2006). CPRD has ethical approval from the National Health Service (NHS) Health Research Authority to support research using anonymised CPRD data. As standard for research using ethically approved anonymised patient data (such as the CPRD data used in our study), individual patient consent was not required.

### Data sources

We used data from CPRD datasets, linked—using an eight step deterministic algorithm based on NHS number, sex, date of birth, and postcode—to Hospital Episode Statistics (HES) Admitted Patient Care (APC), Outpatient (OP), and Accident and Emergency (A&E) datasets, alongside Office for National Statistics (ONS) Death Registration, and patient postcode-level Index of Multiple Deprivation (IMD) data [16].

The CPRD is a database of anonymised routinely-collected primary care data from GP practices in the UK [17]. Two CPRD datasets were used in this study: CPRD GOLD, which collects data from contributing GP practices using Vision software, and CPRD Aurum which collects data from those using EMIS Web software [17,18]. Our analyses were stratified by CPRD dataset (GOLD/Aurum) to account for potential differences in recording between them.

HES datasets contain records of all inpatient admissions, outpatient appointments, and Accident and Emergency attendances at NHS hospitals in England [19]. All deaths occurring in England and Wales are recorded in the ONS Death Registration dataset.

### Study population

Given the exploratory nature of our study, we wanted to include diseases with a wide range of aetiologies for which challenges in the diagnostic process have been previously documented. Specifically, we aimed to include cardiac, respiratory, autoimmune, infectious, and neurological conditions where prior research indicated that prompt diagnosis may be challenging, and that earlier diagnosis could, in theory, be possible [20–23]. The 13 conditions comprised: axial spondyloarthritis (AS, formerly known during the study years as ankylosing spondylitis), coeliac disease, coronary/ischaemic heart disease (CHD), chronic obstructive pulmonary disease (COPD), inflammatory bowel disease (IBD), Lyme disease, multiple sclerosis (MS), Parkinson’s disease, polycystic ovary syndrome (PCOS), rheumatoid arthritis, schizophrenia and other chronic psychoses, subacute bacterial endocarditis (SBE), and tuberculosis (TB).

To supplement our analyses of non-neoplastic diseases, we also included five neoplasms (brain, colon, lung, ovary, and pancreatic cancer) to enable benchmarking against the existing literature. Phenotypes of Read V2 (CPRD GOLD), SNOMED CT (CPRD Aurum), ICD10 (HES, ONS), and ICD9 (ONS) codes used in the study are available at https://doi.org/10.5281/zenodo.13710755 [24].

This study was carried out as a parallel series of disease-specific cohort studies nested in electronic health record datasets. For each condition, case selection and analysis were carried out independently of all other conditions. Considering patients from practices consenting to data linkage, for each condition of interest we identified all patients with a first incident diagnosis between 1st January 1999 and 31st December 2019, who met reasonable disease-specific criteria (e.g., aged at least 30 at diagnosis of Parkinson’s disease) describing the population at risk of the condition of interest (see S1 Appendix). The study period was curtailed at 2019 as we assumed the COVID-19 pandemic could have had a substantial effect on diagnostic processes and pathways.

The diagnosis date was defined as the date of the patient’s earliest record of the condition across CPRD, HES, and ONS records. Patients with a diagnosis date before their registration date or the practice up-to-standard date, or more than 30 days after their ‘end date’ (the earliest of their ONS death date, transfer out date, and practice last collection date) were excluded. Complete details of the case selection process with flow diagrams for each condition are given in S1 Appendix.

### Emergency diagnoses and prognostic outcomes

Emergency hospital admissions were defined in HES APC as an inpatient admission coded with admission method (‘admimeth’) 21, 22, 23, 24, 25, 28, 2A, 2B, or 2D, in line with the contextual definition used by McPhail and colleague’s international study (on cancer emergency diagnosis) [5,25]. Each case was determined to have an emergency diagnosis if they had an emergency hospital admission starting on, or up to 30 days before, their diagnosis date.

We used two measures to investigate prognostic outcomes of an emergency diagnosis.

Death in the year after diagnosis was defined as an ONS death record with any cause of death, between 1 and 365 days after the diagnosis date.

The total duration of overnight hospital stays in the year after diagnosis was defined as the total number of nights the patient was recorded as being admitted to hospital in HES APC between 1 and 365 days after their diagnosis. We focused on number of nights spent in hospital rather than distinct admissions, as a means of focusing on disease severity/complexity, as for some of the included conditions hospital admissions are likely in both patients diagnosed as an emergency and patients who were not diagnosed as an emergency.

### Covariates

Year of diagnosis was defined as the year of the patient’s diagnosis date, and age at diagnosis as the difference between year of diagnosis and year of birth. IMD twentiles (2019) were provided by CPRD based on patients’ last recorded postcodes [26]. The diagnosis source was defined as the dataset (CPRD, HES APC, HES OP, or ONS) in which the patient’s diagnosis was first recorded.

Healthcare use increases with multimorbidity burden [27]. As morbidity scores based on hospital records are biased towards more severe morbidity, we used a morbidity score based on information from primary care records developed by Launders and colleagues—an index of the presence of 24 long-term conditions in patients’ records [28,29]. We calculated this comorbidity score at 6 months (180 days) prior to diagnosis. Unlike the Cambridge Multimorbidity Score, the score used does not require different lookback periods for different conditions, improving scalability [30].

For all conditions except PCOS and ovarian cancer, analyses were stratified by primary care-recorded gender (as declared by patients themselves, except when unable to do so [31]). As the number of patients with gender recorded as other, indeterminate, or unspecified was too small to report for any included condition, results are presented only for men and women.

### Statistical analyses

Analyses were initially planned in 2022 forming part of a broader protocol to examine the potential for earlier diagnosis in non-neoplastic conditions (S1 File). Sensitivity analyses 2 and 3 were not initially planned and were added post-hoc to account for the presence of patients who died very shortly after diagnosis and potential bias during the COVID-19 pandemic era, respectively.

The proportion of patients with each condition diagnosed as an emergency was reported, stratified by gender and CPRD dataset. Chi-squared tests with Yates’ continuity correction were used to examine whether the frequency of emergency diagnoses differed between men and women. For each condition, descriptive statistics of age at diagnosis, IMD twentile, comorbidity score, and diagnosis source were reported, stratified by emergency diagnosis-status.

The frequency of emergency diagnosis was plotted against year of diagnosis, age at diagnosis, and IMD twentile. As the number of patients diagnosed at each individual age was very small for some conditions, for visualisation purposes, age was categorised into <16, 5-year age bands from 16 to 80, 81–90, and >90.

We investigated prognostic outcomes of emergency diagnosis in the year post-diagnosis by fitting two regression models for each condition-gender-CPRD dataset combination. Model 1 evaluated the association between emergency diagnosis status and risk of death in the year post-diagnosis using logistic regression. Model 2 evaluated the association between emergency diagnosis status and total duration of overnight hospital stays in the year post-diagnosis using negative binomial regression. If a patient died in the year post-diagnosis they were still counted in the denominator for all 365 nights.

Age at diagnosis in years, year of diagnosis, IMD twentile, comorbidity score, and diagnosis source were included as covariates in both models. Patients with a death record on the diagnosis date or missing IMD twentile were not included in the models. Crude and adjusted odds/rate ratios are reported for each condition. Likelihood ratio tests were used to assess whether the association of emergency diagnosis with the outcome varied by gender within each CPRD dataset.

Analyses were carried out in Python (version 3.8.5), MySQL 8.0.41, and R (version 4.4.1) using packages including lmtest (v0.9-40), glmmTMB (v1.1.10), and several tidyverse (v2.0.0) packages [32–36]. Analysis scripts are available from https://doi.org/10.5281/zenodo.16840511 [37].

### Sensitivity analysis 1: A&E attendance

We expanded the definition of an emergency diagnosis to also include A&E attendance on the day of diagnosis or during the 30 days pre-diagnosis. This analysis was restricted to patients diagnosed from 1st May 2007 onwards, as HES A&E data were not available before April 2007. Key aspects of the main analysis were repeated.

### Sensitivity analysis 2: Adjusted hospitalisation risk

As a data-driven sensitivity analysis, we added an offset for number of days alive in the year after diagnosis in our models evaluating the association between emergency diagnosis status and the total duration of overnight hospital stays in the year after diagnosis (Model 2). This model adjusts for the presence of seriously ill patients who die very shortly after diagnosis, and who may be more incident in the emergency-diagnosed group.

### Sensitivity analysis 3: COVID-19 impact

We excluded patients diagnosed between 1st January 2019 and 31st December 2019 and repeated our analysis of prognostic outcomes of emergency diagnosis. This post-hoc analysis accounted for the potential impact of the COVID-19 pandemic on patient outcomes in the year after diagnosis for patients diagnosed in 2019.

### Patient and public involvement

We recruited six patient and public involvement (PPI) representatives with lived experience of a broad range of conditions, such as tuberculosis, axial spondyloarthritis, and cancer, as well as other conditions not included in this study, with whom we discussed the project’s possible patient involvement opportunities and priorities, pre-analysis. We agreed that opportunities for PPI were limited due to data protection restrictions and time constraints. PPI representatives suggested producing multimedia dissemination articles to increase accessibility and sharing of results and findings with a wider audience. Three PPI representatives took part in activities to co-produce a lay summary (S1 Appendix) and a video animation describing the study findings.

## Results

Overall, we identified 1,511,617 distinct patients from Aurum and 438,825 distinct patients from GOLD with an eligible new diagnosis of at least one condition (Table 1). Of these patients, 1,323,029 Aurum patients and 378,125 GOLD patients had an eligible diagnosis of at least one of the 13 non-neoplastic conditions included. Condition-specific samples varied substantially in size (being smallest for Lyme disease and highest for CHD in both CPRD data sources). Flowcharts of patient selection and patient characteristics stratified by emergency diagnosis status, CPRD dataset, and gender are given in S1 Appendix for each condition. Hereafter, where findings were highly concordant between the CPRD datasets, findings presented in-text relate to CPRD Aurum, unless otherwise stated. Corresponding results for GOLD and side-by-side comparisons of the main results are available in S1 Appendix.

**Table 1:** Patient characteristics of study population.

	Aurum	GOLD
	(*N* = 1,511,617)	(*N* = 438,825)
**Gender, *n* (%)**		
Men	733,466 (49%)	213,682 (49%)
Women	778,151 (51%)	225,143 (51%)
**Age in 2019**		
Mean (SD)	72.3 (20.1)	76.7 (19.2)
Median (IQR)	75.0 (60.0, 87.0)	80.0 (66.0, 91.0)
**Region, *n* (%)**		
East Midlands	41,372 (2.7%)	12,035 (2.7%)
East of England	69,499 (4.6%)	44,410 (10%)
London	231,238 (15%)	44,461 (10%)
North East	69,799 (4.6%)	10,622 (2.4%)
North West	311,998 (21%)	81,239 (19%)
South East	261,008 (17%)	115,872 (26%)
South West	204,117 (14%)	56,255 (13%)
West Midlands	251,536 (17%)	55,204 (13%)
Yorkshire and The Humber	69,929 (4.6%)	18,727 (4.3%)
None	1,121 (0.074%)	
**IMD quintile, *n* (%)**		
1 - Least deprived	276,049 (18%)	86,163 (20%)
2	287,519 (19%)	91,929 (21%)
3	288,013 (19%)	92,159 (21%)
4	314,358 (21%)	84,784 (19%)
5 - Most deprived	344,269 (23%)	83,350 (19%)
Missing	1,409 (0.093%)	440 (0.10%)
**Number of conditions**		
Mean (SD)	1.2 (0.4)	1.1 (0.4)
Median (IQR)	1.0 (1.0, 1.0)	1.0 (1.0, 1.0)

Note that patients are included only once, regardless of the number of conditions for which they are included in the sample. Age in 2019 is determined as (2019 – patient year of birth), regardless of the patient’s mortality status.

Abbreviations: SD, standard deviation; IQR, interquartile range; IMD, index of multiple deprivation.

### Frequency and characteristics of emergency diagnosis

**Fig 1:**
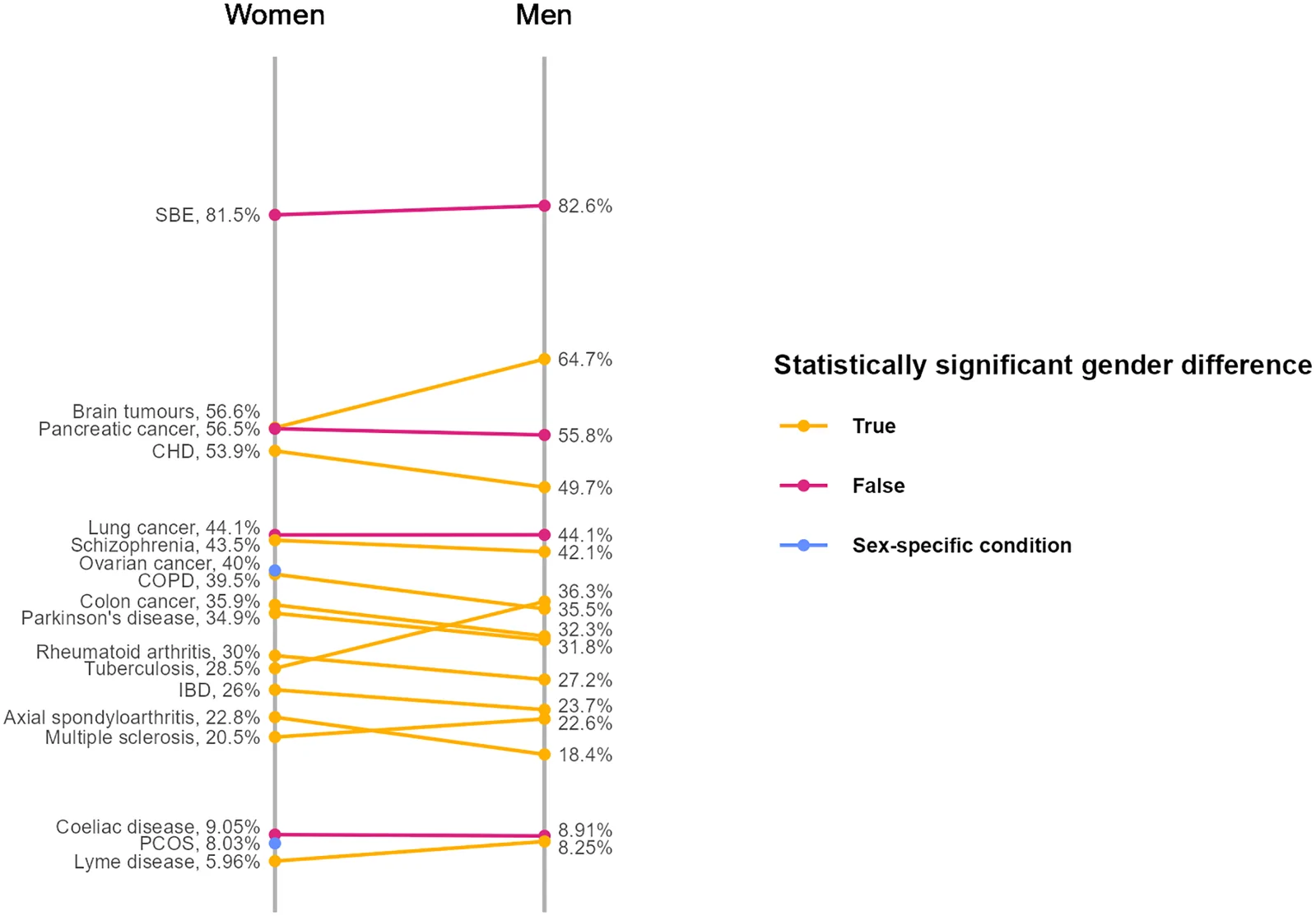
Slope plot of the frequency of emergency diagnosis by condition and gender – Aurum. Abbreviations: CHD, coronary/ischaemic heart disease; COPD, chronic obstructive pulmonary disease; IBD, inflammatory bowel disease; PCOS, polycystic ovary syndrome; SBE, subacute bacterial endocarditis.

The percentage of patients diagnosed as an emergency ranged from 8.3% (Lyme disease, 95% CI [7.2%, 9.4%]) to 83% (SBE, 95% CI [82%, 84%]) in men, and from 6.0% (Lyme disease, 95% CI [5.2%, 6.8%]) to 82% (SBE, 95% CI [80%, 83%]) in women (Fig 1, S1 Appendix). Differences in the frequency of emergency diagnosis between men and women were statistically significant at the 5% level in 12 out of 16 nonsex-specific conditions (10 out of 16 conditions in GOLD), of which emergency diagnoses were more frequent amongst women in 8 (7, GOLD) conditions, respectively. However, the largest absolute differences in emergency diagnosis frequency occurred in conditions for which men experienced emergency diagnoses more frequently than women (brain tumours, TB).

Characteristics of patients diagnosed as an emergency were largely consistent across the conditions for both men and women (S1 Appendix)—patients diagnosed as an emergency were older and slightly more comorbid than patients who were not diagnosed as an emergency. In general, a higher proportion of patients with their diagnosis first captured in inpatient care records (HES APC) were diagnosed as an emergency than those with their diagnosis first captured in another data source (primary care, outpatient care, death records).

**Fig 2:**
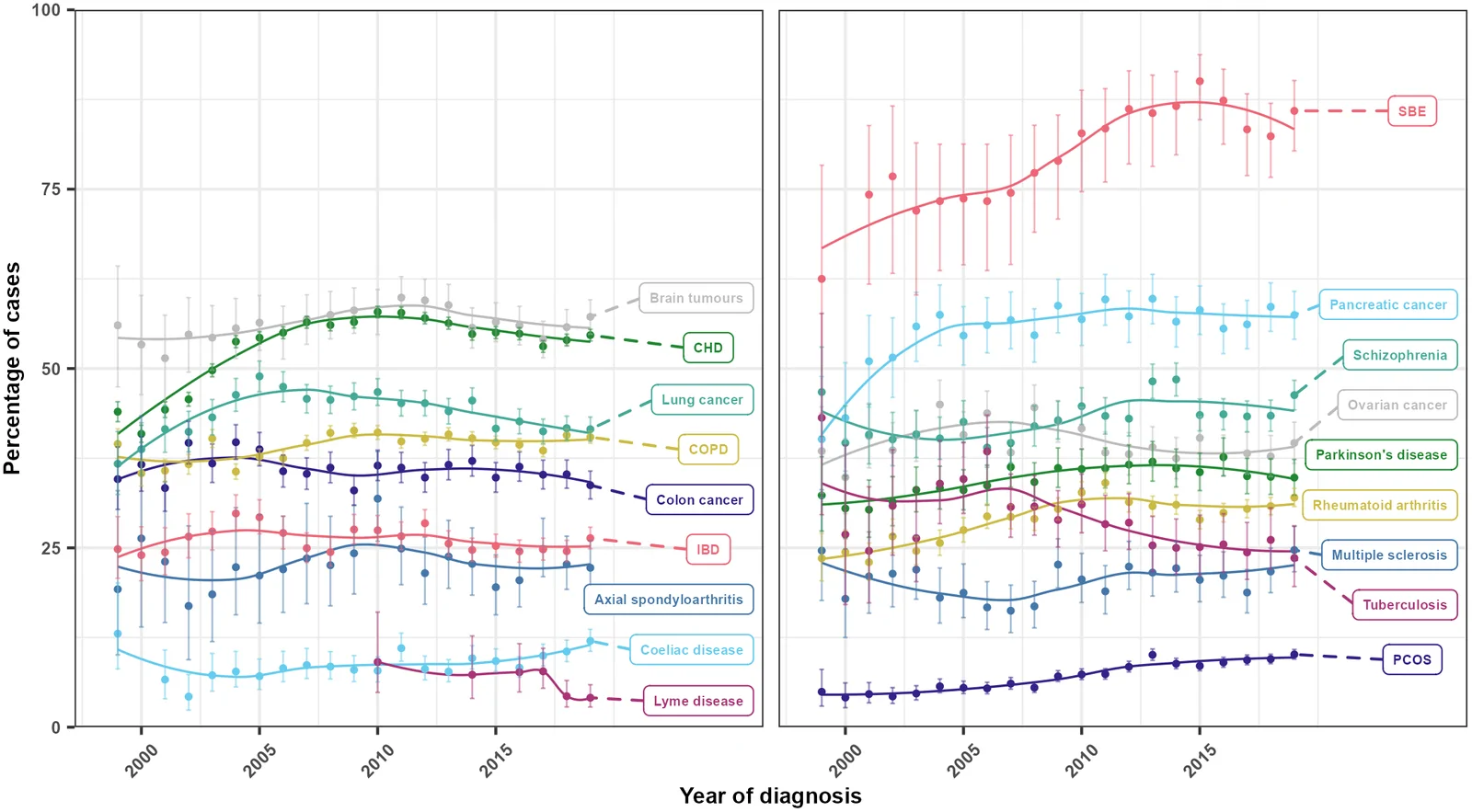
Percentage of patients diagnosed following an emergency presentation (emergency hospital admission) by year of diagnosis – Aurum, Women. Note that only years in which 10 patients were diagnosed as an emergency and 10 patients were not diagnosed as an emergency are shown. Lines are loess curves indicative of general trends over time. Conditions are split alphabetically across panels for visual clarity. Abbreviations: CHD, coronary/ischaemic heart disease; COPD, chronic obstructive pulmonary disease; IBD, inflammatory bowel disease; PCOS, polycystic ovary syndrome; SBE, subacute bacterial endocarditis.

**Fig 3:**
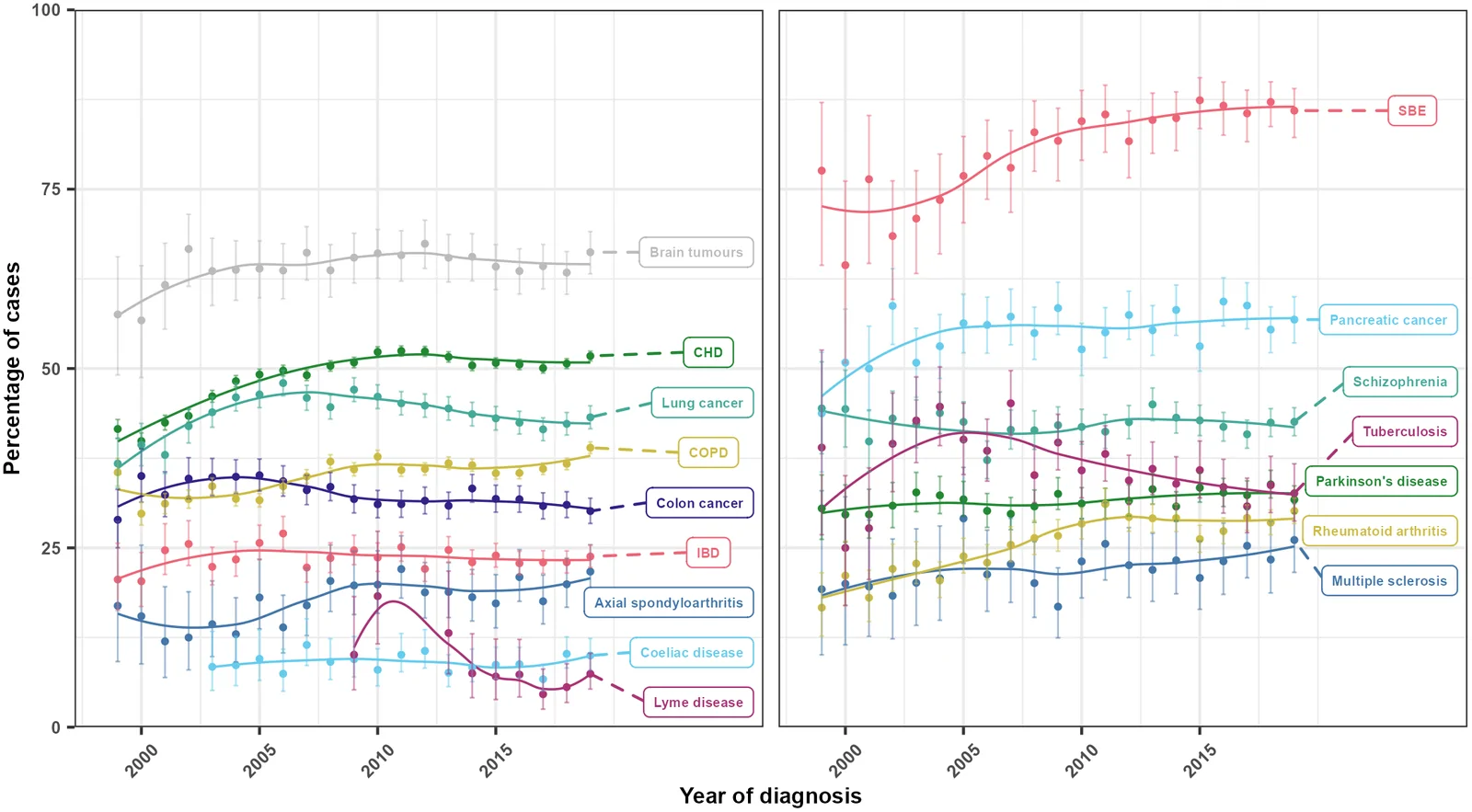
Percentage of patients diagnosed following an emergency presentation (emergency hospital admission) by year of diagnosis – Aurum, Men. Note that only years in which 10 patients were diagnosed as an emergency and 10 patients were not diagnosed as an emergency are shown. Lines are loess curves indicative of general trends over time. Conditions are split alphabetically across panels for visual clarity. Abbreviations: CHD, coronary/ischaemic heart disease; COPD, chronic obstructive pulmonary disease; IBD, inflammatory bowel disease; SBE, subacute bacterial endocarditis.

Condition-specific time trends in the percentage of patients diagnosed as an emergency were generally similar between genders (Figs 2 and 3, S1 Appendix, S1 Data), except for some condition-specific peaks that only occurred in either men or women and a stronger increase in some conditions at the end of the study period in GOLD.

**Fig 4:**
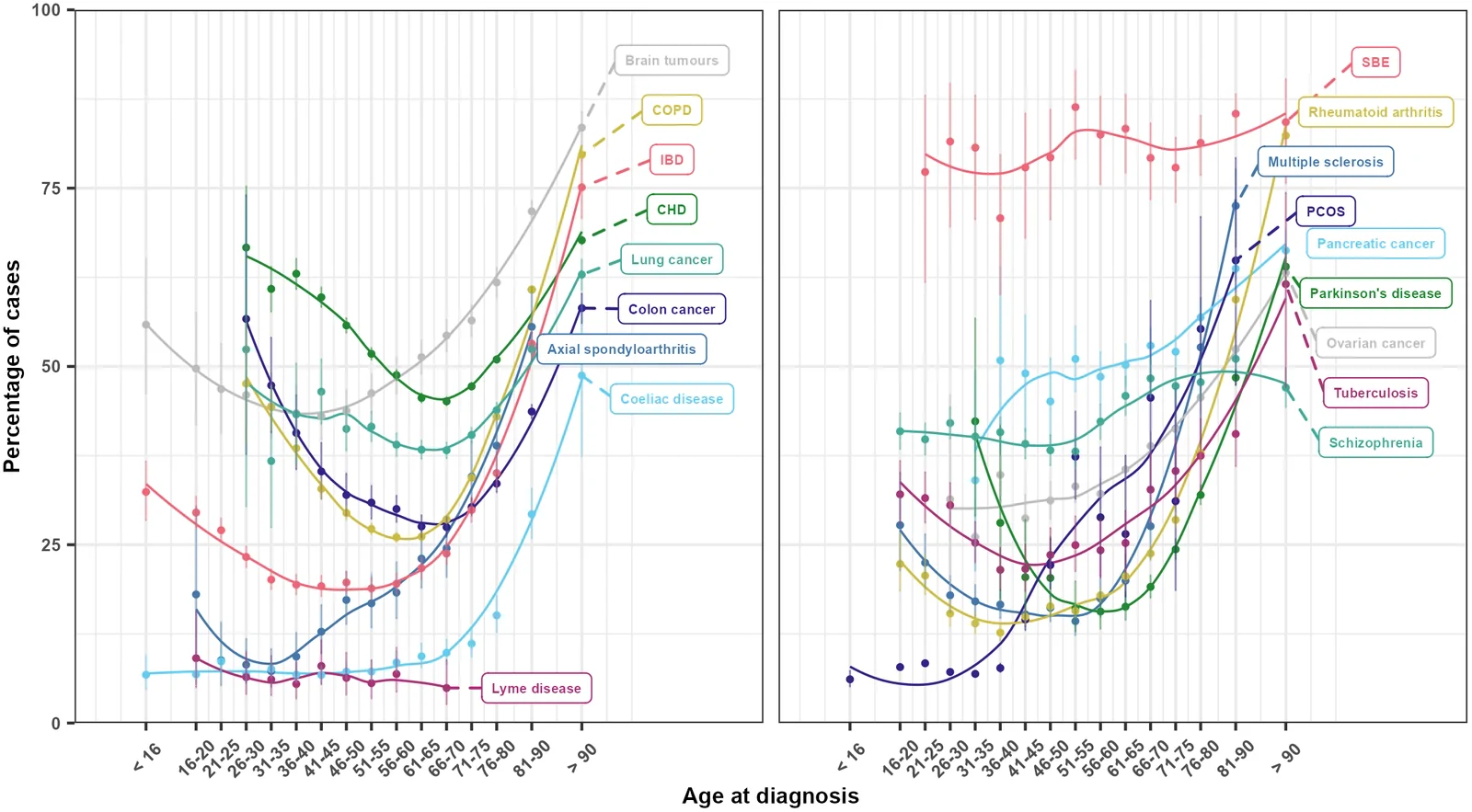
Percentage of patients diagnosed following an emergency presentation (emergency hospital admission) by age at diagnosis – Aurum, Women. Note that only age groups with at least 10 patients diagnosed as an emergency and 10 patients not diagnosed as an emergency are shown. Lines are loess curves indicative of general patterns over age groups. Conditions are split alphabetically across the two panels for visual clarity. Abbreviations: CHD, coronary/ischaemic heart disease; COPD, chronic obstructive pulmonary disease; IBD, inflammatory bowel disease; PCOS, polycystic ovary syndrome; SBE, subacute bacterial endocarditis.

**Fig 5:**
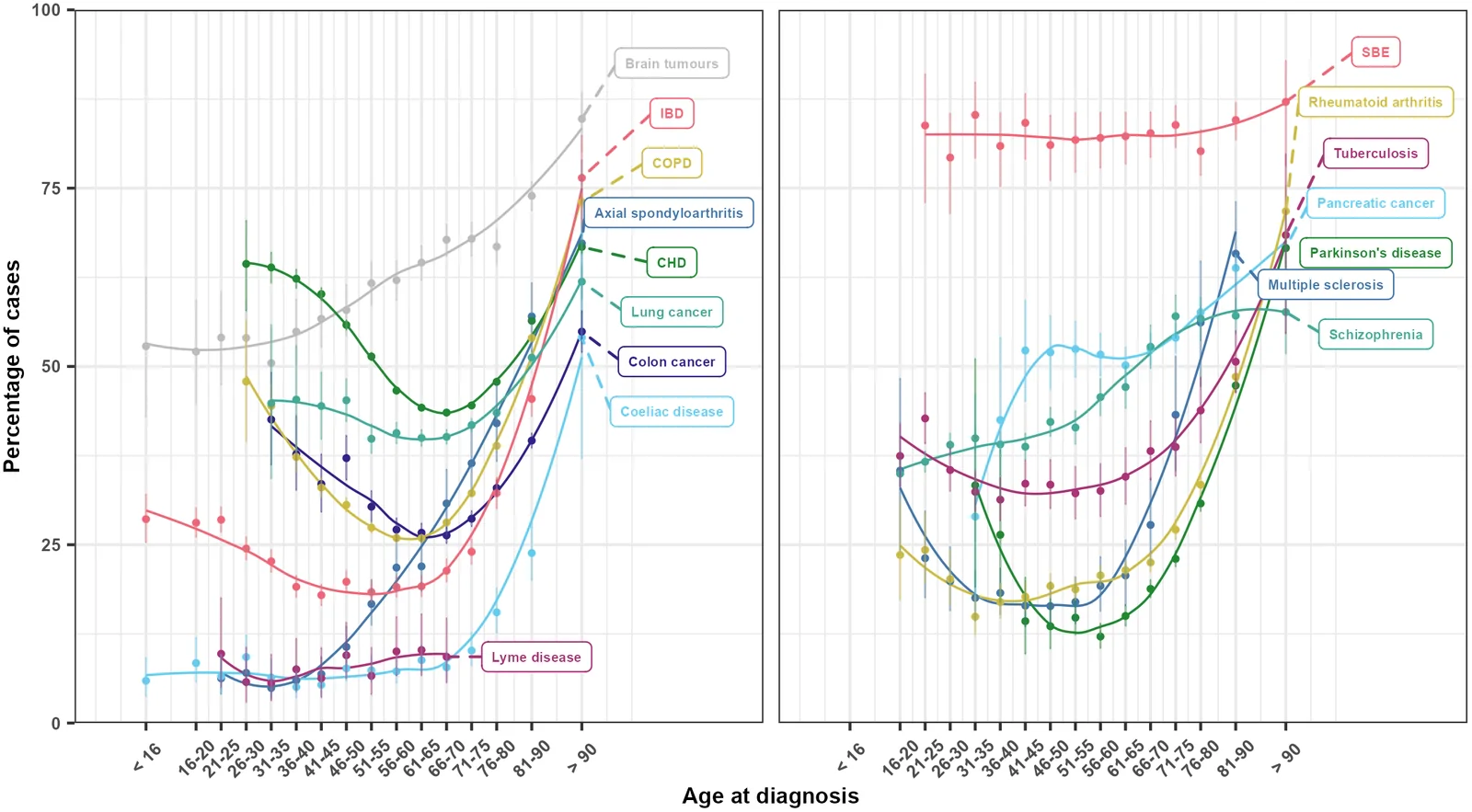
Percentage of patients diagnosed following an emergency presentation (emergency hospital admission) by age at diagnosis – Aurum, Men. Note that only age groups with at least 10 patients diagnosed as an emergency and 10 patients not diagnosed as an emergency are shown. Lines are loess curves indicative of general patterns over age groups. Conditions are split alphabetically across the two panels for visual clarity. Abbreviations: CHD, coronary/ischaemic heart disease; COPD, chronic obstructive pulmonary disease; IBD, inflammatory bowel disease; SBE, subacute bacterial endocarditis.

**Fig 6:**
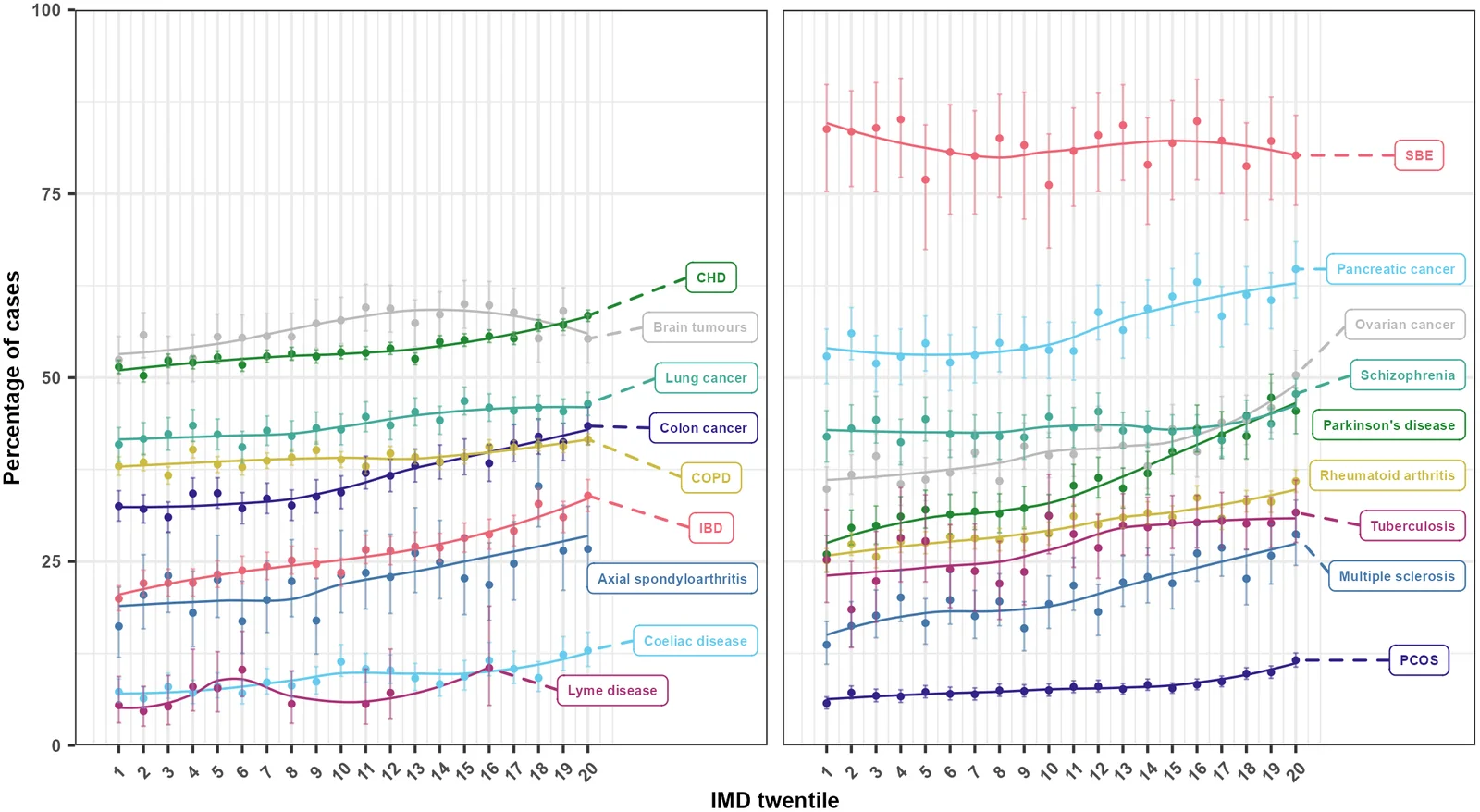
Percentage of patients diagnosed following an emergency presentation (emergency hospital admission) by IMD twentile – Aurum, Women. 1 is least deprived, 20 is most deprived. Note that only twentiles with at least 10 patients diagnosed as an emergency and 10 patients were not diagnosed as an emergency are shown. Lines are loess curves indicative of general patterns over twentiles. Conditions are split alphabetically across the two panels for visual clarity. Abbreviations: CHD, coronary/ischaemic heart disease; COPD, chronic obstructive pulmonary disease; IBD, inflammatory bowel disease; PCOS, polycystic ovary syndrome; SBE, subacute bacterial endocarditis.

**Fig 7:**
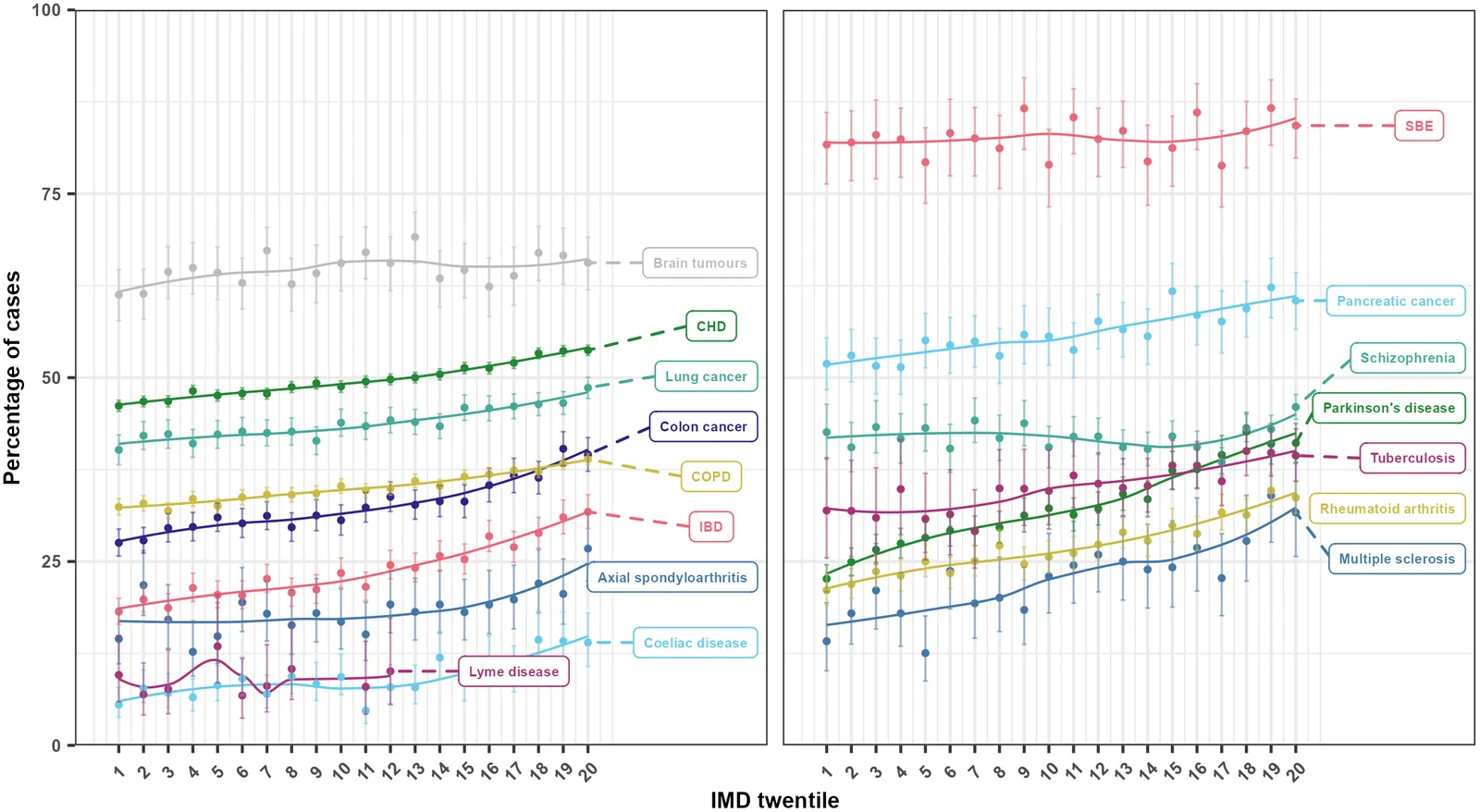
Percentage of patients diagnosed following an emergency presentation (emergency hospital admission) by IMD twentile – Aurum, Men. 1 is least deprived, 20 is most deprived. Note that only twentiles with at least 10 patients diagnosed as an emergency and 10 patients not diagnosed as an emergency are shown. Lines are loess curves indicative of general patterns over twentiles. Conditions are split alphabetically across the two panels for visual clarity. Abbreviations: CHD, coronary/ischaemic heart disease; COPD, chronic obstructive pulmonary disease; IBD, inflammatory bowel disease; SBE, subacute bacterial endocarditis.

The percentage of emergency diagnoses by age followed a U- or J-shaped curve for most conditions. The inflection point (age) of U-/J-shaped associations varied—for example, 66–70 in CHD versus 31–35 in AS. The exceptions to this pattern were Lyme disease, SBE, schizophrenia, and pancreatic cancer, where no consistent patterns of association between emergency diagnosis and age were observed (Figs 4 and 5, S1 Appendix, S1 Data). The percentage of patients diagnosed as an emergency was higher in patients living in areas with higher levels of deprivation for most conditions (Figs 6 and 7, S1 Appendix, S1 Data).

### Association with prognosis

Amongst the 13 non-neoplastic conditions, 1-year mortality in patients who were not diagnosed as an emergency ranged from 0.04% in PCOS to 24% in women diagnosed with SBE. In contrast, in patients diagnosed as an emergency, we observed a corresponding range of 0.5% in PCOS to 40% in men with Parkinson’s disease. There were large differences in 1-year mortality post-diagnosis between patients who were and were not diagnosed as an emergency in almost every condition, including axial spondyloarthritis (20% versus 1.6%, men), coeliac disease (11% versus 0.7%, women), and multiple sclerosis (15% versus 0.9%, men). This difference was 10% or higher in both men and women in 9 of the 13 non-neoplastic conditions examined.

**Fig 8:**
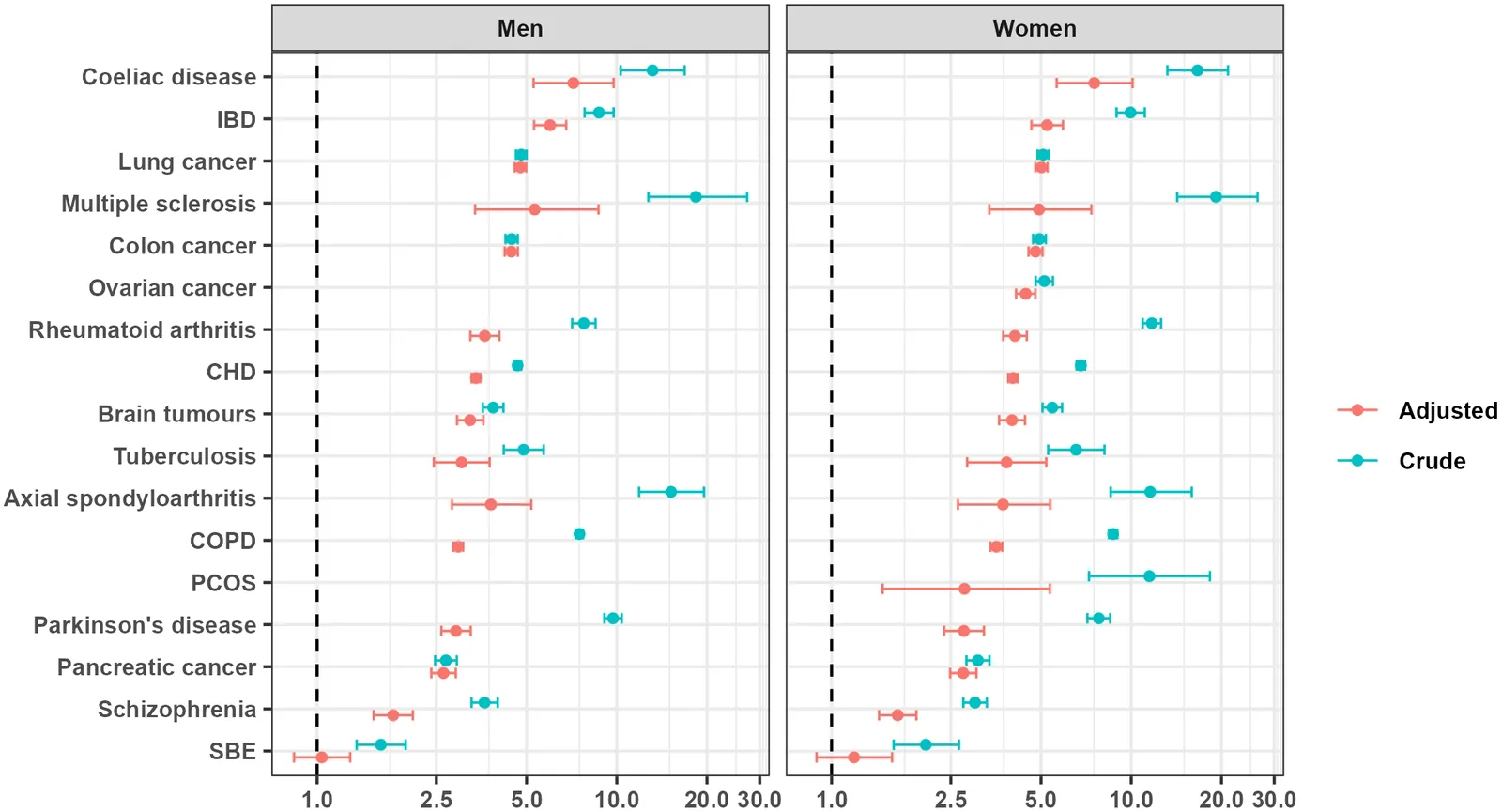
Forest plot of odds ratios for 1-year mortality following an emergency diagnosis – Aurum. Note that only gender-condition combinations with at least 10 patients diagnosed as an emergency who died in the year after diagnosis were examined—consequently, Lyme disease is not included. Odds ratios were adjusted for age at diagnosis, year of diagnosis, IMD score, comorbidity score 6 months (180 days) prior to diagnosis and diagnosis source. Abbreviations: CHD, coronary/ischaemic heart disease; COPD, chronic obstructive pulmonary disease; IBD, inflammatory bowel disease; PCOS, polycystic ovary syndrome; SBE, subacute bacterial endocarditis.

Both crude and adjusted odds ratios further indicated a clear and consistent association between emergency diagnosis and 1-year mortality. Adjusted odds ratios (aORs) for 1-year mortality following an emergency diagnosis ranged from 1.04 (SBE, 95% CI [0.837, 1.29]) to 7.16 (coeliac disease, 95% CI [5.28, 9.76]) in men, and from 1.19 (SBE, 95% CI [0.891, 1.59]) to 7.53 (coeliac disease, 95% CI [5.64, 10.1]) in women (Fig 8, Table 2, S1 Appendix). Adjusted ORs for 1-year mortality following an emergency diagnosis exceeded 2.5 for men and women for all but three conditions in Aurum (Lyme disease – for which the small sample size prevented calculation, SBE, and schizophrenia), and all but six conditions in GOLD (Lyme disease, PCOS – for both of which the small sample size prevented calculation, MS – women only, SBE, schizophrenia, and tuberculosis). The association of emergency diagnosis status with mortality differed by gender at the 5% level in four conditions in Aurum (brain tumours, CHD, colon cancer, and COPD) and one condition in GOLD (CHD).

**Table 2:** Observed 1-year mortality and odds ratios for 1-year mortality in patients who were and were not diagnosed as an emergency by condition and gender – Aurum.

Condition	Gender	Emergency diagnosed		Non-emergency diagnosed		Crude OR (95% CI)	Adjusted^b^ OR (95% CI)	*p*-value^c^
		*N* patients^a^	1-year mortality, % (*n*)	*N* patients	1-year mortality, % (*n*)			
Axial spondyloarthritis	Men	1,265	20% (253)	5,600	1.6% (91)	15.2 (11.9, 19.5)	3.80 (2.82, 5.18)	0.939
	Women	1,145	14% (166)	3,880	1.4% (56)	11.6 (8.54, 15.9)	3.73 (2.64, 5.36)	
Brain tumours	Men	8,845	56% (4,910)	4,620	24% (1,127)	3.87 (3.57, 4.19)	3.24 (2.93, 3.59)	< 0.01
	Women	10,620	45% (4,831)	7,915	13% (1,051)	5.45 (5.06, 5.88)	4.00 (3.62, 4.42)	
Coeliac disease	Men	825	18% (147)	8,480	1.6% (137)	13.2 (10.3, 16.8)	7.16 (5.28, 9.76)	0.818
	Women	1,725	11% (185)	17,450	0.72% (125)	16.6 (13.2, 21.0)	7.53 (5.64, 10.1)	
CHD	Men	162,500	19% (31,205)	130,335	4.8% (6,316)	4.67 (4.54, 4.80)	3.39 (3.28, 3.50)	< 0.01
	Women	133,265	26% (35,085)	90,805	5.0% (4,543)	6.79 (6.57, 7.01)	4.03 (3.88, 4.18)	
Colon cancer	Men	12,435	51% (6,305)	26,500	19% (4,968)	4.46 (4.25, 4.67)	4.44 (4.23, 4.67)	0.0439
	Women	12,340	53% (6,559)	21,915	19% (4,094)	4.94 (4.70, 5.19)	4.79 (4.54, 5.06)	
COPD	Men	71,255	33% (23,715)	128,515	6.2% (8,007)	7.51 (7.30, 7.72)	2.96 (2.85, 3.07)	< 0.01
	Women	72,340	29% (21,191)	110,340	4.5% (5,014)	8.70 (8.43, 8.99)	3.55 (3.39, 3.71)	
IBD	Men	8,555	13% (1,106)	27,675	1.7% (463)	8.73 (7.81, 9.76)	5.99 (5.30, 6.78)	0.129
	Women	10,450	13% (1,385)	29,875	1.5% (452)	9.94 (8.93, 11.1)	5.24 (4.65, 5.92)	
Lung cancer	Men	25,735	85% (21,792)	31,990	54% (17,121)	4.80 (4.61, 5.00)	4.77 (4.57, 4.98)	0.123
	Women	20,980	81% (17,029)	25,760	46% (11,819)	5.08 (4.87, 5.30)	5.02 (4.79, 5.26)	
Lyme disease	Men	200	–	2,225	0.63% (14)			
	Women	195	–	3,060	–			
Multiple sclerosis	Men	1,060	15% (157)	3,630	0.94% (34)	18.4 (12.8, 27.2)	5.32 (3.37, 8.69)	0.802
	Women	2,120	11% (226)	8,245	0.62% (51)	19.2 (14.2, 26.4)	4.92 (3.36, 7.36)	
Ovarian cancer	Women	7,435	53% (3,924)	11,120	18% (1,992)	5.12 (4.80, 5.48)	4.44 (4.13, 4.78)	
Pancreatic cancer	Men	7,125	83% (5,903)	5,275	64% (3,391)	2.69 (2.48, 2.93)	2.64 (2.40, 2.90)	0.563
	Women	6,995	85% (5,951)	4,825	65% (3,133)	3.08 (2.82, 3.36)	2.75 (2.49, 3.04)	
Parkinson’s disease	Men	10,485	40% (4,158)	22,345	6.3% (1,415)	9.72 (9.09, 10.4)	2.91 (2.60, 3.25)	0.607
	Women	7,860	30% (2,385)	14,390	5.3% (762)	7.79 (7.14, 8.50)	2.77 (2.38, 3.22)	
PCOS	Women	7,120	0.51% (36)	81,530	0.044% (36)	11.5 (7.23, 18.3)	2.78 (1.48, 5.35)	
Rheumatoid arthritis	Men	8,840	20% (1,778)	23,715	3.1% (745)	7.76 (7.10, 8.49)	3.63 (3.25, 4.06)	0.101
	Women	21,060	20% (4,166)	49,260	2.1% (1,016)	11.7 (10.9, 12.6)	4.09 (3.74, 4.48)	
SBE	Men	4,100	31% (1,274)	730	22% (158)	1.63 (1.36, 1.98)	1.04 (0.837, 1.29)	0.461
	Women	1,980	39% (778)	385	24% (92)	2.06 (1.61, 2.66)	1.19 (0.891, 1.59)	
Schizophrenia	Men	13,230	10% (1,383)	18,150	3.1% (567)	3.62 (3.28, 4.01)	1.79 (1.54, 2.09)	0.471
	Women	12,425	13% (1,598)	16,115	4.7% (753)	3.01 (2.75, 3.30)	1.66 (1.44, 1.92)	
Tuberculosis	Men	3,475	17% (598)	6,225	4.1% (254)	4.89 (4.20, 5.70)	3.04 (2.45, 3.76)	0.217
	Women	2,170	13% (286)	5,560	2.3% (126)	6.54 (5.28, 8.14)	3.84 (2.83, 5.20)	

^a^To prevent backwards calculation of small patient groups the number of patients has been reported rounded to 5.

^b^Adjusted for age at diagnosis, year of diagnosis, IMD score, comorbidity score 6 months (180 days) prior to diagnosis and diagnosis source.

^c^Note that *p*-values relate to the null hypothesis that there was no difference in the adjusted OR between men and women tested using likelihood ratio tests.

“–” indicates that fewer than 10 patients died within 1 year of diagnosis. Odds ratios were not calculated for these conditions as the results are unlikely to be reliable. Abbreviations: CI, confidence interval; OR, odds ratio; CHD, coronary/ischaemic heart disease; COPD, chronic obstructive pulmonary disease; IBD, inflammatory bowel disease; PCOS, polycystic ovary syndrome; SBE, subacute bacterial endocarditis.

**Fig 9:**
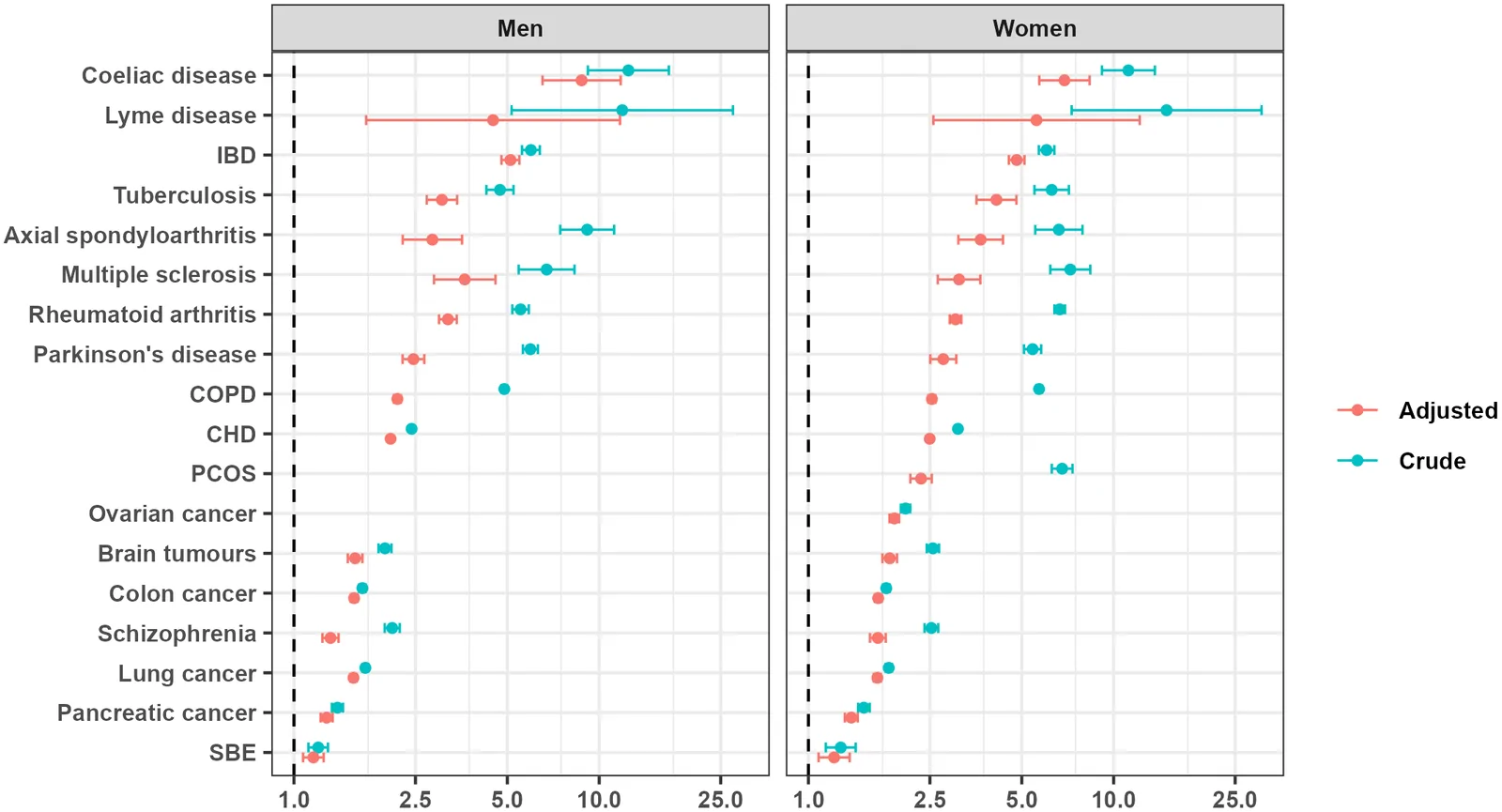
Forest plot of rate ratios for time in hospital in the year post-diagnosis following an emergency diagnosis – Aurum. Rate ratios were adjusted for age at diagnosis, year of diagnosis, IMD score, comorbidity score 6 months (180 days) prior to diagnosis and diagnosis source. Abbreviations: CHD, coronary/ischaemic heart disease; COPD, chronic obstructive pulmonary disease; IBD, inflammatory bowel disease; PCOS, polycystic ovary syndrome; SBE, subacute bacterial endocarditis.

The mean duration of overnight hospital stays in the year after diagnosis ranged from 0.5 (Lyme disease, Women) to 38.0 days (SBE, Men) in patients who were not diagnosed as an emergency (Table 3, S1 Appendix). Amongst patients diagnosed as an emergency, the mean duration ranged from 4.5 (PCOS, Women) to 47.5 days (SBE, Women). Adjusted rate ratios (aRRs) for total duration of overnight hospital stays in the year after diagnosis following an emergency diagnosis ranged from 1.16 (SBE, 95% CI [1.07, 1.25]) to 8.76 (coeliac disease, 95% CI [6.52, 11.8]) in men, and from 1.21 (SBE, 95% CI [1.08, 1.36]) to 6.90 (coeliac disease, 95% CI [5.70, 8.34]) in women (Fig 9, Table 3, S1 Appendix).

**Table 3:** Observed mean number of hospital inpatient nights in year after diagnosis and rate ratios for duration of hospital inpatient nights in year after diagnosis in patients who were and were not diagnosed as an emergency by condition and gender – Aurum.

Condition	Gender	Emergency diagnosed			Non-emergency diagnosed			Crude RR (95% CI)	Adjusted^c^ RR (95% CI)	*p*-value^d^
		*N* patients^a^	Admitted, % (*n*)^b^	Mean admitted duration	*N* patients^a^	Admitted, % (*n*)^b^	Mean admitted duration			
Axial spondyloarthritis	Men	1,265	78% (993)	21.9	5,600	16% (902)	2.4	9.13 (7.46, 11.2)	2.84 (2.27, 3.55)	0.0679
	Women	1,145	76% (869)	17.8	3,880	25% (973)	2.7	6.61 (5.53, 7.90)	3.67 (3.10, 4.34)	
Brain tumours	Men	8,845	93.6% (8,281)	27.3	4,620	62% (2,878)	13.8	1.99 (1.89, 2.09)	1.59 (1.50, 1.68)	< 0.01
	Women	10,620	91.2% (9,689)	27.2	7,915	51% (4,063)	10.6	2.56 (2.44, 2.68)	1.85 (1.75, 1.95)	
Coeliac disease	Men	825	72% (596)	18.8	8,480	12% (1,025)	1.5	12.5 (9.18, 16.9)	8.76 (6.52, 11.8)	0.171
	Women	1,725	69% (1,193)	13.6	17,450	13% (2,248)	1.2	11.2 (9.15, 13.6)	6.90 (5.70, 8.34)	
CHD	Men	162,500	87% (140,818)	14.3	130,335	47% (61,077)	5.9	2.43 (2.40, 2.46)	2.07 (2.04, 2.10)	< 0.01
	Women	133,265	85% (113,712)	18.4	90,805	41% (37,238)	6.0	3.09 (3.04, 3.14)	2.50 (2.45, 2.54)	
Colon cancer	Men	12,435	94.7% (11,773)	24.2	26,500	85% (22,537)	14.4	1.68 (1.64, 1.72)	1.57 (1.54, 1.61)	< 0.01
	Women	12,340	95.2% (11,743)	25.4	21,915	85% (18,737)	14.1	1.80 (1.75, 1.84)	1.69 (1.65, 1.74)	
COPD	Men	71,255	86% (61,566)	20.4	128,515	28% (35,489)	4.2	4.89 (4.79, 4.99)	2.18 (2.13, 2.24)	< 0.01
	Women	72,340	87% (62,651)	21.1	110,340	26% (28,437)	3.7	5.70 (5.58, 5.81)	2.54 (2.47, 2.60)	
IBD	Men	8,555	82% (7,029)	17.9	27,675	21% (5,942)	3.0	5.97 (5.59, 6.39)	5.12 (4.78, 5.48)	0.174
	Women	10,450	83% (8,719)	18.9	29,875	23% (6,974)	3.1	6.03 (5.69, 6.39)	4.81 (4.54, 5.10)	
Lung cancer	Men	25,735	92.2% (23,740)	19.3	31,990	73% (23,317)	11.3	1.71 (1.68, 1.75)	1.57 (1.53, 1.60)	< 0.01
	Women	20,980	92.6% (19,437)	20.1	25,760	72% (18,490)	11.0	1.83 (1.79, 1.88)	1.68 (1.64, 1.73)	
Lyme disease	Men	200	52% (104)	8.1	2,225	5.8% (128)	0.7	11.9 (5.16, 27.4)	4.49 (1.72, 11.7)	0.713
	Women	195	52% (101)	6.9	3,060	7.7% (235)	0.5	14.9 (7.28, 30.5)	5.59 (2.57, 12.2)	
Multiple sclerosis	Men	1,060	76% (808)	19.0	3,630	19% (690)	2.8	6.73 (5.45, 8.30)	3.63 (2.88, 4.57)	0.292
	Women	2,120	74% (1,566)	16.7	8,245	18% (1,505)	2.3	7.21 (6.20, 8.38)	3.11 (2.65, 3.66)	
Ovarian cancer	Women	7,435	94.6% (7,036)	22.3	11,120	81% (9,021)	10.7	2.08 (2.01, 2.15)	1.91 (1.84, 1.98)	
Pancreatic cancer	Men	7,125	93.5% (6,660)	19.7	5,275	76% (4,004)	14.2	1.39 (1.33, 1.45)	1.28 (1.22, 1.34)	0.0236
	Women	6,995	93.8% (6,559)	20.9	4,825	73% (3,534)	13.7	1.52 (1.46, 1.59)	1.38 (1.32, 1.45)	
Parkinson’s disease	Men	10,485	91.5% (9,599)	35.9	22,345	26% (5,703)	6.0	5.95 (5.62, 6.30)	2.46 (2.27, 2.67)	0.0776
	Women	7,860	90.6% (7,124)	34.2	14,390	26% (3,693)	6.3	5.42 (5.08, 5.79)	2.77 (2.51, 3.05)	
PCOS	Women	7,120	59% (4,204)	4.5	81,530	16% (12,956)	0.7	6.78 (6.27, 7.33)	2.34 (2.16, 2.54)	
Rheumatoid arthritis	Men	8,840	80% (7,043)	18.2	23,715	26% (6,278)	3.3	5.52 (5.19, 5.88)	3.19 (2.99, 3.41)	0.201
	Women	21,060	81% (17,027)	20.3	49,260	25% (12,465)	3.0	6.66 (6.40, 6.93)	3.03 (2.90, 3.17)	
SBE	Men	4,100	98.2% (4,026)	45.6	730	80% (587)	38.0	1.20 (1.12, 1.29)	1.16 (1.07, 1.25)	0.497
	Women	1,980	97.0% (1,921)	47.5	385	77% (297)	37.2	1.28 (1.14, 1.43)	1.21 (1.08, 1.36)	
Schizophrenia	Men	13,230	82% (10,786)	40.6	18,150	31% (5,675)	19.3	2.10 (1.98, 2.22)	1.32 (1.24, 1.40)	< 0.01
	Women	12,425	87% (10,826)	44.2	16,115	38% (6,075)	17.5	2.53 (2.40, 2.66)	1.69 (1.59, 1.79)	
Tuberculosis	Men	3,475	85% (2,941)	22.6	6,225	23% (1,441)	4.8	4.73 (4.27, 5.24)	3.05 (2.72, 3.42)	< 0.01
	Women	2,170	85% (1,840)	22.2	5,560	22% (1,247)	3.5	6.26 (5.50, 7.13)	4.13 (3.55, 4.80)	

^a^To prevent backwards calculation of small patient groups the number of patients has been reported rounded to 5.

^b^Percentage (number) of patients admitted for at least one night in the year after diagnosis.

^c^Adjusted for age at diagnosis, year of diagnosis, IMD score, comorbidity score 6 months (180 days) prior to diagnosis and diagnosis source.

^d^Note that *p*-values relate to the null hypothesis that there was no difference in the adjusted RR between men and women tested using likelihood ratio tests.

Abbreviations: CI, confidence interval; RR, rates ratio; CHD, coronary/ischaemic heart disease; COPD, chronic obstructive pulmonary disease; IBD, inflammatory bowel disease; PCOS, polycystic ovary syndrome; SBE, subacute bacterial endocarditis.

### Overview of key findings

**Fig 10:**
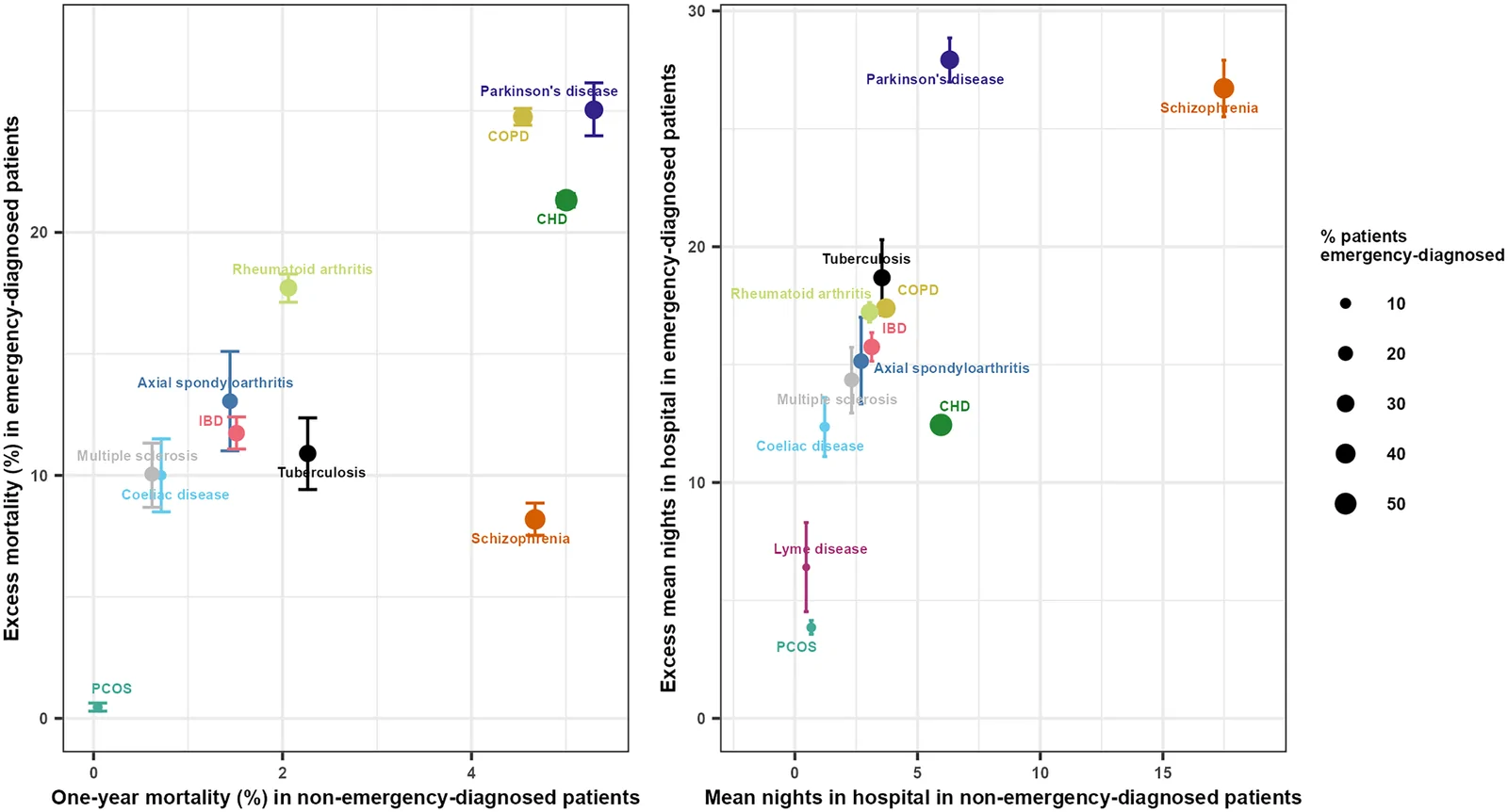
Excess outcomes in the year post-diagnosis following emergency diagnosis of non-neoplastic conditions – Aurum, Women. Excess defined as the ‘mean in patients diagnosed as an emergency’ minus the ‘mean in patients who were not diagnosed as an emergency’. 95% confidence intervals for excess outcomes were calculated using bootstrapping with a normal approximation. Subacute bacterial endocarditis not plotted for visual clarity; its 1-year mortality in patients who were not diagnosed as an emergency was 24% with 15% excess mortality in patients diagnosed as an emergency; mean of 37.2 hospital nights in patients who were not diagnosed as an emergency with 10.3 excess nights in patients diagnosed as an emergency. Abbreviations: CHD, coronary/ischaemic heart disease; COPD, chronic obstructive pulmonary disease; IBD, inflammatory bowel disease; PCOS, polycystic ovary syndrome. For underlying values, please see S1 Data.

Key results concerning the frequency and prognostic outcomes of emergency diagnosis are summarised in Table 4 and Fig 10.

**Table 4:** Overview of results - Aurum.

Condition	Gender	*N*	% emergency diagnosed (95% CI)	Adjusted^a^ OR (95% CI) for 1-year mortality	Adjusted^a^ RR (95% CI) for time in hospital
Axial spondyloarthritis	Men	6,889	18% (18%, 19%)	3.80 (2.82, 5.18)	2.84 (2.27, 3.55)
	Women	5,042	23% (22%, 24%)	3.73 (2.64, 5.36)	3.67 (3.10, 4.34)
Brain tumours	Men	13,930	65% (64%, 65%)	3.24 (2.93, 3.59)	1.59 (1.50, 1.68)
	Women	19,026	57% (56%, 57%)	4.00 (3.62, 4.42)	1.85 (1.75, 1.95)
Coeliac disease	Men	9,320	8.9% (8.3%, 9.5%)	7.16 (5.28, 9.76)	8.76 (6.52, 11.8)
	Women	19,209	9.1% (8.7%, 9.5%)	7.53 (5.64, 10.1)	6.90 (5.70, 8.34)
CHD	Men	349,085	50% (50%, 50%)	3.39 (3.28, 3.50)	2.07 (2.04, 2.10)
	Women	267,971	54% (54%, 54%)	4.03 (3.88, 4.18)	2.50 (2.45, 2.54)
Colon cancer	Men	41,149	32% (32%, 33%)	4.44 (4.23, 4.67)	1.57 (1.54, 1.61)
	Women	36,565	36% (35%, 36%)	4.79 (4.54, 5.06)	1.69 (1.65, 1.74)
COPD	Men	206,997	36% (35%, 36%)	2.96 (2.85, 3.07)	2.18 (2.13, 2.24)
	Women	187,461	40% (39%, 40%)	3.55 (3.39, 3.71)	2.54 (2.47, 2.60)
IBD	Men	36,321	24% (23%, 24%)	5.99 (5.30, 6.78)	5.12 (4.78, 5.48)
	Women	40,456	26% (26%, 26%)	5.24 (4.65, 5.92)	4.81 (4.54, 5.10)
Lung cancer	Men	62,556	44% (44%, 45%)	4.77 (4.57, 4.98)	1.57 (1.53, 1.60)
	Women	50,512	44% (44%, 45%)	5.02 (4.79, 5.26)	1.68 (1.64, 1.73)
Lyme disease	Men	2,423	8.3% (7.2%, 9.4%)	–	4.49 (1.72, 11.7)
	Women	3,256	6.0% (5.2%, 6.8%)	–	5.59 (2.57, 12.2)
Multiple sclerosis	Men	4,724	23% (21%, 24%)	5.32 (3.37, 8.69)	3.63 (2.88, 4.57)
	Women	10,415	20% (20%, 21%)	4.92 (3.36, 7.36)	3.11 (2.65, 3.66)
Ovarian cancer	Women	19,780	40% (39%, 41%)	4.44 (4.13, 4.78)	1.91 (1.84, 1.98)
Pancreatic cancer	Men	13,719	56% (55%, 57%)	2.64 (2.40, 2.90)	1.28 (1.22, 1.34)
	Women	13,206	57% (56%, 57%)	2.75 (2.49, 3.04)	1.38 (1.32, 1.45)
Parkinson’s disease	Men	33,664	32% (31%, 32%)	2.91 (2.60, 3.25)	2.46 (2.27, 2.67)
	Women	22,905	35% (34%, 36%)	2.77 (2.38, 3.22)	2.77 (2.51, 3.05)
PCOS	Women	88,718	8.0% (7.9%, 8.2%)	2.78 (1.48, 5.35)	2.34 (2.16, 2.54)
Rheumatoid arthritis	Men	32,673	27% (27%, 28%)	3.63 (3.25, 4.06)	3.19 (2.99, 3.41)
	Women	70,626	30% (30%, 30%)	4.09 (3.74, 4.48)	3.03 (2.90, 3.17)
SBE	Men	5,173	83% (82%, 84%)	1.04 (0.837, 1.29)	1.16 (1.07, 1.25)
	Women	2,601	82% (80%, 83%)	1.19 (0.891, 1.59)	1.21 (1.08, 1.36)
Schizophrenia	Men	31,484	42% (42%, 43%)	1.79 (1.54, 2.09)	1.32 (1.24, 1.40)
	Women	28,633	43% (43%, 44%)	1.66 (1.44, 1.92)	1.69 (1.59, 1.79)
Tuberculosis	Men	9,976	36% (35%, 37%)	3.04 (2.45, 3.76)	3.05 (2.72, 3.42)
	Women	7,895	28% (28%, 30%)	3.84 (2.83, 5.20)	4.13 (3.55, 4.80)

^a^Adjusted for age at diagnosis, year of diagnosis, IMD score, comorbidity score 6 months (180 days) prior to diagnosis and diagnosis source.

“–” indicates that fewer than 10 patients died within 1 year of diagnosis. Odds ratios were not calculated for these conditions as the results are unlikely to be reliable. Abbreviations: CI, confidence interval; OR, odds ratio; RR, rate ratio; CHD, coronary/ischaemic heart disease; COPD, chronic obstructive pulmonary disease; IBD, inflammatory bowel disease; PCOS, polycystic ovary syndrome; SBE, subacute bacterial endocarditis.

### Sensitivity analysis 1: A&E attendance

Including A&E attendance (not necessarily leading to an emergency admission) in the emergency diagnosis definition modestly increased emergency diagnosis frequency by between 2%–3% (e.g., coeliac disease, COPD) and 8%–9% (Lyme disease, schizophrenia) (Table 5, S1 Appendix). Gender differences in the frequency of emergency diagnosis were no longer significant for schizophrenia (Aurum only), or for MS and IBD (both GOLD only). Patterns in frequency of emergency diagnosis with age and deprivation (IMD) were unchanged (S1 Appendix, S1 Data).

**Table 5:** Percentage (95% confidence interval) of patients diagnosed as an emergency stratified by gender and emergency diagnosis definition - Aurum.

Condition	*Men*		*Women*		*p*-value
	Hospital admission (1999–2019)	Hospital admission or A&E attendance (2007–2019)	Hospital admission (1999–2019)	Hospital admission or A&E attendance (2007–2019)	
Axial spondyloarthritis	18% (18%, 19%)	23% (22%, 24%)	23% (22%, 24%)	26% (24%, 27%)	< 0.01
*Brain tumours*	65% (64%, 65%)	69% (68%, 69%)	57% (56%, 57%)	61% (60%, 62%)	< 0.01
Coeliac disease	8.9% (8.3%, 9.5%)	11% (11%, 12%)	9.1% (8.7%, 9.5%)	12% (11%, 12%)	0.247
CHD	50% (50%, 50%)	56% (56%, 57%)	54% (54%, 54%)	60% (60%, 60%)	< 0.01
Colon cancer	32% (32%, 33%)	34% (33%, 34%)	36% (35%, 36%)	38% (37%, 38%)	< 0.01
COPD	36% (35%, 36%)	39% (39%, 39%)	40% (39%, 40%)	42% (42%, 43%)	< 0.01
IBD	24% (23%, 24%)	27% (26%, 27%)	26% (26%, 26%)	29% (29%, 30%)	< 0.01
Lung cancer	44% (44%, 45%)	47% (47%, 48%)	44% (44%, 45%)	47% (46%, 47%)	0.218
*Lyme disease*	8.3% (7.2%, 9.4%)	17% (15%, 18%)	6.0% (5.2%, 6.8%)	14% (13%, 15%)	< 0.01
*Multiple sclerosis*	23% (21%, 24%)	27% (25%, 28%)	20% (20%, 21%)	25% (24%, 26%)	0.0148
Ovarian cancer			40% (39%, 41%)	42% (41%, 43%)	
Pancreatic cancer	56% (55%, 57%)	59% (58%, 60%)	57% (56%, 57%)	60% (59%, 61%)	0.142
Parkinson’s disease	32% (31%, 32%)	34% (33%, 34%)	35% (34%, 36%)	38% (37%, 38%)	< 0.01
PCOS			8.0% (7.9%, 8.2%)	12% (12%, 12%)	
Rheumatoid arthritis	27% (27%, 28%)	31% (30%, 32%)	30% (30%, 30%)	33% (33%, 34%)	< 0.01
SBE	83% (82%, 84%)	88% (87%, 89%)	82% (80%, 83%)	87% (85%, 88%)	0.155
Schizophrenia	42% (42%, 43%)	51% (50%, 51%)	43% (43%, 44%)	51% (51%, 52%)	0.163
*Tuberculosis*	36% (35%, 37%)	41% (40%, 42%)	28% (28%, 30%)	33% (32%, 34%)	< 0.01

Note that *p*-values relate to the null hypothesis that there was no difference in the percentage of cases diagnosed within 30 days of an emergency hospital admission or A&E attendance between men and women tested using Chi-squared tests with Yates’ continuity correction. Italicised conditions indicate that men had statistically significantly more emergency diagnoses; underlined conditions indicate that women had statistically significantly more emergency diagnoses. Abbreviations: A&E, accident and emergency; CHD, coronary/ischaemic heart disease; COPD, chronic obstructive pulmonary disease; IBD, inflammatory bowel disease; PCOS, polycystic ovary syndrome; SBE, subacute bacterial endocarditis.

Time trends in the percentage of patients diagnosed as an emergency generally remained similar, except for some conditions, such as schizophrenia which exhibited a sharper increase in frequency from 2010 (S1 Appendix).

Whilst 1-year mortality marginally decreased due to the later study period (2007–2019, versus 1999–2019 for main analysis), the poor prognostic outcomes associated with an emergency diagnosis persisted (S1 Appendix). Patterns of association of emergency diagnosis status with mortality varying by gender remained similar in Aurum, with an additional statistically significant difference observed in TB, but in GOLD statistically significant differences were observed in coeliac disease and IBD (in comparison with CHD alone in the main analysis).

### Sensitivity analysis 2: Adjusted hospitalisation risk

**Fig 11:**
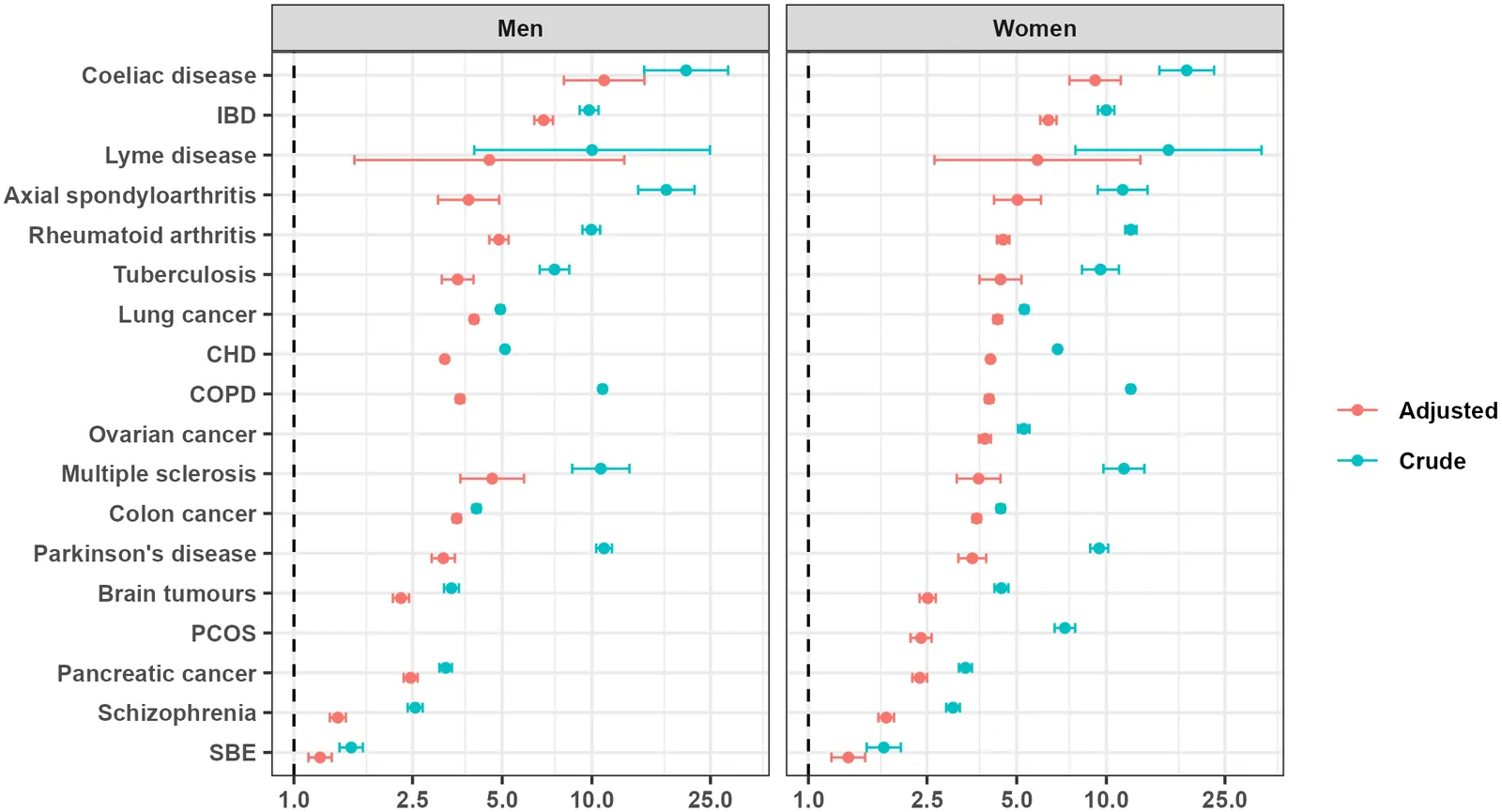
Forest plot of rate ratios for time in hospital whilst alive in the year post-diagnosis following an emergency diagnosis – Aurum. Rate ratios were adjusted for age at diagnosis, year of diagnosis, IMD score, comorbidity score 6 months (180 days) prior to diagnosis and diagnosis source and an offset for number of days alive in the year after diagnosis. Abbreviations: CHD, coronary/ischaemic heart disease; COPD, chronic obstructive pulmonary disease; IBD, inflammatory bowel disease; PCOS, polycystic ovary syndrome; SBE, subacute bacterial endocarditis.

After accounting for time being alive in the year post-diagnosis, both crude and adjusted rate ratios for total duration of overnight hospital stays were higher than those observed in the main analysis (except for SBE in men in GOLD, where hospitalisation burden decreased marginally) (Fig 11, S1 Appendix). Increases in RRs were generally proportional to the magnitude of adjusted ORs for 1-year mortality, described above, with the biggest difference observed for coeliac disease in women (main analysis estimate, 6.90, 95% CI [5.70, 8.34]; revised estimate 9.17, 95% CI [7.52, 11.2]).

### Sensitivity analysis 3: COVID-19 impact

After excluding patients diagnosed in 2019, we did not observe any notable deviance from our main findings (S1 Appendix).

## Discussion

More than 1 in 5 patients were diagnosed as an emergency in 9 out of the 13 examined non-neoplastic conditions, including over 30% of patients diagnosed with Parkinson’s disease and 35% of patients with COPD. Compared with patients who were not diagnosed as an emergency, those diagnosed as emergencies were substantially more likely to die and to spend longer in hospital in the year post-diagnosis for nearly all conditions. Such excess risks among emergency presenters were particularly observed in coeliac disease and inflammatory bowel disease. Gender differences were observed in both the frequency and prognostic outcomes of emergency diagnoses.

Major strengths of the study are the extension of the emergency diagnosis concept to non-neoplastic conditions, and the use of high-quality population-based data. The broad range of cardiac, respiratory, infectious, autoimmune, and neurological conditions studied enabled the assessment of emergency diagnosis and its correlates across a wide spectrum of diseases. Our analyses were repeated in two electronic health record datasets that produced comparable results [38]. We included five cancer sites for comparison and observed findings consistent with existing literature.

A limitation is that the study relies on accurate coding of diagnoses and diagnosis dates. Some GPs may code suspicion rather than confirmation of a diagnosis, resulting in disease-free patients being erroneously included. Some variation in clinical coding patterns is to be expected across primary care physicians and clinical settings. A patient may be diagnosed erroneously, and their diagnosis revised later; conversely, in some patients, treatment may have been started before a precise diagnosis was confirmed—schizophrenia, for example [39]. Our approach nonetheless has face validity as to the diagnoses made and the date of their recording, and we have minimised potential inaccuracies by excluding records indicating prevalent disease and verifying that observed sample characteristics conform to expected gender and age case-mix with clinical co-authors [40,41].

Consistent with international practice, we have defined emergency diagnosis as emergency hospital admission on the day of diagnosis or during the 30 preceding days (a ‘contextual’ rather than clinical definition of emergency diagnosis) [3,5,42]. Our definition, appropriately, encompasses circumstances where it is not possible to reach a diagnosis during the acute emergency care episode. Although scalable and reproducible, this definition may capture instances of genuine diagnoses occurring incidentally during emergency admissions triggered by unrelated pathology. Such incidental diagnoses may occur more frequently in older and more comorbid patients, which may explain some of the excess mortality observed in emergency-diagnosed patients. Further research is needed to understand the strengths and limitations of the operational definition used, and potential refinements.

Evidence on emergency diagnoses of non-neoplastic conditions is limited. We are aware of a single small (169 patients) study examining diagnostic pathways among tuberculosis patients in Sweden, noting that ~27% of patients with active TB were referred from an emergency department [43], compared with around 33% of women and 43% of men in our study.

The frequency of emergency presentations in cancer diagnosis is routinely reported by the National Disease Registration Service of NHS England [44], using a more complex definition—namely, the ‘Routes to Diagnosis’ hierarchical algorithm, which assigns patients to one of eight diagnostic routes [6,38,45]. We have used a simpler (single-criterion) definition which aligns with an increasing number of international studies [3,42,46–48]. Our findings of poor mortality outcomes among emergency-diagnosed cancer patients are consistent with previous studies [5,48]. Pethick and colleagues explored the number of days a patient was in hospital for inpatient or outpatient care in the year post-diagnosis of cancer; we have extended this through our examination of time admitted to hospital in the year after diagnosis stratified by emergency diagnosis status [49]. Most evidence on emergency diagnosis of cancer in England relies on the ascertainment of case status and diagnosis date from cancer registration data, which is known to have some differences compared with electronic health record data [50–52]. Evidence suggests that across the included sites, for cases that are captured in both EHR and cancer registration data, between 60%–80% of cases would have the diagnosis recorded on the same date or earlier in EHR data [50]. This may explain why, on average, our frequency estimates for cancers are a few percentage points higher than the existing literature, whilst our odds ratios are slightly lower.

Patterns that have been observed previously in the cancer literature—a U-/J-shaped pattern of emergency diagnosis frequency with age, and a small increase in frequency with deprivation—were also noted in many other conditions in this study [6,53]. We also observed an increase in the frequency of emergency diagnosis between 1999 and 2009 for some (CHD, SBE, and rheumatoid arthritis, for example), but not all, conditions. These findings highlight the need for further research into both general (“disease-agnostic”) drivers of emergency diagnosis, and disease-specific drivers—such as changes to diagnostic processes or public awareness—that may result in patterns that are only observed in specific conditions.

Although varied and complex reasons are likely implicated in emergency diagnosis, breakdowns in the diagnostic process are a credible factor. For conditions like COPD, rheumatoid arthritis, coeliac disease, and PCOS—which can typically be diagnosed in a primary care setting—capacity issues in primary care may result in emergency diagnoses. For example, patients unable to see their GP or have prompt investigation in the community, may resort to presenting to the emergency department. Similarly, for conditions that typically require specialist assessment or investigation for a firm diagnosis to be made—such as multiple sclerosis, Parkinson’s disease, or inflammatory bowel disease—long waiting times for secondary care may mean that the condition of patients waiting to be seen or investigated in hospital may deteriorate markedly, triggering an emergency admission. While access issues across the healthcare system need resolving, they are unlikely to explain the extent of emergency diagnoses that we observed in non-neoplastic conditions.

Emergency diagnoses resulting from severe manifestations of disease without, or with minimal, prior prodromal symptoms are likely to represent the best standard of care for affected patients. However, those that occur following active and sustained healthcare-seeking by patients could be delayed diagnoses. Further research is needed to explore drivers of emergency diagnosis in non-neoplastic conditions, particularly the role of the healthcare system.

The extent to which patient and social factors contribute to emergency diagnoses also merits consideration. In cancer, public health education campaigns have been used to encourage symptomatic patients to present to primary care earlier [54,55]. Such approaches could, in theory, help reduce emergency diagnoses of other selected conditions, although the extent to which emergency diagnoses are driven by such factors is not yet clear and further research is needed to determine whether and how such campaigns could be effective [56]. Variation in emergency diagnosis by gender is particularly important and warrants further exploration; it has long been established that in some conditions the quality of the diagnostic process is worse in women, and our findings align with such a potential ‘Gender Health Gap’ in some (though not all) conditions [57–59].

Patients who are diagnosed as an emergency also exhibit notably worse clinical outcomes. Our findings suggest that in conditions for which baseline mortality is either negligible or quite high (e.g., PCOS, SBE) emergency diagnosis status has limited association with mortality. However, in conditions that are generally perceived as having ‘good’ prognosis—coeliac disease, inflammatory bowel disease, and MS, for example—mortality is amplified in patients diagnosed as an emergency, proportional to the disease-specific baseline (Fig 10).

It is unclear what is driving this excess mortality, or whether ‘re-directing’ patients to other diagnostic routes would lead to improved patient outcomes. Intuitively, patients who are hospitalised are more likely to die [60,61]. However, it is possible that patients who are diagnosed as emergencies differ from those diagnosed electively in both their overall characteristics and the severity of their illness. Amongst undiagnosed patients, one reason for patients to have more severe illness could be delayed diagnosis—namely that their disease has developed with limited treatment which may have led to complications, and subsequently the need for an emergency diagnosis. Furthermore, there may be interactions between patient’s morbidity and delayed diagnosis. These considerations should caveat the interpretation of our findings and motivate future research.

Further research is needed to understand the extent to which emergency diagnoses can be prevented, and whether doing so would improve outcomes for these patients. In particular, evaluating healthcare-seeking behaviour prior to diagnosis and missed diagnostic opportunities could help to elucidate the extent to which emergency diagnoses constitute delayed diagnoses, and may therefore be preventable. In addition, establishing whether, and for what reason, patients diagnosed as an emergency are ‘sicker’ at the point of diagnosis—for instance, by examining presenting signs and symptoms, or treatments initiated at diagnosis—than patients who are not diagnosed as an emergency, and analysing information on causes of death could allow for consideration of whether preventing emergency diagnosis would improve patient outcomes.

Emergency diagnoses affect many patients across a wide range of conditions, and patients diagnosed as an emergency are consistently more likely to die and to spend longer in hospital in the year after diagnosis. Further research is needed to understand whether it is possible to diagnose these patients through alternative diagnostic routes, whether doing so would positively impact patient outcomes, and, if so, how these aims could be achieved in practice.

## Supporting information

S1 Checklist RECORD statement. (PDF)

S1 Appendix Additional study methods and findings. (PDF)

S1 File Data extract protocol (22_002264). (PDF)

S1 Data Values underlying figures. (XLSX)

## Abbreviations

APC: Admitted Patient Care
AS: axial spondyloarthritis
CHD: coronary/ischaemic heart disease
COPD: chronic obstructive pulmonary disease
CPRD: Clinical Practice Research Datalink
HES: Hospital Episode Statistics
IBD: inflammatory bowel disease
IMD: Index of Multiple Deprivation
MS: multiple sclerosis
NHS: National Health Service
ONS: Office for National Statistics
PPI: patient and public involvement
SBE: subacute bacterial endocarditis.
