# Supplementary material for: Frequency and prognostic outcomes of emergency diagnosis in 13 non-neoplastic conditions in England: A population-based cohort study using linked electronic health records of 1.7 million patients
Source: PLoS Med. 2026 Aug 25;23(8):e1005182. doi: 10.1371/journal.pmed.1005182 (PMC13505939; doi:10.1371/journal.pmed.1005182)
Supplement: Supplementary file 2 [file pmed.1005182.s002.pdf]

Frequency and prognostic outcomes of emergency diagnosis in 13 non-neoplastic conditions in England: a population-based cohort study using linked electronic health records of 1.7 million patients – S1 Appendix

Additional study methods and findings

Whitfield E et al.

Contents

Lay summary 2

Study population 3

Patient characteristics 24

Main results – GOLD 58

Main results – dataset comparison 67

Sensitivity analysis 1: A&E attendance 71

Sensitivity analysis 2: Adjusted hospitalisation risk 85

Sensitivity Analysis 3: COVID-19 impact 89

Lay summary

Co-produced with PPI representatives Julie Halliwell and Helena Ward

There is a growing understanding that improvements are needed in diagnosing medical conditions. When patients get the right diagnosis earlier, it can lead to better health and a smoother experience. Despite a strong focus on achieving earlier diagnoses of cancer, we don't know as much about diagnosing other conditions earlier.

Using health records from NHS patients in England, we examined the possibility of earlier diagnosis of 18 conditions between 1999 and 2019. 13 non-cancer conditions (like tuberculosis, Parkinson's, and coeliac disease) were investigated, along with 5 cancers.

We investigated the paths patients took through the healthcare system to get diagnosed. We looked at how often patients with each condition took one specific path: emergency diagnosis (when a patient is diagnosed with a condition after receiving emergency hospital care).

Emergency diagnoses were surprisingly common: at least 20% of patients were diagnosed as an emergency in 9 of the 13 non-cancer conditions.

To understand what happened after an emergency diagnosis, we examined outcomes in the year after diagnosis. We looked at the number of patients who died, and the amount of time patients spent in hospital, accounting for factors like age and other illnesses.

Previous research found that patients diagnosed with cancer as an emergency were more likely to have worse outcomes than those diagnosed in other ways. We found the same pattern in many other conditions as well. Patients diagnosed as an emergency were more likely to die within a year than those diagnosed in other ways. This happened even in conditions where both emergency diagnosis and death in the year after diagnosis were uncommon, like coeliac disease.

In some scenarios, it is unlikely that emergency diagnoses could be avoided, and they may represent the most appropriate way for a patient to be diagnosed. However, emergency diagnoses appear to occur quite frequently and be linked to poorer outcomes across many conditions. This suggests efforts to reduce the number of patients diagnosed as an emergency may be required. More research is needed to find out who is most at risk of emergency diagnosis; why these patients have worse outcomes; and whether some emergency diagnoses could be prevented.

Our findings suggest that there may be potential to improve the diagnosis of many conditions and that more research is needed in this area.

Study population

For each condition of interest, and in each of GOLD and Aurum, the following steps were taken to select cases for inclusion:

1. All records of the condition identified in CPRD by CALIBER, and in HES APC, HES OP, and ONS by CPRD. These records were then shared with the study team to identify the study population.
2. Clean all records of the condition from CPRD, HES APC, HES OP, and ONS by excluding
    a. Records with missing event dates
    b. Records dated prior to the patient's year of birth
    c. Patients with missing year of birth
    d. Patients who were not deemed acceptable (per CPRD flag)
    e. Patients who were not linkage eligible
3. For each patient, the earliest record of the condition in each dataset was identified, and all other records were dropped.
4. For each patient, the earliest record of the condition across all four datasets was identified. The date of this record was deemed to be the 'diagnosis date' and all other records were dropped. When multiple records of the condition were captured on the same date, any record indicating that the condition was prevalent was prioritised (e.g., a record using the code 'History of [condition]'). Then if there were still records from multiple datasets, datasets were prioritised in the following order: (a) ONS, (b) HES, (c) CPRD[1]
5. Any patient with a prevalent record indicating pre-existing disease on their diagnosis date was excluded – these were unlikely to be true 'incident diagnoses'
6. Any patient who did not meet condition-specific criteria defining the population at risk was excluded (Table 1)
7. Any patient diagnosed outside of the study period (1st January 1999 – 31st December 2019) was excluded

Any patient not registered in primary care on the diagnosis date was excluded, with specific criteria applied determined by the data source in which the earliest incident record of the condition was identified (

8. Table 2)

Table 1. Criteria describing the population at risk for each included condition. Juvenile or paediatric forms of the included conditions are excluded a priori.

**Condition**	**Age at diagnosis date**	**Gender**
Axial spondyloarthritis	Over 16	All
Brain tumours	Over 13	All
Coeliac disease	Over 13	All
Coronary/Ischaemic heart disease	Over 30	All
Colon cancer	Over 30	All
Chronic obstructive pulmonary disease	Over 30	All
Inflammatory bowel disease	Over 13	All
Lung cancer	Over 30	All
Lyme disease	Over 16	All
Multiple sclerosis	Over 16	All
Ovarian cancer	Over 30	Women
Pancreatic cancer	Over 30	All
Parkinson's disease	Over 30	All
Polycystic ovary syndrome	Over 13	Women
Rheumatoid arthritis	Over 16	All

[1] This hierarchy was determined such that a patient with a valid incident diagnosis was most likely to be included in the study population based on the criteria applied in step x.

Subacute bacterial endocarditis	Over 16	All
Schizophrenia, and other chronic psychoses	Over 16	All
Tuberculosis	Over 16	All

Table 2. Registration criteria applied to select study population

Data source	Registration criteria
CPRD	• Be registered and up-to-standard for at least one year at diagnosis date<br>• Diagnosis date is before the patient's transfer-out date, death date, and practice last collection date
HES	• Be registered and up-to-standard at diagnosis date<br>• Diagnosis date is before the patient's transfer-out date, death date, and practice last collection date
ONS	• Be registered and up-to-standard at diagnosis date<br>• Diagnosis date is at most 30 days after the patient's transfer-out date, death date, and practice last collection date (to allow for small variation in practice recording of patient death)

Following the case selection process, complete records were received for all included patients. A number of additional cleaning steps were then taken[2]. This included

- Restricting the study population to those with a recorded gender of 'Man' or 'Woman'
- Excluding all patients registered at practices in Aurum flagged by CPRD as being 'migrators' (i.e., a practice that may also be present in CPRD GOLD) or as 'mergers' (practices that have merged into other Aurum practices)
- The creation of practice 'up-to-standard' dates in Aurum. At the time our dataset was extracted no up-to-standard date was provided for CPRD Aurum practices. In order to address practice-level irregularities noted during the data exploration phase we produced an up-to-standard date for each Aurum practice based on four criteria[3]:
    - Size: the earliest date after which the number of registered patients surpassed 20% of the total number of patients included from that practice for at least 12 consecutive months
    - Stability: the earliest date after which the number of registered patients did not differ by more than 30% from the number of registered patients a year earlier, for at least 12 consecutive months
    - Observations: the earliest date after which at least 20% of registered patients had a recorded observation each month for 12 consecutive months (N.B. It was not required that the same 20% of registered patients had an observation in each of the 12 months, only that some 20% had an observation). The CPRD Aurum Observation table contains records of symptoms, diagnoses, and test results recorded in the GP system.
    - Consultations: the earliest date after which at least 20% of registered patients had a recorded consultation, and the practice recorded at least five different types of consultation records (as determined by conssourceid) each month for 12 consecutive months

  For each practice, data from January 1985 (the earliest UTS date from CPRD GOLD) were used to identify the earliest date that each of these criteria were met. The latest of these four dates (i.e., the date that all criteria were first met) was then assigned as the up-to-standard date for the practice. If a suitable

[2] The study data extract also included up to five controls per case (per condition) matched on age, sex, and GP practice, for use in separate, but related studies. Additional cleaning steps were also applied to controls as relevant, with controls additionally being excluded when their matched case had been excluded. Specifically, controls were counted towards the practice sizes used to develop UTS dates and to exclude small practices

[3] Note that these criteria were developed in line with the intended use of the study data extract, and are unlikely to necessarily be the most applicable or appropriate criteria to develop up-to-standard dates for all data extracts.

date was not identified for one of these criteria, the practice was excluded from all analyses. We assumed that the pre-existing UTS date in CPRD GOLD was sufficient to ensure data quality. Registration criteria were then re-applied to remove any cases who were no longer deemed suitable for inclusion.

- When cases were initially identified, as described above, ONS death records were only available when they contained a record of one of the conditions of interest. Once complete records were available, all ONS death records for included patients were available, and for some patients ONS death dates preceded the death date recorded in CPRD. Re-application of registration criteria also removed any cases who were no longer deemed suitable for inclusion once their ONS death date was used.
- A code for latent tuberculosis was erroneously included on the SNOMED codelist (see https://doi.org/10.5281/zenodo.13710755) for Tuberculosis at the point of case identification. This was corrected in the additional cleaning phase by removing any case with the flagged code recorded on their diagnosis date.
- Any CPRD patient with a HES ID (gen_hesid) linked to 100 or more other CPRD patient IDs (patid), as determined from the 'n_patid_hes' variable in the HES APC, OP, and A&E Patient tables was excluded.
- If any CPRD practices with fewer than 100 patients included in the study extract remained, all patients at these practices were excluded because unusual recording patterns or quality issues would be harder to detect due to the small sample size.

Axial spondyloarthritis

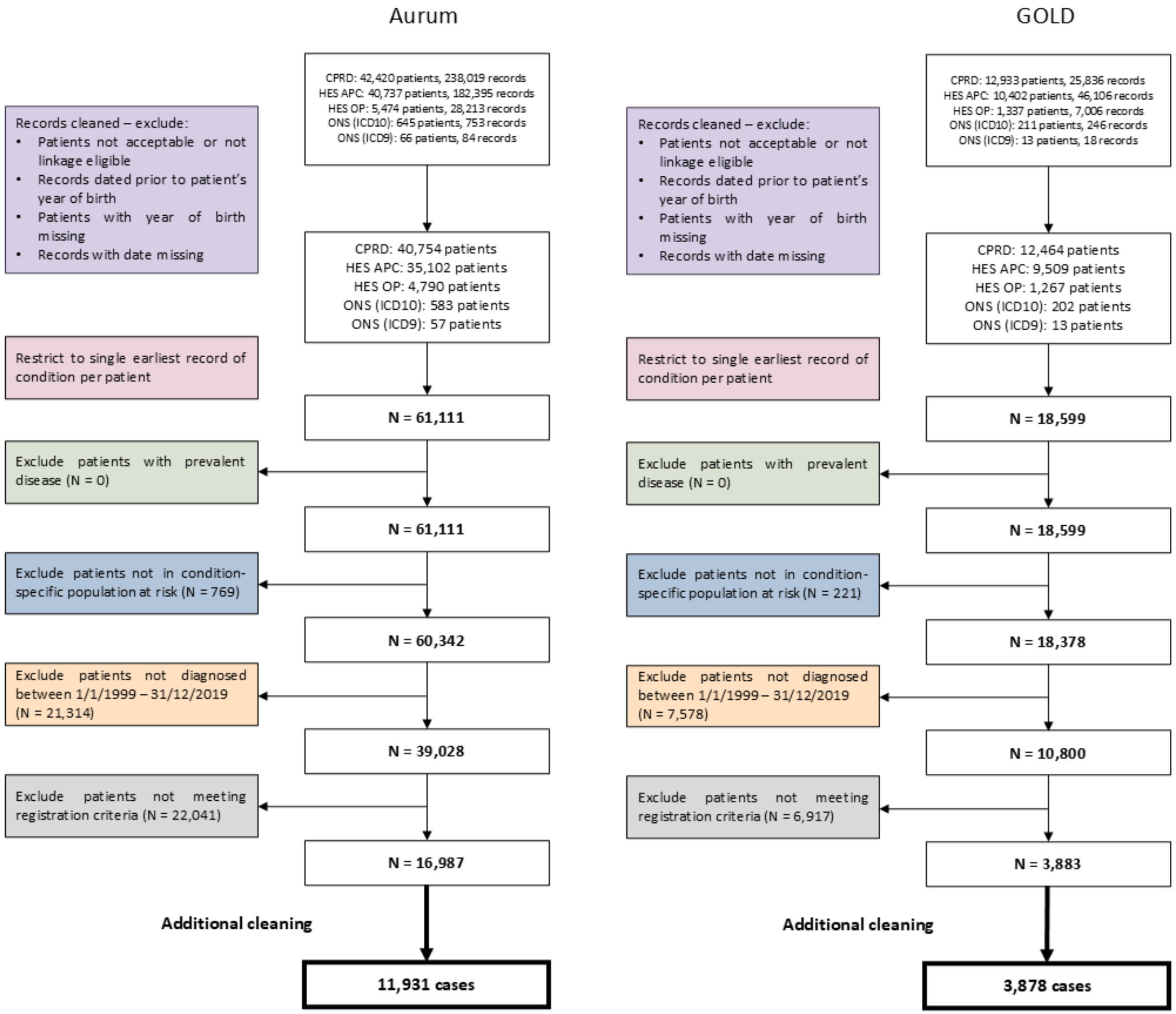

Figure 1. Flowcharts of patient selection for axial spondyloarthritis

Brain tumours

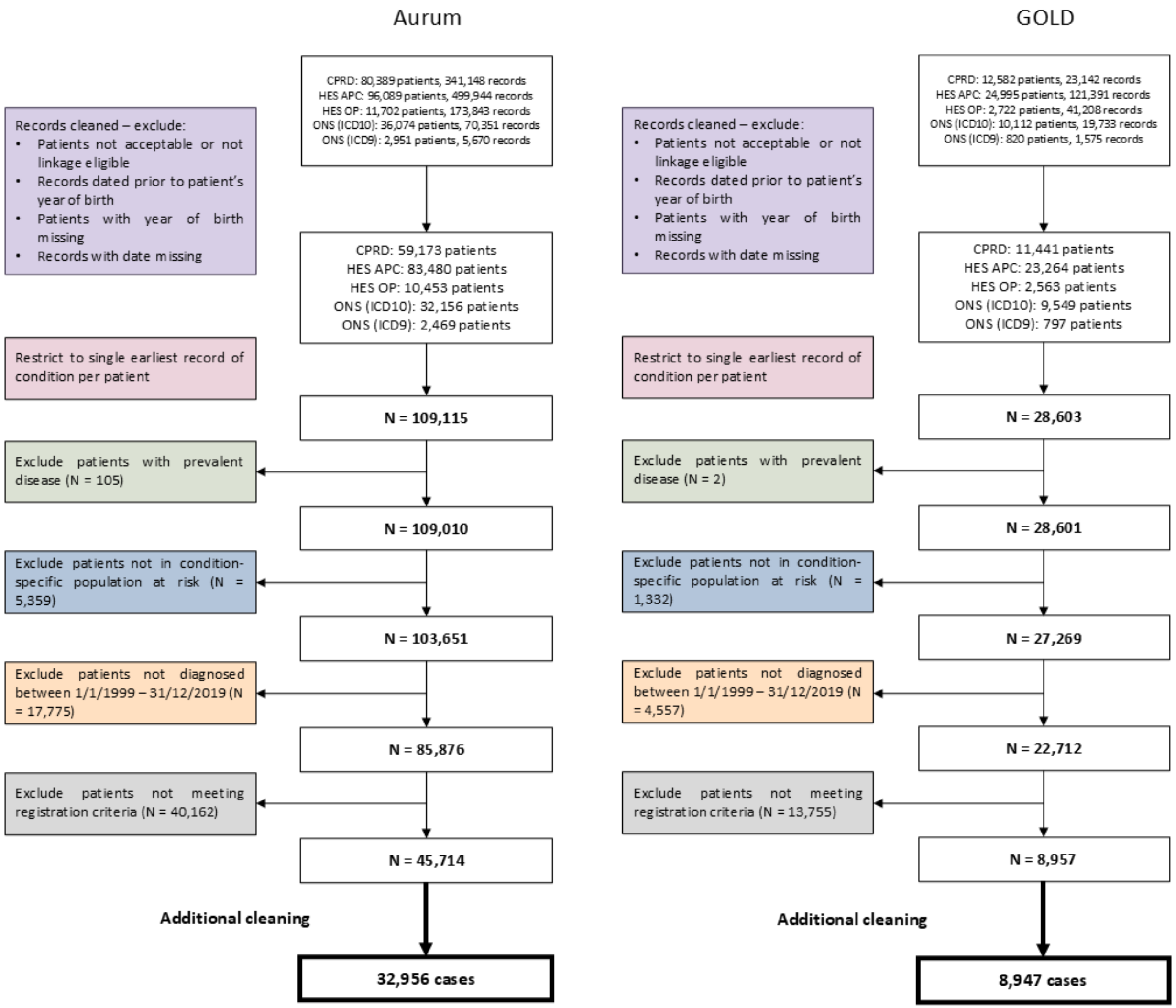

Figure 2. Flowcharts of patient selection for brain tumours

Coeliac disease

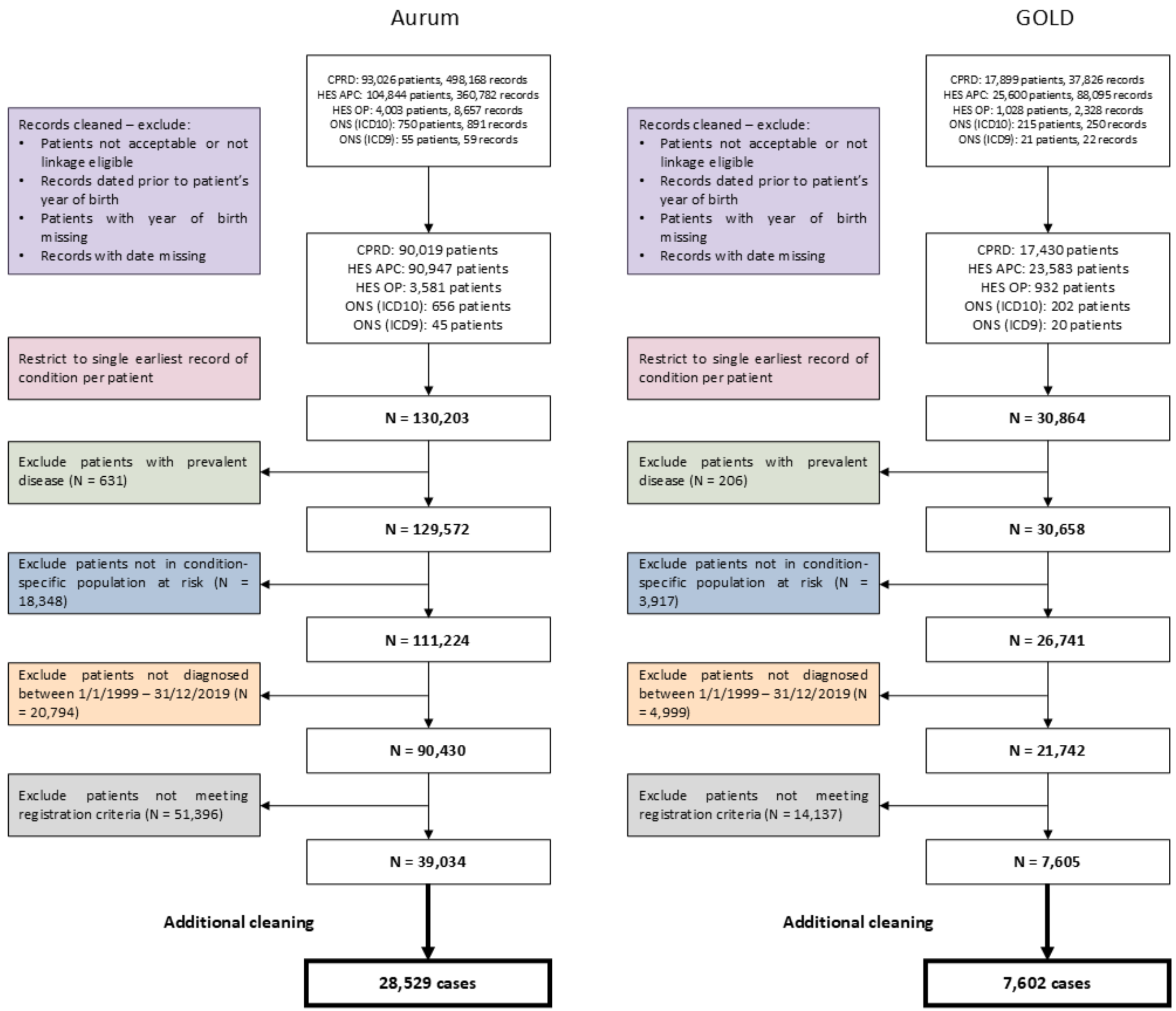

Figure 3. Flowcharts of patient selection for coeliac disease

Coronary/Ischaemic heart disease

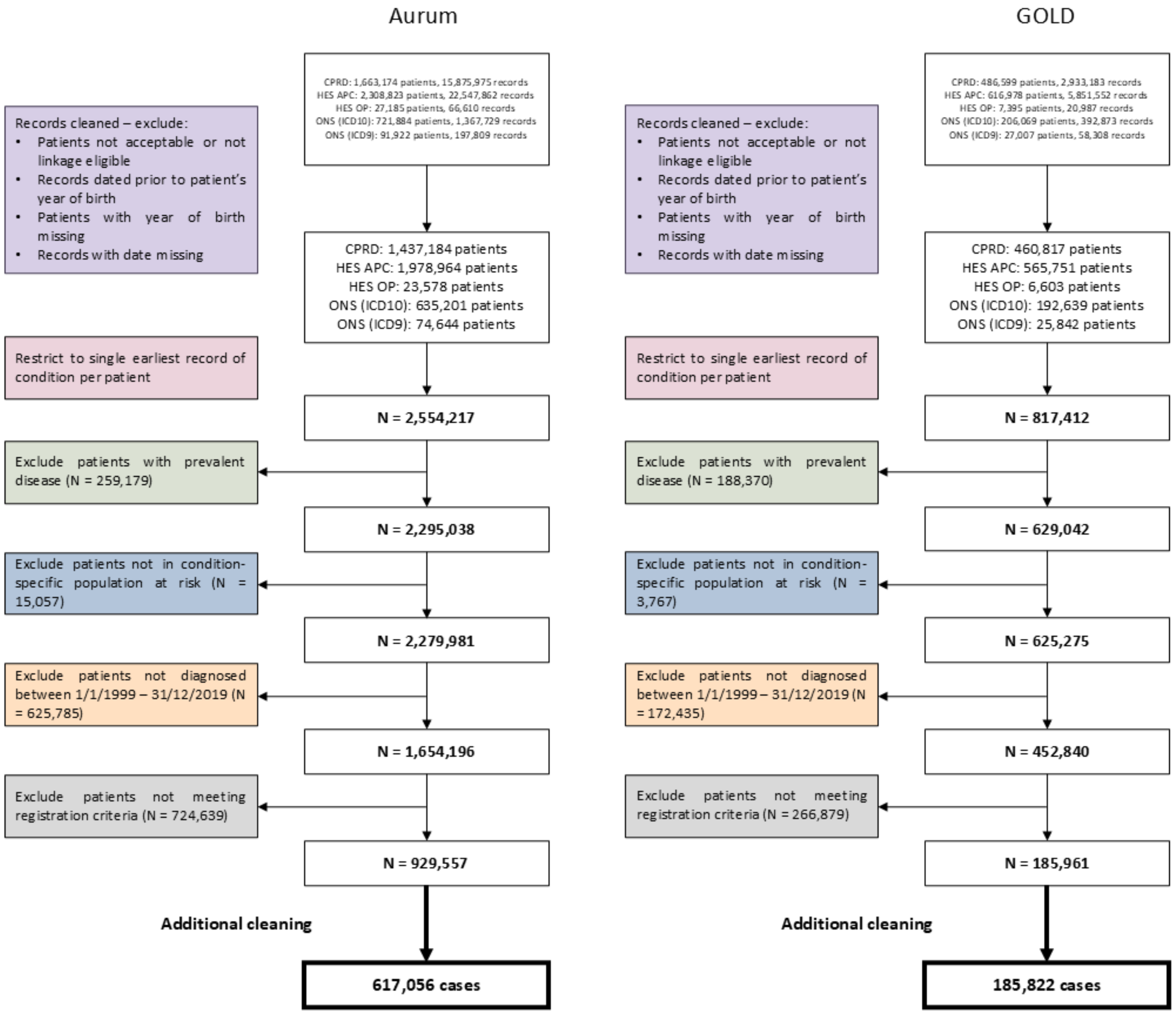

Figure 4. Flowcharts of patient selection for coronary/ischaemic heart disease (CHD)

Colon cancer

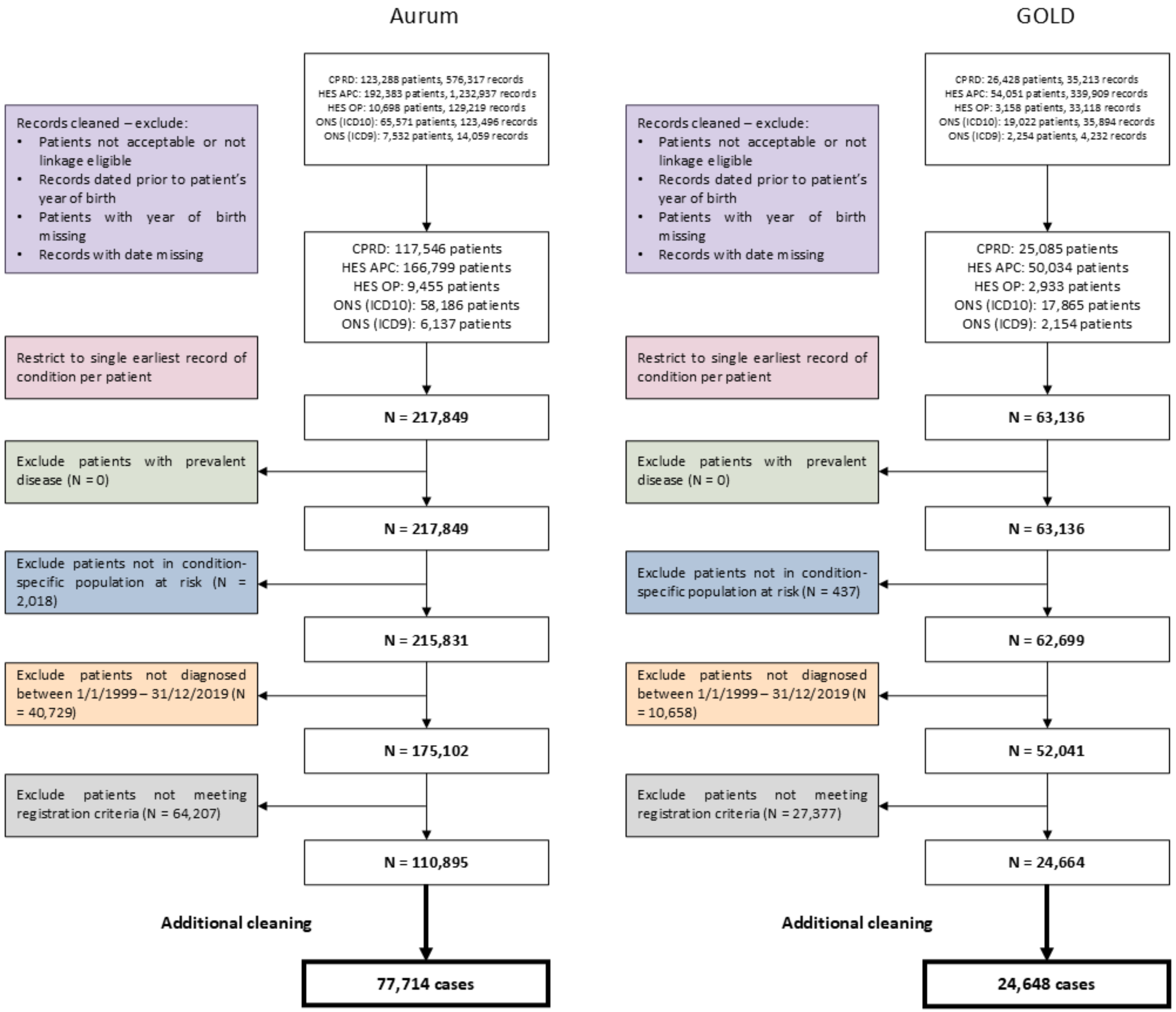

Figure 5. Flowcharts of patient selection for colon cancer

Chronic obstructive pulmonary disorder

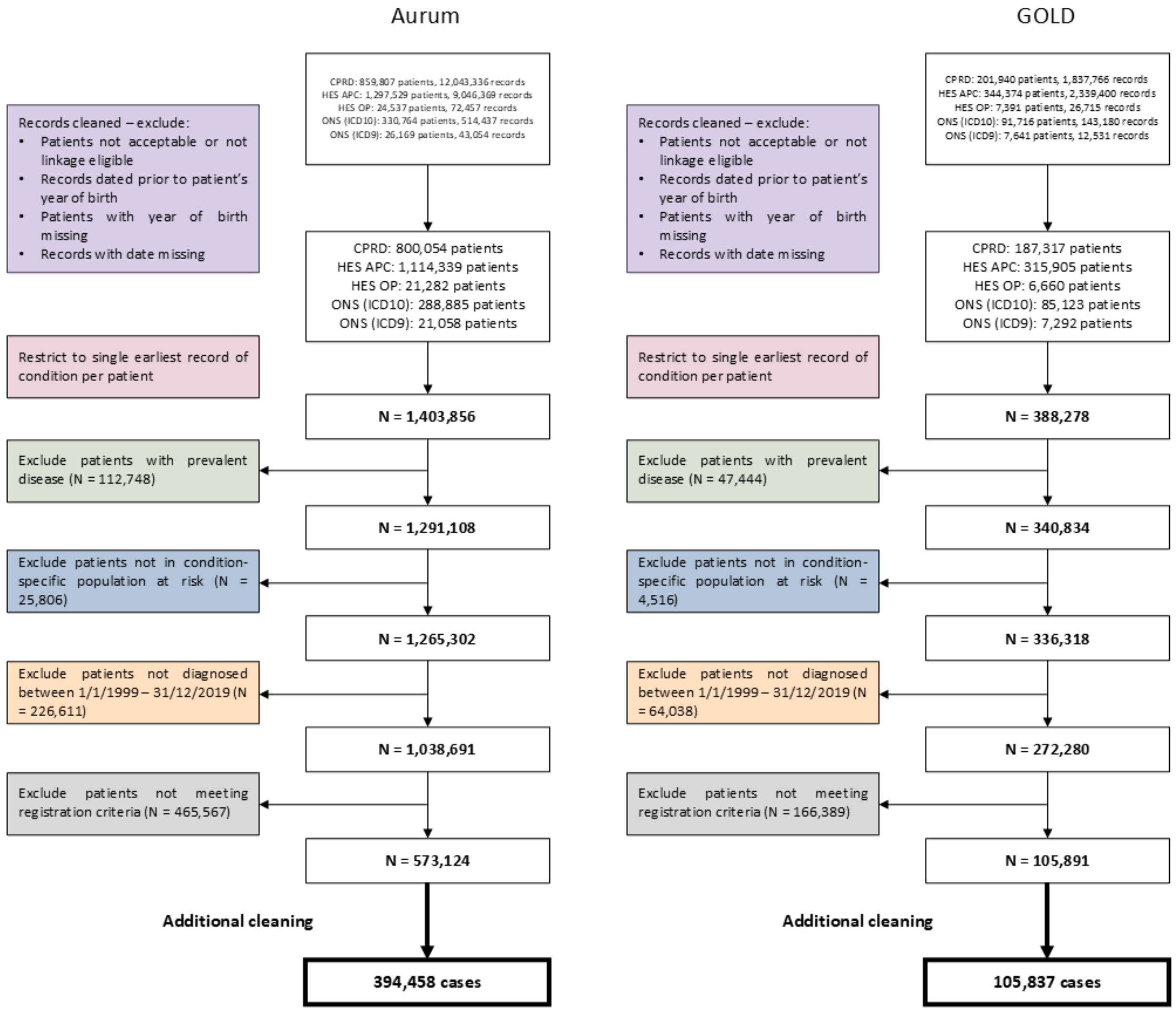

Figure 6. Flowcharts of patient selection for chronic obstructive pulmonary disorder (COPD)

Inflammatory bowel disease

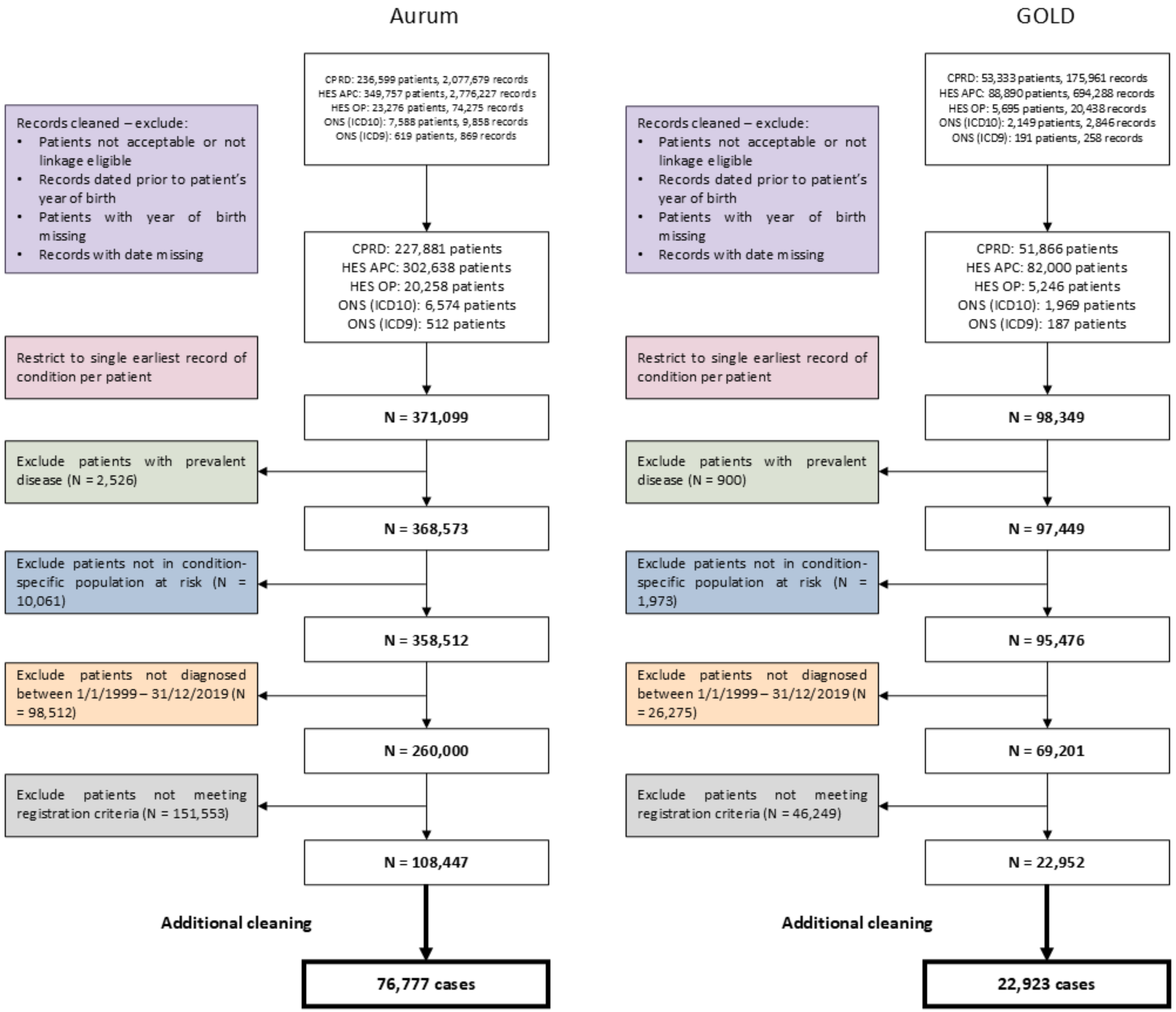

Figure 7. Flowcharts of patient selection for inflammatory bowel disease (IBD)

Lung cancer

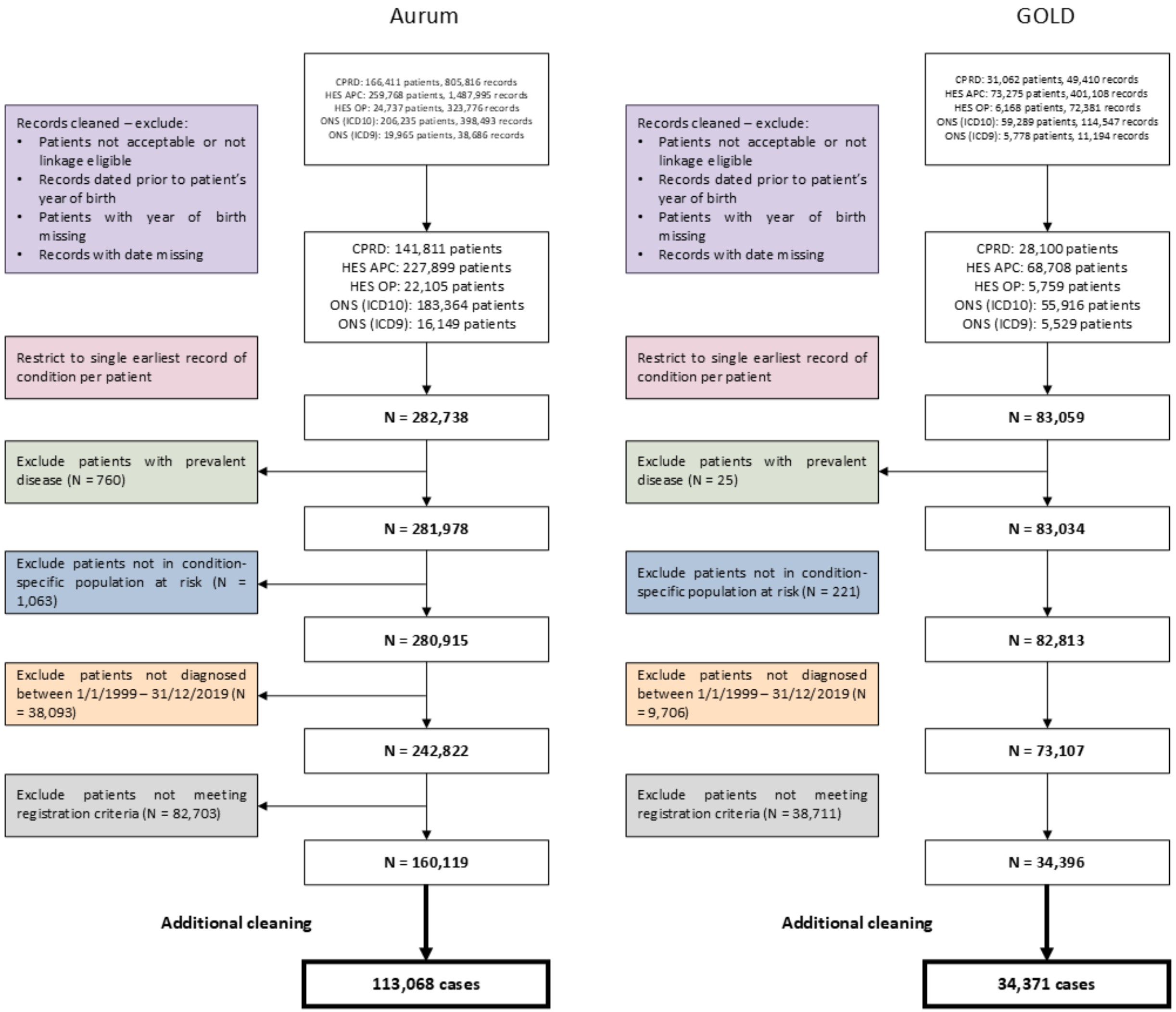

Figure 8. Flowcharts of patient selection for lung cancer

Lyme disease

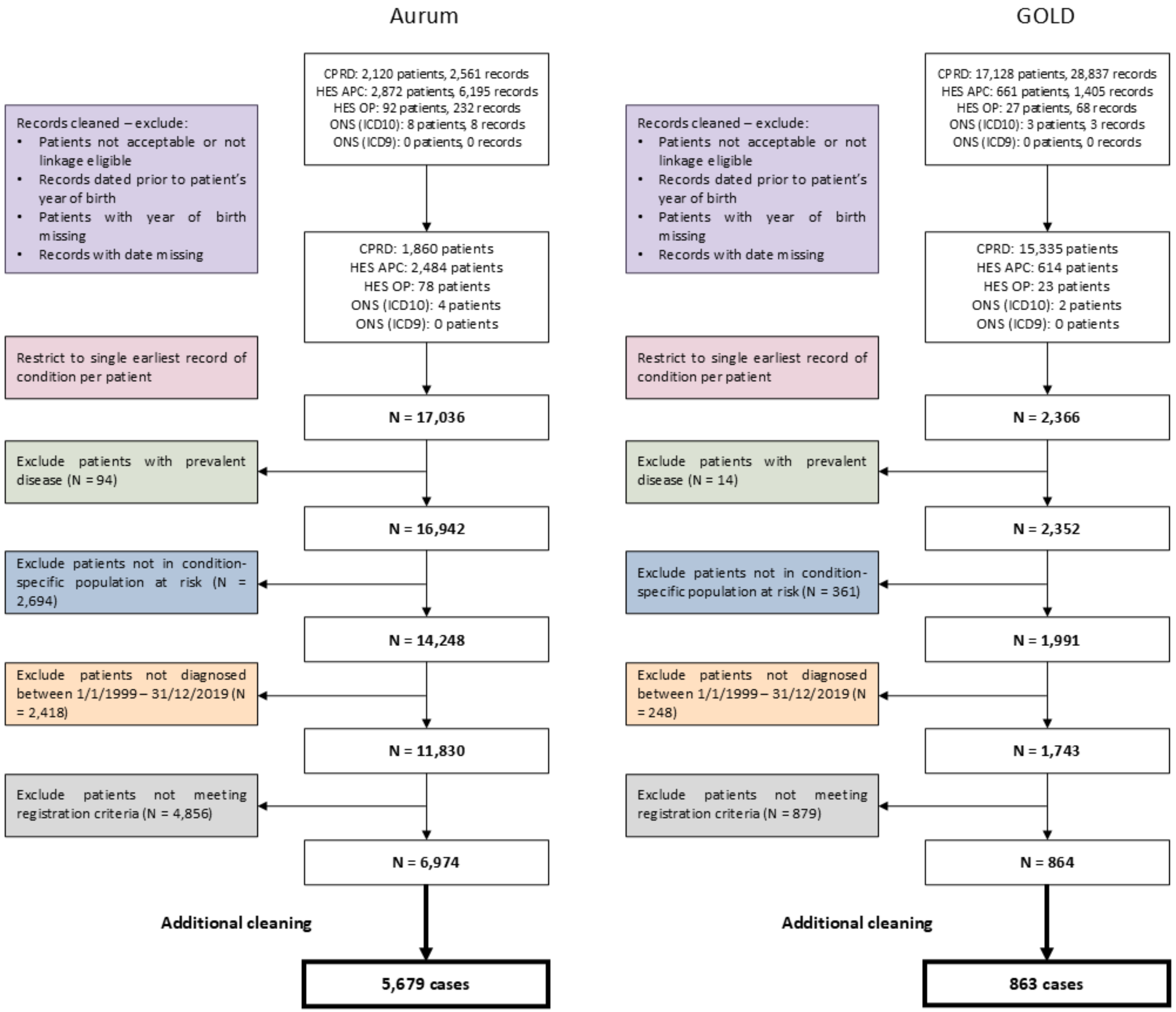

Figure 9. Flowcharts of patient selection for Lyme disease

Multiple sclerosis

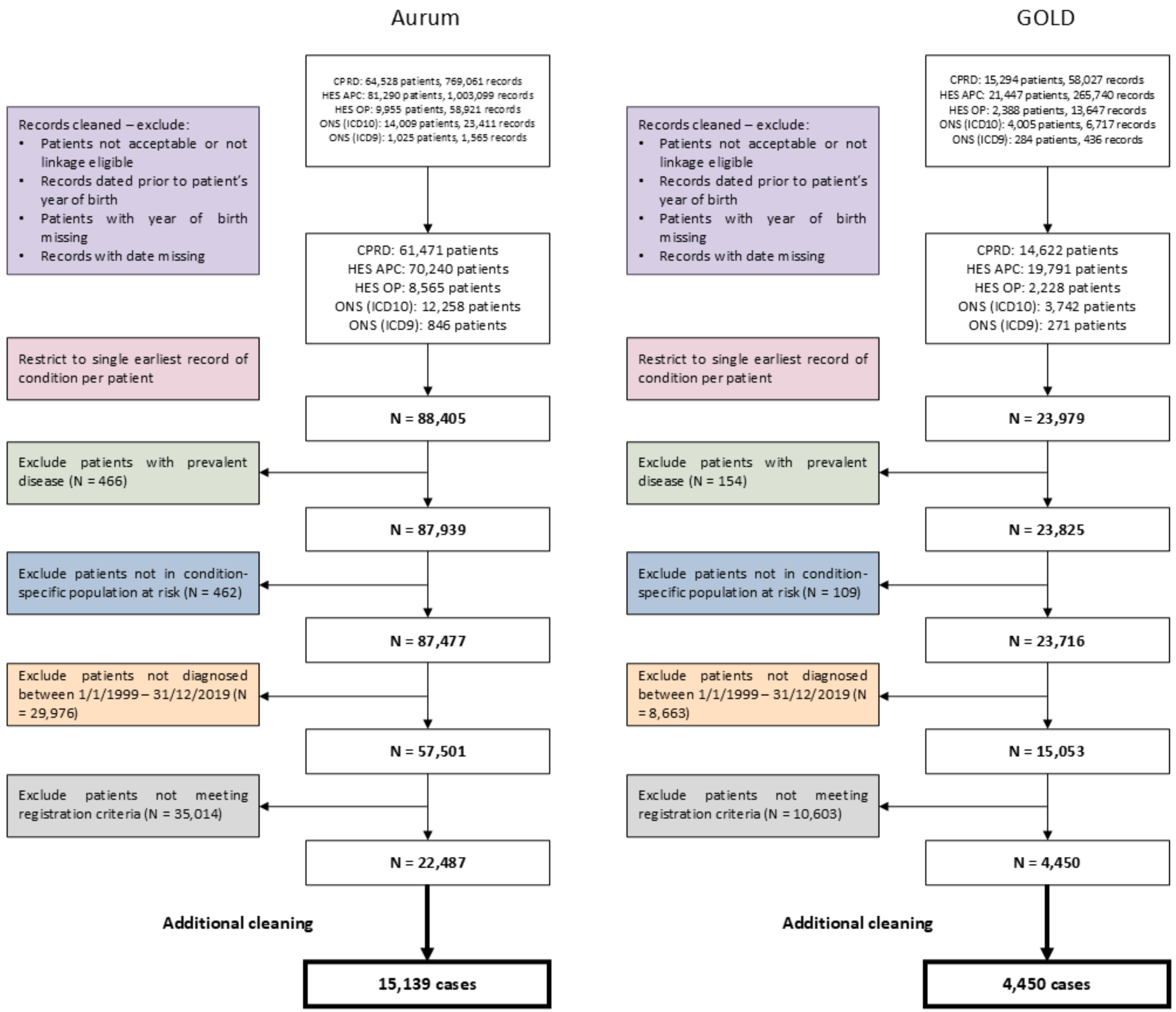

Figure 10. Flowcharts of patient selection for multiple sclerosis (MS)

Ovarian cancer

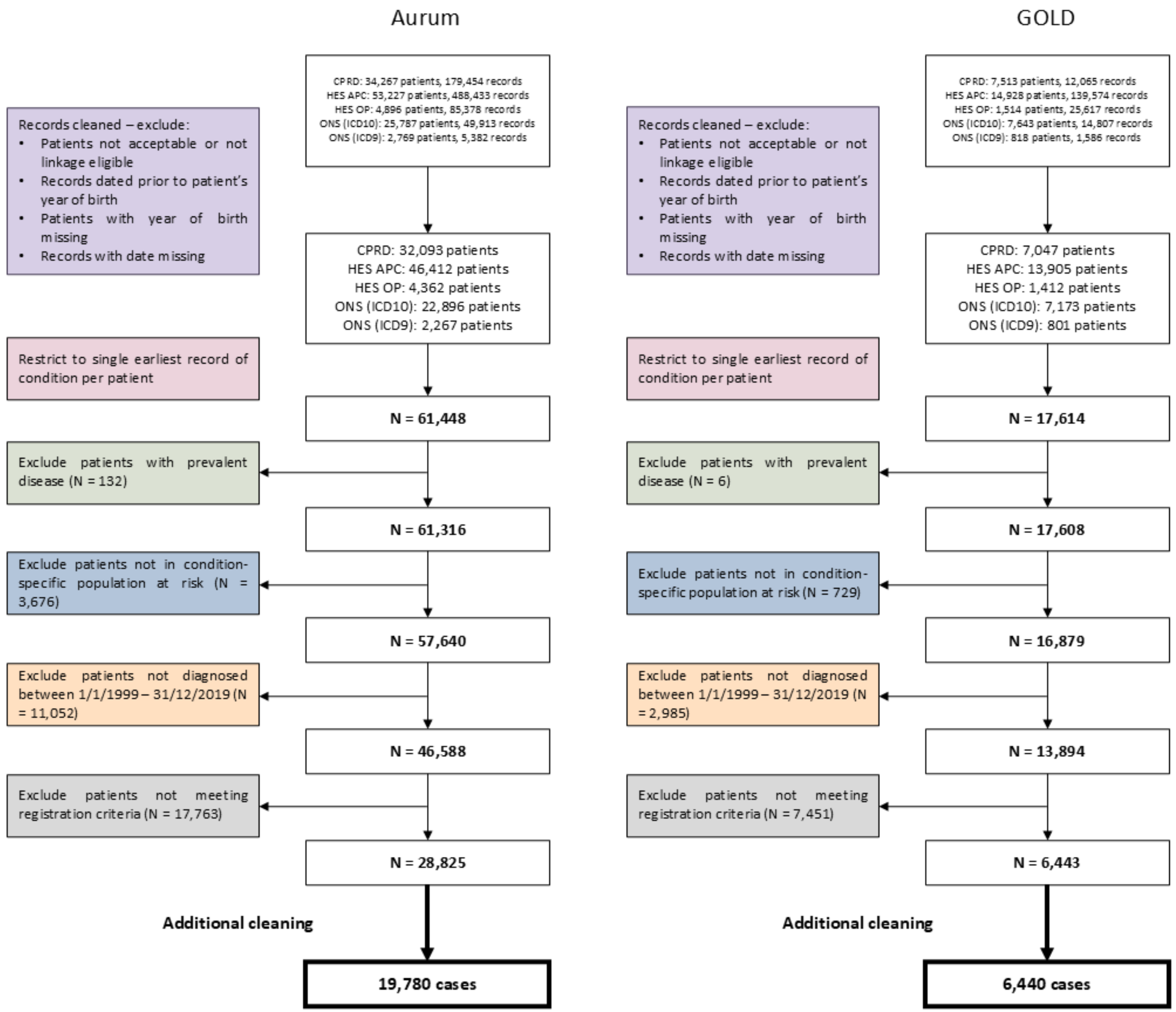

Figure 11. Flowcharts of patient selection for ovarian cancer

Pancreatic cancer

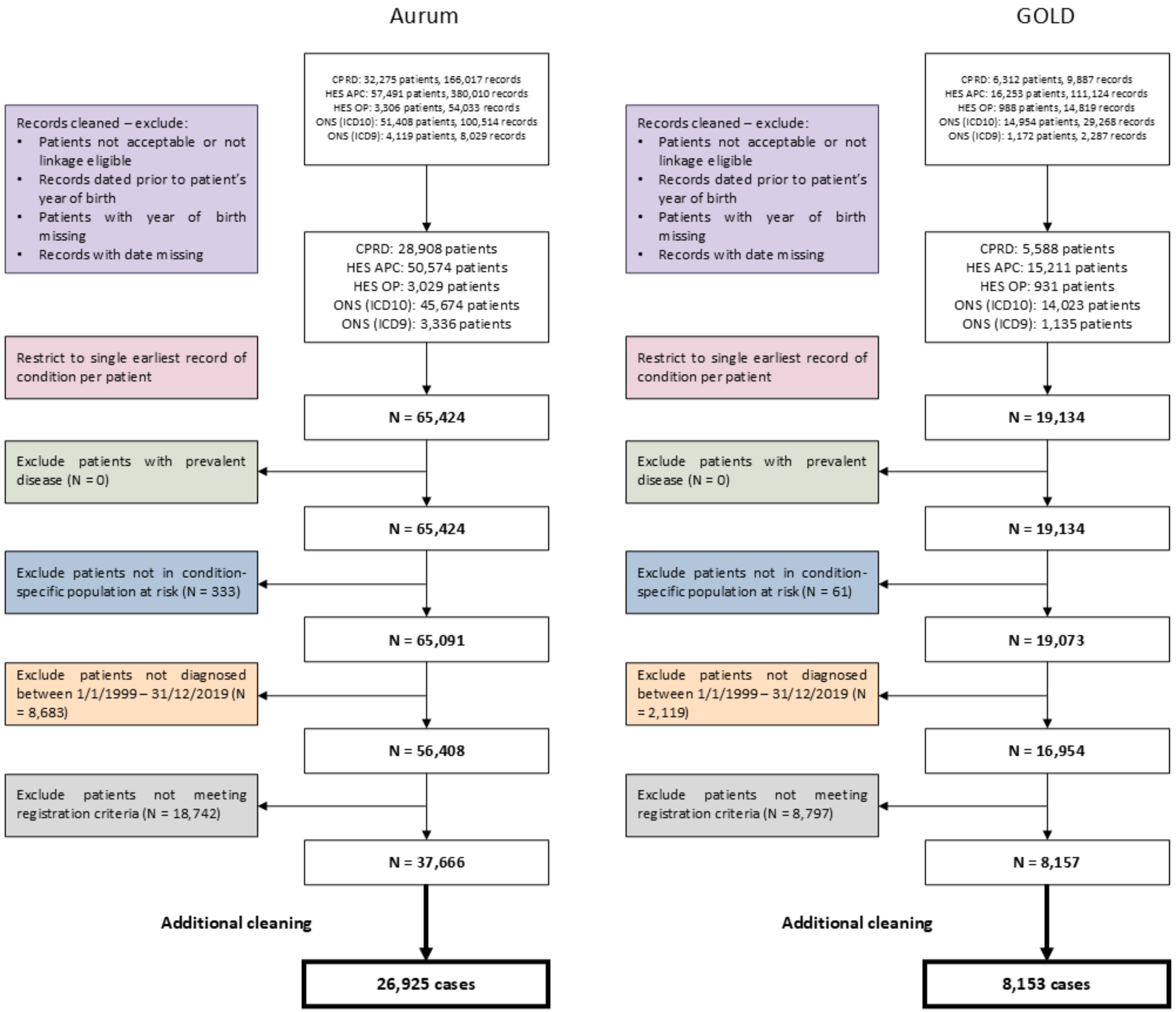

Figure 12. Flowcharts of patient selection for pancreatic cancer

Parkinson's disease

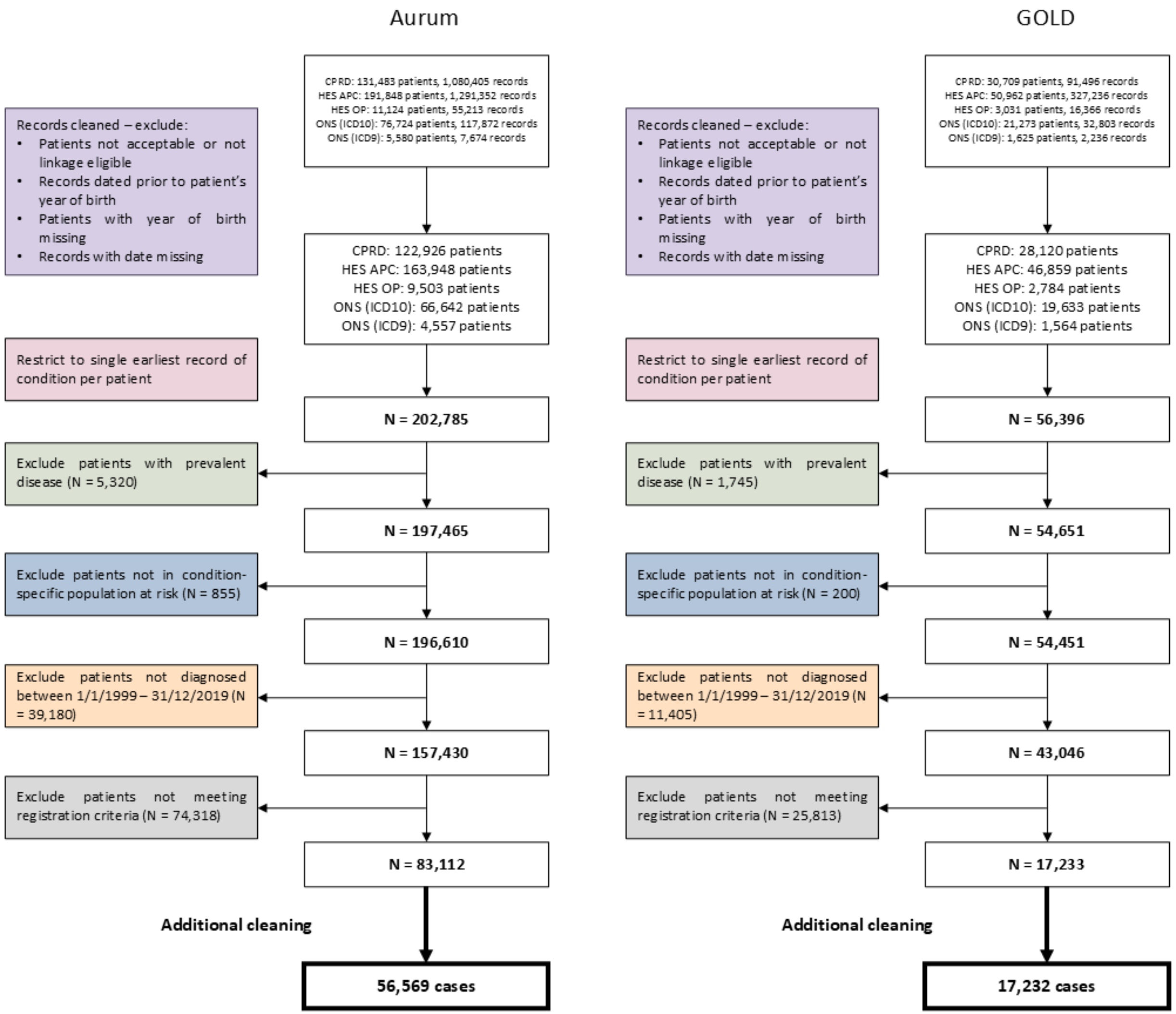

Figure 13. Flowcharts of patient selection for Parkinson's disease

Polycystic ovary syndrome

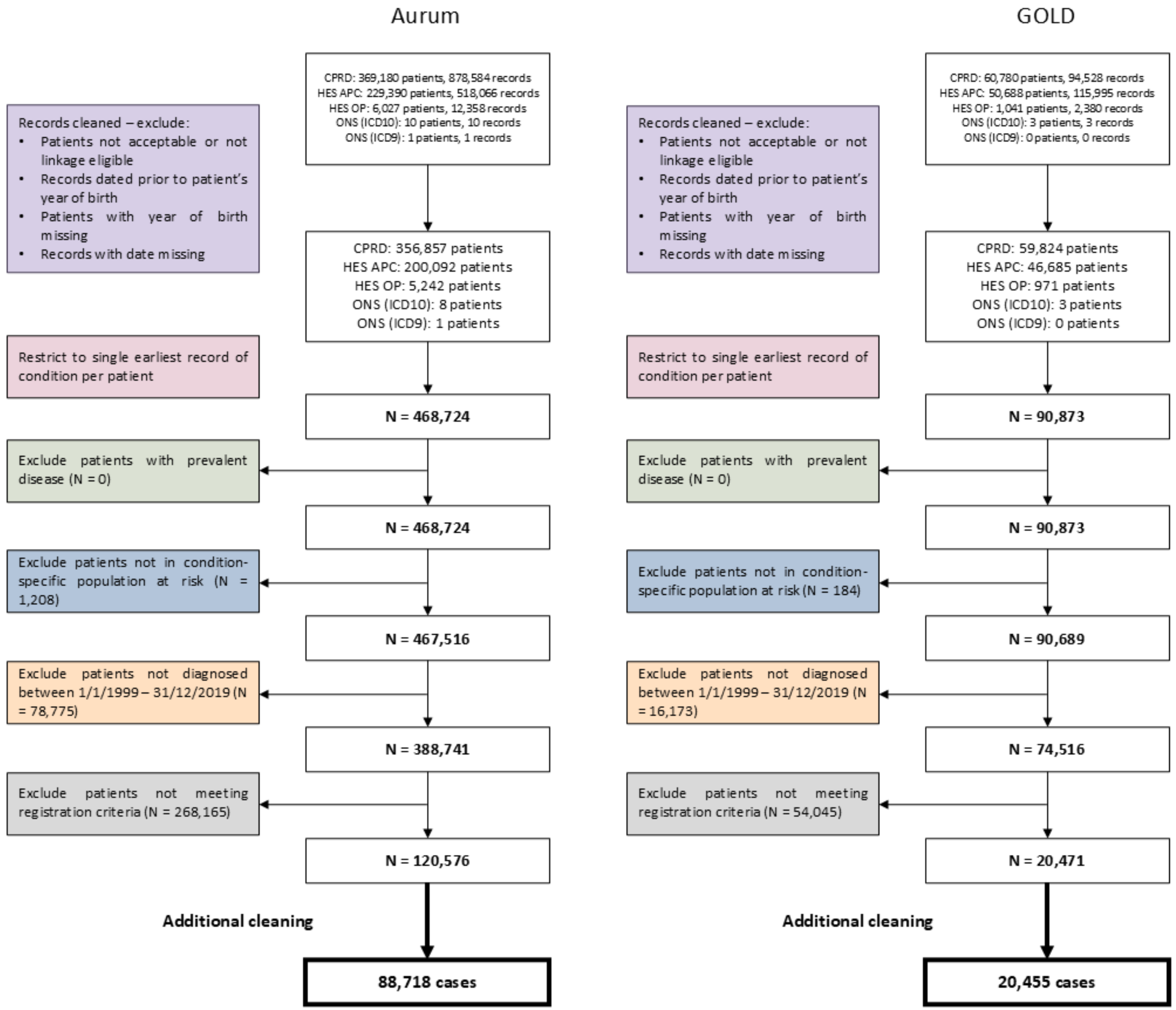

Figure 14. Flowcharts of patient selection for polycystic ovary syndrome (PCOS)

Rheumatoid arthritis

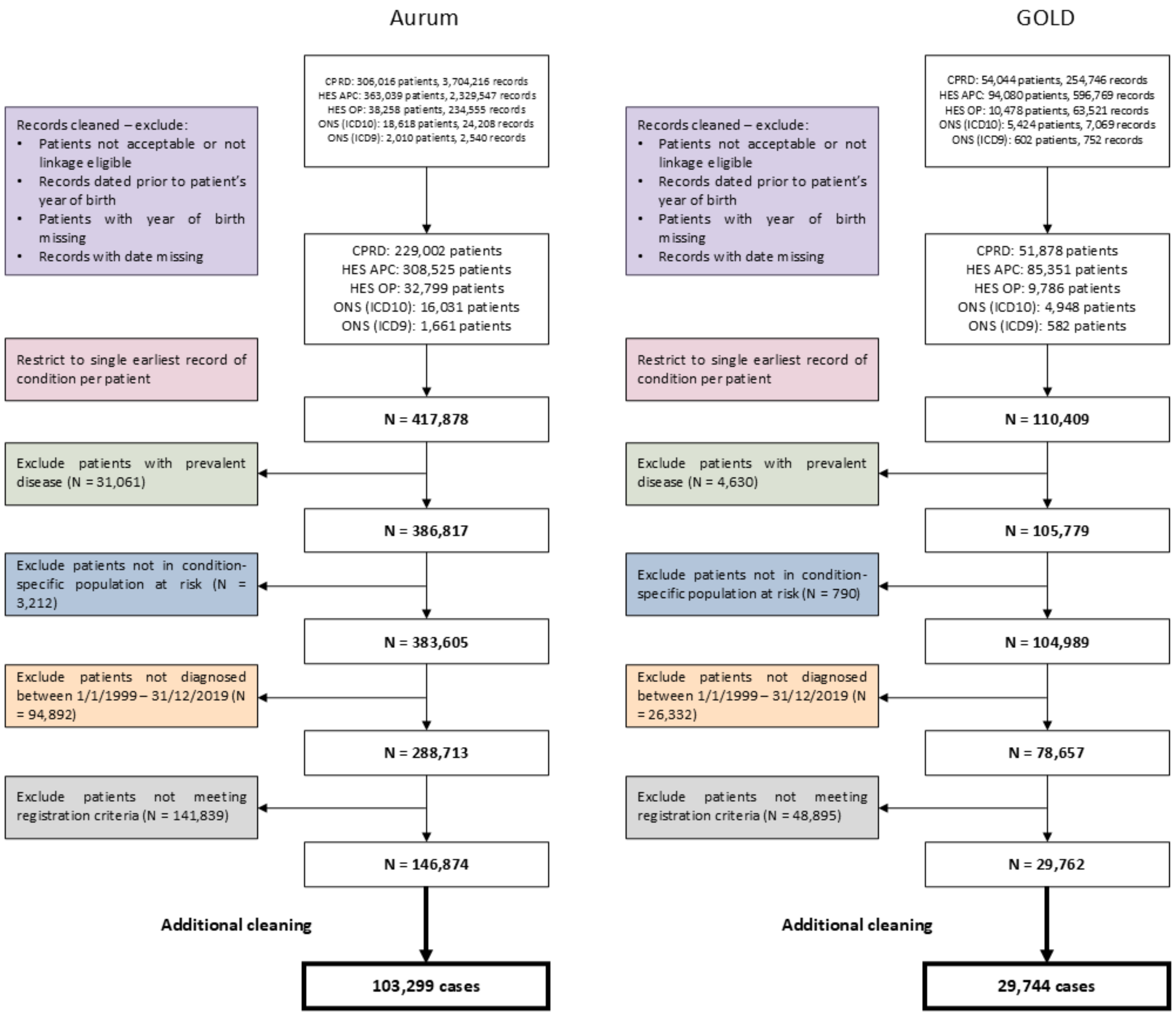

Figure 15 Flowcharts of patient selection for rheumatoid arthritis

Subacute bacterial endocarditis

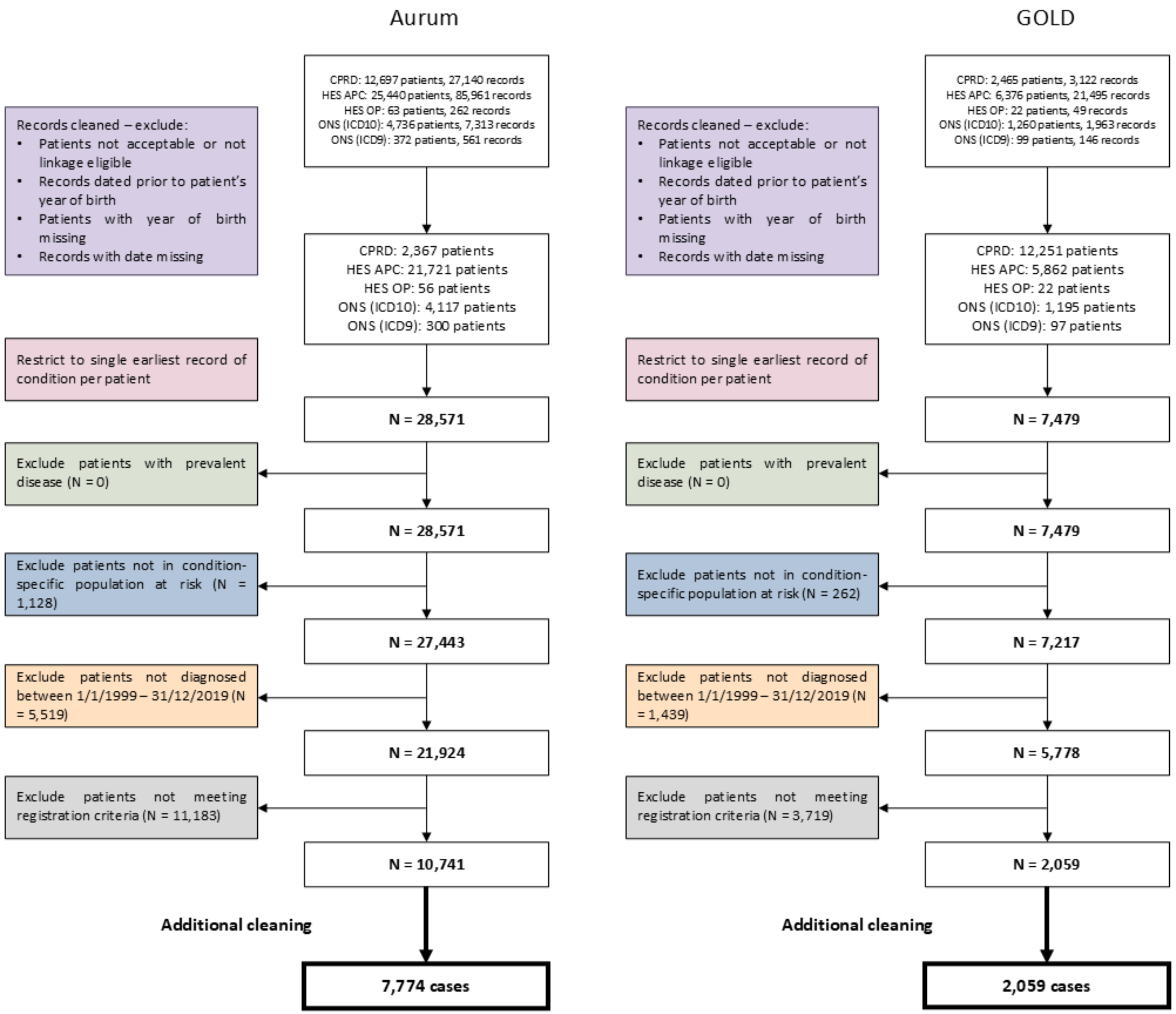

Figure 16. Flowcharts of patient selection for subacute bacterial endocarditis (SBE)

Schizophrenia, and other chronic psychoses

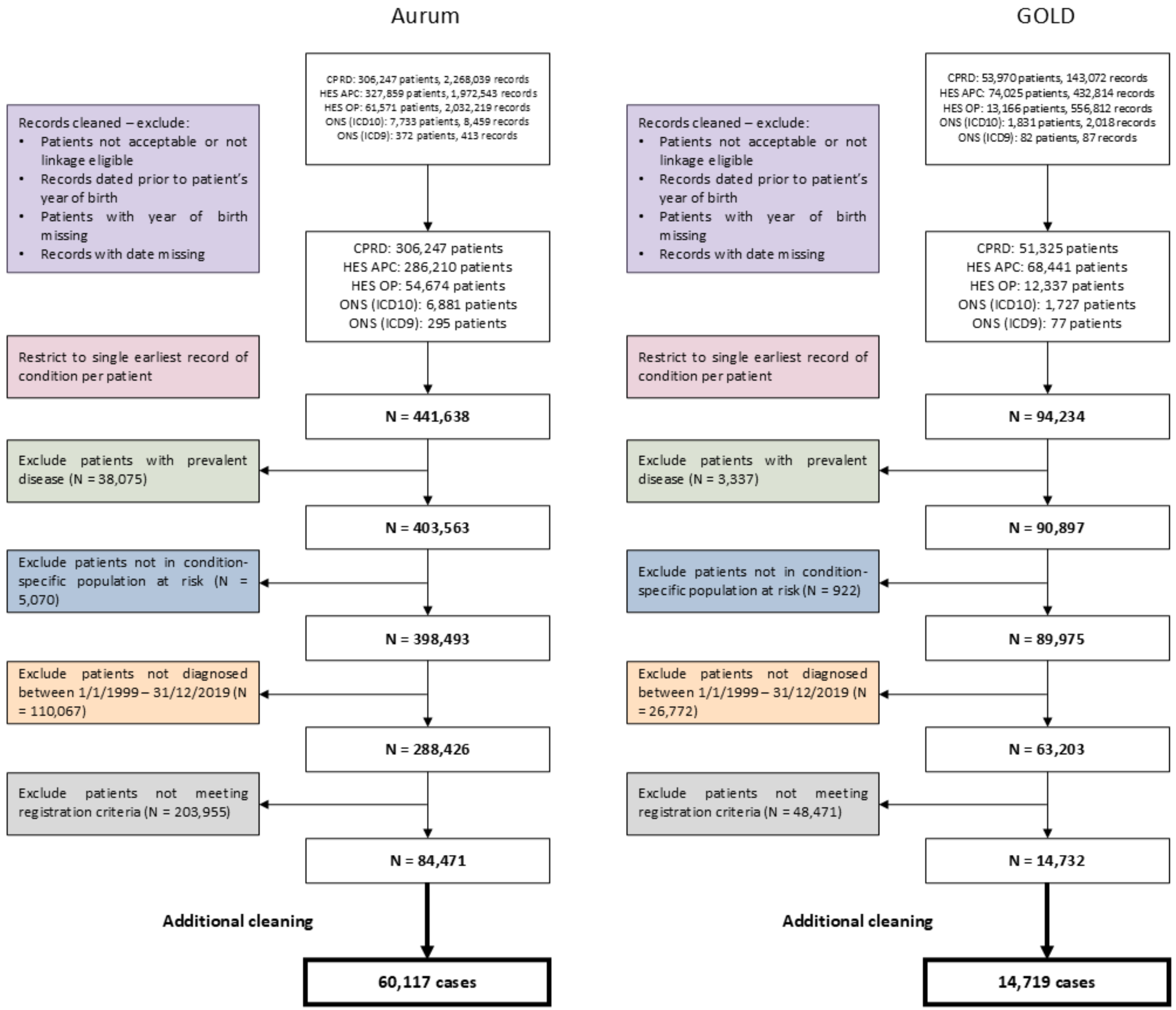

Figure 17. Flowcharts of patient selection for schizophrenia

Tuberculosis

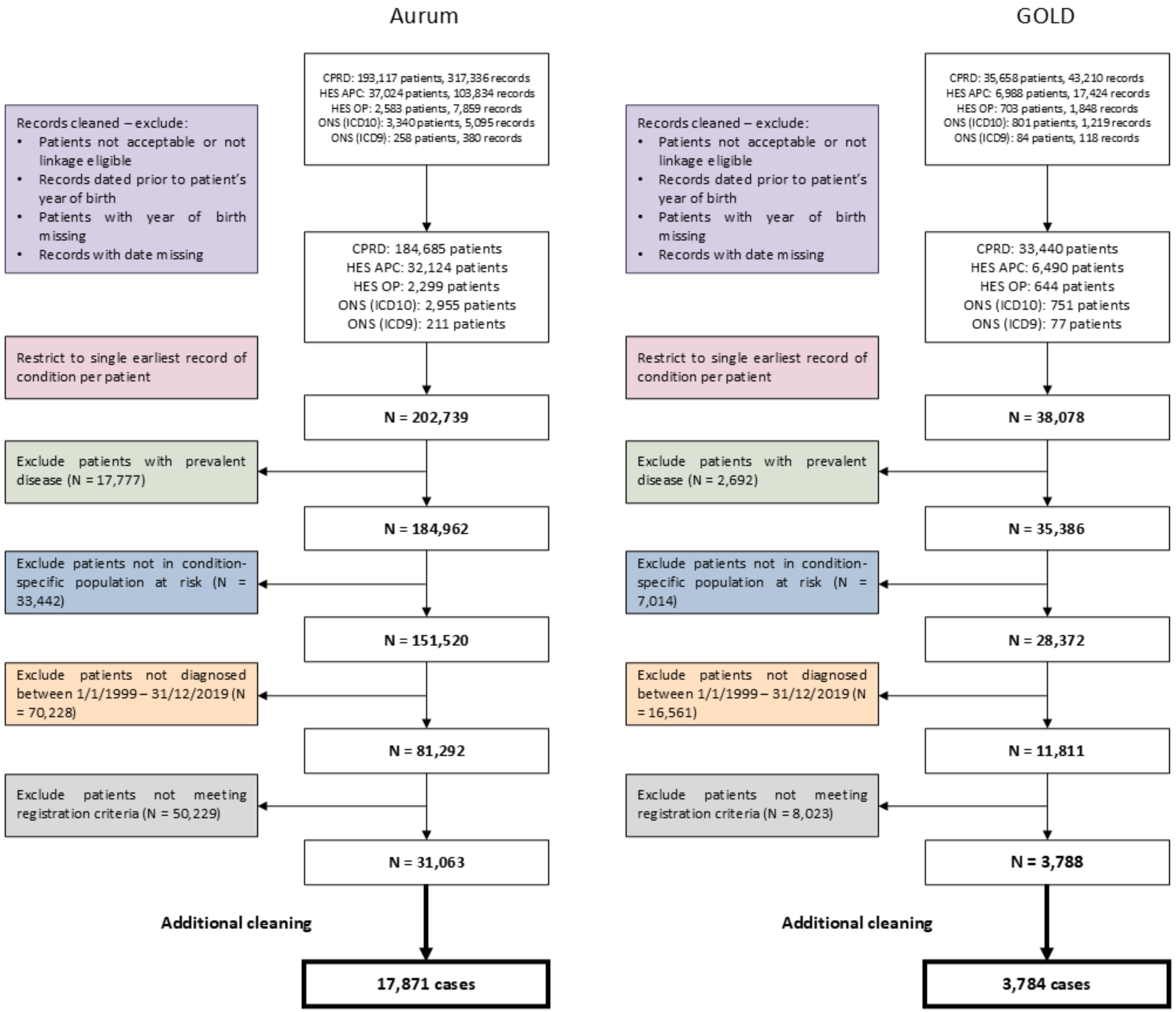

Figure 18. Flowcharts of patient selection for tuberculosis

Patient characteristics

Note that in all patient characteristics tables cells containing fewer than 10 patients have been suppressed (indicated by '-') to reduce the possibility of re-identification. Where this has occurred, the next smallest cell in the group has also been suppressed to prevent re-calculation of the cell size.

Table 3. Patient characteristics for men with axial spondyloarthritis. Note that cells containing fewer than 10 patients have been suppressed (indicated by '-'). Where this has occurred, the next smallest cell in the group has also been suppressed to prevent re-calculation.

Axial spondyloarthritis	Aurum		GOLD	
	**ED**	**Non-ED**	**ED**	**Non-ED**
Men	(N=1,270)	(N=5,619)	(N=329)	(N=1,804)
**Age at diagnosis**				
Mean (SD)	65.1 (17.7)	47.2 (17.1)	65.0 (17.4)	49.0 (17.3)
Median (IQR)	68.0 (54.0, 79.0)	45.0 (33.0, 59.0)	67.0 (54.0, 79.0)	48.0 (35.0, 63.0)
**Comorbidity score**				
Mean (SD)	2.5 (2.1)	1.1 (1.4)	1.9 (1.8)	1.0 (1.4)
Median (IQR)	2.0 (1.0, 4.0)	1.0 (0.0, 2.0)	1.0 (0.0, 3.0)	1.0 (0.0, 1.0)
**Deprivation quintile, n (%)**				
1 – Most deprived	225 (18%)	1,132 (20%)	48 (15%)	399 (22%)
2	226 (18%)	1,086 (19%)	78 (24%)	416 (23%)
3	237 (19%)	1,134 (20%)	61 (19%)	394 (22%)
4	261 (21%)	1,140 (20%)	66 (20%)	313 (17%)
5 – Least deprived	321 (25%)	1,114 (20%)	76 (23%)	-
Missing		13 (0.2%)		-
**Diagnosis source, n (%)**				
CPRD	66 (5.2%)	4,269 (76%)	26 (7.9%)	1,492 (83%)
HES APC	1,194 (94%)	1,064 (19%)	300 (91%)	259 (14%)
HES OP	-	-	-	-
ONS	-	-	-	-

Table 4. Patient characteristics for women with axial spondyloarthritis. Note that cells containing fewer than 10 patients have been suppressed (indicated by '-'). Where this has occurred, the next smallest cell in the group has also been suppressed to prevent re-calculation.

Axial spondyloarthritis	Aurum		GOLD	
	**ED**	**Non-ED**	**ED**	**Non-ED**
Women	(N=1,149)	(N=3,893)	(N=311)	(N=1,434)
**Age at diagnosis**				
Mean (SD)	66.6 (18.4)	51.3 (17.6)	68.0 (17.3)	54.1 (17.3)
Median (IQR)	71.0 (54.0, 81.0)	50.0 (37.0, 66.0)	72.0 (58.0, 82.0)	55.0 (40.0, 67.0)
**Comorbidity score**				
Mean (SD)	2.7 (2.1)	1.6 (1.6)	2.3 (1.7)	1.3 (1.4)
Median (IQR)	2.0 (1.0, 4.0)	1.0 (0.0, 2.0)	2.0 (1.0, 3.0)	1.0 (0.0, 2.0)
**Deprivation quintile, n (%)**				
1 – Most deprived	193 (17%)	798 (20%)	62 (20%)	307 (21%)
2	206 (18%)	806 (21%)	68 (22%)	317 (22%)
3	210 (18%)	756 (19%)	67 (22%)	290 (20%)
4	241 (21%)	768 (20%)	56 (18%)	-
5 – Least deprived	299 (26%)	753 (19%)	58 (19%)	261 (18%)
Missing		12 (0.3%)		-
**Diagnosis source, n (%)**				
CPRD	31 (2.7%)	2,126 (55%)	16 (5.1%)	1,037 (72%)
HES APC	1,110 (97%)	1,538 (40%)	293 (94%)	330 (23%)
HES OP	-	-	-	67 (4.7%)
ONS	-	-	-	

Table 5. Patient characteristics for men with brain tumours. Note that cells containing fewer than 10 patients have been suppressed (indicated by '-'). Where this has occurred, the next smallest cell in the group has also been suppressed to prevent re-calculation.

Brain tumours	**Aurum**		**GOLD**	
Men	**ED** (N=9,011)	**Non-ED** (N=4,919)	**ED** (N=2,546)	**Non-ED** (N=1,355)
**Age at diagnosis**				
Mean (SD)	64.6 (17.3)	59.5 (18.1)	64.9 (16.3)	59.7 (17.9)
Median (IQR)	67.0 (55.0, 77.0)	62.0 (48.0, 73.0)	67.0 (56.0, 77.0)	63.0 (49.0, 73.0)
**Comorbidity score**				
Mean (SD)	1.8 (1.9)	1.6 (1.7)	1.6 (1.7)	1.4 (1.6)
Median (IQR)	1.0 (0.0, 3.0)	1.0 (0.0, 2.0)	1.0 (0.0, 2.0)	1.0 (0.0, 2.0)
**Deprivation quintile, n (%)**				
1 – Most deprived	1,940 (22%)	1,143 (23%)	597 (23%)	323 (24%)
2	1,966 (22%)	1,089 (22%)	595 (23%)	334 (25%)
3	1,728 (19%)	904 (18%)	543 (21%)	298 (22%)
4	1,691 (19%)	908 (18%)	442 (17%)	220 (16%)
5 – Least deprived	-	-	-	180 (13%)
Missing	-	-	-	
**Diagnosis source, n (%)**				
CPRD	1,170 (13%)	2,733 (56%)	290 (11%)	712 (53%)
HES APC	7,609 (84%)	1,471 (30%)	2,200 (86%)	473 (35%)
HES OP	91 (1.0%)	422 (8.6%)	-	69 (5.1%)
ONS	141 (1.6%)	293 (6.0%)	-	101 (7.5%)

Table 6. Patient characteristics for women with brain tumours. Note that cells containing fewer than 10 patients have been suppressed (indicated by '-'). Where this has occurred, the next smallest cell in the group has also been suppressed to prevent re-calculation.

Brain tumours	Aurum		GOLD	
	**ED**	**Non-ED**	**ED**	**Non-ED**
Women	(N=10,777)	(N=8,249)	(N=2,923)	(N=2,123)
**Age at diagnosis**				
Mean (SD)	68.7 (18.0)	61.3 (17.3)	68.8 (17.3)	61.4 (16.9)
Median (IQR)	72.0 (58.0, 83.0)	63.0 (49.0, 75.0)	72.0 (58.0, 82.0)	62.0 (49.0, 75.0)
**Comorbidity score**				
Mean (SD)	2.2 (2.0)	1.7 (1.7)	1.8 (1.7)	1.4 (1.5)
Median (IQR)	2.0 (1.0, 3.0)	1.0 (0.0, 3.0)	1.0 (0.0, 3.0)	1.0 (0.0, 2.0)
**Deprivation quintile, n (%)**				
1 – Most deprived	2,108 (20%)	1,841 (22%)	655 (22%)	537 (25%)
2	2,200 (20%)	1,761 (21%)	652 (22%)	498 (23%)
3	2,188 (20%)	1,549 (19%)	604 (21%)	429 (20%)
4	2,194 (20%)	-	549 (19%)	360 (17%)
5 – Least deprived	-	1,564 (19%)	-	-
Missing	-	-	-	-
**Diagnosis source, n (%)**				
CPRD	1,305 (12%)	5,604 (68%)	306 (10%)	1,387 (65%)
HES APC	9,243 (86%)	1,773 (21%)	2,550 (87%)	546 (26%)
HES OP	99 (0.9%)	550 (6.7%)	10 (0.3%)	66 (3.1%)
ONS	130 (1.2%)	322 (3.9%)	57 (2.0%)	124 (5.8%)

Table 7. Patient characteristics for men with coeliac disease. Note that cells containing fewer than 10 patients have been suppressed (indicated by '-'). Where this has occurred, the next smallest cell in the group has also been suppressed to prevent re-calculation.

Coeliac disease	Aurum		GOLD	
	**ED**	**Non-ED**	**ED**	**Non-ED**
Men	(N=830)	(N=8,490)	(N=221)	(N=2,405)
**Age at diagnosis**				
Mean (SD)	58.1 (20.9)	51.2 (18.9)	59.4 (20.6)	52.9 (17.9)
Median (IQR)	61.0 (43.0, 76.0)	53.0 (37.0, 66.0)	64.0 (44.0, 77.0)	55.0 (41.0, 66.0)
**Comorbidity score**				
Mean (SD)	2.0 (2.1)	1.2 (1.5)	1.7 (1.8)	1.1 (1.3)
Median (IQR)	1.0 (0.0, 3.0)	1.0 (0.0, 2.0)	1.0 (0.0, 3.0)	1.0 (0.0, 2.0)
**Deprivation quintile, n (%)**				
1 – Most deprived	150 (18%)	2,067 (24%)	39 (18%)	613 (25%)
2	177 (21%)	1,936 (23%)	55 (25%)	606 (25%)
3	-	1,669 (20%)	44 (20%)	466 (19%)
4	158 (19%)	1,451 (17%)	33 (15%)	389 (16%)
5 – Least deprived	206 (25%)	-	50 (23%)	-
Missing	-	-		-
**Diagnosis source, n (%)**				
CPRD	114 (14%)	5,577 (66%)	31 (14%)	1,620 (67%)
HES APC	713 (86%)	2,839 (33%)	190 (86%)	772 (32%)
HES OP	-	-		-
ONS	-	-		-

Table 8. Patient characteristics for women with coeliac disease. Note that cells containing fewer than 10 patients have been suppressed (indicated by '-'). Where this has occurred, the next smallest cell in the group has also been suppressed to prevent re-calculation.

Coeliac disease	Aurum		GOLD	
	**ED**	**Non-ED**	**ED**	**Non-ED**
Women	(N=1,739)	(N=17,470)	(N=400)	(N=4,576)
**Age at diagnosis**				
Mean (SD)	52.8 (22.0)	45.6 (18.5)	55.4 (22.1)	48.1 (18.6)
Median (IQR)	52.0 (34.0, 72.0)	44.0 (31.0, 60.0)	57.5 (37.0, 74.0)	47.0 (34.0, 63.0)
**Comorbidity score**				
Mean (SD)	1.8 (1.9)	1.1 (1.3)	1.7 (1.8)	1.1 (1.3)
Median (IQR)	1.0 (0.0, 3.0)	1.0 (0.0, 2.0)	1.0 (0.0, 2.0)	1.0 (0.0, 2.0)
**Deprivation quintile, n (%)**				
1 – Most deprived	-	4,089 (23%)	92 (23%)	1,193 (26%)
2	330 (19%)	3,824 (22%)	83 (21%)	1,039 (23%)
3	380 (22%)	3,366 (19%)	64 (16%)	944 (21%)
4	341 (20%)	3,244 (19%)	79 (20%)	792 (17%)
5 – Least deprived	370 (21%)	2,929 (17%)	82 (21%)	-
Missing	-	18 (0.1%)		-
**Diagnosis source, n (%)**				
CPRD	196 (11%)	11,100 (64%)	47 (12%)	2,961 (65%)
HES APC	1,537 (88%)	6,239 (36%)	353 (88%)	1,585 (35%)
HES OP	-	-		-
ONS	-	-		-

Table 9. Patient characteristics for men with CHD. Note that cells containing fewer than 10 patients have been suppressed (indicated by '-'). Where this has occurred, the next smallest cell in the group has also been suppressed to prevent re-calculation.

CHD	**Aurum**		**GOLD**	
	**ED**	**Non-ED**	**ED**	**Non-ED**
Men	(N=173,463)	(N=175,622)	(N=51,301)	(N=52,782)
**Age at diagnosis**				
Mean (SD)	67.5 (14.3)	67.3 (12.4)	67.5 (14.2)	67.6 (12.4)
Median (IQR)	68.0 (57.0, 79.0)	68.0 (59.0, 76.0)	68.0 (57.0, 79.0)	68.0 (59.0, 77.0)
**Comorbidity score**				
Mean (SD)	1.9 (1.8)	1.8 (1.7)	1.6 (1.6)	1.5 (1.5)
Median (IQR)	2.0 (0.0, 3.0)	2.0 (1.0, 3.0)	1.0 (0.0, 2.0)	1.0 (0.0, 2.0)
**Deprivation quintile, n (%)**				
1 – Most deprived	32,536 (19%)	36,726 (21%)	10,021 (20%)	11,507 (22%)
2	33,344 (19%)	36,136 (21%)	10,941 (21%)	11,615 (22%)
3	33,088 (19%)	33,982 (19%)	10,801 (21%)	11,251 (21%)
4	35,229 (20%)	34,196 (19%)	9,935 (19%)	9,287 (18%)
5 – Least deprived	39,105 (23%)	34,391 (20%)	9,554 (19%)	9,060 (17%)
Missing	161 (0.1%)	191 (0.1%)	49 (0.1%)	62 (0.1%)
**Diagnosis source, n (%)**				
CPRD	7,908 (4.6%)	63,692 (36%)	-	20,784 (39%)
HES APC	154,983 (89%)	66,077 (38%)	45,406 (89%)	17,383 (33%)
HES OP	46 (0.0%)	741 (0.4%)	-	134 (0.3%)
ONS	10,526 (6.1%)	45,112 (26%)	3,357 (6.5%)	14,481 (27%)

Table 10. Patient characteristics for women with CHD. Note that cells containing fewer than 10 patients have been suppressed (indicated by '-'). Where this has occurred, the next smallest cell in the group has also been suppressed to prevent re-calculation.

CHD	Aurum		GOLD	
	**ED**	**Non-ED**	**ED**	**Non-ED**
Women	(N=144,550)	(N=123,421)	(N=42,649)	(N=39,090)
**Age at diagnosis**				
Mean (SD)	74.9 (14.1)	72.5 (12.8)	75.2 (13.9)	73.2 (12.8)
Median (IQR)	78.0 (66.0, 86.0)	74.0 (64.0, 82.0)	78.0 (66.0,, 86.0)	74.0 (65.0, 83.0)
**Comorbidity score**				
Mean (SD)	2.5 (1.9)	2.2 (1.8)	2.1 (1.7)	1.9 (1.6)
Median (IQR)	2.0 (1.0, 4.0)	2.0 (1.0, 3.0)	2.0 (1.0, 3.0)	2.0 (1.0, 3.0)
**Deprivation quintile, n (%)**				
1 – Most deprived	25,774 (18%)	24,287 (20%)	7,835 (18%)	7,711 (20%)
2	27,358 (19%)	24,579 (20%)	8,792 (21%)	8,411 (22%)
3	27,472 (19%)	23,932 (19%)	9,004 (21%)	8,359 (21%)
4	29,996 (21%)	25,048 (20%)	8,499 (20%)	7,463 (19%)
5 – Least deprived	33,835 (23%)	25,447 (21%)	8,477 (20%)	7,098 (18%)
Missing	115 (0.1%)	128 (0.1%)	42 (0.1%)	48 (0.1%)
**Diagnosis source, n (%)**				
CPRD	6,922 (4.8%)	42,672 (35%)	-	15,434 (39%)
HES APC	126,732 (88%)	47,659 (39%)	37,034 (87%)	12,024 (31%)
HES OP	30 (0.0%)	629 (0.5%)	-	119 (0.3%)
ONS	10,866 (7.5%)	32,461 (26%)	3,541 (8.3%)	11,513 (29%)

Table 11. Patient characteristics for men with colon cancer. Note that cells containing fewer than 10 patients have been suppressed (indicated by '-'). Where this has occurred, the next smallest cell in the group has also been suppressed to prevent re-calculation.

Colon cancer	Aurum		GOLD	
	**ED**	**Non-ED**	**ED**	**Non-ED**
Men	(N=13,277)	(N=27,872)	(N=4,194)	(N=8,725)
**Age at diagnosis**				
Mean (SD)	73.0 (12.6)	70.9 (11.3)	73.2 (12.1)	71.1 (11.0)
Median (IQR)	75.0 (65.0, 82.0)	72.0 (64.0, 79.0)	75.0 (66.0, 82.0)	72.0 (64.0, 79.0)
**Comorbidity score**				
Mean (SD)	2.3 (2.0)	2.1 (1.8)	2.0 (1.8)	1.8 (1.7)
Median (IQR)	2.0 (1.0, 3.0)	2.0 (1.0, 3.0)	2.0 (1.0, 3.0)	1.0 (1.0, 3.0)
**Deprivation quintile, n (%)**				
1 – Most deprived	2,678 (20%)	6,680 (24%)	933 (22%)	2,167 (25%)
2	2,726 (21%)	6,203 (22%)	944 (23%)	2,040 (23%)
3	2,604 (20%)	5,520 (20%)	860 (21%)	1,883 (22%)
4	-	5,060 (18%)	771 (18%)	1,499 (17%)
5 – Least deprived	2,711 (20%)	4,380 (16%)	-	-
Missing	-	29 (0.1%)	-	-
**Diagnosis source, n (%)**				
CPRD	1,552 (12%)	9,634 (35%)	414 (9.9%)	2,803 (32%)
HES APC	10,881 (82%)	16,503 (59%)	3,449 (82%)	5,232 (60%)
HES OP	41 (0.3%)	406 (1.5%)	-	149 (1.7%)
ONS	803 (6.0%)	1,329 (4.8%)	-	541 (6.2%)

Table 12. Patient characteristics for women with colon cancer. Note that cells containing fewer than 10 patients have been suppressed (indicated by '-'). Where this has occurred, the next smallest cell in the group has also been suppressed to prevent re-calculation.

Colon cancer	**Aurum**		**GOLD**	
	**ED**	**Non-ED**	**ED**	**Non-ED**
Women	(N=13,137)	(N=23,428)	(N=4,096)	(N=7,633)
**Age at diagnosis**				
Mean (SD)	75.3 (13.6)	72.2 (12.4)	75.4 (13.0)	72.6 (12.0)
Median (IQR)	78.0 (68.0, 85.0)	74.0 (64.0, 81.0)	78.0 (68.0, 85.0)	74.0 (65.0, 82.0)
**Comorbidity score**				
Mean (SD)	2.2 (1.9)	2.0 (1.7)	2.0 (1.8)	1.8 (1.6)
Median (IQR)	2.0 (1.0, 3.0)	2.0 (2.01.0, 3.0)	2.0 (1.0, 3.0)	1.0 (0.0, 3.0)
**Deprivation quintile, n (%)**				
1 – Most deprived	2,725 (21%)	5,668 (24%)	839 (20%)	1,902 (25%)
2	2,670 (20%)	5,374 (23%)	894 (22%)	1,837 (24%)
3	2,581 (20%)	4,684 (20%)	890 (22%)	1,658 (22%)
4	2,577 (20%)	4,115 (18%)	774 (19%)	1,264 (17%)
5 – Least deprived	-	3,567 (15%)	-	-
Missing	-	20 (0.1%)	-	-
**Diagnosis source, n (%)**				
CPRD	1,475 (11%)	8,259 (35%)	431 (11%)	2,406 (32%)
HES APC	10,879 (83%)	13,345 (57%)	3,401 (83%)	4,392 (58%)
HES OP	33 (0.3%)	336 (1.4%)	10 (0.2%)	179 (2.3%)
ONS	750 (5.7%)	1,488 (6.4%)	254 (6.2%)	656 (8.6%)

Table 13. Patient characteristics for men with COPD. Note that cells containing fewer than 10 patients have been suppressed (indicated by '-'). Where this has occurred, the next smallest cell in the group has also been suppressed to prevent re-calculation.

COPD	**Aurum**		**GOLD**	
	**ED**	**Non-ED**	**ED**	**Non-ED**
Men	(N=73,398)	(N=133,599)	(N=20,487)	(N=34,720)
**Age at diagnosis**				
Mean (SD)	71.9 (13.4)	67.2 (11.7)	72.7 (13.0)	68.2 (11.4)
Median (IQR)	74.0 (63.0, 82.0)	68.0 (59.0, 76.0)	75.0 (64.0, 82.0)	69.0 (61.0, 77.0)
**Comorbidity score**				
Mean (SD)	2.6 (2.1)	2.0 (1.7)	2.3 (1.9)	1.7 (1.6)
Median (IQR)	2.0 (1.0, 4.0)	2.0 (1.0, 3.0)	2.0 (1.0, 3.0)	1.0 (1.0, 3.0)
**Deprivation quintile, n (%)**				
1 – Most deprived	10,135 (14%)	20,847 (16%)	3,076 (15%)	5,574 (16%)
2	12,114 (17%)	23,912 (18%)	3,703 (18%)	6,897 (20%)
3	13,513 (18%)	25,319 (19%)	4,199 (20%)	7,068 (20%)
4	16,178 (22%)	28,564 (21%)	4,472 (22%)	7,233 (21%)
5 – Least deprived	21,387 (29%)	34,856 (26%)	5,023 (25%)	7,904 (23%)
Missing	71 (0.1%)	101 (0.1%)	14 (0.1%)	44 (0.1%)
**Diagnosis source, n (%)**				
CPRD	2,876 (3.9%)	100,475 (75%)	844 (4.1%)	25,638 (74%)
HES APC	68,658 (94%)	27,448 (21%)	19,038 (93%)	7,253 (21%)
HES OP	44 (0.1%)	697 (0.5%)	10 (0.0%)	176 (0.5%)
ONS	1,820 (2.5%)	4,979 (3.7%)	595 (2.9%)	1,653 (4.8%)

Table 14. Patient characteristics for women with COPD. Note that cells containing fewer than 10 patients have been suppressed (indicated by '-'). Where this has occurred, the next smallest cell in the group has also been suppressed to prevent re-calculation.

COPD	**Aurum**		**GOLD**	
	**ED**	**Non-ED**	**ED**	**Non-ED**
Women	(N=74,070)	(N=113,391)	(N=20,675)	(N=29,955)
**Age at diagnosis**				
Mean (SD)	74.0 (14.1)	67.0 (12.2)	74.6 (13.6)	67.9 (12.3)
Median (IQR)	77.0 (65.0, 85.0)	68.0 (59.0, 76.0)	77.0 (66.0, 85.0)	69.0 (60.0, 77.0)
**Comorbidity score**				
Mean (SD)	2.8 (2.0)	2.0 (1.7)	2.5 (1.9)	1.8 (1.6)
Median (IQR)	2.0 (1.0, 4.0)	2.0 (1.0, 3.0)	2.0 (1.0, 4.0)	1.0 (1.0, 3.0)
**Deprivation quintile, n (%)**				
1 – Most deprived	10,197 (14%)	16,348 (14%)	2,939 (14%)	4,462 (15%)
2	12,090 (16%)	19,326 (17%)	3,729 (18%)	5,665 (19%)
3	13,395 (18%)	20,826 (18%)	4,182 (20%)	6,087 (20%)
4	16,208 (22%)	24,925 (22%)	4,499 (22%)	6,352 (21%)
5 – Least deprived	22,114 (30%)	31,871 (28%)	5,306 (26%)	7,363 (25%)
Missing	66 (0.1%)	95 (0.1%)	20 (0.1%)	26 (0.1%)
**Diagnosis source, n (%)**				
CPRD	2,760 (3.7%)	84,661 (75%)	759 (3.7%)	22,048 (74%)
HES APC	69,846 (94%)	25,150 (22%)	19,397 (94%)	6,617 (22%)
HES OP	46 (0.1%)	636 (0.6%)	13 (0.1%)	169 (0.6%)
ONS	1,418 (1.9%)	2,944 (2.6%)	506 (2.4%)	1,121 (3.7%)

Table 15. Patient characteristics for men with IBD. Note that cells containing fewer than 10 patients have been suppressed (indicated by '-'). Where this has occurred, the next smallest cell in the group has also been suppressed to prevent re-calculation.

IBD	**Aurum**		**GOLD**	
	**ED**	**Non-ED**	**ED**	**Non-ED**
Men	(N=8,594)	(N=27,727)	(N=2,409)	(N=8,171)
**Age at diagnosis**				
Mean (SD)	50.3 (22.2)	48.5 (18.7)	51.8 (22.7)	49.9 (18.9)
Median (IQR)	50.0 (30.0, 70.0)	49.0 (33.0, 64.0)	52.0 (31.0, 72.0)	50.0 (35.0, 65.0)
**Comorbidity score**				
Mean (SD)	1.5 (1.9)	1.1 (1.5)	1.4 (1.7)	1.0 (1.3)
Median (IQR)	1.0 (0.0, 2.0)	1.0 (0.0, 2.0)	1.0 (0.0, 2.0)	1.0 (0.0, 1.0)
**Deprivation quintile, n (%)**				
1 – Most deprived	1,467 (17%)	6,046 (22%)	-	2,035 (25%)
2	1,560 (18%)	5,836 (21%)	464 (19%)	1,836 (22%)
3	1,645 (19%)	5,595 (20%)	485 (20%)	1,753 (21%)
4	1,896 (22%)	5,431 (20%)	524 (22%)	1,408 (17%)
5 – Least deprived	2,016 (23%)	4,803 (17%)	474 (20%)	-
Missing	10 (0.1%)	16 (0.1%)	-	-
**Diagnosis source, n (%)**				
CPRD	1,215 (14%)	12,224 (44%)	378 (16%)	4,136 (51%)
HES APC	7,342 (85%)	14,686 (53%)	2,014 (84%)	3,927 (48%)
HES OP	17 (0.2%)	784 (2.8%)	-	-
ONS	20 (0.2%)	33 (0.1%)	-	-

Table 16. Patient characteristics for women with IBD. Note that cells containing fewer than 10 patients have been suppressed (indicated by '-'). Where this has occurred, the next smallest cell in the group has also been suppressed to prevent re-calculation.

IBD	Aurum		GOLD	
	**ED**	**Non-ED**	**ED**	**Non-ED**
Women	(N=10,516)	(N=29,940)	(N=3,062)	(N=9,281)
**Age at diagnosis**				
Mean (SD)	55.4 (23.2)	49.4 (19.0)	55.3 (23.3)	50.8 (19.1)
Median (IQR)	57.0 (34.0, 76.0)	49.0 (33.0, 65.0)	57.5 (34.0, 76.0)	51.0 (35.0, 66.0)
**Comorbidity score**				
Mean (SD)	1.8 (2.0)	1.2 (1.5)	1.6 (1.7)	1.0 (1.3)
Median (IQR)	1.0 (0.0, 3.0)	1.0 (0.0, 2.0)	1.0 (0.0, 2.0)	1.0 (0.0, 2.0)
**Deprivation quintile, n (%)**				
1 – Most deprived	1,801 (17%)	6,570 (22%)	-	2,208 (24%)
2	2,002 (19%)	6,296 (21%)	618 (20%)	2,104 (23%)
3	2,011 (19%)	5,927 (20%)	649 (21%)	1,936 (21%)
4	2,254 (21%)	5,893 (20%)	641 (21%)	1,603 (17%)
5 – Least deprived	2,434 (23%)	5,231 (17%)	606 (20%)	-
Missing	14 (0.1%)	23 (0.1%)	-	-
**Diagnosis source, n (%)**				
CPRD	1,506 (14%)	13,166 (44%)	504 (16%)	4,989 (54%)
HES APC	8,963 (85%)	15,715 (52%)	2,542 (83%)	4,159 (45%)
HES OP	14 (0.1%)	1,018 (3.4%)	-	119 (1.3%)
ONS	33 (0.3%)	41 (0.1%)	-	14 (0.2%)

Table 17. Patient characteristics for men with lung cancer

Lung cancer	**Aurum**		**GOLD**	
	**ED**	**Non-ED**	**ED**	**Non-ED**
Men	(N=27,602)	(N=34,954)	(N=8,524)	(N=10,894)
**Age at diagnosis**				
Mean (SD)	73.4 (11.0)	71.7 (10.3)	73.4 (10.9)	71.6 (10.3)
Median (IQR)	74.0 (66.0, 81.0)	72.0 (65.0, 79.0)	75.0 (67.0, 81.0)	72.0 (65.0, 79.0)
**Comorbidity score**				
Mean (SD)	2.6 (2.1)	2.4 (2.0)	2.2 (1.9)	2.0 (1.8)
Median (IQR)	2.0 (1.0, 4.0)	2.0 (1.0, 4.0)	2.0 (1.0, 3.0)	2.0 (1.0, 3.0)
**Deprivation quintile, n (%)**				
1 – Most deprived	4,229 (15%)	5,973 (17%)	1,376 (16%)	1,943 (18%)
2	4,787 (17%)	6,470 (19%)	1,680 (20%)	2,148 (20%)
3	5,047 (18%)	6,611 (19%)	1,697 (20%)	2,255 (21%)
4	5,943 (22%)	7,333 (21%)	1,751 (21%)	2,267 (21%)
5 – Least deprived	7,572 (27%)	8,531 (24%)	2,008 (24%)	2,263 (21%)
Missing	24 (0.1%)	36 (0.1%)	12 (0.1%)	18 (0.2%)
**Diagnosis source, n (%)**				
CPRD	3,423 (12%)	15,437 (44%)	882 (10%)	4,012 (37%)
HES APC	22,256 (81%)	15,815 (45%)	6,925 (81%)	5,404 (50%)
HES OP	142 (0.5%)	775 (2.2%)	34 (0.4%)	197 (1.8%)
ONS	1,781 (6.5%)	2,927 (8.4%)	683 (8.0%)	1,281 (12%)

Table 18. Patient characteristics for women with lung cancer. Note that cells containing fewer than 10 patients have been suppressed (indicated by '-'). Where this has occurred, the next smallest cell in the group has also been suppressed to prevent re-calculation.

Lung cancer	Aurum		GOLD	
	**ED**	**Non-ED**	**ED**	**Non-ED**
Women	(N=22,282)	(N=28,230)	(N=6,598)	(N=8,355)
**Age at diagnosis**				
Mean (SD)	74.0 (11.7)	71.6 (11.0)	74.0 (11.5)	71.5 (11.1)
Median (IQR)	75.0 (66.0, 83.0)	72.0 (65.0, 80.0)	75.0 (67.0, 83.0)	72.0 (64.0, 80.0)
**Comorbidity score**				
Mean (SD)	2.6 (2.1)	2.4 (1.9)	2.2 (1.8)	2.0 (1.8)
Median (IQR)	2.0 (1.0, 4.0)	2.0 (1.0, 3.0)	2.0 (1.0, 3.0)	2.0 (1.0, 3.0)
**Deprivation quintile, n (%)**				
1 – Most deprived	3,279 (15%)	4,503 (16%)	1,062 (16%)	-
2	3,743 (17%)	5,179 (18%)	1,250 (19%)	1,636 (20%)
3	3,990 (18%)	5,164 (18%)	1,313 (20%)	1,732 (21%)
4	4,949 (22%)	5,909 (21%)	1,345 (20%)	1,714 (21%)
5 – Least deprived	6,305 (28%)	7,445 (26%)	1,618 (25%)	1,827 (22%)
Missing	16 (0.1%)	30 (0.1%)	10 (0.2%)	-
**Diagnosis source, n (%)**				
CPRD	2,869 (13%)	12,890 (46%)	715 (11%)	3,298 (39%)
HES APC	18,049 (81%)	12,248 (43%)	5,443 (82%)	3,873 (46%)
HES OP	112 (0.5%)	658 (2.3%)	25 (0.4%)	144 (1.7%)
ONS	1,252 (5.6%)	2,434 (8.6%)	415 (6.3%)	1,040 (12%)

Table 19. Patient characteristics for men with Lyme disease. Note that cells containing fewer than 10 patients have been suppressed (indicated by '-'). Where this has occurred, the next smallest cell in the group has also been suppressed to prevent re-calculation.

Lyme disease	**Aurum**		**GOLD**	
	**ED**	**Non-ED**	**ED**	**Non-ED**
Men	(N=200)	(N=2,223)	(N=29)	(N=370)
**Age at diagnosis**				
Mean (SD)	50.9 (17.0)	48.1 (16.3)	55.1 (14.9)	51.3 (15.7)
Median (IQR)	52.0 (38.5, 62.0)	48.0 (35.0, 61.0)	57.0 (50.0, 68.0)	52.0 (40.0, 62.0)
**Comorbidity score**				
Mean (SD)	0.8 (1.2)	0.8 (1.2)	0.8 (1.1)	0.7 (1.1)
Median (IQR)	0.0 (0.0, 1.0)	0.0 (0.0, 1.0)	0.0 (0.0, 1.0)	0.0 (0.0, 1.0)
**Deprivation quintile, n (%)**				
1 – Most deprived	51 (26%)	689 (31%)	-	128 (35%)
2	60 (30%)	573 (26%)	-	105 (28%)
3	41 (21%)	487 (22%)	10 (34%)	74 (20%)
4	29 (15%)	303 (14%)	-	46 (12%)
5 – Least deprived	-	171 (7.7%)	-	-
Missing	-			-
**Diagnosis source, n (%)**				
CPRD	65 (33%)	2,186 (98%)	-	357 (96%)
HES APC	135 (68%)	-	23 (79%)	13 (3.5%)
HES OP		-		
ONS			-	

Table 20. Patient characteristics for women with Lyme disease. Note that cells containing fewer than 10 patients have been suppressed (indicated by '-'). Where this has occurred, the next smallest cell in the group has also been suppressed to prevent re-calculation.

Lyme disease	**Aurum**		**GOLD**	
	**ED**	**Non-ED**	**ED**	**Non-ED**
Women	(N=194)	(N=3,062)	(N=32)	(N=432)
**Age at diagnosis**				
Mean (SD)	45.5 (16.5)	46.8 (16.3)	47.0 (21.7)	49.9 (16.0)
Median (IQR)	44.0 (33.0, 57.0)	46.0 (34.0, 59.0)	43.5 (26.0, 66.0)	49.0 (39.0, 62.0)
**Comorbidity score**				
Mean (SD)	0.9 (1.1)	0.9 (1.1)	1.1 (1.3)	0.7 (1.0)
Median (IQR)	1.0 (0.0, 1.0)	1.0 (0.0, 1.0)	1.0 (0.0, 2.0)	0.0 (0.0, 1.0)
**Deprivation quintile, n (%)**				
1 – Most deprived	52 (27%)	861 (28%)	15 (47%)	138 (32%)
2	54 (28%)	753 (25%)	-	119 (28%)
3	31 (16%)	626 (20%)	-	101 (23%)
4	28 (14%)	488 (16%)	-	50 (12%)
5 – Least deprived	29 (15%)	-	-	24 (5.6%)
Missing		-		
**Diagnosis source, n (%)**				
CPRD	49 (25%)	3,006 (98%)	10 (31%)	-
HES APC	145 (75%)	-	22 (69%)	-
HES OP		-		
ONS				

Table 21. Patient characteristics for men with multiple sclerosis. Note that cells containing fewer than 10 patients have been suppressed (indicated by '-'). Where this has occurred, the next smallest cell in the group has also been suppressed to prevent re-calculation.

Multiple sclerosis	Aurum		GOLD	
	**ED**	**Non-ED**	**ED**	**Non-ED**
Men	(N=1,067)	(N=3,657)	(N=327)	(N=1,029)
**Age at diagnosis**				
Mean (SD)	53.7 (19.6)	46.7 (14.1)	52.1 (18.7)	47.7 (14.2)
Median (IQR)	52.0 (38.0, 70.0)	46.0 (36.0, 56.0)	49.0 (37.0, 68.0)	46.0 (38.0, 57.0)
**Comorbidity score**				
Mean (SD)	1.9 (2.2)	0.9 (1.2)	1.4 (1.8)	0.8 (1.2)
Median (IQR)	1.0 (0.0, 3.0)	0.0 (0.0, 1.0)	1.0 (0.0, 2.0)	0.0 (0.0, 1.0)
**Deprivation quintile, n (%)**				
1 – Most deprived	-	801 (22%)	-	249 (24%)
2	183 (17%)	784 (21%)	60 (18%)	270 (26%)
3	228 (21%)	759 (21%)	75 (23%)	226 (22%)
4	222 (21%)	668 (18%)	71 (22%)	157 (15%)
5 – Least deprived	260 (24%)	-	72 (22%)	-
Missing	-	-	-	-
**Diagnosis source, n (%)**				
CPRD	139 (13%)	2,707 (74%)	-	789 (77%)
HES APC	920 (86%)	753 (21%)	276 (84%)	206 (20%)
HES OP	-	174 (4.8%)		-
ONS	-	23 (0.6%)	-	-

Table 22. Patient characteristics for women with multiple sclerosis. Note that cells containing fewer than 10 patients have been suppressed (indicated by '-'). Where this has occurred, the next smallest cell in the group has also been suppressed to prevent re-calculation.

Multiple sclerosis	Aurum		GOLD	
Women	**ED** (N=2,131)	**Non-ED** (N=8,284)	**ED** (N=621)	**Non-ED** (N=2,473)
**Age at diagnosis**				
Mean (SD)	51.4 (19.7)	44.7 (13.3)	51.3 (19.0)	45.3 (13.3)
Median (IQR)	48.0 (36.0, 67.0)	44.0 (35.0, 53.0)	48.0 (37.0, 65.0)	44.0 (36.0, 54.0)
**Comorbidity score**				
Mean (SD)	1.6 (2.0)	0.9 (1.1)	1.4 (1.7)	0.7 (1.1)
Median (IQR)	1.0 (0.0, 2.0)	1.0 (0.0, 1.0)	1.0 (0.0, 2.0)	0.0 (0.0, 1.0)
**Deprivation quintile, n (%)**				
1 – Most deprived	380 (18%)	1,873 (23%)	124 (20%)	592 (24%)
2	397 (19%)	1,771 (21%)	119 (19%)	605 (24%)
3	-	1,635 (20%)	145 (23%	504 (20%)
4	489 (23%)	1,616 (20%)	116 (19%)	435 (18%)
5 – Least deprived	483 (23%)	-	117 (19%)	-
Missing	-	-		-
**Diagnosis source, n (%)**				
CPRD	354 (17%)	6,477 (78%)	105 (17%)	1,907 (77%)
HES APC	1,757 (82%)	1,438 (17%)	514 (83%)	499 (20%)
HES OP	-	337 (4.1%)	-	49 (2.0%)
ONS	-	32 (0.4%)	-	18 (0.7%)

Table 23. Patient characteristics for women with ovarian cancer. Note that cells containing fewer than 10 patients have been suppressed (indicated by '-'). Where this has occurred, the next smallest cell in the group has also been suppressed to prevent re-calculation.

Ovarian cancer	Aurum		GOLD	
	**ED**	**Non-ED**	**ED**	**Non-ED**
Women	(N=7,903)	(N=11,877)	(N=2,602)	(N=3,838)
**Age at diagnosis**				
Mean (SD)	69.3 (14.2)	64.7 (14.0)	69.0 (13.9)	65.2 (13.4)
Median (IQR)	71.0 (60.0, 80.0)	66.0 (55.0, 75.0)	70.0 (60.0, 80.0)	66.0 (56.0, 75.0)
**Comorbidity score**				
Mean (SD)	1.9 (1.8)	1.5 (1.5)	1.6 (1.6)	1.3 (1.4)
Median (IQR)	1.0 (0.0, 3.0)	1.0 (0.0, 2.0)	1.0 (0.0, 2.0)	1.0 (0.0, 2.0)
**Deprivation quintile, n (%)**				
1 – Most deprived	1,609 (20%)	2,783 (23%)	576 (22%)	963 (25%)
2	1,592 (20%)	2,678 (23%)	542 (21%)	892 (23%)
3	1,654 (21%)	2,406 (20%)	556 (21%)	799 (21%)
4	-	2,173 (18%)	504 (19%)	666 (17%)
5 – Least deprived	1,567 (20%)	-	-	518 (13%)
Missing	-	-	-	
**Diagnosis source, n (%)**				
CPRD	1,456 (18%)	4,727 (40%)	415 (16%)	1,376 (36%)
HES APC	5,960 (75%)	6,140 (52%)	2,008 (77%)	2,094 (55%)
HES OP	-	265 (2.2%)	10 (0.4%)	60 (1.6%)
ONS	-	745 (6.3%)	169 (6.5%)	308 (8.0%)

Table 24. Patient characteristics for men with pancreatic cancer. Note that cells containing fewer than 10 patients have been suppressed (indicated by '-'). Where this has occurred, the next smallest cell in the group has also been suppressed to prevent re-calculation.

Pancreatic cancer	**Aurum**		**GOLD**	
	**ED**	**Non-ED**	**ED**	**Non-ED**
Men	(N=7,656)	(N=6,063)	(N=2,204)	(N=1,754)
**Age at diagnosis**				
Mean (SD)	72.3 (11.7)	70.1 (11.6)	72.0 (11.9)	70.6 (11.4)
Median (IQR)	74.0 (65.0, 81.0)	71.0 (63.0, 78.0)	73.0 (64.0, 81.0)	71.0 (63.0, 79.0)
**Comorbidity score**				
Mean (SD)	2.4 (2.0)	2.0 (1.8)	2.0 (1.8)	1.8 (1.7)
Median (IQR)	2.0 (1.0, 4.0)	2.0 (1.0, 3.0)	2.0 (1.0, 3.0)	1.0 (0.0, 3.0)
**Deprivation quintile, n (%)**				
1 – Most deprived	1,558 (20%)	1,438 (24%)	489 (22%)	424 (24%)
2	1,586 (21%)	1,331 (22%)	474 (22%)	423 (24%)
3	1,503 (20%)	1,194 (20%)	472 (21%)	401 (23%)
4	1,539 (20%)	1,114 (18%)	424 (19%)	263 (15%)
5 – Least deprived	-	976 (16%)	-	-
Missing	-	10 (0.2%)	-	-
**Diagnosis source, n (%)**				
CPRD	1,132 (15%)	2,875 (47%)	271 (12%)	697 (40%)
HES APC	5,987 (78%)	2,322 (38%)	1,749 (79%)	719 (41%)
HES OP	25 (0.3%)	101 (1.7%)	-	21 (1.2%)
ONS	512 (6.7%)	765 (13%)	-	317 (18%)

Table 25. Patient characteristics for women with pancreatic cancer. Note that cells containing fewer than 10 patients have been suppressed (indicated by '-'). Where this has occurred, the next smallest cell in the group has also been suppressed to prevent re-calculation.

Pancreatic cancer	**Aurum**		**GOLD**	
	**ED**	**Non-ED**	**ED**	**Non-ED**
Women	(N=7,466)	(N=5,740)	(N=2,301)	(N=1,894)
**Age at diagnosis**				
Mean (SD)	75.7 (12.0)	72.7 (12.4)	75.2 (11.7)	73.5 (11.9)
Median (IQR)	78.0 (68.0, 85.0)	74.0 (65.0, 82.0)	77.0 (68.0, 84.0)	74.0 (66.0, 82.0)
**Comorbidity score**				
Mean (SD)	2.4 (2.0)	2.0 (1.8)	2.0 (1.7)	1.8 (1.6)
Median (IQR)	2.0 (1.0, 4.0)	2.0 (1.0, 3.0)	2.0 (1.0, 3.0)	1.0 (0.0, 3.0)
**Deprivation quintile, n (%)**				
1 – Most deprived	1,541 (21%)	1,339 (23%)	500 (22%)	470 (25%)
2	1,467 (20%)	1,269 (22%)	526 (23%)	471 (25%)
3	-	1,151 (20%)	478 (21%)	408 (22%)
4	1,519 (20%)	1,019 (18%)	439 (19%)	321 (17%)
5 – Least deprived	1,518 (20%)	-	-	-
Missing	-	-	-	-
**Diagnosis source, n (%)**				
CPRD	1,048 (14%)	2,679 (47%)	317 (14%)	739 (39%)
HES APC	5,944 (80%)	2,060 (36%)	1,822 (79%)	707 (37%)
HES OP	19 (0.3%)	93 (1.6%)	-	18.0 (1.0%)
ONS	455 (6.1%)	908 (16%)	-	430 (23%)

Table 26. Patient characteristics for men with Parkinson's disease. Note that cells containing fewer than 10 patients have been suppressed (indicated by '-'). Where this has occurred, the next smallest cell in the group has also been suppressed to prevent re-calculation.

Parkinson's disease	**Aurum**		**GOLD**	
	**ED**	**Non-ED**	**ED**	**Non-ED**
Men	(N=10,710)	(N=22,954)	(N=3,006)	(N=6,995)
**Age at diagnosis**				
Mean (SD)	79.7 (9.2)	73.5 (10.0)	79.6 (9.1)	73.5 (10.0)
Median (IQR)	81.0 (75.0, 86.0)	75.0 (68.0, 81.0)	81.0 (75.0, 86.0)	75.0 (68.0, 81.0)
**Comorbidity score**				
Mean (SD)	3.0 (2.1)	2.1 (1.8)	2.5 (1.9)	1.8 (1.6)
Median (IQR)	3.0 (1.0, 4.0)	2.0 (1.0, 3.0)	2.0 (1.0, 4.0)	1.0 (1.0, 3.0)
**Deprivation quintile, n (%)**				
1 – Most deprived	2,014 (19%)	5,931 (26%)	622 (21%)	1,836 (26%)
2	2,182 (20%)	5,332 (23%)	660 (22%)	1,656 (24%)
3	2,130 (20%)	4,576 (20%)	615 (20%)	1,535 (22%)
4	2,140 (20%)	3,883 (17%)	579 (19%)	1,123 (16%)
5 – Least deprived	2,232 (21%)	3,201 (14%)	-	-
Missing	12 (0.1%)	31 (0.1%)	-	-
**Diagnosis source, n (%)**				
CPRD	588 (5.5%)	19,782 (86%)	161 (5.4%)	6,071 (87%)
HES APC	9,935 (93%)	2,067 (9.0%)	2,788 (93%)	609 (8.7%)
HES OP	-	519 (2.3%)	-	86 (1.2%)
ONS	-	586 (2.6%)	-	229 (3.3%)

Table 27. Patient characteristics for women with Parkinson's disease. Note that cells containing fewer than 10 patients have been suppressed (indicated by '-'). Where this has occurred, the next smallest cell in the group has also been suppressed to prevent re-calculation.

Parkinson's disease	Aurum		GOLD	
	**ED**	**Non-ED**	**ED**	**Non-ED**
Women	(N=8,005)	(N=14,900)	(N=2,376)	(N=4,855)
**Age at diagnosis**				
Mean (SD)	80.6 (9.7)	74.6 (10.3)	81.2 (9.3)	75.1 (10.4)
Median (IQR)	82.0 (76.0, 87.0)	76.0 (69.0, 82.0)	82.0 (77.0, 87.0)	76.0 (69.0, 82.0)
**Comorbidity score**				
Mean (SD)	2.8 (2.1)	2.1 (1.8)	2.3 (1.8)	1.8 (1.6)
Median (IQR)	3.0 (1.0, 4.0)	2.0 (1.0, 3.0)	2.0 (1.0, 3.0)	1.0 (1.0, 3.0)
**Deprivation quintile, n (%)**				
1 – Most deprived	-	3,661 (25%)	493 (21%)	1,228 (25%)
2	1,606 (20%)	3,459 (23%)	472 (20%)	1,101 (23%)
3	1,514 (19%)	2,953 (20%)	516 (22%)	1,061 (22%)
4	1,642 (21%)	2,628 (18%)	498 (21%)	815 (17%)
5 – Least deprived	1,732 (22%)	2,182 (15%)	-	-
Missing	-	17 (0.1%)	-	-
**Diagnosis source, n (%)**				
CPRD	403 (5.0%)	12,655 (85%)	136 (5.7%)	4,099 (84%)
HES APC	7,478 (93%)	1,411 (9.5%)	2,196 (92%)	433 (8.9%)
HES OP	10 (0.1%)	339 (2.3%)	-	72 (1.5%)
ONS	114 (1.4%)	495 (3.3%)	-	251 (5.2%)

Table 28. Patient characteristics for women with PCOS. Note that cells containing fewer than 10 patients have been suppressed (indicated by '-'). Where this has occurred, the next smallest cell in the group has also been suppressed to prevent re-calculation.

PCOS	Aurum		GOLD	
	**ED**	**Non-ED**	**ED**	**Non-ED**
Women	(N=7,126)	(N=81,592)	(N=1,600)	(N=18,855)
**Age at diagnosis**				
Mean (SD)	28.6 (10.0)	27.1 (7.1)	28.2 (9.4)	27.2 (7.4)
Median (IQR)	27.0 (22.0, 33.0)	27.0 (22.0, 32.0)	27.0 (22.0, 33.0)	27.0 (22.0, 32.0)
**Comorbidity score**				
Mean (SD)	0.7 (1.0)	0.4 (0.7)	0.6 (0.8)	0.4 (0.6)
Median (IQR)	0.0 (0.0, 1.0)	0.0 (0.0, 1.0)	0.0 (0.0, 1.0)	0.0 (0.0, 1.0)
**Deprivation quintile, n (%)**				
1 – Most deprived	-	12,784 (16%)	-	3,940 (21%)
2	1,067 (15%)	13,814 (17%)	272 (17%)	3,801 (20%)
3	1,314 (18%)	15,680 (19%)	295 (18%)	4,006 (21%)
4	1,745 (24%)	20,138 (25%)	385 (24%)	3,614 (19%)
5 – Least deprived	2,095 (29%)	19,114 (23%)	400 (25%)	3,474 (18%)
Missing	-	62 (0.1%)	-	20 (0.1%)
**Diagnosis source, n (%)**				
CPRD	689 (9.7%)	71,620 (88%)	151 (9.4%)	16,613 (88%)
HES APC	6,418 (90%)	9,538 (12%)	1,449 (91%)	2,195 (12%)
HES OP	19 (0.3%)	434 (0.5%)		47 (0.2%)
ONS				

Table 29. Patient characteristics for men with rheumatoid arthritis. Note that cells containing fewer than 10 patients have been suppressed (indicated by '-'). Where this has occurred, the next smallest cell in the group has also been suppressed to prevent re-calculation.

Rheumatoid arthritis	Aurum		GOLD	
	**ED**	**Non-ED**	**ED**	**Non-ED**
Men	(N=8,884)	(N=23,789)	(N=2,298)	(N=6,927)
**Age at diagnosis**				
Mean (SD)	69.5 (15.8)	62.3 (14.9)	69.7 (15.4)	61.9 (14.8)
Median (IQR)	72.0 (60.0, 82.0)	64.0 (53.0, 74.0)	72.0 (60.0, 81.0)	63.0 (52.0, 73.0)
**Comorbidity score**				
Mean (SD)	2.9 (2.2)	1.8 (1.8)	2.4 (2.0)	1.5 (1.6)
Median (IQR)	3.0 (1.0, 4.0)	1.0 (0.0, 3.0)	2.0 (1.0, 4.0)	1.0 (0.0, 2.0)
**Deprivation quintile, n (%)**				
1 – Most deprived	-	4,783 (20%)	399 (17%)	1,477 (21%)
2	1,687 (19%)	5,001 (21%)	445 (19%)	1,554 (22%)
3	1,640 (18%)	4,675 (20%)	479 (21%)	1,480 (21%)
4	1,891 (21%)	4,662 (20%)	465 (20%)	1,267 (18%)
5 – Least deprived	2,274 (26%)	4,641 (20%)	510 (22%)	-
Missing	-	27 (0.1%)		-
**Diagnosis source, n (%)**				
CPRD	281 (3.2%)	13,705 (58%)	74 (3.2%)	4,665 (67%)
HES APC	8,550 (96%)	8,589 (36%)	2,216 (96%)	1,914 (28%)
HES OP	32 (0.4%)	1,448 (6.1%)	-	330 (4.8%)
ONS	21 (0.2%)	47 (0.2%)	-	18 (0.3%)

Table 30. Patient characteristics for women with rheumatoid arthritis. Note that cells containing fewer than 10 patients have been suppressed (indicated by '-'). Where this has occurred, the next smallest cell in the group has also been suppressed to prevent re-calculation.

Rheumatoid arthritis	Aurum		GOLD	
Women	**ED** (N=21,183)	**Non-ED** (N=49,443)	**ED** (N=5,745)	**Non-ED** (N=14,774)
**Age at diagnosis**				
Mean (SD)	72.9 (16.8)	60.1 (16.2)	73.7 (16.3)	60.4 (16.1)
Median (IQR)	77.0 (63.0, 86.0)	61.0 (49.0, 73.0)	78.0 (65.0, 86.0)	61.0 (50.0, 73.0)
**Comorbidity score**				
Mean (SD)	2.9 (2.1)	1.8 (1.7)	2.5 (1.9)	1.5 (1.5)
Median (IQR)	3.0 (1.0, 4.0)	1.0 (1.0, 3.0)	2.0 (1.0, 4.0)	1.0 (0.0, 2.0)
**Deprivation quintile, n (%)**				
1 – Most deprived	3,454 (16%)	9,571 (19%)	-	3,057 (21%)
2	3,838 (18%)	9,869 (20%)	1,136 (20%)	3,190 (22%)
3	3,995 (19%)	9,531 (19%)	1,220 (21%)	3,144 (21%)
4	4,601 (22%)	9,871 (20%)	1,149 (20%)	2,821 (19%)
5 – Least deprived	5,275 (25%)	10,561 (21%)	1,294 (23%)	2,550 (17%)
Missing	20 (0.1%)	40 (0.1%)	-	12 (0.1%)
**Diagnosis source, n (%)**				
CPRD	392 (1.9%)	28,198 (57%)	123 (2.1%)	9,480 (64%)
HES APC	20,651 (97%)	18,236 (37%)	5,576 (97%)	4,463 (30%)
HES OP	73 (0.3%)	2,868 (5.8%)	11 (0.2%)	759 (5.1%)
ONS	67 (0.3%)	141 (0.3%)	35 (0.6%)	72 (0.5%)

Table 31. Patient characteristics for men with subacute bacterial endocarditis. Note that cells containing fewer than 10 patients have been suppressed (indicated by '-'). Where this has occurred, the next smallest cell in the group has also been suppressed to prevent re-calculation.

Subacute bacterial endocarditis	**Aurum**		**GOLD**	
Men	**ED**<br>(N=4,274)	**Non-ED**<br>(N=899)	**ED**<br>(N=1,092)	**Non-ED**<br>(N=237)
**Age at diagnosis**				
Mean (SD)	63.6 (17.4)	62.9 (17.3)	63.9 (16.6)	64.1 (17.0)
Median (IQR)	67.0 (51.0, 77.0)	66.0 (51.0, 77.0)	67.0 (53.0, 77.0)	68.0 (52.0, 78.0)
**Comorbidity score**				
Mean (SD)	2.7 (2.3)	2.6 (2.3)	2.4 (2.2)	2.4 (2.1)
Median (IQR)	2.0 (1.0, 4.0)	2.0 (1.0, 4.0)	2.0 (1.0, 4.0)	2.0 (1.0, 4.0)
**Deprivation quintile, n (%)**				
1 – Most deprived	833 (19%)	180 (20%)	245 (22%)	58 (24%)
2	-	185 (21%)	224 (21%)	55 (23%)
3	834 (20%)	168 (19%)	243 (22%)	43 (18%)
4	856 (20%)	180 (20%)	180 (16%)	45 (19%)
5 – Least deprived	932 (22%)	186 (21%)	200 (18%)	36 (15%)
Missing	-			
**Diagnosis source, n (%)**				
CPRD	539 (13%)	433 (48%)	121 (11%)	112 (47%)
HES APC	3,564 (83%)	300 (33%)	921 (84%)	88 (37%)
HES OP	-			
ONS	-	166 (18%)	50 (4.6%)	37 (16%)

Table 32. Patient characteristics for women with subacute bacterial endocarditis. Note that cells containing fewer than 10 patients have been suppressed (indicated by '-'). Where this has occurred, the next smallest cell in the group has also been suppressed to prevent re-calculation.

Subacute bacterial endocarditis	**Aurum**		**GOLD**	
	**ED**	**Non-ED**	**ED**	**Non-ED**
Women	(N=2,121)	(N=480)	(N=588)	(N=142)
**Age at diagnosis**				
Mean (SD)	67.1 (18.4)	65.2 (18.5)	67.1 (18.6)	65.4 (18.8)
Median (IQR)	72.0 (56.0, 81.0)	70.0 (53.0, 79.0)	72.0 (55.0, 82.0)	70.0 (53.0, 79.0)
**Comorbidity score**				
Mean (SD)	3.3 (2.5)	2.9 (2.2)	2.7 (2.2)	2.7 (2.3)
Median (IQR)	3.0 (1.0, 5.0)	3.0 (1.0, 4.0)	2.0 (1.0, 4.0)	2.0 (1.0, 4.0)
**Deprivation quintile, n (%)**				
1 – Most deprived	401 (19%)	76 (16%)	102 (17%)	17 (12%)
2	-	96 (20%)	131 (22%)	44 (31%)
3	397 (19%)	97 (20%)	119 (20%)	38 (27%)
4	432 (20%)	92 (19%)	125 (21%)	20 (14%)
5 – Least deprived	499 (24%)	119 (25%)	111 (19%)	23 (16%)
Missing	-			
**Diagnosis source, n (%)**				
CPRD	270 (13%)	223 (46%)	48 (8.2%)	61 (43%)
HES APC	1,719 (81%)	159 (33%)	486 (83%)	41 (29%)
HES OP		-		
ONS	132 (6.2%)	-	54 (9.2%)	40 (28%)

Table 33. Patient characteristics for men with schizophrenia. Note that cells containing fewer than 10 patients have been suppressed (indicated by '-'). Where this has occurred, the next smallest cell in the group has also been suppressed to prevent re-calculation.

Schizophrenia	Aurum		GOLD	
	**ED**	**Non-ED**	**ED**	**Non-ED**
Men	(N=13,269)	(N=18,215)	(N=3,177)	(N=4,380)
**Age at diagnosis**				
Mean (SD)	45.8 (20.8)	40.6 (18.5)	45.9 (20.5)	40.7 (18.9)
Median (IQR)	41.0 (28.0, 61.0)	37.0 (26.0, 51.0)	42.0 (29.0, 62.0)	36.0 (25.0, 52.0)
**Comorbidity score**				
Mean (SD)	1.2 (1.6)	0.8 (1.3)	0.9 (1.4)	0.7 (1.1)
Median (IQR)	1.0 (0.0, 2.0)	0.0 (0.0, 1.0)	0.0 (0.0, 1.0)	0.0 (0.0, 1.0)
**Deprivation quintile, n (%)**				
1 – Most deprived	1,327 (10%)	1,835 (10%)	-	-
2	1,671 (13%)	2,266 (12%)	471 (15%)	687 (16%)
3	2,150 (16%)	2,967 (16%)	580 (18%)	817 (19%)
4	3,224 (24%)	4,657 (26%)	786 (25%)	1,016 (23%)
5 – Least deprived	4,876 (37%)	6,468 (36%)	988 (31%)	1,313 (30%)
Missing	21 (0.2%)	22 (0.1%)	-	-
**Diagnosis source, n (%)**				
CPRD	2,069 (16%)	14,821 (81%)	493 (16%)	3,610 (82%)
HES APC	11,146 (84%)	2,307 (13%)	2,667 (84%)	551 (13%)
HES OP	42 (0.3%)	1,043 (5.7%)	-	199 (4.5%)
ONS	12 (0.1%)	44 (0.2%)	-	20 (0.5%)

Table 34. Patient characteristics for women with schizophrenia. Note that cells containing fewer than 10 patients have been suppressed (indicated by '-'). Where this has occurred, the next smallest cell in the group has also been suppressed to prevent re-calculation.

Schizophrenia	**Aurum**		**GOLD**	
	**ED**	**Non-ED**	**ED**	**Non-ED**
Women	(N=12,455)	(N=16,178)	(N=3,149)	(N=4,013)
**Age at diagnosis**				
Mean (SD)	56.2 (23.2)	52.8 (22.5)	58.0 (22.9)	55.9 (22.9)
Median (IQR)	55.0 (36.0, 78.0)	50.0 (34.0, 73.0)	59.0 (38.0, 79.0)	54.0 (37.0, 78.0)
**Comorbidity score**				
Mean (SD)	1.7 (1.9)	1.4 (1.7)	1.4 (1.7)	1.1 (1.4)
Median (IQR)	1.0 (0.0, 3.0)	1.0 (0.0, 2.0)	1.0 (0.0, 2.0)	1.0 (0.0, 2.0)
**Deprivation quintile, n (%)**				
1 – Most deprived	1,584 (13%)	2,130 (13%)	-	-
2	1,798 (14%)	2,412 (15%)	532 (17%)	768 (19%)
3	2,232 (18%)	2,849 (18%)	610 (19%)	795 (20%)
4	2,951 (24%)	3,950 (24%)	715 (23%)	875 (22%)
5 – Least deprived	3,876 (31%)	4,817 (30%)	806 (26%)	943 (23%)
Missing	14 (0.1%)	20 (0.1%)	-	-
**Diagnosis source, n (%)**				
CPRD	1,924 (15%)	13,174 (81%)	483 (15%)	3,249 (81%)
HES APC	10,454 (84%)	2,136 (13%)	2,656 (84%)	533 (13%)
HES OP	66 (0.5%)	825 (5.1%)	-	218 (5.4%)
ONS	11 (0.1%)	43 (0.3%)	-	13 (0.3%)

Table 35. Patient characteristics for men with tuberculosis. Note that cells containing fewer than 10 patients have been suppressed (indicated by '-'). Where this has occurred, the next smallest cell in the group has also been suppressed to prevent re-calculation.

Tuberculosis	Aurum		GOLD	
	**ED**	**Non-ED**	**ED**	**Non-ED**
Men	(N=3,624)	(N=6,352)	(N=779)	(N=1,238)
**Age at diagnosis**				
Mean (SD)	48.9 (20.6)	46.8 (18.4)	52.9 (21.0)	51.1 (19.5)
Median (IQR)	45.5 (31.0, 66.0)	44.0 (32.0, 61.0)	52.0 (34.0, 72.0)	50.0 (34.0 ,68.0)
**Comorbidity score**				
Mean (SD)	1.3 (1.8)	1.1 (1.5)	1.3 (1.7)	1.1 (1.5)
Median (IQR)	0.0 (0.0, 2.0)	0.0 (0.0, 2.0)	1.0 (0.0, 2.0)	0.0 (0.0, 2.0)
**Deprivation quintile, n (%)**				
1 – Most deprived	-	-	-	179 (14%)
2	368 (10%)	790 (12%)	109 (14%)	199 (16%)
3	590 (16%)	1,069 (17%)	170 (22%)	244 (20%)
4	1,113 (31%)	1,909 (30%)	160 (21%)	281 (23%)
5 – Least deprived	1,281 (35%)	2,020 (32%)	258 (33%)	335 (27%)
Missing	-	-	-	
**Diagnosis source, n (%)**				
CPRD	700 (19%)	5,403 (85%)	150 (19%)	1,012 (82%)
HES APC	2,770 (76%)	688 (11%)	591 (76%)	157 (13%)
HES OP	13 (0.4%)	139 (2.2%)	-	35 (2.8%)
ONS	141 (3.9%)	122 (1.9%)	-	34 (2.7%)

Table 36. Patient characteristics for men with tuberculosis. Note that cells containing fewer than 10 patients have been suppressed (indicated by '-'). Where this has occurred, the next smallest cell in the group has also been suppressed to prevent re-calculation.

Tuberculosis	Aurum		GOLD	
	**ED**	**Non-ED**	**ED**	**Non-ED**
Women	(N=2,250)	(N=5,645)	(N=512)	(N=1,255)
**Age at diagnosis**				
Mean (SD)	48.6 (21.7)	45.9 (18.8)	53.4 (22.4)	49.2 (19.0)
Median (IQR)	45.0 (29.0, 69.0)	42.0 (31.0, 60.0)	53.0 (32.0, 73.0)	47.0 (33.0, 65.0)
**Comorbidity score**				
Mean (SD)	1.4 (1.8)	1.1 (1.5)	1.4 (1.7)	1.1 (1.4)
Median (IQR)	1.0 (0.0, 2.0)	1.0 (0.0, 2.0)	1.0 (0.0, 2.0)	1.0 (0.0, 2.0)
**Deprivation quintile, n (%)**				
1 – Most deprived	-	-	-	-
2	226 (10%)	703 (12%)	84 (16%)	229 (18%)
3	387 (17%)	1,019 (18%)	103 (20%)	256 (20%)
4	703 (31%)	1,636 (29%)	101 (20%)	306 (24%)
5 – Least deprived	748 (33%)	1,700 (30%)	151 (29%)	289 (23%)
Missing	-	-	-	-
**Diagnosis source, n (%)**				
CPRD	488 (22%)	4,903 (87%)	89 (17%)	1,071 (85%)
HES APC	1,676 (74%)	504 (8.9%)	394 (77%)	119 (9.5%)
HES OP	12 (0.5%)	154 (2.7%)	-	53 (4.2%)
ONS	74 (3.3%)	84 (1.5%)	-	12 (1.0%)

Main results – GOLD

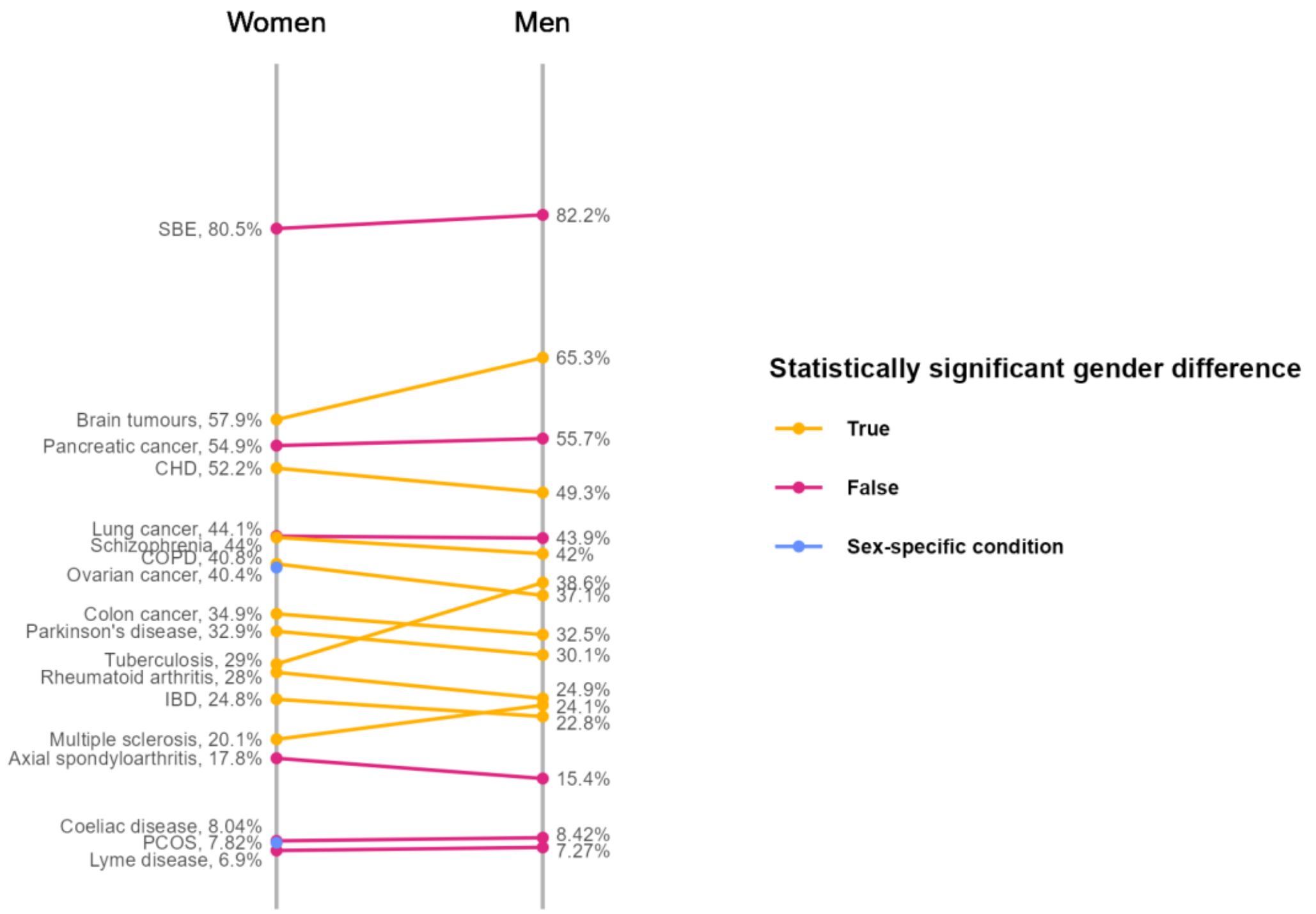

Figure 19. Slope plot of the frequency of emergency diagnosis by condition and gender – GOLD. CHD, coronary/ischaemic heart disease; COPD, chronic obstructive pulmonary disease; IBD, inflammatory bowel disease; PCOS, polycystic ovary syndrome; SBE, subacute bacterial endocarditis.

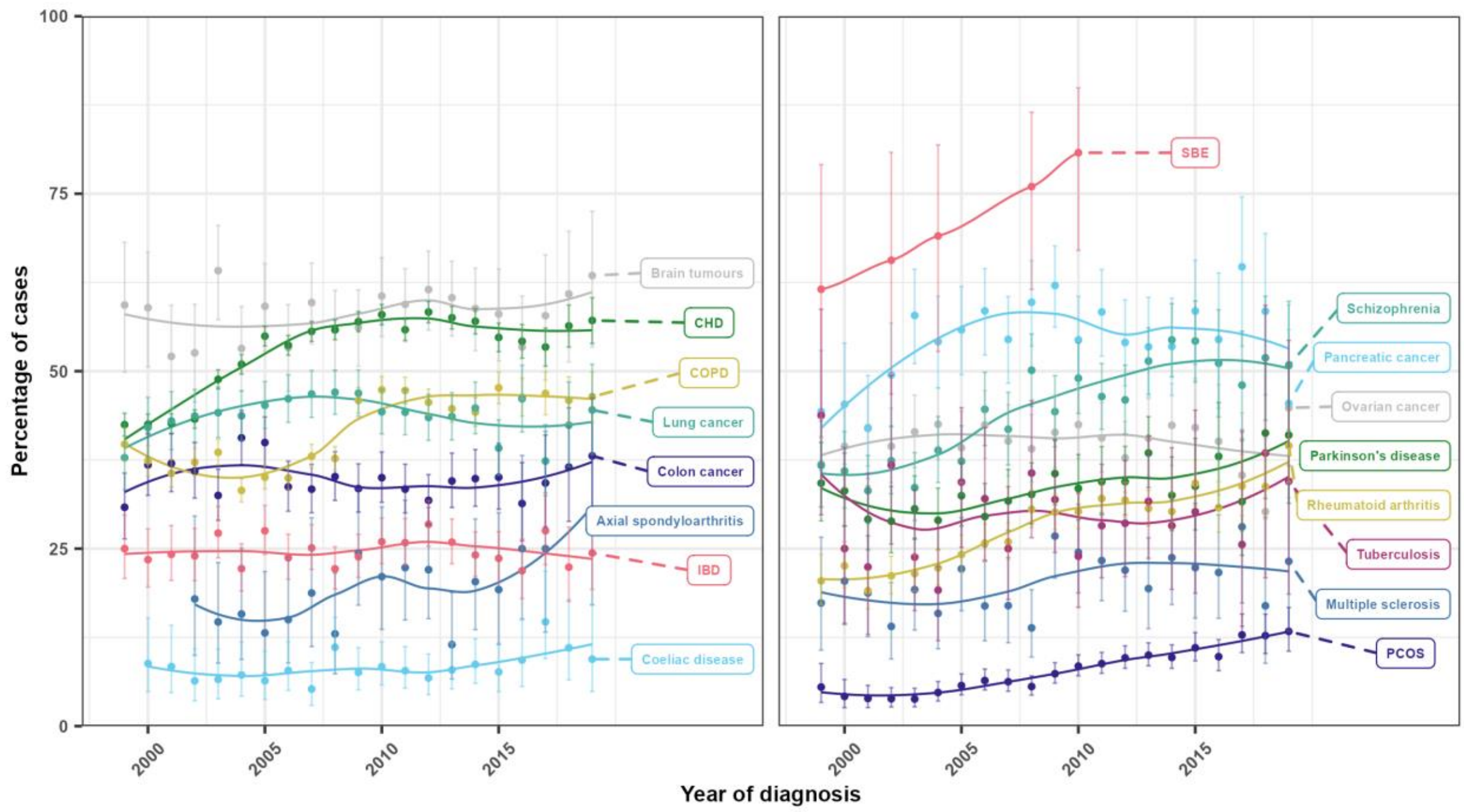

Figure 20. Percentage of cases diagnosed following an emergency presentation (emergency hospital admission) by year of diagnosis – GOLD, Women. Note that only years in which at least 10 cases were diagnosed as an emergency and 10 cases not diagnosed as an emergency are shown. Lines are loess curves indicative of general trends over time. Conditions are split alphabetically across the two panels for visual clarity. CHD, coronary/ischaemic heart disease; COPD, chronic obstructive pulmonary disease; IBD, inflammatory bowel disease; PCOS, polycystic ovary syndrome; SBE, subacute bacterial endocarditis.

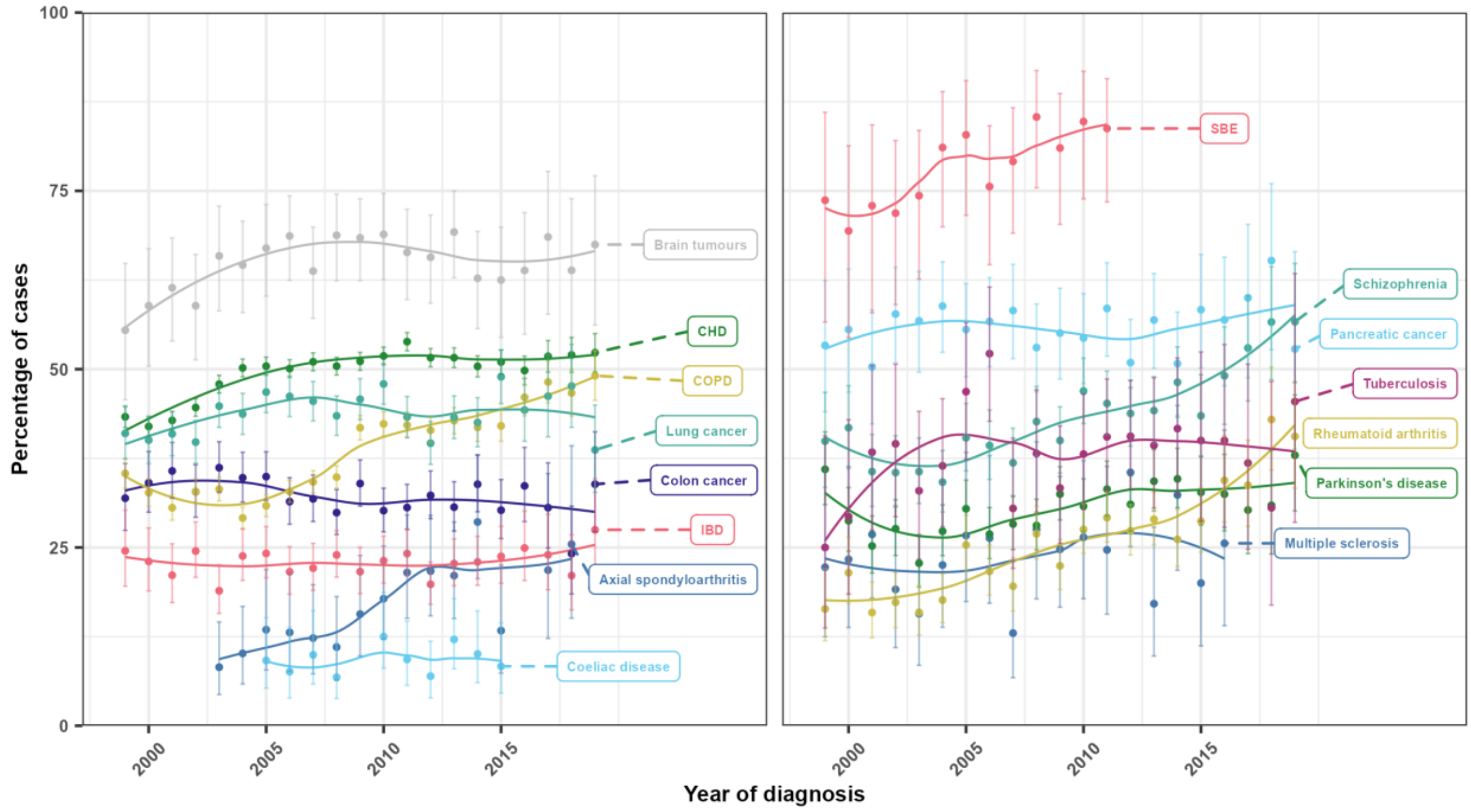

Figure 21. Percentage of cases diagnosed following an emergency presentation (emergency hospital admission) by year of diagnosis – GOLD, Men. Note that only years in which at least 10 cases were diagnosed as an emergency and 10 cases not diagnosed as an emergency are shown. Lines are loess curves indicative of general trends over time. Conditions are split alphabetically across the two panels for visual clarity. CHD, coronary/ischaemic heart disease; COPD, chronic obstructive pulmonary disease; IBD, inflammatory bowel disease; SBE, subacute bacterial endocarditis.

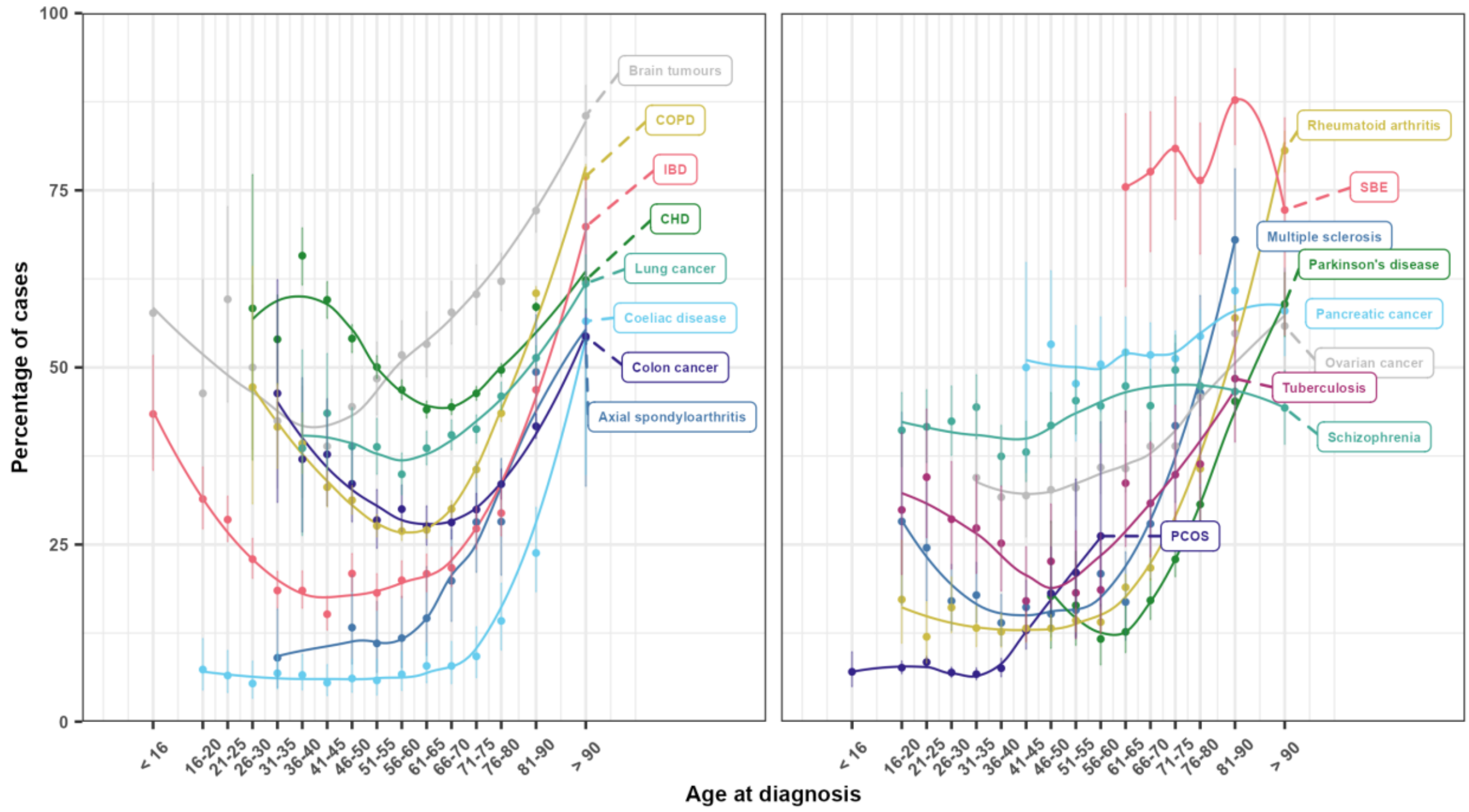

Figure 22. Percentage of cases diagnosed following an emergency presentation (emergency hospital admission) by age at diagnosis - GOLD, Women. Note that only age groups with at least 10 cases diagnosed as an emergency and 10 cases not diagnosed as an emergency are shown. Lines are loess curves indicative of general patterns over age groups. Conditions are split alphabetically across the two panels for visual clarity. CHD, coronary/ischaemic heart disease; COPD, chronic obstructive pulmonary disease; IBD, inflammatory bowel disease; PCOS, polycystic ovary syndrome; SBE, subacute bacterial endocarditis.

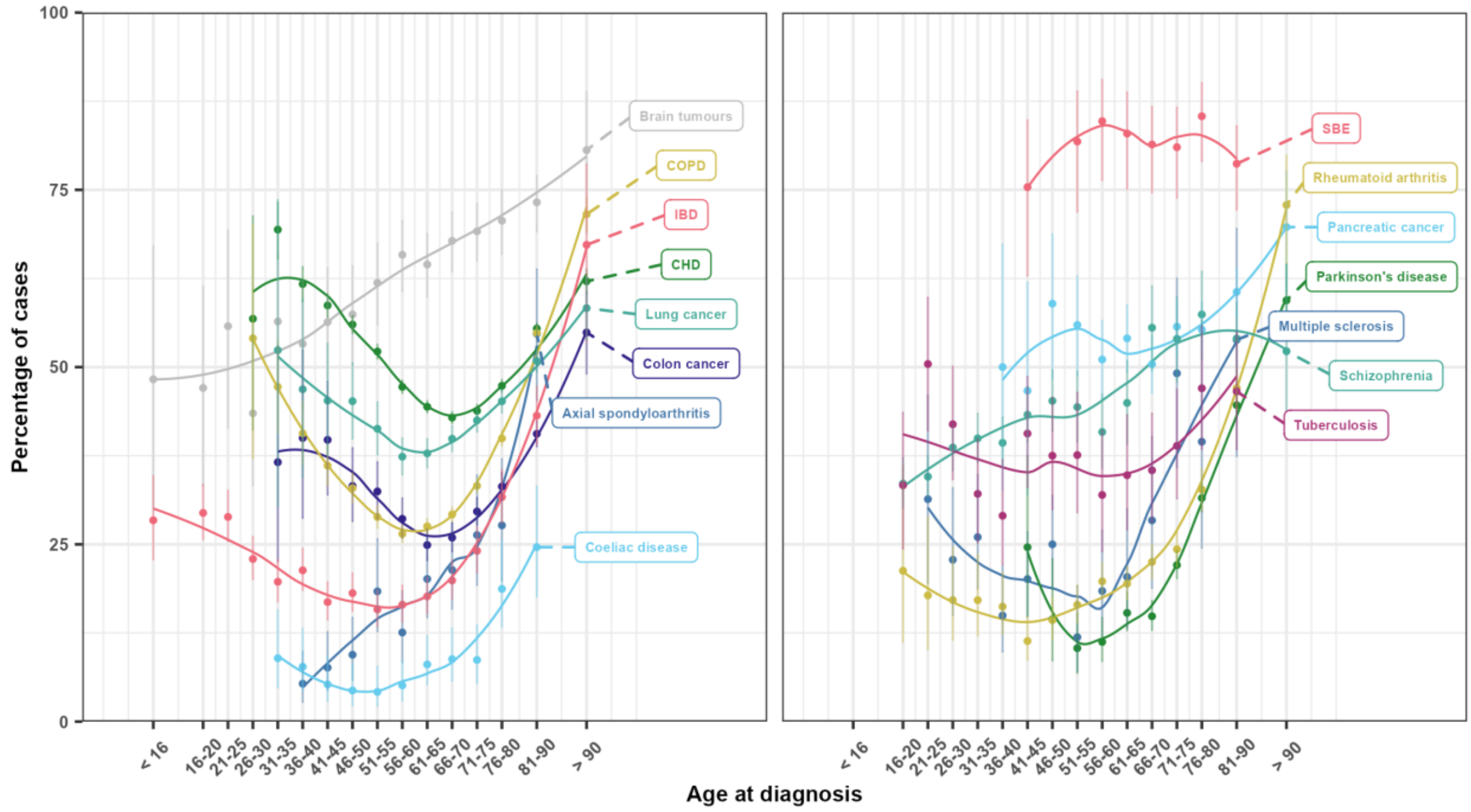

Figure 23. Percentage of cases diagnosed following an emergency presentation (emergency hospital admission) by age at diagnosis - GOLD, Men. Note that only age groups with at least 10 cases diagnosed as an emergency and 10 cases not diagnosed as an emergency are shown. Lines are loess curves indicative of general patterns over age groups. Conditions are split alphabetically across the two panels for visual clarity. CHD, coronary/ischaemic heart disease; COPD, chronic obstructive pulmonary disease; IBD, inflammatory bowel disease; SBE, subacute bacterial endocarditis.

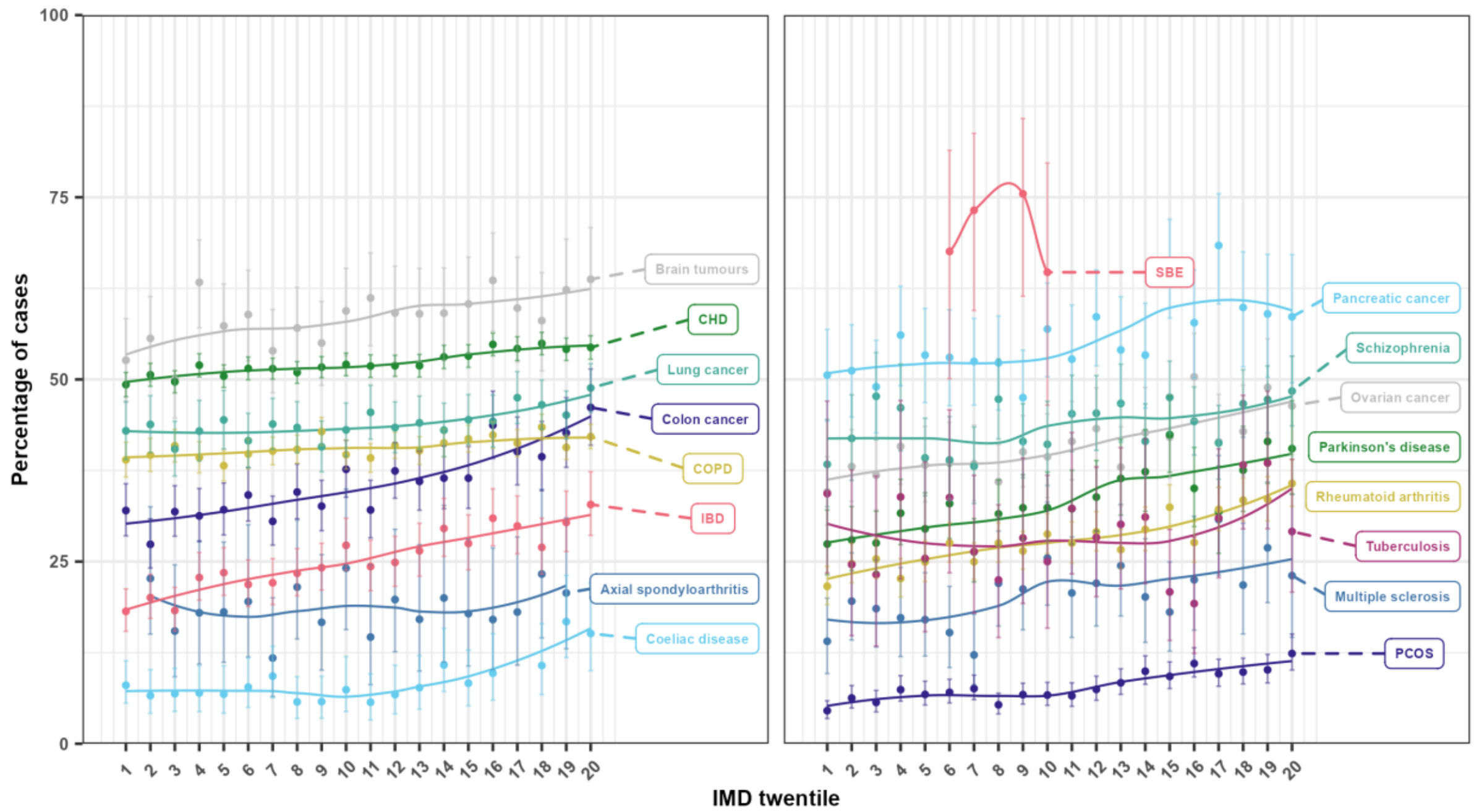

Figure 24. Percentage of cases diagnosed following an emergency presentation (emergency hospital admission) by IMD twentile - GOLD, Women. Note that only twentiles with at least 10 cases diagnosed as an emergency and 10 cases not diagnosed as an emergency are shown. Lines are loess curves indicative of general patterns over twentiles. Note that 1 is least deprived, 20 is most deprived. Conditions are split alphabetically across the two panels for visual clarity. CHD, coronary/ischaemic heart disease; COPD, chronic obstructive pulmonary disease; IBD, inflammatory bowel disease; PCOS, polycystic ovary syndrome; SBE, subacute bacterial endocarditis.

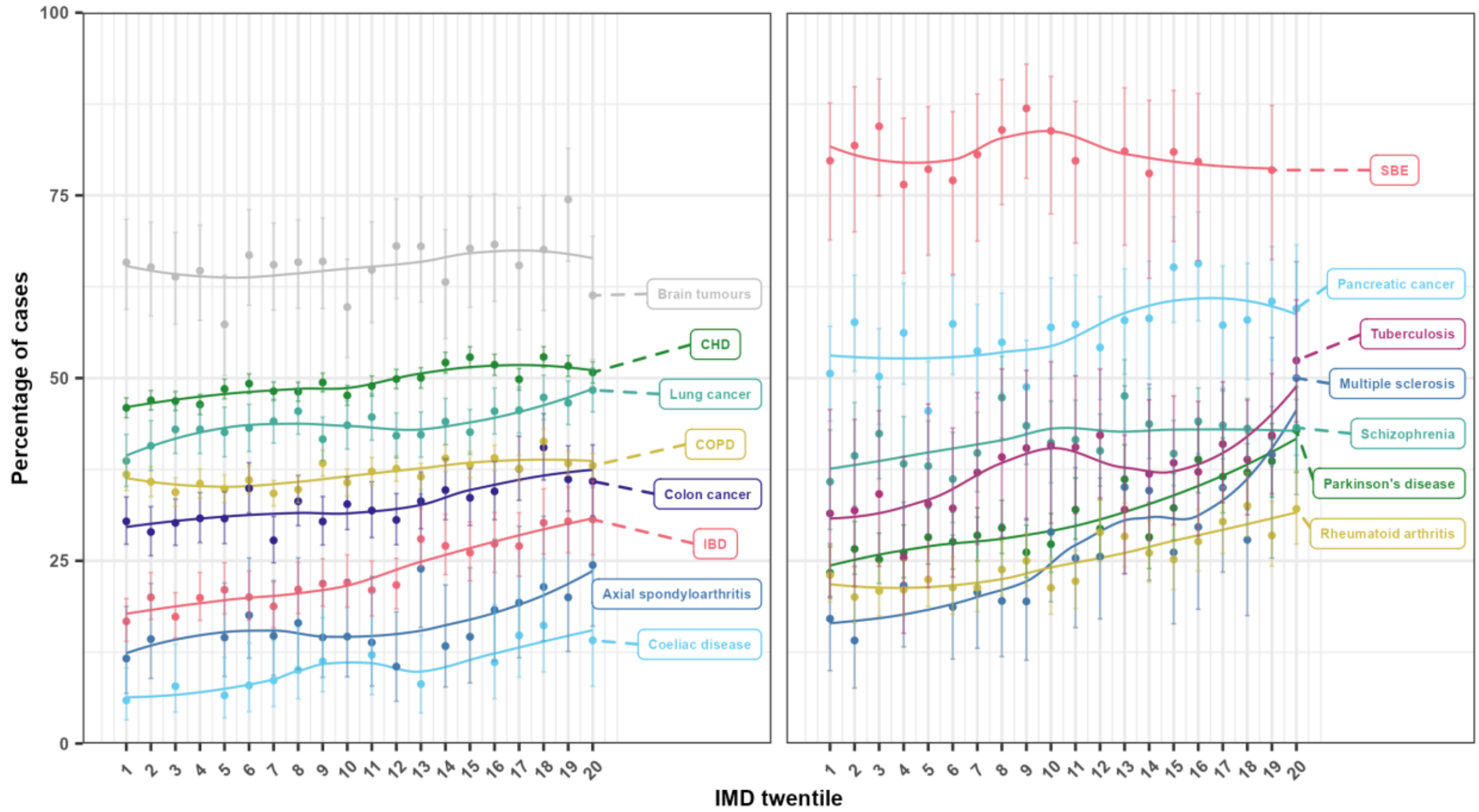

Figure 25. Percentage of cases diagnosed following an emergency presentation (emergency hospital admission) by IMD twentile - GOLD, Men. Note that only twentiles with at least 10 cases diagnosed as an emergency and 10 cases not diagnosed as an emergency are shown. Lines are loess curves indicative of general patterns over twentiles. Note that 1 is least deprived, 20 is most deprived. Conditions are split alphabetically across the two panels for visual clarity. CHD, coronary/ischaemic heart disease; COPD, chronic obstructive pulmonary disease; IBD, inflammatory bowel disease; SBE, subacute bacterial endocarditis.

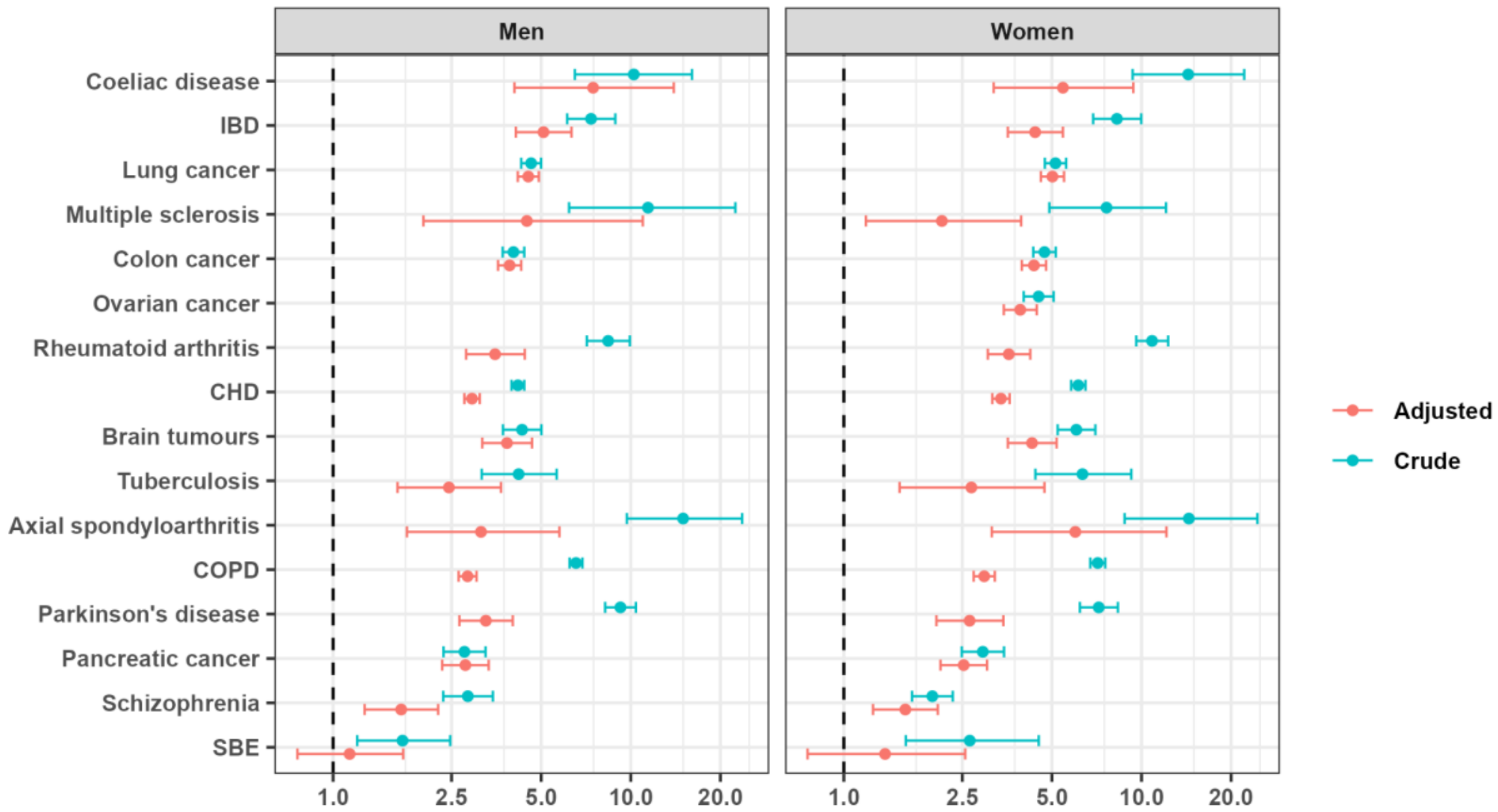

Figure 26. Forest plot of odds ratios for 1-year mortality following an emergency diagnosis – GOLD. Note that only gender-condition combinations with at least 10 emergency diagnosed patients who died in the year after diagnosis were examined – consequently Lyme disease is not included. Odds ratios were adjusted for age at diagnosis, year of diagnosis, IMD score, comorbidity score six months (180 days) prior to diagnosis and diagnosis source. CHD, coronary/ischaemic heart disease; COPD, chronic obstructive pulmonary disease; IBD, inflammatory bowel disease; SBE, subacute bacterial endocarditis.

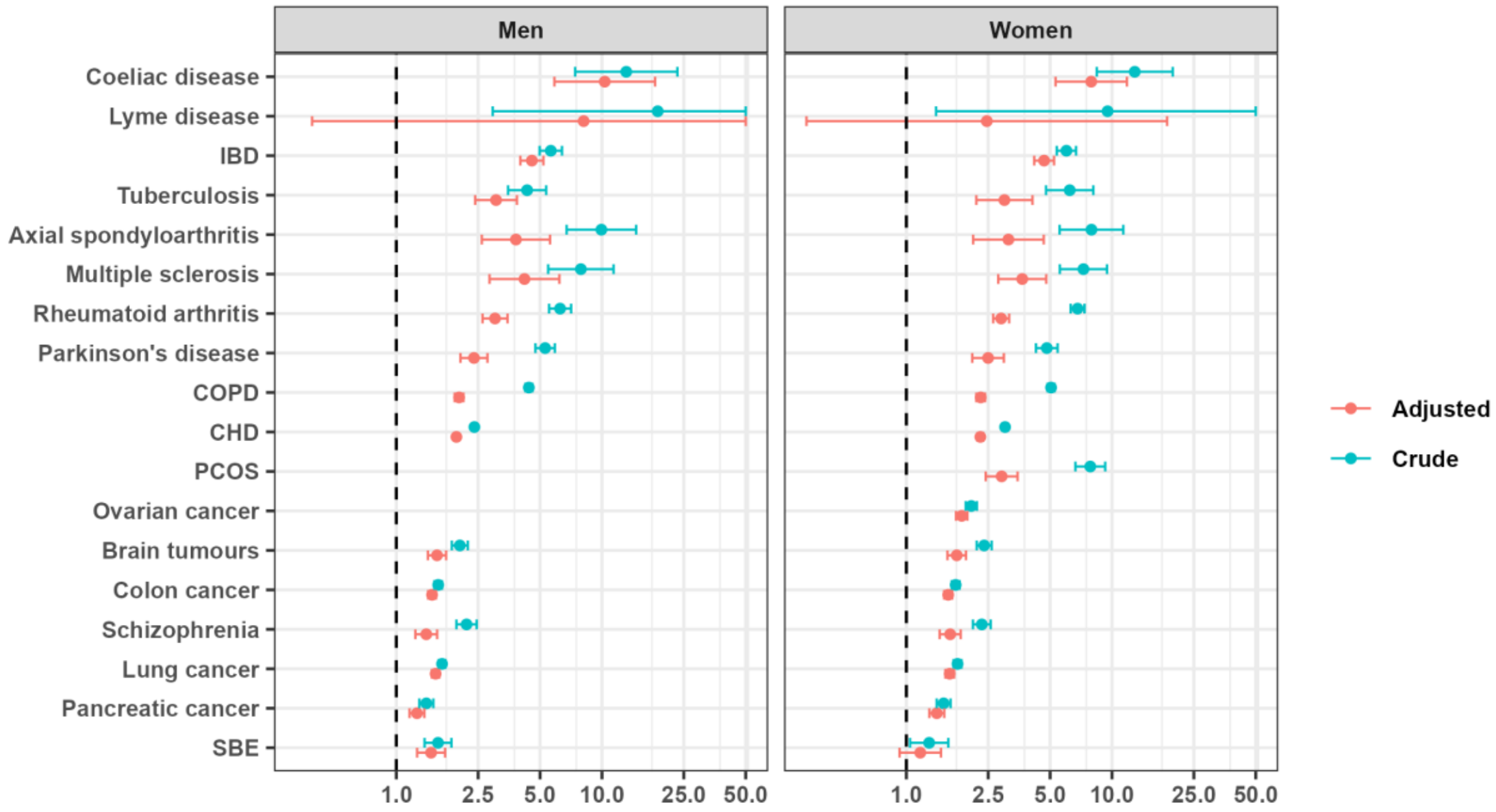

Figure 27. Forest plot of rate ratios for time in hospital in the year post-diagnosis following an emergency diagnosis – GOLD. N.B. Upper confidence intervals that exceed 50 have been reduced to 50 for visualisation purposes. Rate ratios were adjusted for age at diagnosis, year of diagnosis, IMD score, comorbidity score six months (180 days) prior to diagnosis and diagnosis source. CHD, coronary/ischaemic heart disease; COPD, chronic obstructive pulmonary disease; IBD, inflammatory bowel disease; PCOS, polycystic ovary syndrome; SBE, subacute bacterial endocarditis.

Table 37. Overview of results - GOLD

Condition	Gender	N	% emergency diagnosed	Adjusted [a] OR (95% CI) for 1-year mortality	Adjusted [a] RR (95% CI) for time in hospital
Axial spondyloarthritis	Men	2,133	15% (14%, 17%)	3.14 (1.77, 5.76)	3.82 (2.61, 5.59)
	Women	1,745	18% (16%, 20%)	5.99 (3.14, 12.1)	3.14 (2.11, 4.66)
Brain tumours	Men	3,901	65% (64%, 67%)	3.84 (3.17, 4.66)	1.58 (1.42, 1.75)
	Women	5,046	58% (57%, 59%)	4.29 (3.56, 5.18)	1.76 (1.58, 1.95)
Coeliac disease	Men	2,626	8.4% (7.4%, 9.6%)	7.47 (4.06, 14.0)	10.3 (5.87, 18.1)
	Women	4,976	8.0% (7.3%, 8.8%)	5.45 (3.19, 9.38)	7.92 (5.31, 11.8)
CHD	Men	104,083	49% (49%, 50%)	2.93 (2.76, 3.11)	1.96 (1.91, 2.01)
	Women	81,739	52% (52%, 53%)	3.37 (3.15, 3.60)	2.29 (2.22, 2.36)
Colon cancer	Men	12,919	32% (32%, 33%)	3.92 (3.58, 4.28)	1.49 (1.43, 1.56)
	Women	11,729	35% (34%, 36%)	4.35 (3.96, 4.78)	1.60 (1.53, 1.67)
COPD	Men	55,207	37% (37%, 38%)	2.83 (2.64, 3.03)	2.02 (1.92, 2.12)
	Women	50,630	41% (40%, 41%)	2.96 (2.73, 3.21)	2.30 (2.19, 2.41)
IBD	Men	10,580	23% (22%, 24%)	5.09 (4.11, 6.32)	4.56 (4.01, 5.19)
	Women	12,343	25% (24%, 26%)	4.39 (3.56, 5.44)	4.67 (4.18, 5.22)
Lung cancer	Men	19,418	44% (43%, 45%)	4.53 (4.17, 4.91)	1.55 (1.49, 1.62)
	Women	14,953	44% (43%, 45%)	5.02 (4.59, 5.49)	1.62 (1.55, 1.71)
Lyme disease	Men	399	7.3% (5.0%, 10%)	-	8.14 (0.390, 170)
	Women	464	6.9% (4.8%, 9.7%)	-	2.46 (0.327, 18.5)
Multiple sclerosis	Men	1,356	24% (22%, 27%)	4.48 (2.01, 11.0)	4.20 (2.84, 6.21)
	Women	3,094	20% (19%, 22%)	2.14 (1.19, 3.94)	3.66 (2.80, 4.79)
Ovarian cancer	Women	6,440	40% (39%, 42%)	3.91 (3.45, 4.45)	1.86 (1.74, 1.98)
Pancreatic cancer	Men	3,958	56% (54%, 57%)	2.78 (2.33, 3.33)	1.26 (1.16, 1.37)
	Women	4,195	55% (53%, 56%)	2.53 (2.11, 3.03)	1.41 (1.29, 1.53)
Parkinson's disease	Men	10,001	30% (29%, 31%)	3.26 (2.66, 4.01)	2.39 (2.05, 2.77)
	Women	7,231	33% (32%, 34%)	2.64 (2.05, 3.44)	2.50 (2.09, 2.98)
PCOS	Women	20,455	7.8% (7.5%, 8.2%)	-	2.91 (2.43, 3.47)
Rheumatoid arthritis	Men	9,225	25% (24%, 26%)	3.50 (2.80, 4.41)	3.02 (2.63, 3.47)
	Women	20,519	28% (27%, 29%)	3.58 (3.05, 4.23)	2.89 (2.64, 3.16)
SBE	Men	1,329	82% (80%, 84%)	1.14 (0.759, 1.72)	1.48 (1.26, 1.73)
	Women	730	81% (77%, 83%)	1.38 (0.755, 2.56)	1.17 (0.927, 1.47)
Schizophrenia	Men	7,557	42% (41%, 43%)	1.69 (1.28, 2.25)	1.40 (1.24, 1.58)
	Women	7,162	44% (43%, 45%)	1.61 (1.25, 2.07)	1.63 (1.45, 1.84)
Tuberculosis	Men	2,017	39% (36%, 41%)	2.45 (1.65, 3.67)	3.06 (2.42, 3.86)
	Women	1,767	29% (27%, 31%)	2.68 (1.54, 4.72)	3.00 (2.19, 4.10)

[a] Adjusted for age at diagnosis, year of diagnosis, IMD score, comorbidity score six months (180 days) prior to diagnosis and diagnosis source.
"–" indicates that fewer than 10 patients died within 1 year of diagnosis. Odds ratios were not calculated for these conditions as the results are unlikely to be reliable.

Table 38. Observed 1-year mortality and odds ratios (ORs) for 1-year mortality in emergency diagnosed vs non-emergency diagnosed patients by condition and gender - GOLD.

Condition	Gender	Emergency diagnosed		Non-emergency diagnosed		Crude OR (95% CI)	Adjusted [b] OR (95% CI)	p-value [c]
		N patients [a]	1-year mortality, % (n)	N patients [a]	1-year mortality, % (n)			
Axial spondyloarthritis	Men	325	21% (68)	1,800	1.7% (31)	15.0 (9.70, 23.7)	3.14 (1.77, 5.76)	0.154
	Women	305	18% (56)	1,435	1.5% (22)	14.4 (8.78, 24.5)	5.99 (3.14, 12.1)	
Brain tumours	Men	2,490	61% (1,525)	1,255	27% (336)	4.31 (3.72, 5.01)	3.84 (3.17, 4.66)	0.420
	Women	2,860	50% (1,428)	1,990	14% (282)	6.04 (5.23, 7.00)	4.29 (3.56, 5.18)	
Coeliac disease	Men	220	17% (38)	2,400	2.0% (48)	10.2 (6.49, 16.1)	7.47 (4.06, 14.0)	0.448
	Women	400	12% (47)	4,570	0.92% (42)	14.4 (9.34, 22.1)	5.45 (3.19, 9.38)	
CHD	Men	47,795	20% (9,436)	38,225	5.6% (2,127)	4.18 (3.98, 4.39)	2.93 (2.76, 3.11)	< 0.01
	Women	38,970	27% (10,694)	27,485	5.8% (1,597)	6.13 (5.80, 6.48)	3.37 (3.15, 3.60)	
Colon cancer	Men	3,845	50% (1,919)	8,165	20% (1,617)	4.03 (3.71, 4.39)	3.92 (3.58, 4.28)	0.112
	Women	3,830	53% (2,040)	6,955	19% (1,351)	4.72 (4.33, 5.15)	4.35 (3.96, 4.78)	
COPD	Men	19,795	35% (6,988)	33,015	7.7% (2,540)	6.55 (6.23, 6.88)	2.83 (2.64, 3.03)	0.404
	Women	20,080	31% (6,134)	28,800	5.8% (1,674)	7.13 (6.73, 7.55)	2.96 (2.73, 3.21)	
IBD	Men	2,395	14% (344)	8,160	2.2% (182)	7.35 (6.11, 8.87)	5.09 (4.11, 6.32)	0.338
	Women	3,040	13% (399)	9,255	1.8% (166)	8.27 (6.88, 9.98)	4.39 (3.56, 5.44)	
Lung cancer	Men	7,800	87% (6,753)	9,585	58% (5,579)	4.63 (4.29, 5.00)	4.53 (4.17, 4.91)	0.0931
	Women	6,155	84% (5,175)	7,290	51% (3,699)	5.14 (4.74, 5.58)	5.02 (4.59, 5.49)	
Lyme disease	Men	30	-	370	-	-	-	-
	Women	30	-	430	-	-	-	
Multiple sclerosis	Men	320	13% (41)	1,020	1.3% (13)	11.4 (6.2, 22.5)	4.48 (2.01, 11.0)	0.153
	Women	620	8.9% (55)	2,450	1.3% (31)	7.63 (4.9, 12.1)	2.14 (1.19, 3.94)	
Ovarian cancer	Women	2,420	52% (1,247)	3,525	19% (673)	4.51 (4.02, 5.07)	3.91 (3.45, 4.45)	
Pancreatic cancer	Men	2,015	84% (1,699)	1,430	66% (946)	2.76 (2.35, 3.25)	2.78 (2.33, 3.33)	0.459
	Women	2,140	86% (1,835)	1,455	67% (982)	2.93 (2.49, 3.45)	2.53 (2.11, 3.03)	
Parkinson's disease	Men	2,935	41% (1,205)	6,760	7.0% (474)	9.23 (8.20, 10.4)	3.26 (2.66, 4.01)	0.217
	Women	2,325	33% (762)	4,600	6.3% (292)	7.18 (6.21, 8.33)	2.64 (2.05, 3.44)	
PCOS	Women	1,600	0.75% (12)	18,835	-	-	-	
Rheumatoid arthritis	Men	2,290	22% (500)	6,900	3.2% (222)	8.39 (7.12, 9.93)	3.50 (2.80, 4.41)	0.871
	Women	5,690	21% (1,205)	14,690	2.4% (355)	10.8 (9.60, 12.3)	3.58 (3.05, 4.23)	
SBE	Men	1,040	33% (340)	200	22% (44)	1.71 (1.20, 2.47)	1.14 (0.759, 1.72)	0.608
	Women	530	42% (221)	100	21% (21)	2.65 (1.62, 4.52)	1.38 (0.755, 2.56)	
Schizophrenia	Men	3,165	10% (325)	4,355	3.9% (169)	2.84 (2.34, 3.44)	1.69 (1.28, 2.25)	0.792
	Women	3,140	13% (422)	3,995	7.3% (290)	1.98 (1.69, 2.32)	1.61 (1.25, 2.07)	
Tuberculosis	Men	750	22% (165)	1,205	6.3% (76)	4.20 (3.16, 5.64)	2.45 (1.65, 3.67)	0.796
	Women	485	20% (95)	1,240	3.7% (46)	6.33 (4.40, 9.24)	2.68 (1.54, 4.72)	

[a] To prevent backwards calculation of small patient groups the number of patients has been reported rounded to 5.
[b] Adjusted for age at diagnosis, year of diagnosis, IMD score, comorbidity score six months (180 days) prior to diagnosis and diagnosis source.
[c] Note that p-values relate to the null hypothesis that there was no difference in the adjusted OR between men and women tested using likelihood ratio tests.

"–" indicates that fewer than 10 patients died within 1 year of diagnosis. Odds ratios were not calculated for these conditions as the results are unlikely to be reliable.
CI, confidence interval; OR, odds ratio; CHD, coronary/ischaemic heart disease; COPD, chronic obstructive pulmonary disease; IBD, inflammatory bowel disease; PCOS, polycystic ovary syndrome; SBE, subacute bacterial endocarditis.

Table 39. Observed mean number of hospital inpatient nights in year after diagnosis and rate ratios (RRs) for duration of hospital inpatient nights in year after diagnosis in emergency diagnosed vs non-emergency diagnosed patients by condition and gender - GOLD.

Condition	Gender	Emergency diagnosed			Non-emergency diagnosed			Crude RR (95% CI)	Adjusted [c] RR (95% CI)	p-value [d]
		N patients [a]	Admitted, % (n) [b]	Mean admitted duration	N patients [a]	Admitted, % (n) [b]	Mean admitted duration			
Axial spondyloarthritis	Men	325	85% (277)	21.1	1,800	16% (289)	2.1	9.94 (6.73, 14.7)	3.82 (2.61, 5.59)	0.495
	Women	305	85% (258)	22.9	1,435	23% (331)	2.9	7.94 (5.56, 11.3)	3.14 (2.11, 4.66)	
Brain tumours	Men	2,490	94.6% (2,355)	28.6	1,255	64% (807)	14.0	2.04 (1.86, 2.23)	1.58 (1.42, 1.75)	0.148
	Women	2,860	93.9% (2,686)	29.5	1,990	58% (1,151)	12.3	2.39 (2.20, 2.60)	1.76 (1.58, 1.95)	
Coeliac disease	Men	220	79% (174)	21.2	2,400	13% (309)	1.6	13.1 (7.41, 23.3)	10.3 (5.87, 18.1)	0.450
	Women	400	72% (289)	16.4	4,570	12% (571)	1.3	12.9 (8.43, 19.7)	7.92 (5.31, 11.8)	
CHD	Men	47,795	87% (41,781)	14.3	38,225	47% (17,790)	5.9	2.40 (2.34, 2.45)	1.96 (1.91, 2.01)	< 0.01
	Women	38,970	86% (33,550)	18.4	27,485	40% (10,902)	6.1	3.02 (2.94, 3.10)	2.29 (2.22, 2.36)	
Colon cancer	Men	3,845	96.2% (3,698)	24.5	8,165	86% (7,008)	15.3	1.60 (1.53, 1.67)	1.49 (1.43, 1.56)	0.0320
	Women	3,830	95.6% (3,661)	26.0	6,955	85% (5,900)	15.0	1.74 (1.66, 1.81)	1.60 (1.53, 1.67)	
COPD	Men	19,795	87% (17,318)	20.7	33,015	30% (9,936)	4.7	4.41 (4.25, 4.58)	2.02 (1.92, 2.12)	< 0.01
	Women	20,080	88% (17,666)	21.4	28,800	28% (7,930)	4.2	5.05 (4.86, 5.25)	2.30 (2.19, 2.41)	
IBD	Men	2,395	85% (2,039)	19.7	8,160	23% (1,870)	3.5	5.64 (4.98, 6.39)	4.56 (4.01, 5.19)	0.778
	Women	3,040	84% (2,552)	19.5	9,255	24% (2,184)	3.2	6.00 (5.39, 6.69)	4.67 (4.18, 5.22)	
Lung cancer	Men	7,800	92.3% (7,198)	19.2	9,585	74% (7,058)	11.5	1.67 (1.61, 1.73)	1.55 (1.49, 1.62)	0.161
	Women	6,155	93.0% (5,718)	20.3	7,290	72% (5,252)	11.5	1.77 (1.70, 1.85)	1.62 (1.55, 1.71)	
Lyme disease	Men	30	57% (17)	16.4	370	9.6% (35)	0.9	18.7 (2.94, 118)	8.14 (0.390, 170)	0.548
	Women	30	67% (20)	8.8	430	7.7% (33)	0.9	9.52 (1.39, 64.9)	2.46 (0.327, 18.5)	
Multiple sclerosis	Men	320	79% (252)	22.0	1,020	20% (205)	2.8	7.90 (5.48, 11.4)	4.20 (2.84, 6.21)	0.574
	Women	620	77% (479)	18.9	2,450	21% (515)	2.6	7.26 (5.58, 9.46)	3.66 (2.80, 4.79)	
Ovarian cancer	Women	2,420	95.2% (2,304)	23.2	3,525	79% (2,791)	11.2	2.07 (1.94, 2.20)	1.86 (1.74, 1.98)	
Pancreatic cancer	Men	2,015	93.9% (1,892)	20.3	1,430	77% (1,108)	14.5	1.40 (1.29, 1.51)	1.26 (1.16, 1.37)	0.0697
	Women	2,140	95.3% (2,040)	21.8	1,455	74% (1,075)	14.4	1.52 (1.40, 1.64)	1.41 (1.29, 1.53)	
Parkinson's disease	Men	2,935	92.4% (2,713)	36.2	6,760	26% (1,745)	6.8	5.29 (4.75, 5.90)	2.39 (2.05, 2.77)	0.703
	Women	2,325	90.7% (2,109)	35.3	4,600	26% (1,210)	7.3	4.82 (4.27, 5.44)	2.50 (2.09, 2.98)	
PCOS	Women	1,600	63% (1,002)	5.2	18,835	15% (2,879)	0.7	7.84 (6.63, 9.26)	2.91 (2.43, 3.47)	
Rheumatoid arthritis	Men	2,290	82% (1,875)	20.1	6,900	25% (1,747)	3.2	6.26 (5.53, 7.08)	3.02 (2.63, 3.47)	0.600
	Women	5,690	83% (4,727)	22.0	14,690	25% (3,612)	3.2	6.80 (6.29, 7.35)	2.89 (2.64, 3.16)	
SBE	Men	1,040	97.2% (1,011)	45.0	200	74% (147)	28.2	1.59 (1.37, 1.85)	1.48 (1.26, 1.73)	0.0978
	Women	530	98.5% (522)	45.4	100	72% (72)	35.2	1.29 (1.04, 1.60)	1.17 (0.927, 1.47)	

Condition	Gender	Emergency diagnosed			Non-emergency diagnosed			Crude RR (95% CI)	Adjusted [c] RR (95% CI)	p-value [d]
		N patients [a]	Admitted, % (n) [b]	Mean admitted duration	N patients [a]	Admitted, % (n) [b]	Mean admitted duration			
Schizophrenia	Men	3,165	83% (2,620)	45.9	4,355	33% (1,435)	20.9	2.20 (1.96, 2.46)	1.40 (1.24, 1.58)	0.0728
	Women	3,140	89% (2,785)	47.9	3,995	41% (1,628)	20.6	2.33 (2.11, 2.57)	1.63 (1.45, 1.84)	
Tuberculosis	Men	750	86% (646)	25.1	1,205	26% (318)	5.8	4.33 (3.50, 5.35)	3.06 (2.42, 3.86)	0.918
	Women	485	86% (418)	25.0	1,240	25% (314)	4.0	6.23 (4.78, 8.11)	3.00 (2.19, 4.10)	

[a] To prevent backwards calculation of small patient groups the number of patients has been reported rounded to 5.

[b] Percentage (number) of patients admitted for at least one night in the year after diagnosis

[c] Adjusted for age at diagnosis, year of diagnosis, IMD score, comorbidity score six months (180 days) prior to diagnosis and diagnosis source.

[d] Note that p-values relate to the null hypothesis that there was no difference in the adjusted RR between men and women tested using likelihood ratio tests.

CI, confidence interval; RR, rate ratio; CHD, coronary/ischaemic heart disease; COPD, chronic obstructive pulmonary disease; IBD, inflammatory bowel disease; PCOS, polycystic ovary syndrome; SBE, subacute bacterial endocarditis.

Main results – dataset comparison

Table 40. Percentage (95% confidence interval) of cases diagnosed within 30 days of an emergency hospital admission by condition, gender, and CPRD dataset.

Condition	Aurum			GOLD		
	Men	Women	p-value	Men	Women	p-value
Axial spondyloarthritis	18% (18%, 19%)	23% (22%, 24%)	< 0.01	15% (14%, 17%)	18% (16%, 20%)	0.0502
Brain tumours	65% (64%, 65%)	57% (56%, 57%)	< 0.01	65% (64%, 67%)	58% (57%, 59%)	< 0.01
Coeliac disease	8.9% (8.3%, 9.5%)	9.1% (8.7%, 9.5%)	0.699	8.4% (7.4%, 9.6%)	8.0% (7.3%, 8.8%)	0.598
CHD	50% (50%, 50%)	54% (54%, 54%)	< 0.01	49% (49%, 50%)	52% (52%, 53%)	< 0.01
Colon cancer	32% (32%, 33%)	36% (35%, 36%)	< 0.01	32% (32%, 33%)	35% (34%, 36%)	< 0.01
COPD	36% (35%, 36%)	40% (39%, 40%)	< 0.01	37% (37%, 38%)	41% (40%, 41%)	< 0.01
IBD	24% (23%, 24%)	26% (26%, 26%)	< 0.01	23% (22%, 24%)	25% (24%, 26%)	< 0.01
Lung cancer	44% (44%, 45%)	44% (44%, 45%)	0.974	44% (43%, 45%)	44% (43%, 45%)	0.682
Lyme disease	8.3% (7.2%, 9.4%)	6.0% (5.2%, 6.8%)	< 0.01	7.3% (5.0%, 10%)	6.9% (4.8%, 9.7%)	0.937
MS	23% (21%, 24%)	20% (20%, 21%)	< 0.01	24% (22%, 27%)	20% (19%, 22%)	< 0.01
Ovarian cancer		40% (39%, 41%)			40% (39%, 42%)	
Pancreatic cancer	56% (55%, 57%)	57% (56%, 57%)	0.233	56% (54%, 57%)	55% (53%, 56%)	0.463
Parkinson’s disease	32% (31%, 32%)	35% (34%, 36%)	< 0.01	30% (29%, 31%)	33% (32%, 34%)	< 0.01
PCOS		8.0% (7.9%, 8.2%)			7.8% (7.5%, 8.2%)	
Rheumatoid arthritis	27% (27%, 28%)	30% (30%, 30%)	< 0.01	25% (24%, 26%)	28% (27%, 29%)	< 0.01
SBE	83% (82%, 84%)	82% (80%, 83%)	0.254	82% (80%, 84%)	81% (77%, 83%)	0.397
Schizophrenia	42% (42%, 43%)	43% (43%, 44%)	< 0.01	42% (41%, 43%)	44% (43%, 45%)	0.0190
TB	36% (35%, 37%)	28% (28%, 30%)	< 0.01	39% (36%, 41%)	29% (27%, 31%)	< 0.01

Note that p-values relate to the null hypothesis that there was no difference in the percentage of cases diagnosed within 30 days of an emergency hospital admission between men and women tested using Chi-squared tests with Yates’ continuity correction. CHD, coronary/ischaemic heart disease; COPD, chronic obstructive pulmonary disease; IBD, inflammatory bowel disease; PCOS, polycystic ovary syndrome; SBE, subacute bacterial endocarditis.

Table 41. Mortality within 1 year of emergency diagnosis by condition, gender, and CPRD dataset.

Condition	Gender	Aurum		GOLD	
		1-year mortality amongst ED patients	Adjusted [a] OR (95% CI)	1-year mortality amongst ED patients	Adjusted [a] OR (95% CI)
Axial spondyloarthritis	Men	20%	3.80 (2.82, 5.18)	21%	3.14 (1.77, 5.76)
	Women	14%	3.73 (2.64, 5.36)	18%	5.99 (3.14, 12.1)
Brain tumours	Men	56%	3.24 (2.93, 3.59)	61%	3.84 (3.17, 4.66)
	Women	45%	4.00 (3.62, 4.42)	50%	4.29 (3.56, 5.18)
Coeliac disease	Men	18%	7.16 (5.28, 9.76)	17%	7.47 (4.06, 14.0)
	Women	11%	7.53 (5.64, 10.1)	12%	5.45 (3.19, 9.38)
CHD	Men	19%	3.39 (3.28, 3.50)	20%	2.93 (2.76, 3.11)
	Women	26%	4.03 (3.88, 4.18)	27%	3.37 (3.15, 3.60)
Colon cancer	Men	51%	4.44 (4.23, 4.67)	50%	3.92 (3.58, 4.28)
	Women	53%	4.79 (4.54, 5.06)	53%	4.35 (3.96, 4.78)
COPD	Men	33%	2.96 (2.85, 3.07)	35%	2.83 (2.64, 3.03)
	Women	29%	3.55 (3.39, 3.71)	31%	2.96 (2.73, 3.21)
IBD	Men	13%	5.99 (5.30, 6.78)	14%	5.09 (4.11, 6.32)
	Women	13%	5.24 (4.65, 5.92)	13%	4.39 (3.56, 5.44)
Lung cancer	Men	85%	4.77 (4.57, 4.98)	87%	4.53 (4.17, 4.91)
	Women	81%	5.02 (4.79, 5.26)	84%	5.02 (4.59, 5.49)
Lyme disease	Men	-		-	
	Women	-		-	
Multiple sclerosis	Men	15%	5.32 (3.37, 8.69)	13%	4.48 (2.01, 11.0)
	Women	11%	4.92 (3.36, 7.36)	8.9%	2.14 (1.19, 3.94)
Ovarian cancer	Women	53%	4.44 (4.13, 4.78)	52%	3.91 (3.45, 4.45)
Pancreatic cancer	Men	83%	2.64 (2.40, 2.90)	84%	2.78 (2.33, 3.33)
	Women	85%	2.75 (2.49, 3.04)	86%	2.53 (2.11, 3.03)
Parkinson's disease	Men	40%	2.91 (2.60, 3.25)	41%	3.26 (2.66, 4.01)
	Women	30%	2.77 (2.38, 3.22)	33%	2.64 (2.05, 3.44)
PCOS	Women	0.51%	2.78 (1.48, 5.35)	0.75%	-
Rheumatoid arthritis	Men	20%	3.63 (3.25, 4.06)	22%	3.50 (2.80, 4.41)
	Women	20%	4.09 (3.74, 4.48)	21%	3.58 (3.05, 4.23)
SBE	Men	31%	1.04 (0.837, 1.29)	33%	1.14 (0.759, 1.72)
	Women	39%	1.19 (0.891, 1.59)	42%	1.38 (0.755, 2.56)
Schizophrenia	Men	10%	1.79 (1.54, 2.09)	10%	1.69 (1.28, 2.25)
	Women	13%	1.66 (1.44, 1.92)	13%	1.61 (1.25, 2.07)
Tuberculosis	Men	17%	3.04 (2.45, 3.76)	22%	2.45 (1.65, 3.67)
	Women	13%	3.84 (2.83, 5.20)	20%	2.68 (1.54, 4.72)

[a] Adjusted for age at diagnosis, year of diagnosis, IMD score, comorbidity score six months (180 days) prior to diagnosis and diagnosis source.
"–" indicates that fewer than 10 patients died within 1 year of diagnosis. Odds ratios were not calculated for these conditions as the results are unlikely to be reliable
ED, emergency diagnosed; CI, confidence interval; OR, odds ratio; CHD, coronary/ischaemic heart disease; COPD, chronic obstructive pulmonary disease; IBD, inflammatory bowel disease; PCOS, polycystic ovary syndrome; SBE, subacute bacterial endocarditis.

Table 42. Hospitalisation within 1 year of emergency diagnosis by condition, gender, and CPRD dataset.

Condition	Gender	Aurum			GOLD		
		**Admitted, % (n) [a]**	**Mean admitted duration**	**Adjusted [a] RR (95% CI)**	**Admitted, % (n) [a]**	**Mean admitted duration**	**Adjusted [b] RR (95% CI)**
Axial spondyloarthritis	Men	78%	21.9	2.84 (2.27, 3.55)	85%	21.1	3.82 (2.61, 5.59)
	Women	76%	17.8	3.67 (3.10, 4.34)	85%	22.9	3.14 (2.11, 4.66)
Brain tumours	Men	93.6%	27.3	1.59 (1.50, 1.68)	94.6%	28.6	1.58 (1.42, 1.75)
	Women	91.2%	27.2	1.85 (1.75, 1.95)	93.9%	29.5	1.76 (1.58, 1.95)
Coeliac disease	Men	72%	18.8	8.76 (6.52, 11.8)	79%	21.2	10.3 (5.87, 18.1)
	Women	69%	13.6	6.90 (5.70, 8.34)	72%	16.4	7.92 (5.31, 11.8)
CHD	Men	87%	14.3	2.07 (2.04, 2.10)	87%	14.3	1.96 (1.91, 2.01)
	Women	85%	18.4	2.50 (2.45, 2.54)	86%	18.4	2.29 (2.22, 2.36)
Colon cancer	Men	94.7%	24.2	1.57 (1.54, 1.61)	96.2%	24.5	1.49 (1.43, 1.56)
	Women	95.2%	25.4	1.69 (1.65, 1.74)	95.6%	26.0	1.60 (1.53, 1.67)
COPD	Men	86%	20.4	2.18 (2.13, 2.24)	87%	20.7	2.02 (1.92, 2.12)
	Women	87%	21.1	2.54 (2.47, 2.60)	88%	21.4	2.30 (2.19, 2.41)
IBD	Men	82%	17.9	5.12 (4.78, 5.48)	85%	19.7	4.56 (4.01, 5.19)
	Women	83%	18.9	4.81 (4.54, 5.10)	84%	19.5	4.67 (4.18, 5.22)
Lung cancer	Men	92.2%	19.3	1.57 (1.53, 1.60)	92.3%	19.2	1.55 (1.49, 1.62)
	Women	92.6%	20.1	1.68 (1.64, 1.73)	93.0%	20.3	1.62 (1.55, 1.71)
Lyme disease	Men	52%	8.1	4.49 (1.72, 11.7)	57%	16.4	8.14 (0.390, 170)
	Women	52%	6.9	5.59 (2.57, 12.2)	67%	8.8	2.46 (0.327, 18.5)
Multiple sclerosis	Men	76%	19.0	3.63 (2.88, 4.57)	79%	22.0	4.20 (2.84, 6.21)
	Women	74%	16.7	3.11 (2.65, 3.66)	77%	18.9	3.66 (2.80, 4.79)
Ovarian cancer	Women	94.6%	22.3	1.91 (1.84, 1.98)	95.2%	23.2	1.86 (1.74, 1.98)
Pancreatic cancer	Men	93.5%	19.7	1.28 (1.22, 1.34)	93.9%	20.3	1.26 (1.16, 1.37)
	Women	93.8%	20.9	1.38 (1.32, 1.45)	95.3%	21.8	1.41 (1.29, 1.53)
Parkinson's disease	Men	91.5%	35.9	2.46 (2.27, 2.67)	92.4%	36.2	2.39 (2.05, 2.77)
	Women	90.6%	34.2	2.77 (2.51, 3.05)	90.7%	35.3	2.50 (2.09, 2.98)
PCOS	Women	59%	4.5	2.34 (2.16, 2.54)	63%	5.2	2.91 (2.43, 3.47)
Rheumatoid arthritis	Men	80%	18.2	3.19 (2.99, 3.41)	82%	20.1	3.02 (2.63, 3.47)
	Women	81%	20.3	3.03 (2.90, 3.17)	83%	22.0	2.89 (2.64, 3.16)
SBE	Men	98.2%	45.6	1.16 (1.07, 1.25)	97.2%	45.0	1.48 (1.26, 1.73)
	Women	97.0%	47.5	1.21 (1.08, 1.36)	98.5%	45.4	1.17 (0.927, 1.47)
Schizophrenia	Men	82%	40.6	1.32 (1.24, 1.40)	83%	45.9	1.40 (1.24, 1.58)
	Women	87%	44.2	1.69 (1.59, 1.79)	89%	47.9	1.63 (1.45, 1.84)
Tuberculosis	Men	85%	22.6	3.05 (2.72, 3.42)	86%	25.1	3.06 (2.42, 3.86)
	Women	85%	22.2	4.13 (3.55, 4.80)	86%	25.0	3.00 (2.19, 4.10)

[a] Percentage of patients diagnosed as an emergency admitted for at least one night in the year after diagnosis.

[b] Adjusted for age at diagnosis, year of diagnosis, IMD score, comorbidity score six months (180 days) prior to diagnosis and diagnosis source.

Condition	Gender	Aurum			GOLD		
		Admitted, % (n) [a]	Mean admitted duration	Adjusted [a] RR (95% CI)	Admitted, % (n) [a]	Mean admitted duration	Adjusted [b] RR (95% CI)

"–" indicates that fewer than 10 patients died within 1 year of diagnosis. Odds ratios were not calculated for these conditions as the results are unlikely to be reliable
CI, confidence interval; RR, rate ratio; CHD, coronary/ischaemic heart disease; COPD, chronic obstructive pulmonary disease; IBD, inflammatory bowel disease; PCOS, polycystic ovary syndrome; SBE, subacute bacterial endocarditis.

Sensitivity analysis 1: A&E attendance

Table 43. Percentage (95% confidence interval) of patients diagnosed as an emergency stratified by gender and emergency diagnosis definition - GOLD

Condition	*Men*		*Women*		p-value
	**Hospital admission (1999 – 2019)**	**Hospital admission or A&E attendance (2007 – 2019)**	**Hospital admission (1999 – 2019)**	**Hospital admission or A&E attendance (2007 – 2019)**	
Axial spondyloarthritis	15% (14%, 17%)	21% (19%, 23%)	18% (16%, 20%)	22% (20%, 25%)	0.399
Brain tumours	65% (64%, 67%)	69% (67%, 71%)	58% (57%, 59%)	62% (60%, 63%)	< 0.01
Coeliac disease	8.4% (7.4%, 9.6%)	10% (9.0%, 12%)	8.0% (7.3%, 8.8%)	11% (9.9%, 12%)	0.611
<u>CHD</u>	49% (49%, 50%)	57% (56%, 57%)	52% (52%, 53%)	61% (60%, 61%)	< 0.01
<u>Colon cancer</u>	32% (32%, 33%)	33% (32%, 34%)	35% (34%, 36%)	36% (35%, 37%)	< 0.01
<u>COPD</u>	37% (37%, 38%)	44% (43%, 44%)	41% (40%, 41%)	46% (46%, 47%)	< 0.01
IBD	23% (22%, 24%)	26% (25%, 27%)	25% (24%, 26%)	27% (26%, 28%)	0.262
Lung cancer	44% (43%, 45%)	47% (46%, 48%)	44% (43%, 45%)	48% (47%, 49%)	0.746
Lyme disease	7.3% (5.0%, 10%)	14% (10%, 18%)	6.9% (4.8%, 9.7%)	13% (9.4%, 17%)	0.791
Multiple sclerosis	24% (22%, 27%)	27% (24%, 30%)	20% (19%, 22%)	24% (22%, 26%)	0.165
Ovarian cancer			40% (39%, 42%)	42% (41%, 44%)	
Pancreatic cancer	56% (54%, 57%)	58% (56%, 60%)	55% (53%, 56%)	59% (57%, 61%)	0.475
<u>Parkinson's disease</u>	30% (29%, 31%)	33% (32%, 35%)	33% (32%, 34%)	36% (35%, 38%)	< 0.01
PCOS			7.8% (7.5%, 8.2%)	12% (12%, 13%)	
<u>Rheumatoid arthritis</u>	25% (24%, 26%)	30% (29%, 32%)	28% (27%, 29%)	33% (32%, 34%)	< 0.01
SBE	82% (80%, 84%)	88% (86%, 90%)	81% (77%, 83%)	86% (82%, 89%)	0.234
Schizophrenia	42% (41%, 43%)	51% (50%, 53%)	44% (43%, 45%)	54% (53%, 56%)	0.0146
Tuberculosis	39% (36%, 41%)	43% (41%, 46%)	29% (27%, 31%)	33% (31%, 36%)	< 0.01

Note that p-values relate to the null hypothesis that there was no difference in the percentage of cases diagnosed within 30 days of an emergency hospital admission or A&E attendance between men and women tested using Chi-squared tests with Yates' continuity correction. Italicised conditions indicate that men had statistically significantly more emergency diagnoses; <u>underlined</u> conditions indicate that women had statistically significantly more emergency diagnoses. A&E, accident and emergency; CHD, coronary/ischaemic heart disease; COPD, chronic obstructive pulmonary disease; IBD, inflammatory bowel disease; PCOS, polycystic ovary syndrome; SBE, subacute bacterial endocarditis.

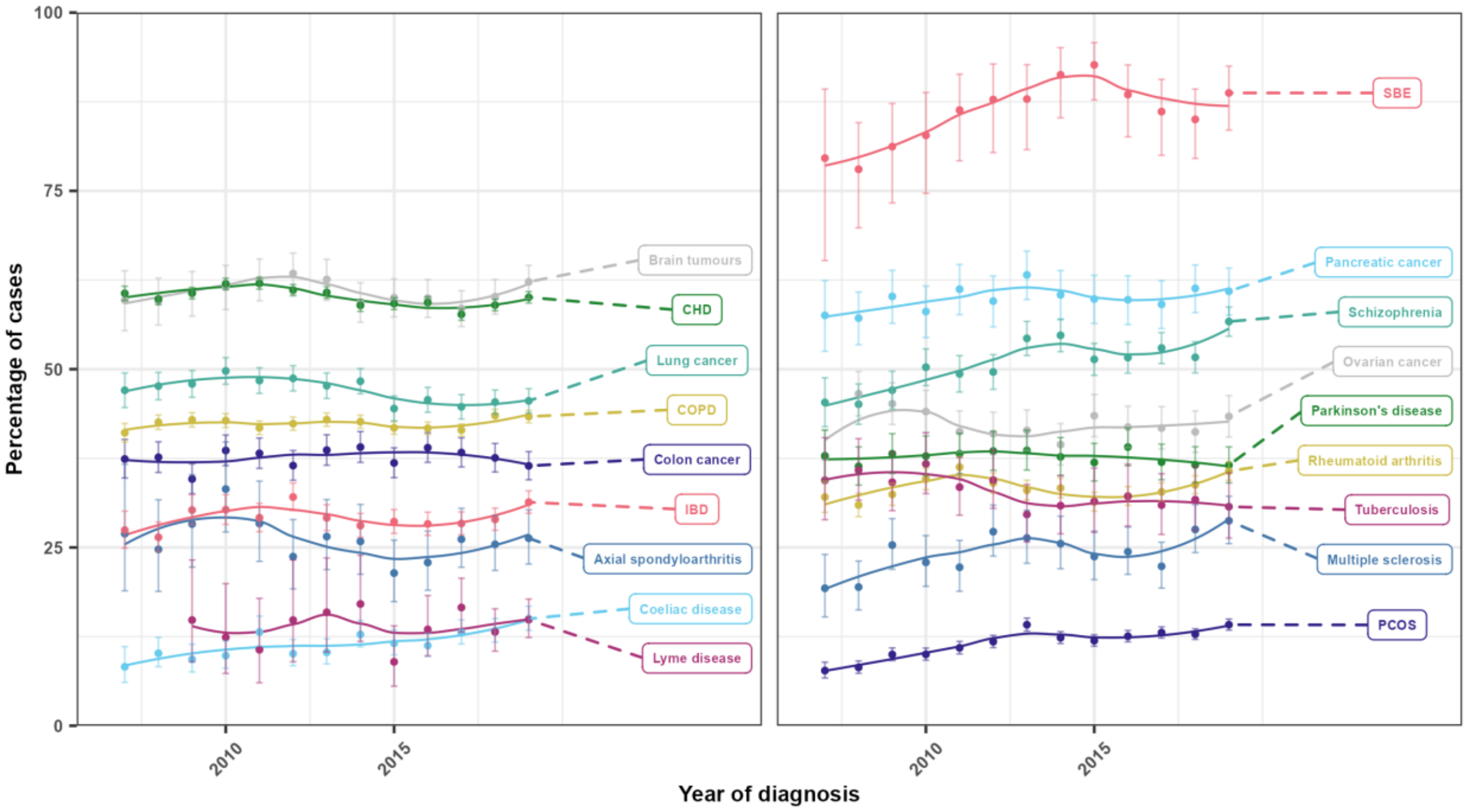

Figure 28. Percentage of cases diagnosed following an emergency presentation (emergency hospital admission or A&E attendance) by year of diagnosis – Aurum, Women. Note that only years in which at least 10 cases were diagnosed as an emergency and 10 cases not diagnosed as an emergency are shown. Lines are loess curves indicative of general trends over time. Conditions are split alphabetically across the two panels for visual clarity. CHD, coronary/ischaemic heart disease; COPD, chronic obstructive pulmonary disease; IBD, inflammatory bowel disease; PCOS, polycystic ovary syndrome; SBE, subacute bacterial endocarditis.

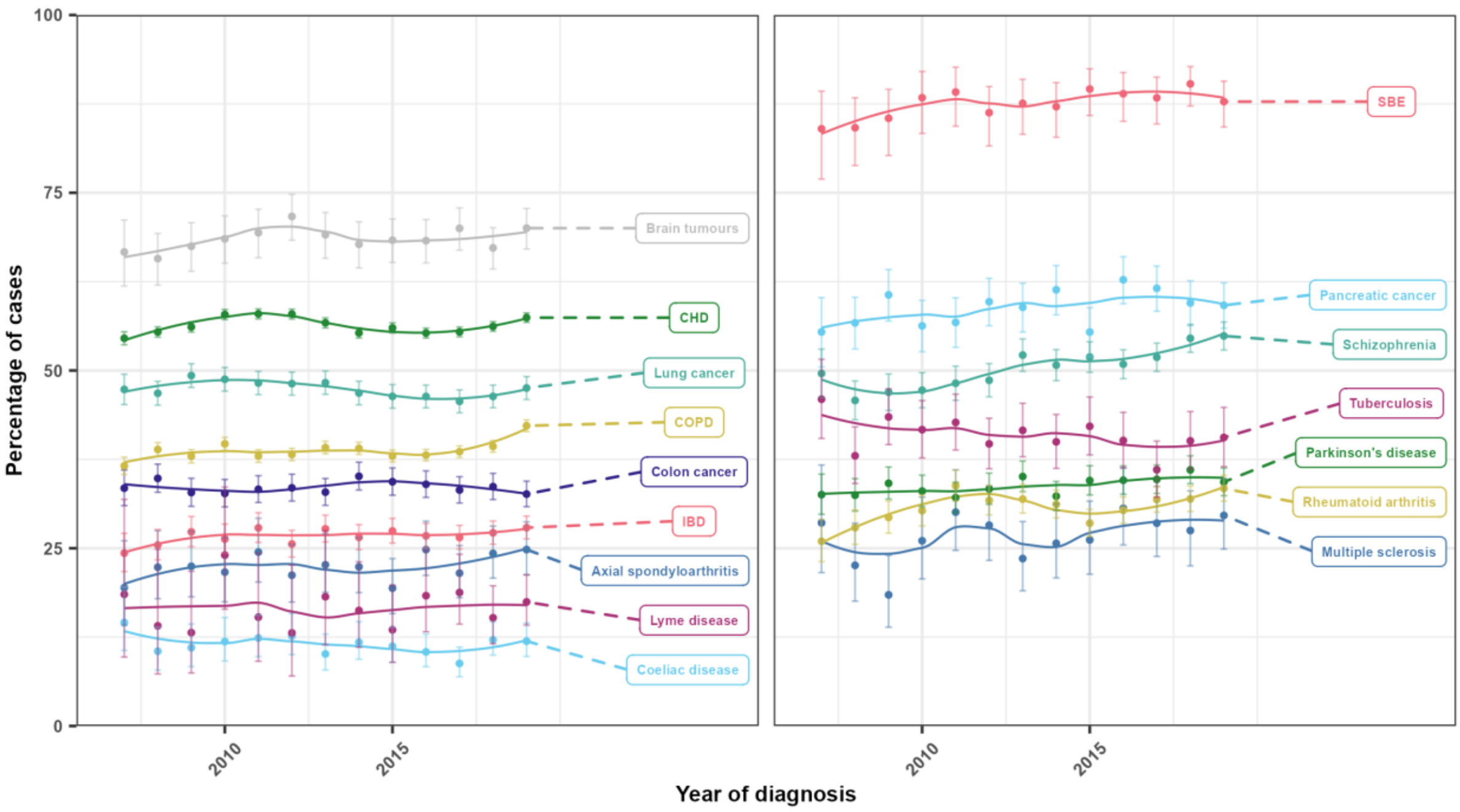

Figure 29. Percentage of cases diagnosed following an emergency presentation (emergency hospital admission or A&E attendance) by year of diagnosis – Aurum, Men. Note that only years in which at least 10 cases were diagnosed as an emergency and 10 cases not diagnosed as an emergency are shown. Lines are loess curves indicative of general trends over time. Conditions are split alphabetically across the two panels for visual clarity. CHD, coronary/ischaemic heart disease; COPD, chronic obstructive pulmonary disease; IBD, inflammatory bowel disease; SBE, subacute bacterial endocarditis.

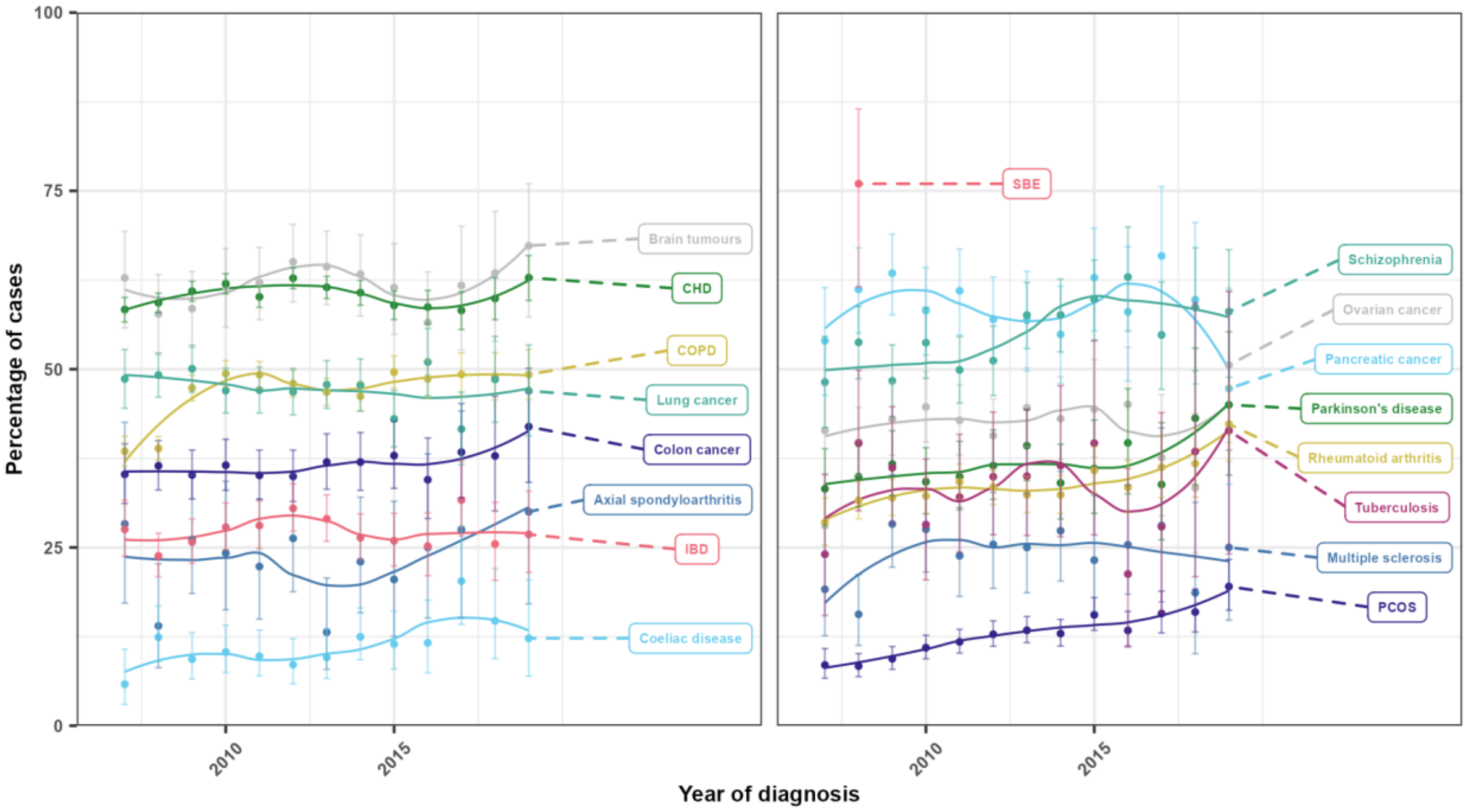

Figure 30. Percentage of cases diagnosed following an emergency presentation (emergency hospital admission or A&E attendance) by year of diagnosis – GOLD, Women. Note that only years in which at least 10 cases were diagnosed as an emergency and 10 cases not diagnosed as an emergency are shown. Lines are loess curves indicative of general trends over time. Conditions are split alphabetically across the two panels for visual clarity. CHD, coronary/ischaemic heart disease; COPD, chronic obstructive pulmonary disease; IBD, inflammatory bowel disease; PCOS, polycystic ovary syndrome; SBE, subacute bacterial endocarditis.

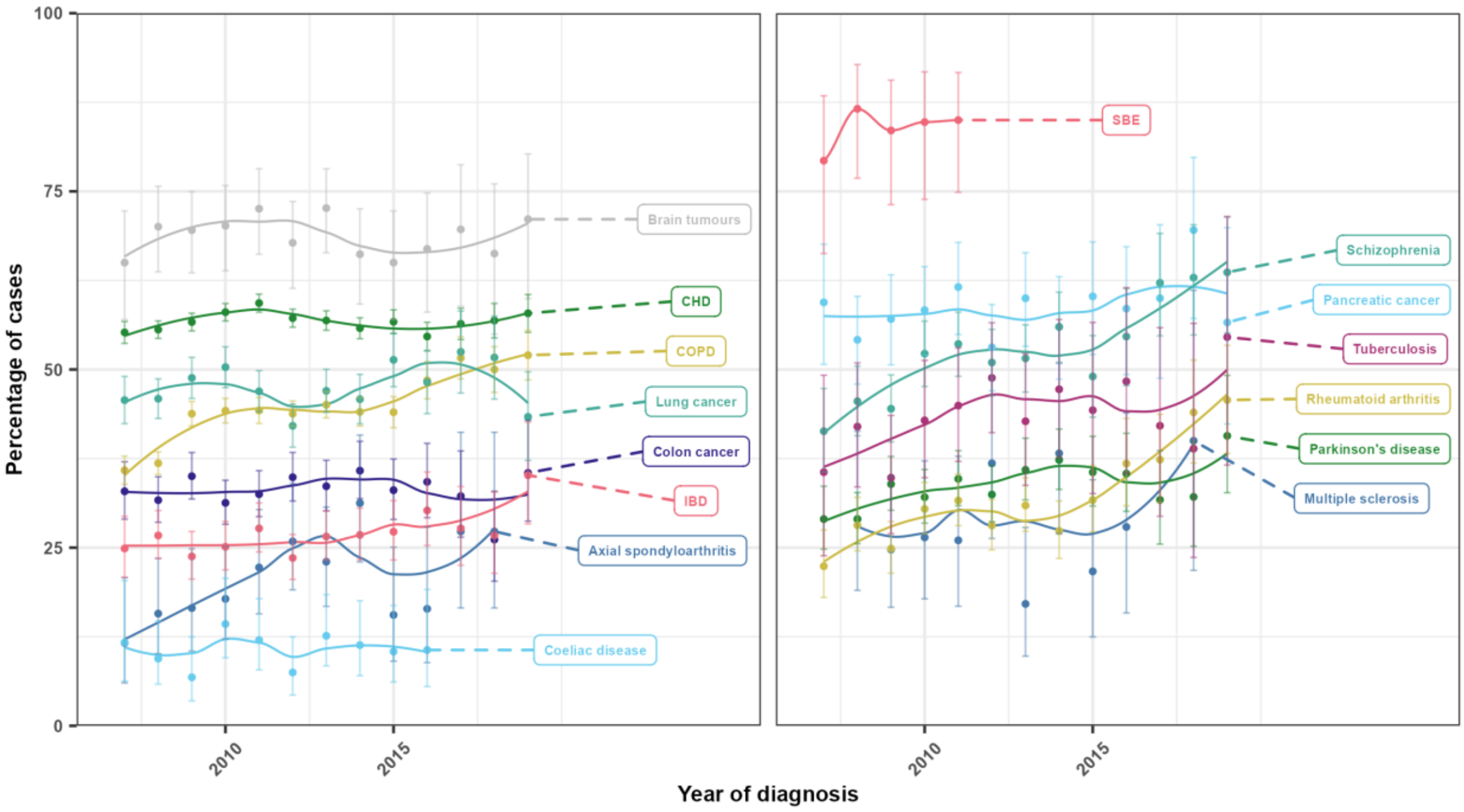

Figure 31. Percentage of cases diagnosed following an emergency presentation (emergency hospital admission or A&E attendance) by year of diagnosis – GOLD, Men. Note that only years in which at least 10 cases were diagnosed as an emergency and 10 cases not diagnosed as an emergency are shown. Lines are loess curves indicative of general trends over time. Conditions are split alphabetically across the two panels for visual clarity. CHD, coronary/ischaemic heart disease; COPD, chronic obstructive pulmonary disease; IBD, inflammatory bowel disease; SBE, subacute bacterial endocarditis.

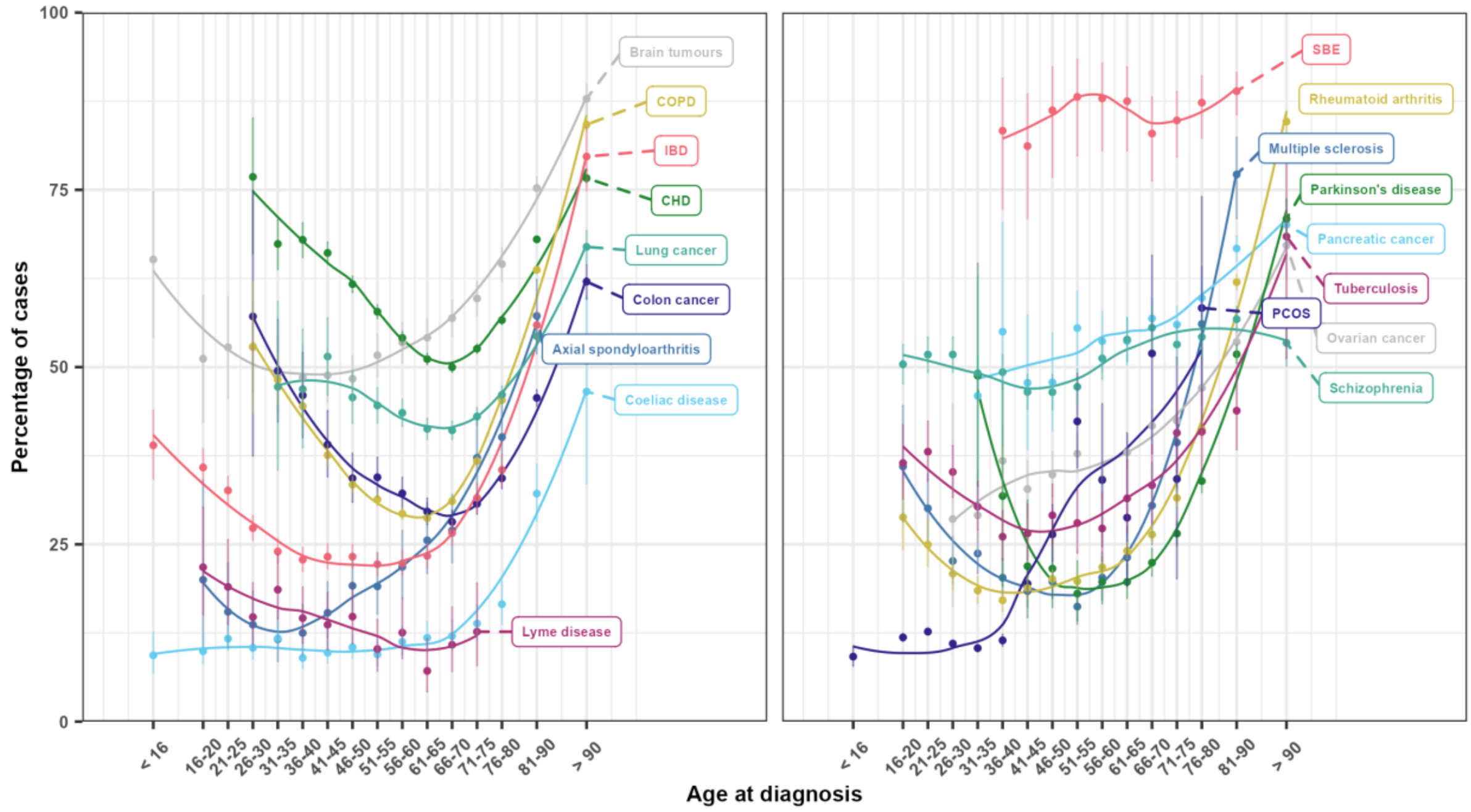

Figure 32. Percentage of cases diagnosed following an emergency presentation (emergency hospital admission or A&E attendance) by age at diagnosis - Aurum, Women. Note that only age groups with at least 10 cases diagnosed as an emergency and 10 cases not diagnosed as an emergency are shown. Lines are loess curves indicative of general patterns over age groups. Conditions are split alphabetically across the two panels for visual clarity. CHD, coronary/ischaemic heart disease; COPD, chronic obstructive pulmonary disease; IBD, inflammatory bowel disease; PCOS, polycystic ovary syndrome; SBE, subacute bacterial endocarditis.

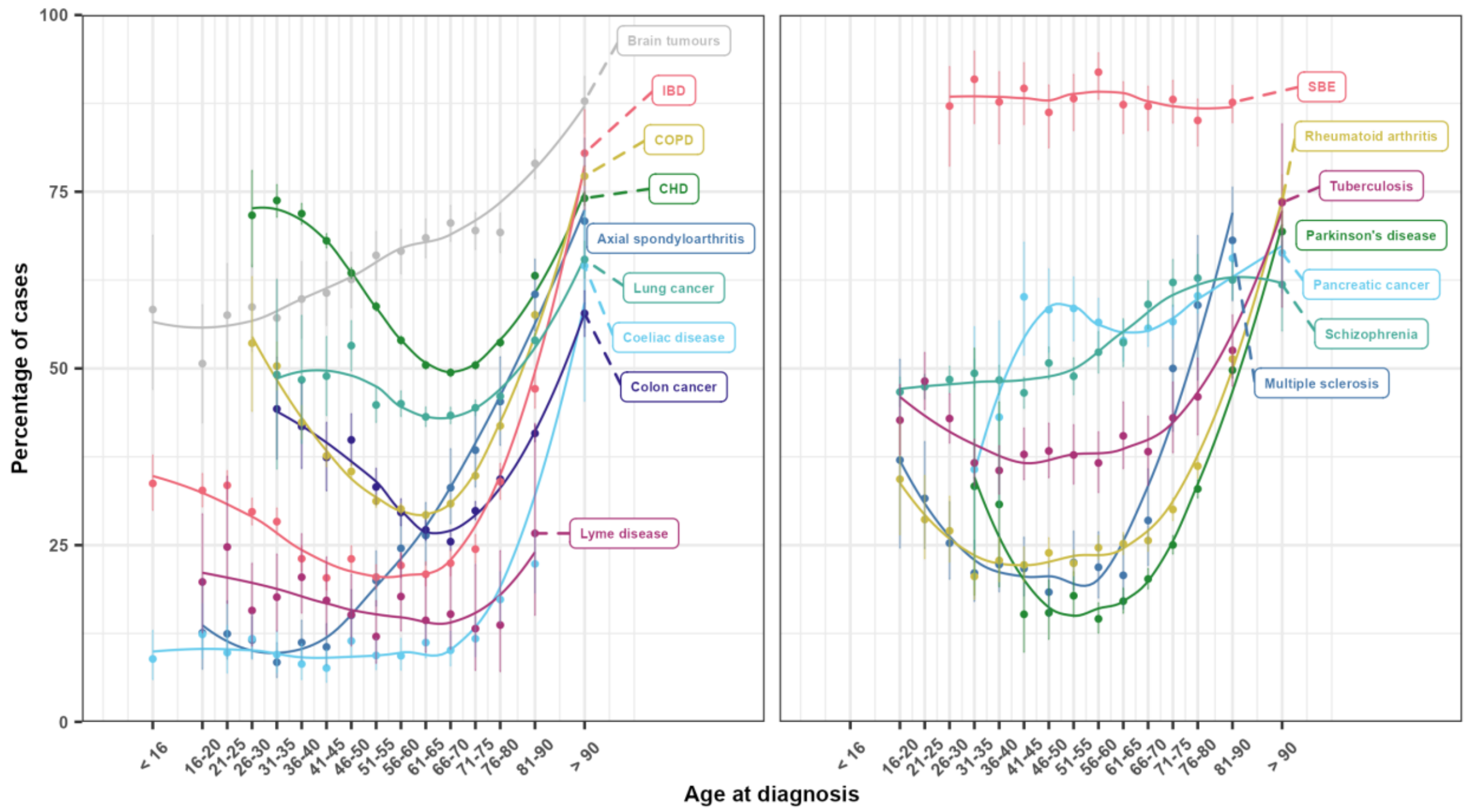

Figure 33. Percentage of cases diagnosed following an emergency presentation (emergency hospital admission or A&E attendance) by age at diagnosis - Aurum, Men. Note that only age groups with at least 10 cases diagnosed as an emergency and 10 cases not diagnosed as an emergency are shown. Lines are loess curves indicative of general patterns over age groups. Conditions are split alphabetically across the two panels for visual clarity. CHD, coronary/ischaemic heart disease; COPD, chronic obstructive pulmonary disease; IBD, inflammatory bowel disease; SBE, subacute bacterial endocarditis.

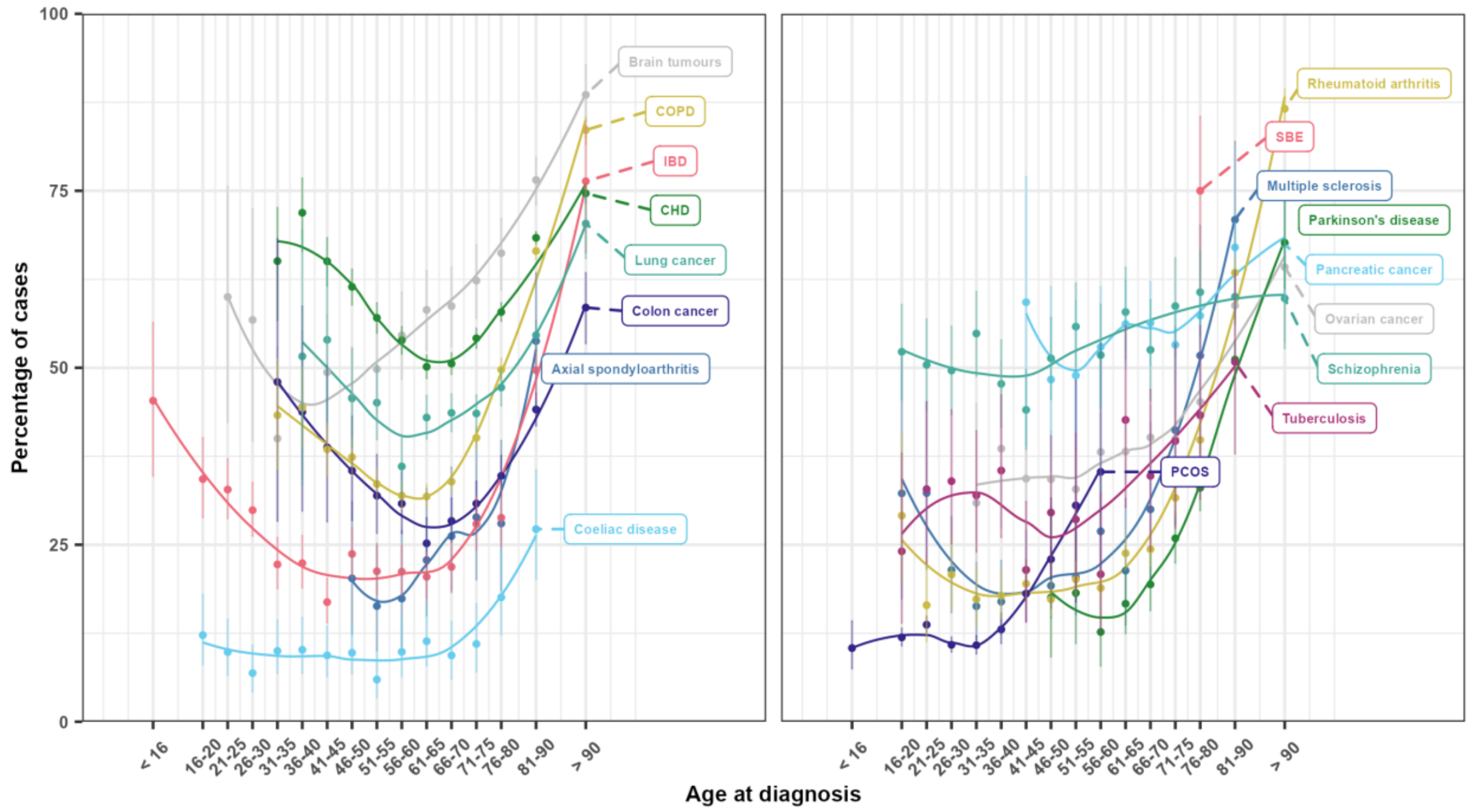

Figure 34. Percentage of cases diagnosed following an emergency presentation (emergency hospital admission or A&E attendance) by age at diagnosis - GOLD, Women. Note that only age groups with at least 10 cases diagnosed as an emergency and 10 cases not diagnosed as an emergency are shown. Lines are loess curves indicative of general patterns over age groups. Conditions are split alphabetically across the two panels for visual clarity. CHD, coronary/ischaemic heart disease; COPD, chronic obstructive pulmonary disease; IBD, inflammatory bowel disease; PCOS, polycystic ovary syndrome; SBE, subacute bacterial endocarditis.

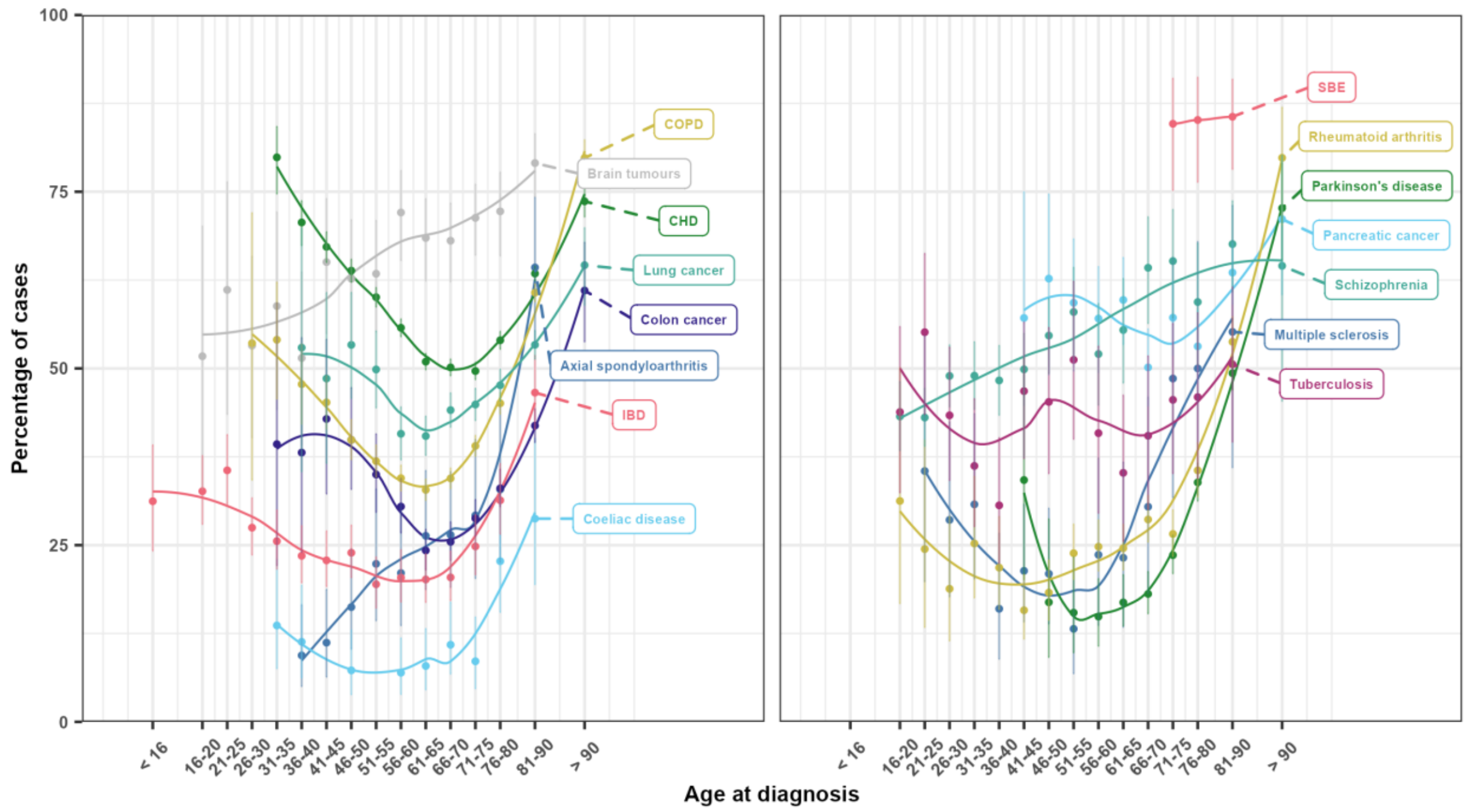

Figure 35. Percentage of cases diagnosed following an emergency presentation (emergency hospital admission or A&E attendance) by age at diagnosis - GOLD, Men. Note that only age groups with at least 10 cases diagnosed as an emergency and 10 cases not diagnosed as an emergency are shown. Lines are loess curves indicative of general patterns over age groups. Conditions are split alphabetically across the two panels for visual clarity. CHD, coronary/ischaemic heart disease; COPD, chronic obstructive pulmonary disease; IBD, inflammatory bowel disease; SBE, subacute bacterial endocarditis.

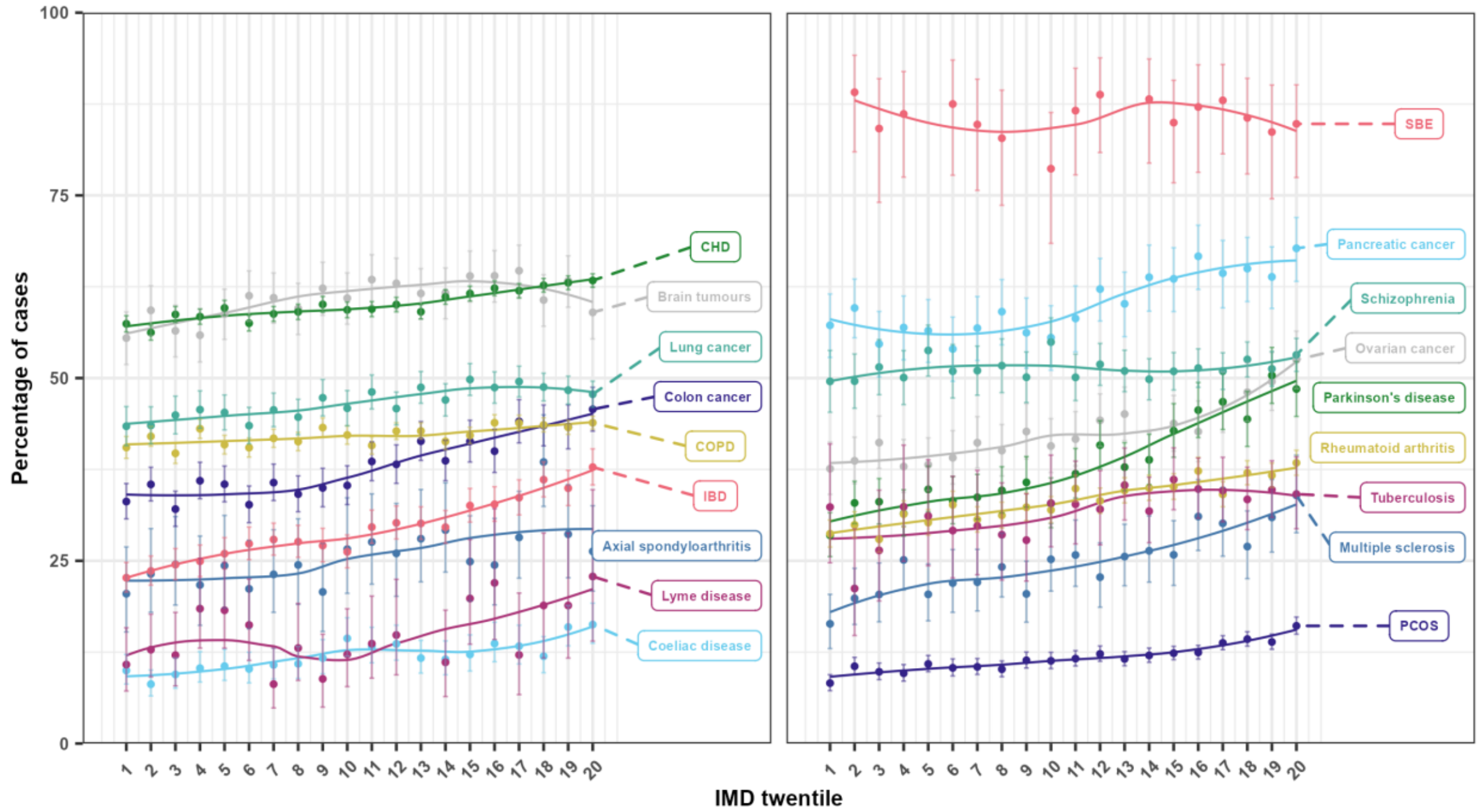

Figure 36. Percentage of cases diagnosed following an emergency presentation (emergency hospital admission or A&E attendance) by IMD twentile - Aurum, Women. Note that only twentiles with at least 10 cases diagnosed as an emergency and 10 cases not diagnosed as an emergency are shown. Lines are loess curves indicative of general patterns over twentiles. Note that 1 is least deprived, 20 is most deprived. Conditions are split alphabetically across the two panels for visual clarity. CHD, coronary/ischaemic heart disease; COPD, chronic obstructive pulmonary disease; IBD, inflammatory bowel disease; PCOS, polycystic ovary syndrome; SBE, subacute bacterial endocarditis.

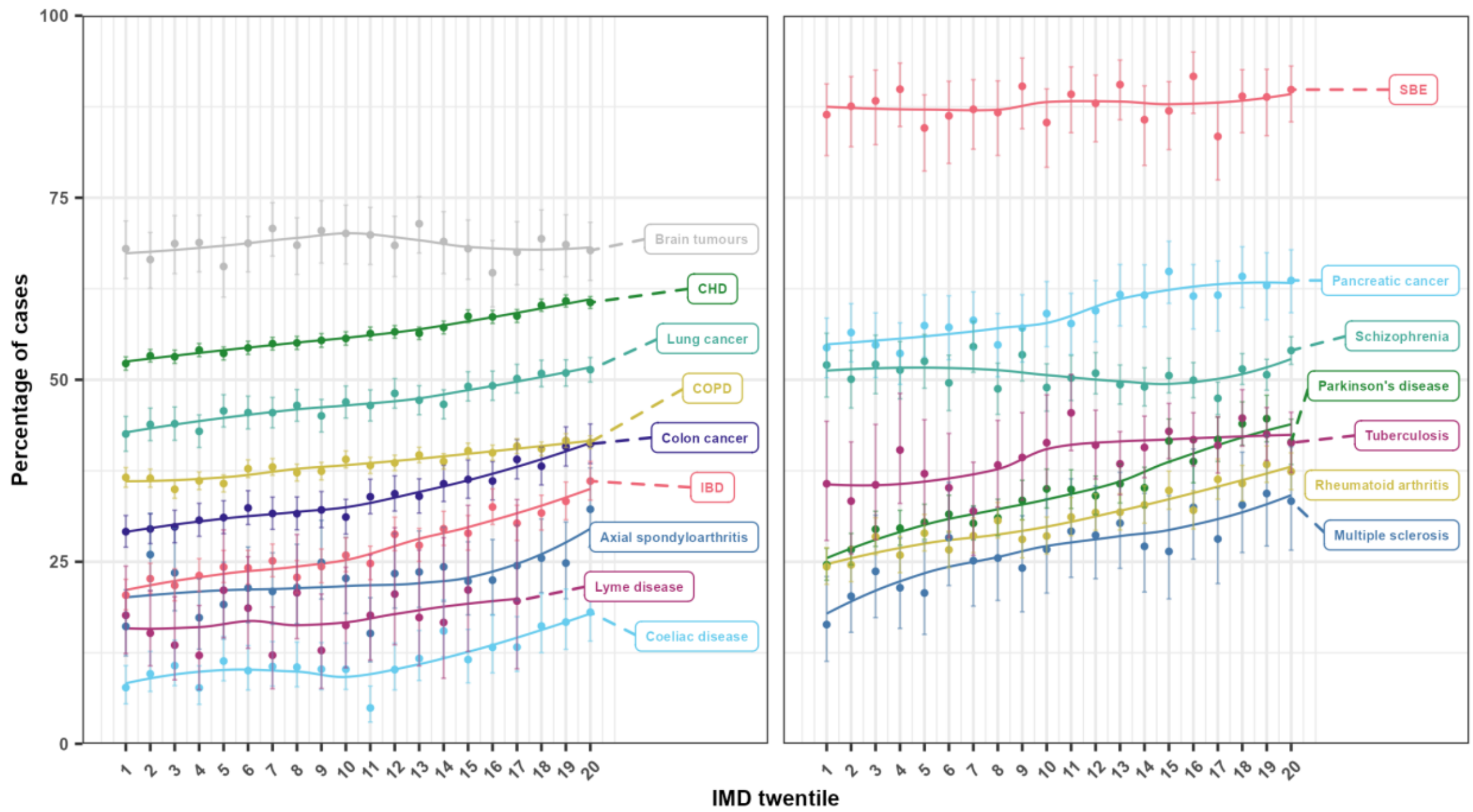

Figure 37. Percentage of cases diagnosed following an emergency presentation (emergency hospital admission or A&E attendance) by IMD twentile - Aurum, Men. Note that only twentiles with at least 10 cases diagnosed as an emergency and 10 cases not diagnosed as an emergency are shown. Lines are loess curves indicative of general patterns over twentiles. Note that 1 is least deprived, 20 is most deprived. Conditions are split alphabetically across the two panels for visual clarity. CHD, coronary/ischaemic heart disease; COPD, chronic obstructive pulmonary disease; IBD, inflammatory bowel disease; SBE, subacute bacterial endocarditis.

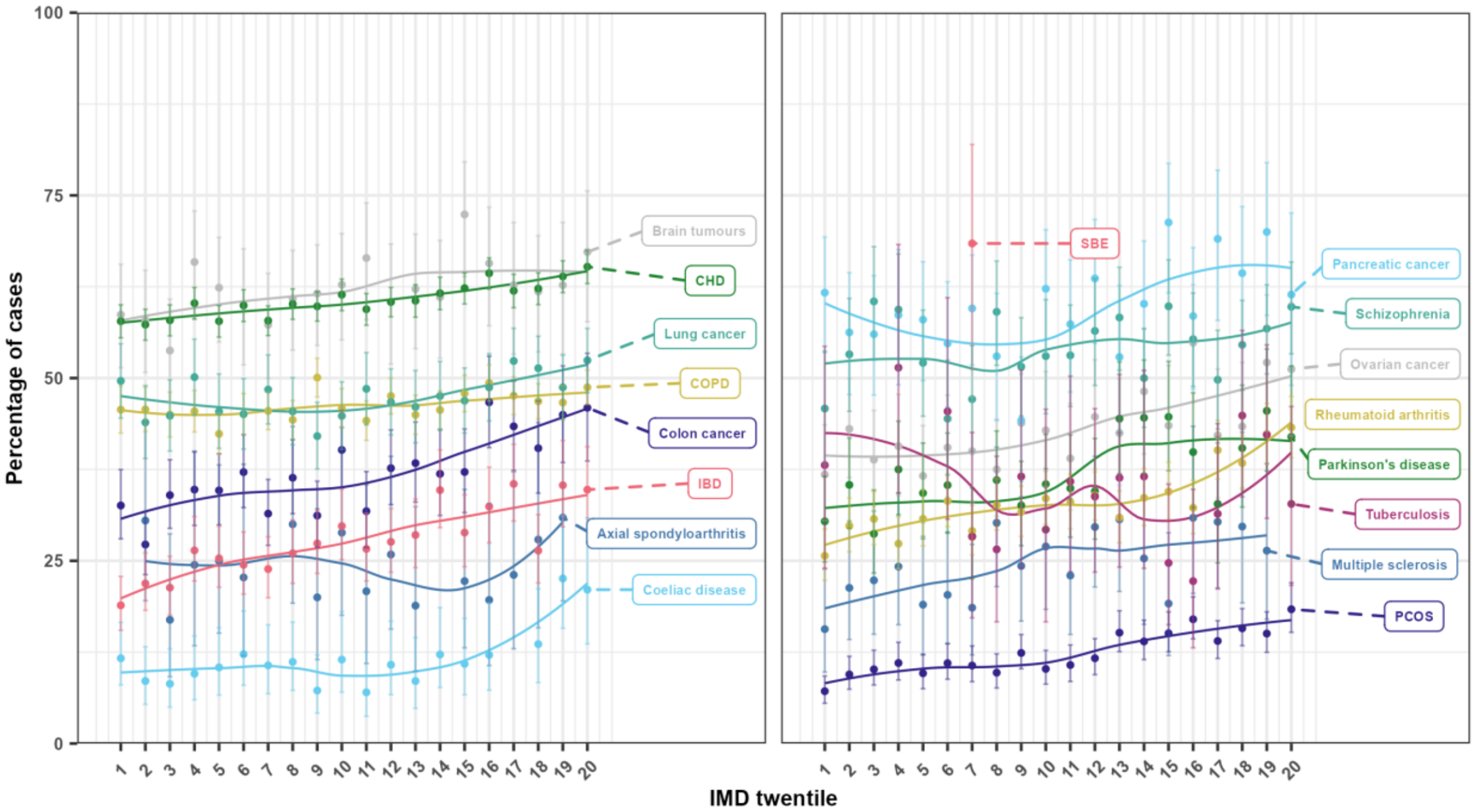

Figure 38. Percentage of cases diagnosed following an emergency presentation (emergency hospital admission or A&E attendance) by IMD twentile - GOLD, Women. Note that only twentiles with at least 10 cases diagnosed as an emergency and 10 cases not diagnosed as an emergency are shown. Lines are loess curves indicative of general patterns over twentiles. Note that 1 is least deprived, 20 is most deprived. Conditions are split alphabetically across the two panels for visual clarity. CHD, coronary/ischaemic heart disease; COPD, chronic obstructive pulmonary disease; IBD, inflammatory bowel disease; PCOS, polycystic ovary syndrome; SBE, subacute bacterial endocarditis.

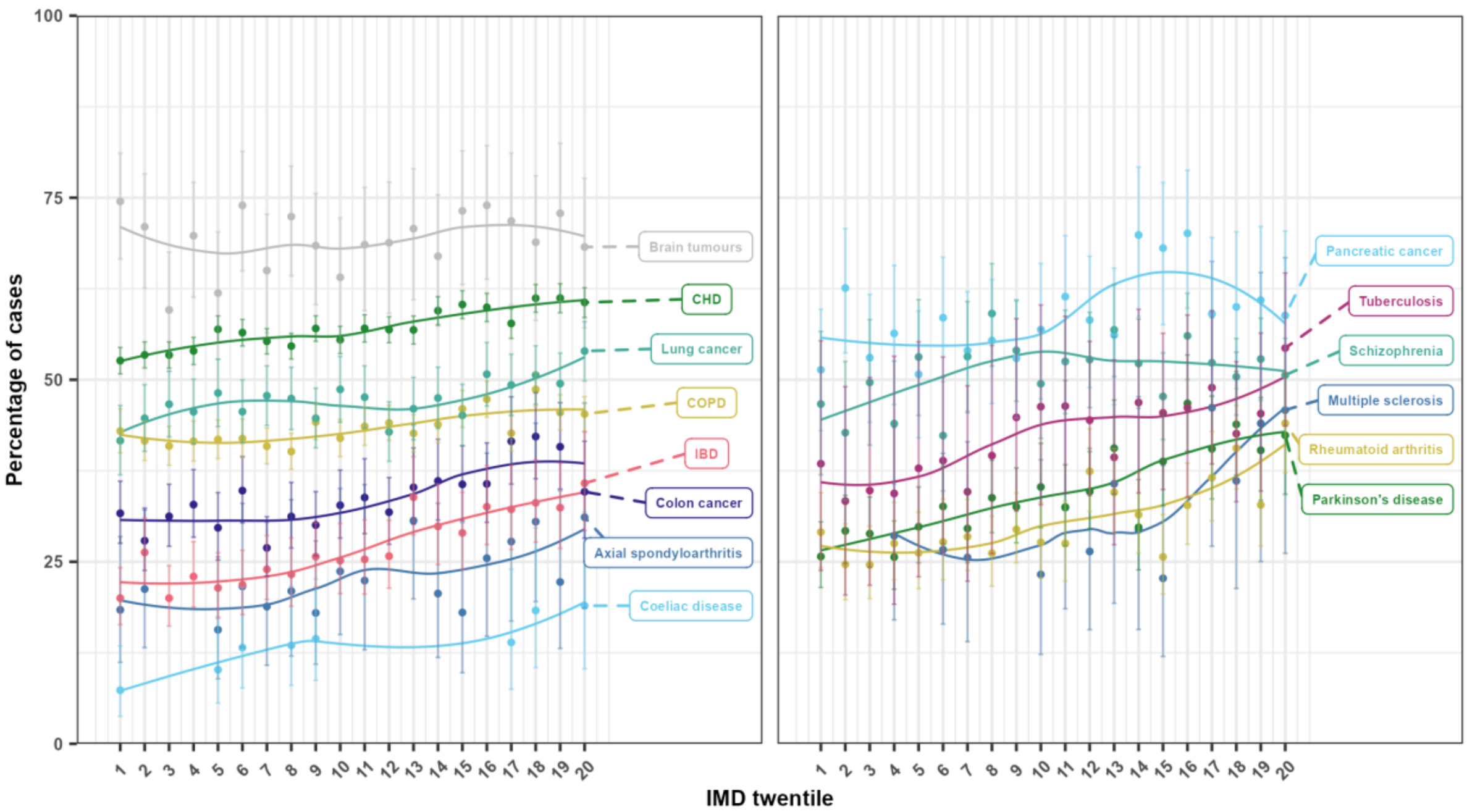

Figure 39. Percentage of cases diagnosed following an emergency presentation (emergency hospital admission or A&E attendance) by IMD twentile - GOLD, Men. Note that only twentiles with at least 10 cases diagnosed as an emergency and 10 cases not diagnosed as an emergency are shown. Lines are loess curves indicative of general patterns over twentiles. Note that 1 is least deprived, 20 is most deprived. Conditions are split alphabetically across the two panels for visual clarity. CHD, coronary/ischaemic heart disease; COPD, chronic obstructive pulmonary disease; IBD, inflammatory bowel disease; SBE, subacute bacterial endocarditis.

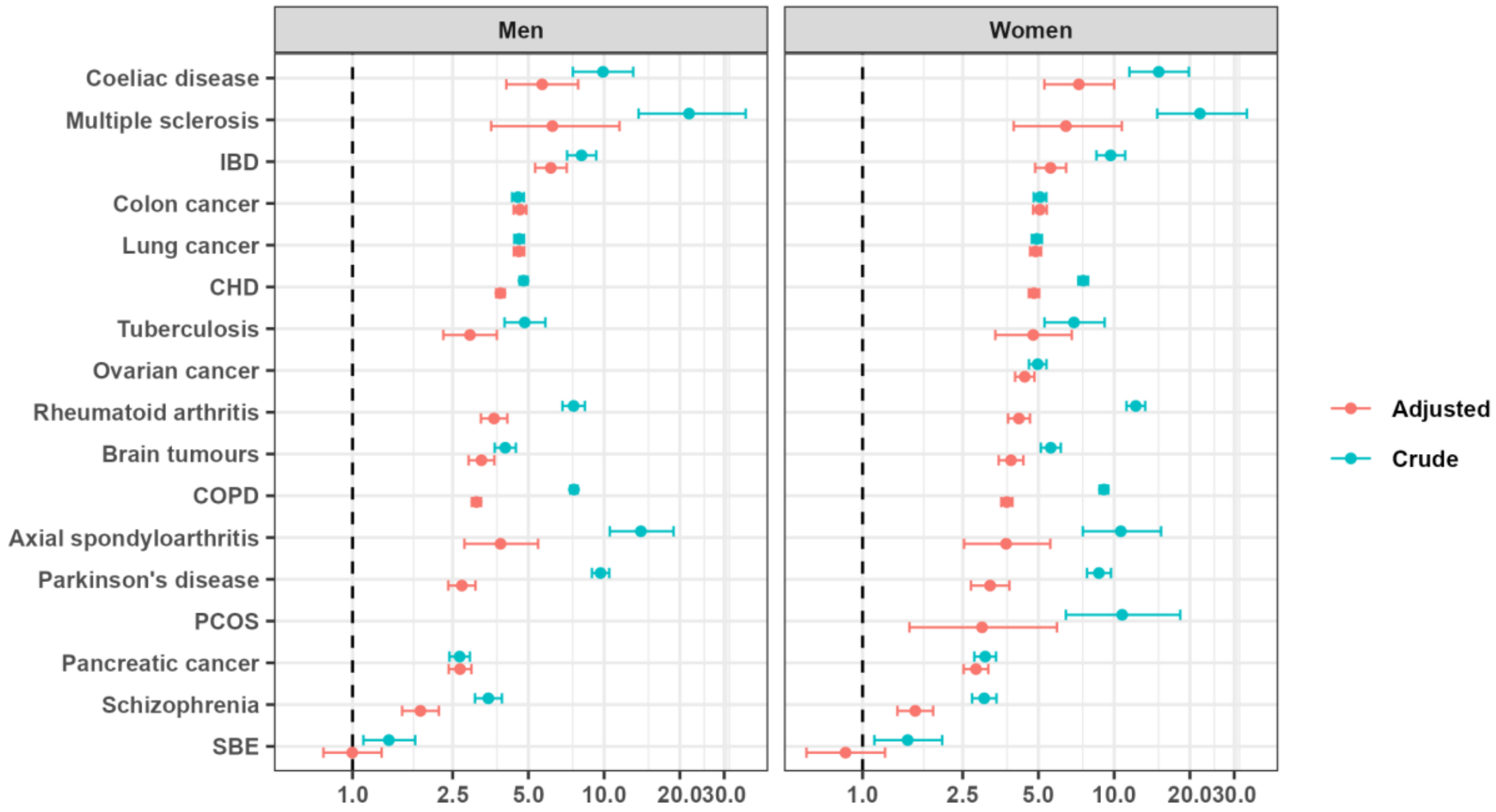

Figure 40. Forest plot of odds ratios for 1-year mortality following an emergency diagnosis (emergency hospital admission or A&E attendance) - Aurum. Note that only gender combinations with at least 10 emergency diagnosed patients who died in the year after diagnosis were examined – consequently Lyme disease is not included. Odds ratios were adjusted for age at diagnosis, year of diagnosis, IMD score, comorbidity score six months (180 days) prior to diagnosis and diagnosis source. CHD, coronary/ischaemic heart disease; COPD, chronic obstructive pulmonary disease; IBD, inflammatory bowel disease; PCOS, polycystic ovary syndrome; SBE, subacute bacterial endocarditis.

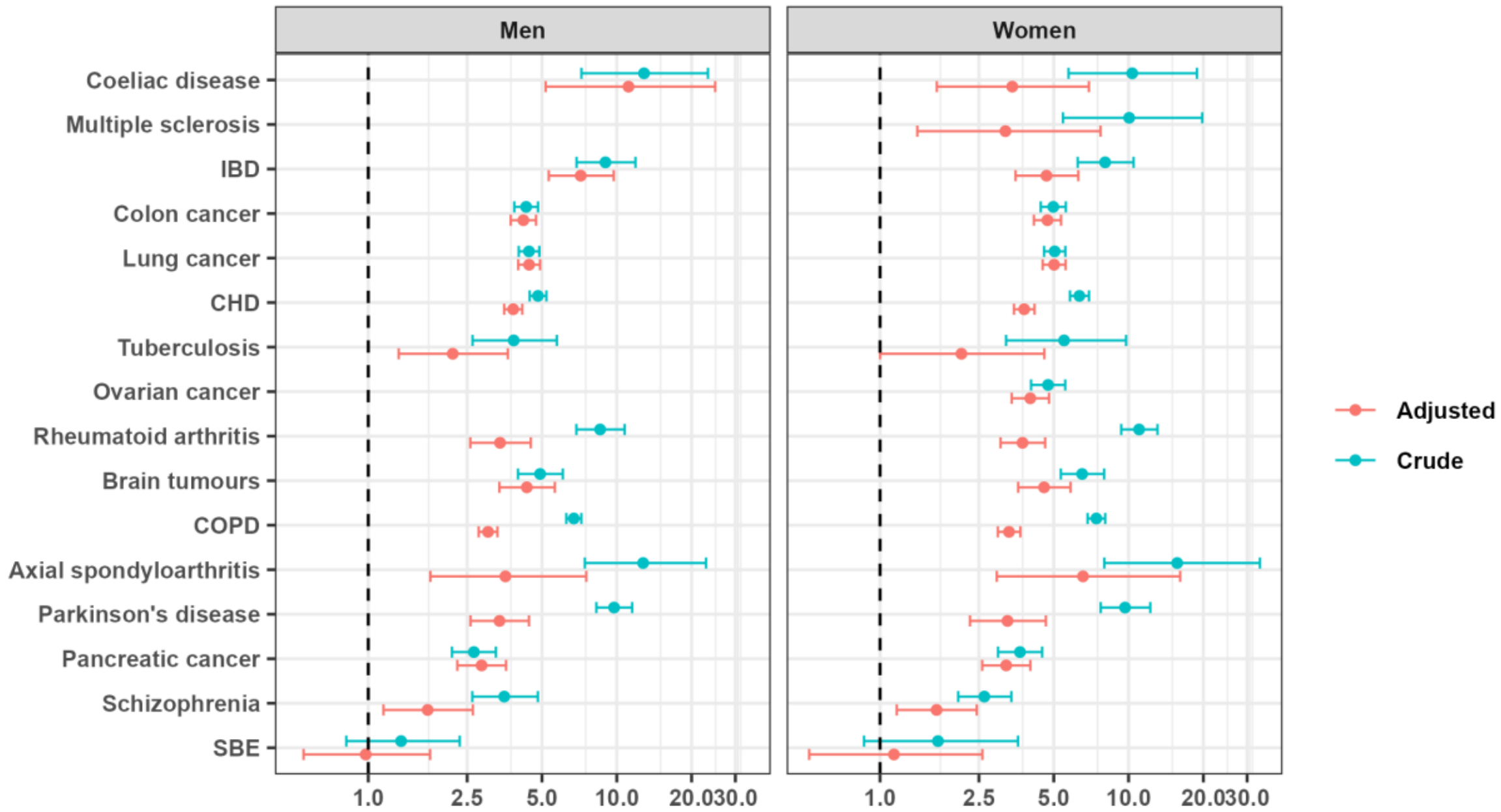

Figure 41. Forest plot of odds ratios for 1-year mortality following an emergency diagnosis (emergency hospital admission or A&E attendance) - GOLD. Note that only gender combinations with at least 10 emergency diagnosed patients who died in the year after diagnosis were examined – consequently Lyme disease is not included. Odds ratios were adjusted for age at diagnosis, year of diagnosis, IMD score, comorbidity score six months (180 days) prior to diagnosis and diagnosis source. CHD, coronary/ischaemic heart disease; COPD, chronic obstructive pulmonary disease; IBD, inflammatory bowel disease; SBE, subacute bacterial endocarditis.

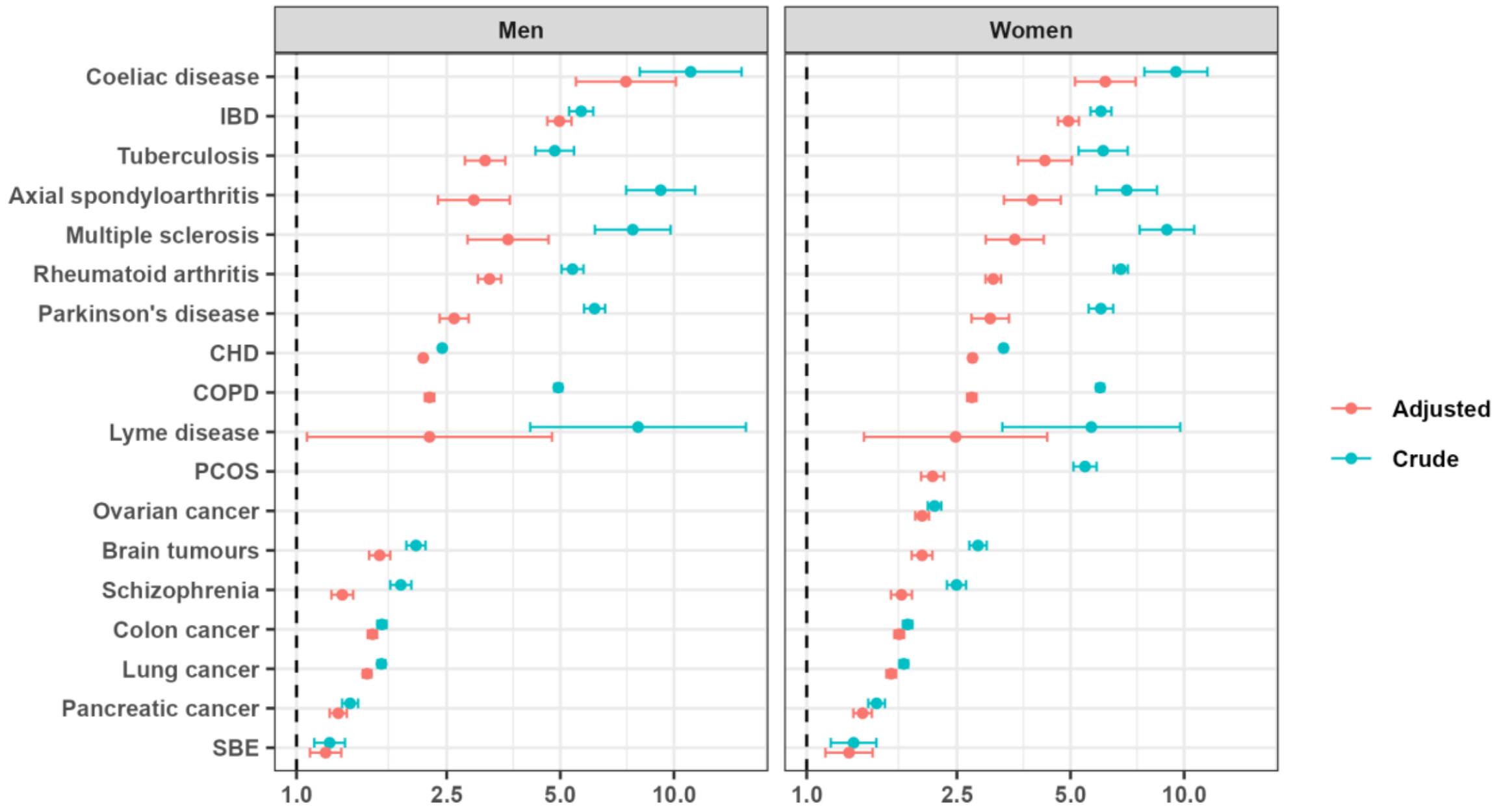

Figure 42. Forest plot of rate ratios for time in hospital in the year post-diagnosis following an emergency diagnosis (emergency hospital admission or A&E attendance) – Aurum. Rate ratios were adjusted for age at diagnosis, year of diagnosis, IMD score, comorbidity score six months (180 days) prior to diagnosis and diagnosis source. CHD, coronary/ischaemic heart disease; COPD, chronic obstructive pulmonary disease; IBD, inflammatory bowel disease; PCOS, polycystic ovary syndrome; SBE, subacute bacterial endocarditis.

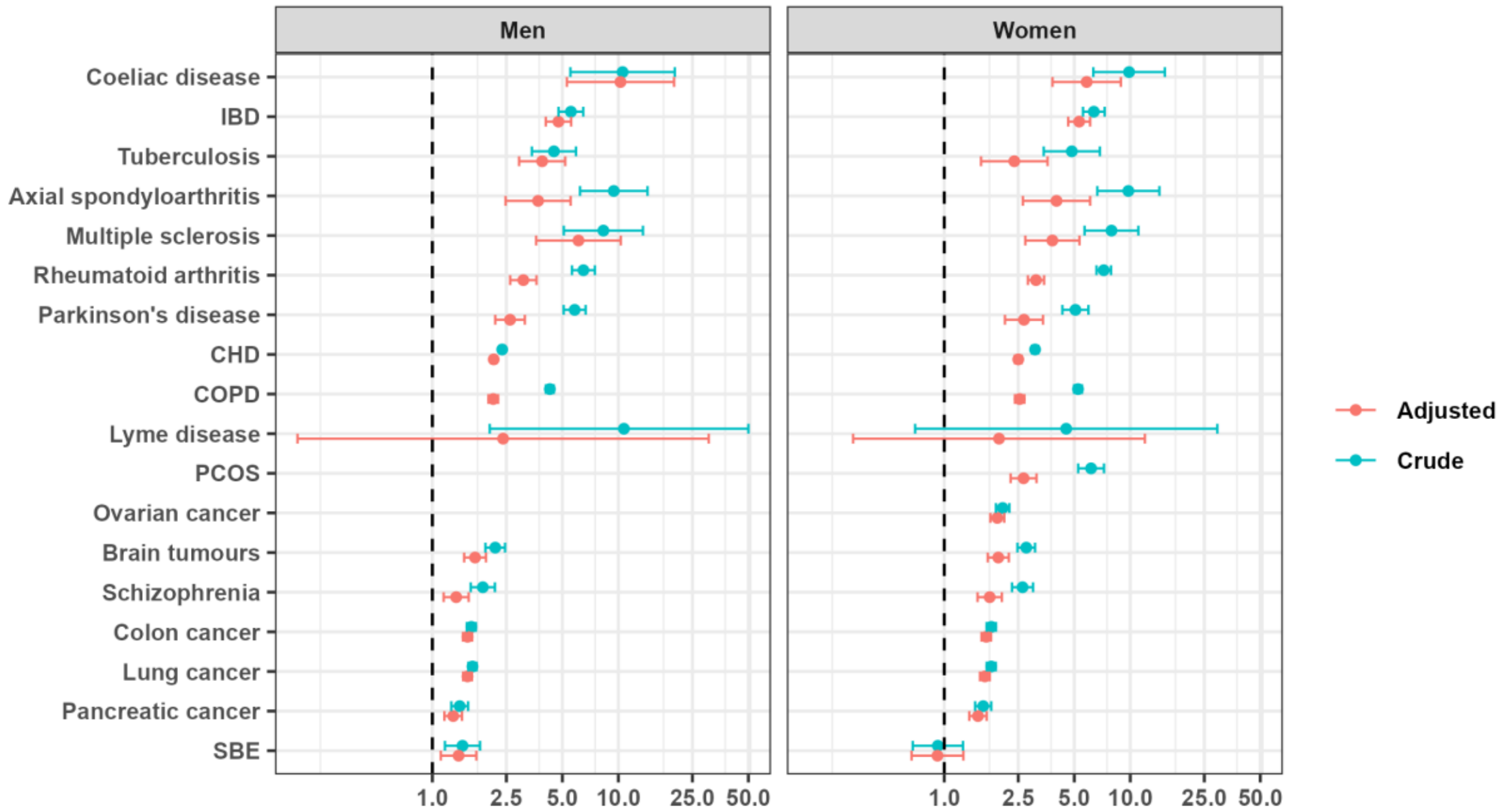

Figure 43. Forest plot of rate ratios for time in hospital in the year post-diagnosis following an emergency diagnosis (emergency hospital admission or A&E attendance) – GOLD. Rate ratios were adjusted for age at diagnosis, year of diagnosis, IMD score, comorbidity score six months (180 days) prior to diagnosis and diagnosis source. N.B. Upper confidence intervals that exceed 50 have been reduced to 50 for visualisation purposes. CHD, coronary/ischaemic heart disease; COPD, chronic obstructive pulmonary disease; IBD, inflammatory bowel disease; PCOS, polycystic ovary syndrome; SBE, subacute bacterial endocarditis.

Table 44. Observed 1-year mortality and odds ratios (ORs) by emergency diagnosis status (emergency hospitalisation or A&E attendance), condition, gender, and CPRD dataset.

Condition	Gender	Emergency diagnosed		Non-emergency diagnosed		Crude OR (95% CI)	Adjusted [b] OR (95% CI)	p-value [c]
		N patients [a]	1-year mortality, % (n)	N patients [a]	1-year mortality, % (n)			
Aurum								
Axial spondyloarthritis	Men	1,215	18% (213)	4,155	1.5% (62)	14.0 (10.5, 18.9)	3.87 (2.78, 5.46)	0.877
	Women	1,085	12% (134)	3,135	1.3% (41)	10.6 (7.50, 15.4)	3.72 (2.53, 5.57)	
Brain tumours	Men	7,150	52% (3,719)	3,180	21% (672)	4.04 (3.67, 4.46)	3.25 (2.89, 3.66)	0.0323
	Women	9,185	41% (3,799)	5,845	11% (654)	5.60 (5.11, 6.14)	3.89 (3.48, 4.35)	
Coeliac disease	Men	855	13% (115)	6,745	1.5% (104)	9.90 (7.51, 13.1)	5.67 (4.09, 7.89)	0.295
	Women	1,870	8.2% (154)	13,980	0.59% (83)	15.0 (11.5, 19.8)	7.24 (5.28, 10.0)	
CHD	Men	124,400	17% (21,432)	85,205	4.2% (3,553)	4.78 (4.61, 4.96)	3.87 (3.72, 4.03)	< 0.01
	Women	97,320	24% (23,296)	57,165	4.0% (2,293)	7.53 (7.21, 7.87)	4.80 (4.58, 5.03)	
Colon cancer	Men	9,670	49% (4,772)	19,570	18% (3,453)	4.55 (4.31, 4.80)	4.63 (4.37, 4.91)	0.0383
	Women	9,445	51% (4,857)	15,835	17% (2,733)	5.07 (4.79, 5.38)	5.07 (4.76, 5.40)	
COPD	Men	57,970	32% (18,408)	91,545	5.8% (5,290)	7.59 (7.34, 7.84)	3.11 (2.98, 3.24)	< 0.01
	Women	58,100	28% (16,214)	79,290	4.1% (3,234)	9.10 (8.75, 9.47)	3.74 (3.56, 3.94)	
IBD	Men	7,665	11% (832)	20,945	1.5% (309)	8.13 (7.12, 9.30)	6.14 (5.32, 7.10)	0.361
	Women	9,325	11% (1,054)	22,705	1.3% (295)	9.68 (8.50, 11.1)	5.58 (4.85, 6.45)	
Lung cancer	Men	20,265	82% (16,686)	22,580	50% (11,373)	4.59 (4.39, 4.80)	4.59 (4.38, 4.81)	0.0908
	Women	17,120	78% (13,403)	19,130	42% (8,086)	4.93 (4.71, 5.16)	4.87 (4.63, 5.13)	
Lyme disease	Men	365	-	1,815	0.77% (14)			
	Women	415	-	2,585	-			
Multiple sclerosis	Men	955	14% (131)	2,625	0.72% (19)	21.8 (13.7, 36.5)	6.23 (3.56, 11.5)	0.936
	Women	1,890	9.3% (175)	5,835	0.46% (27)	21.9 (14.8, 33.7)	6.43 (3.99, 10.7)	
Ovarian cancer	Women	5,600	49% (2,737)	7,720	16% (1,246)	4.96 (4.58, 5.38)	4.41 (4.04, 4.82)	
Pancreatic cancer	Men	5,730	82% (4,674)	3,815	62% (2,381)	2.66 (2.43, 2.93)	2.67 (2.41, 2.97)	0.491
	Women	5,535	84% (4,637)	3,420	63% (2,145)	3.07 (2.78, 3.39)	2.82 (2.52, 3.16)	
Parkinson's disease	Men	8,390	37% (3,115)	16,385	5.7% (942)	9.68 (8.94, 10.5)	2.72 (2.40, 3.08)	0.129
	Women	6,040	29% (1,749)	10,005	4.5% (448)	8.69 (7.80, 9.71)	3.21 (2.70, 3.83)	
PCOS	Women	8,865	0.39% (35)	65,150	0.037% (24)	10.8 (6.43, 18.3)	2.98 (1.53, 5.92)	
Rheumatoid arthritis	Men	8,100	19% (1,518)	18,045	3.0% (534)	7.56 (6.83, 8.38)	3.65 (3.24, 4.12)	0.0906
	Women	18,545	18% (3,341)	37,130	1.8% (658)	12.2 (11.2, 13.3)	4.18 (3.78, 4.63)	
SBE	Men	3,400	31% (1,048)	415	24% (100)	1.39 (1.10, 1.77)	0.998 (0.766, 1.30)	0.501
	Women	1,600	38% (608)	220	29% (63)	1.51 (1.11, 2.07)	0.856 (0.599, 1.23)	
Schizophrenia	Men	12,495	9.2% (1,148)	12,090	2.8% (343)	3.47 (3.07, 3.93)	1.86 (1.57, 2.20)	0.243
	Women	11,340	11% (1,304)	10,695	4.1% (438)	3.04 (2.72, 3.41)	1.62 (1.38, 1.91)	
Tuberculosis	Men	3,070	15% (456)	4,565	3.5% (159)	4.84 (4.02, 5.84)	2.93 (2.30, 3.75)	0.0249
	Women	1,935	11% (212)	4,070	1.7% (71)	6.92 (5.29, 9.16)	4.77 (3.37, 6.78)	
GOLD								
Axial spondyloarthritis	Men	270	19% (50)	1,040	1.7% (18)	12.8 (7.44, 22.9)	3.56 (1.78, 7.54)	0.277
	Women	240	16% (38)	835	1.2% (10)	15.7 (7.99, 33.8)	6.56 (2.95, 16.1)	

Condition	Gender	Emergency diagnosed		Non-emergency diagnosed		Crude OR (95% CI)	Adjusted [b] OR (95% CI)	p-value [c]
		N patients [a]	1-year mortality, % (n)	N patients [a]	1-year mortality, % (n)			
Brain tumours	Men	1,590	57% (907)	690	21% (147)	4.91 (4.00, 6.06)	4.34 (3.37, 5.63)	0.777
	Women	1,970	45% (887)	1,200	11% (134)	6.50 (5.34, 7.97)	4.57 (3.60, 5.84)	
Coeliac disease	Men	180	16% (28)	1,550	1.4% (22)	12.9 (7.21, 23.3)	11.1 (5.17, 24.9)	0.0262
	Women	365	6.8% (25)	2,975	0.71% (21)	10.3 (5.74, 18.8)	3.40 (1.69, 6.92)	
CHD	Men	28,045	17% (4,903)	18,850	4.2% (794)	4.82 (4.46, 5.21)	3.83 (3.52, 4.17)	0.910
	Women	21,930	24% (5,340)	12,295	4.8% (594)	6.34 (5.81, 6.93)	3.80 (3.46, 4.18)	
Colon cancer	Men	2,320	49% (1,126)	4,795	18% (859)	4.32 (3.87, 4.82)	4.21 (3.74, 4.73)	0.194
	Women	2,255	51% (1,148)	4,005	17% (690)	4.98 (4.43, 5.59)	4.72 (4.16, 5.35)	
COPD	Men	12,635	34% (4,328)	16,330	7.2% (1,176)	6.72 (6.26, 7.20)	3.03 (2.78, 3.31)	0.216
	Women	12,655	30% (3,736)	14,650	5.3% (783)	7.42 (6.84, 8.05)	3.30 (2.98, 3.67)	
IBD	Men	1,715	12% (209)	4,810	1.5% (73)	9.00 (6.89, 11.9)	7.16 (5.32, 9.72)	0.0457
	Women	2,070	11% (223)	5,545	1.5% (82)	8.05 (6.24, 10.5)	4.67 (3.51, 6.28)	
Lung cancer	Men	4,655	84% (3,912)	5,225	54% (2,835)	4.43 (4.03, 4.88)	4.44 (4.01, 4.91)	0.103
	Women	3,995	81% (3,217)	4,305	45% (1,940)	5.04 (4.57, 5.57)	5.02 (4.51, 5.58)	
Lyme disease	Men	40	-	265	-			
	Women	40	-	280	-			
Multiple sclerosis	Men	200	12% (24)	565	-			
	Women	435	8.7% (38)	1,370	0.95% (13)	10.1 (5.45, 19.8)	3.20 (1.41, 7.71)	
Ovarian cancer	Women	1,410	49% (697)	1,925	17% (328)	4.74 (4.05, 5.56)	4.02 (3.39, 4.79)	
Pancreatic cancer	Men	1,215	82% (999)	840	63% (532)	2.66 (2.17, 3.26)	2.86 (2.28, 3.59)	0.464
	Women	1,355	86% (1,159)	855	61% (525)	3.66 (2.98, 4.49)	3.22 (2.58, 4.03)	
Parkinson's disease	Men	1,860	38% (705)	3,705	5.9% (218)	9.75 (8.28, 11.5)	3.37 (2.58, 4.43)	0.878
	Women	1,375	30% (412)	2,410	4.2% (102)	9.68 (7.73, 12.2)	3.26 (2.30, 4.65)	
PCOS	Women	1,700	0.59% (10)	12,045	-			
Rheumatoid arthritis	Men	1,760	19% (342)	4,010	2.7% (110)	8.57 (6.88, 10.8)	3.39 (2.57, 4.51)	0.577
	Women	4,250	19% (796)	8,595	2% (176)	11.0 (9.34, 13.1)	3.74 (3.05, 4.62)	
SBE	Men	660	32% (213)	80	26% (21)	1.36 (0.816, 2.33)	0.977 (0.549, 1.77)	0.761
	Women	335	41% (138)	40	30% (12)	1.71 (0.862, 3.59)	1.14 (0.519, 2.58)	
Schizophrenia	Men	2,275	8.7% (197)	2,135	2.6% (56)	3.52 (2.62, 4.81)	1.73 (1.15, 2.63)	0.927
	Women	2,195	12% (265)	1,855	5.0% (92)	2.63 (2.06, 3.38)	1.69 (1.17, 2.45)	
Tuberculosis	Men	545	18% (99)	710	5.5% (39)	3.85 (2.63, 5.74)	2.19 (1.33, 3.64)	0.951
	Women	350	13% (45)	725	2.6% (19)	5.50 (3.21, 9.78)	2.13 (1.00, 4.58)	

[a] To prevent backwards calculation of small patient groups the number of patients has been reported rounded to 5.

[b] Adjusted for age at diagnosis, year of diagnosis, IMD score, comorbidity score six months (180 days) prior to diagnosis and diagnosis source. Note that p-value indicates difference between genders.

[c] Note that p-values relate to the null hypothesis that there was no difference in the adjusted OR between men and women tested using likelihood ratio tests.

"–" indicates that fewer than 10 patients died within 1 year of diagnosis. Odds ratios were not calculated for these conditions as the results are unlikely to be reliable.

CI, confidence interval; OR, odds ratio; CHD, coronary/ischaemic heart disease; COPD, chronic obstructive pulmonary disease; IBD, inflammatory bowel disease; PCOS, polycystic ovary syndrome; SBE, subacute bacterial endocarditis.

Table 45. Observed mean number of hospital inpatient nights in year after diagnosis and rate ratios (RRs) by emergency diagnosis status (emergency hospitalisation or A&E attendance), condition, gender, and CPRD dataset.

Condition	Gender	Emergency diagnosed			Non-emergency diagnosed			Crude RR (95% CI)	Adjusted [c] RR (95% CI)	p-value [d]
		N patients [a]	Admitted, % (n) [b]	Mean admitted duration	N patients [a]	Admitted, % (n) [b]	Mean admitted duration			
Aurum										
Axial spondyloarthritis	Men	1,215	70% (854)	19.3	4,155	15% (635)	2.1	9.22 (7.47, 11.4)	2.95 (2.37, 3.67)	0.0363
	Women	1,085	70% (763)	15.3	3,135	24% (753)	2.2	7.04 (5.86, 8.47)	3.96 (3.33, 4.71)	
Brain tumours	Men	7,150	92.1% (6,583)	25.3	3,180	59% (1,861)	12.2	2.07 (1.95, 2.20)	1.66 (1.56, 1.77)	< 0.01
	Women	9,185	89% (8,134)	24.6	5,845	47% (2,768)	8.6	2.84 (2.70, 3.00)	2.02 (1.90, 2.15)	
Coeliac disease	Men	855	60% (515)	15.1	6,745	11% (759)	1.4	11.1 (8.12, 15.1)	7.46 (5.50, 10.1)	0.282
	Women	1,870	58% (1,091)	10.3	13,980	13% (1,756)	1.1	9.51 (7.85, 11.5)	6.18 (5.14, 7.44)	
CHD	Men	124,400	85% (105,184)	13.1	85,205	44% (37,889)	5.4	2.43 (2.40, 2.47)	2.17 (2.13, 2.20)	< 0.01
	Women	97,320	83% (81,106)	17.0	57,165	39% (22,307)	5.1	3.32 (3.26, 3.39)	2.75 (2.70, 2.80)	
Colon cancer	Men	9,670	93.6% (9,050)	22.6	19,570	85% (16,625)	13.4	1.68 (1.64, 1.73)	1.59 (1.54, 1.64)	< 0.01
	Women	9,445	94.4% (8,916)	23.4	15,835	85% (13,515)	12.6	1.85 (1.80, 1.91)	1.76 (1.71, 1.81)	
COPD	Men	57,970	83% (48,001)	18.9	91,545	27% (24,594)	3.8	4.94 (4.82, 5.06)	2.25 (2.19, 2.32)	< 0.01
	Women	58,100	83% (48,304)	19.6	79,290	25% (19,892)	3.3	5.99 (5.85, 6.13)	2.74 (2.66, 2.82)	
IBD	Men	7,665	75% (5,725)	15.1	20,945	20% (4,215)	2.7	5.68 (5.28, 6.11)	4.97 (4.62, 5.35)	0.890
	Women	9,325	76% (7,114)	16.0	22,705	22% (4,937)	2.7	6.02 (5.65, 6.42)	4.94 (4.63, 5.26)	
Lung cancer	Men	20,265	90.9% (18,423)	18.0	22,580	73% (16,407)	10.7	1.68 (1.64, 1.72)	1.54 (1.50, 1.58)	< 0.01
	Women	17,120	90.9% (15,569)	18.6	19,130	72% (13,702)	10.3	1.81 (1.76, 1.86)	1.67 (1.63, 1.73)	
Lyme disease	Men	365	29% (106)	4.6	1,815	5.4% (98)	0.6	8.03 (4.16, 15.5)	2.25 (1.07, 4.75)	0.828
	Women	415	27% (110)	2.5	2,585	7.2% (186)	0.4	5.68 (3.30, 9.77)	2.48 (1.42, 4.34)	
Multiple sclerosis	Men	955	68% (650)	15.9	2,625	16% (423)	2.0	7.78 (6.17, 9.80)	3.63 (2.84, 4.65)	0.891
	Women	1,890	66% (1,238)	13.8	5,835	15% (861)	1.5	9.00 (7.62, 10.6)	3.56 (2.98, 4.25)	
Ovarian cancer	Women	5,600	93.6% (5,239)	20.4	7,720	80% (6,183)	9.3	2.18 (2.09, 2.27)	2.02 (1.94, 2.11)	
Pancreatic cancer	Men	5,730	92.6% (5,304)	18.6	3,815	74% (2,837)	13.4	1.39 (1.32, 1.46)	1.29 (1.22, 1.36)	0.0282
	Women	5,535	92.8% (5,137)	19.5	3,420	72% (2,467)	12.7	1.53 (1.46, 1.61)	1.41 (1.33, 1.49)	
Parkinson's disease	Men	8,390	88% (7,416)	32.6	16,385	24% (3,990)	5.3	6.16 (5.78, 6.57)	2.62 (2.39, 2.86)	0.0337
	Women	6,040	88% (5,294)	30.4	10,005	24% (2,385)	5.0	6.02 (5.59, 6.49)	3.06 (2.73, 3.44)	
PCOS	Women	8,865	49% (4,315)	3.5	65,150	16% (10,402)	0.6	5.46 (5.09, 5.86)	2.16 (2.01, 2.31)	
Rheumatoid arthritis	Men	8,100	75% (6,036)	16.1	18,045	25% (4,580)	3.0	5.39 (5.04, 5.76)	3.25 (3.02, 3.48)	0.359
	Women	18,545	76% (14,071)	17.6	37,130	24% (8,999)	2.6	6.79 (6.50, 7.10)	3.12 (2.98, 3.27)	
SBE	Men	3,400	97.9% (3,330)	45.3	415	79% (329)	37.0	1.22 (1.11, 1.34)	1.19 (1.09, 1.31)	0.347
	Women	1,600	97.3% (1,557)	47.0	220	76% (167)	35.3	1.33 (1.16, 1.53)	1.29 (1.12, 1.50)	
Schizophrenia	Men	12,495	73% (9,169)	32.4	12,090	28% (3,395)	17.1	1.89 (1.77, 2.01)	1.32 (1.24, 1.41)	< 0.01
	Women	11,340	81% (9,162)	37.0	10,695	35% (3,787)	14.8	2.49 (2.35, 2.64)	1.78 (1.67, 1.90)	
Tuberculosis	Men	3,070	76% (2,329)	19.4	4,565	21% (939)	4.0	4.83 (4.30, 5.43)	3.16 (2.79, 3.58)	< 0.01
	Women	1,935	73% (1,413)	18.2	4,070	20% (812)	3.0	6.10 (5.25, 7.09)	4.28 (3.63, 5.04)	

Condition	Gender	Emergency diagnosed: N patients [a]	Emergency diagnosed: Admitted, % (n) [b]	Emergency diagnosed: Mean admitted duration	Non-emergency diagnosed: N patients [a]	Non-emergency diagnosed: Admitted, % (n) [b]	Non-emergency diagnosed: Mean admitted duration	Crude RR (95% CI)	Adjusted [c] RR (95% CI)	p-value [d]
GOLD										
Axial spondyloarthritis	Men	270	77% (209)	18.9	1,040	16% (168)	2.0	9.45 (6.22, 14.3)	3.70 (2.47, 5.54)	0.774
	Women	240	77% (185)	21.7	835	24% (201)	2.2	9.77 (6.65, 14.3)	4.01 (2.65, 6.09)	
Brain tumours	Men	1,590	92.7% (1,474)	25.8	690	62% (425)	11.8	2.18 (1.93, 2.46)	1.70 (1.48, 1.94)	0.149
	Women	1,970	91.5% (1,803)	26.3	1,200	53% (632)	9.5	2.76 (2.48, 3.08)	1.95 (1.71, 2.22)	
Coeliac disease	Men	180	66% (119)	14.9	1,550	12% (190)	1.4	10.5 (5.52, 20.1)	10.3 (5.28, 19.9)	0.150
	Women	365	61% (224)	10.9	2,975	12% (357)	1.1	9.85 (6.33, 15.3)	5.83 (3.82, 8.90)	
CHD	Men	28,045	86% (23,979)	13.1	18,850	46% (8,631)	5.5	2.37 (2.30, 2.45)	2.14 (2.07, 2.21)	< 0.01
	Women	21,930	84% (18,424)	16.8	12,295	40% (4,935)	5.5	3.08 (2.96, 3.20)	2.50 (2.40, 2.60)	
Colon cancer	Men	2,320	95.6% (2,219)	22.4	4,795	86% (4,109)	13.8	1.62 (1.53, 1.71)	1.55 (1.46, 1.64)	0.0424
	Women	2,255	94.7% (2,135)	23.8	4,005	86% (3,429)	13.3	1.79 (1.69, 1.90)	1.68 (1.59, 1.78)	
COPD	Men	12,635	84% (10,664)	19.6	16,330	31% (5,042)	4.6	4.28 (4.08, 4.49)	2.13 (2.00, 2.25)	< 0.01
	Women	12,655	85% (10,735)	19.9	14,650	28% (4,119)	3.8	5.24 (4.99, 5.50)	2.54 (2.40, 2.69)	
IBD	Men	1,715	78% (1,334)	16.5	4,810	21% (1,030)	3.0	5.56 (4.77, 6.47)	4.76 (4.07, 5.57)	0.301
	Women	2,070	78% (1,619)	16.6	5,545	22% (1,214)	2.6	6.37 (5.57, 7.28)	5.31 (4.64, 6.09)	
Lung cancer	Men	4,655	90.9% (4,232)	17.5	5,225	74% (3,868)	10.6	1.64 (1.56, 1.73)	1.55 (1.46, 1.63)	0.102
	Women	3,995	91.0% (3,634)	18.2	4,305	72% (3,111)	10.2	1.79 (1.69, 1.89)	1.65 (1.56, 1.76)	
Lyme disease	Men	40	40% (16)	9.7	265	8.7% (23)	0.9	10.7 (2.03, 56.4)	2.4 (0.188, 30.6)	0.891
	Women	40	35% (14)	3.6	280	6.8% (19)	0.8	4.52 (0.697, 29.4)	1.97 (0.324, 12.0)	
Multiple sclerosis	Men	200	74% (148)	19.0	565	16% (93)	2.3	8.31 (5.09, 13.6)	6.10 (3.62, 10.3)	0.143
	Women	435	69% (300)	14.9	1,370	17% (237)	1.9	7.92 (5.68, 11.1)	3.81 (2.73, 5.33)	
Ovarian cancer	Women	1,410	93.9% (1,324)	20.1	1,925	80% (1,533)	9.8	2.06 (1.90, 2.24)	1.93 (1.77, 2.10)	
Pancreatic cancer	Men	1,215	92.6% (1,125)	18.7	840	77% (646)	13.4	1.40 (1.26, 1.56)	1.29 (1.16, 1.44)	0.0407
	Women	1,355	94.5% (1,281)	20.5	855	73% (627)	12.7	1.62 (1.47, 1.79)	1.52 (1.36, 1.69)	
Parkinson's disease	Men	1,860	90% (1,670)	32.2	3,705	24% (900)	5.5	5.82 (5.08, 6.67)	2.62 (2.18, 3.14)	0.868
	Women	1,375	86% (1,189)	29.7	2,410	24% (583)	5.9	5.07 (4.32, 5.96)	2.68 (2.12, 3.39)	
PCOS	Women	1,700	53% (902)	4.0	12,045	16% (1,890)	0.6	6.16 (5.25, 7.22)	2.67 (2.27, 3.13)	
Rheumatoid arthritis	Men	1,760	77% (1,363)	17.5	4,010	24% (952)	2.7	6.49 (5.63, 7.48)	3.08 (2.62, 3.63)	0.924
	Women	4,250	79% (3,338)	19.3	8,595	24% (2,075)	2.7	7.20 (6.58, 7.88)	3.11 (2.82, 3.44)	
SBE	Men	660	97.2% (642)	45.5	80	76% (61)	31.3	1.45 (1.17, 1.8)	1.38 (1.11, 1.72)	0.0350
	Women	335	97.9% (328)	45.9	40	85% (34)	49.6	0.924 (0.677, 1.26)	0.920 (0.667, 1.27)	
Schizophrenia	Men	2,275	75% (1,697)	35.8	2,135	29% (623)	19.2	1.87 (1.61, 2.17)	1.34 (1.15, 1.57)	0.0159
	Women	2,195	83% (1,822)	39.8	1,855	37% (680)	15.1	2.64 (2.31, 3.00)	1.76 (1.51, 2.04)	
Tuberculosis	Men	545	79% (433)	22.3	710	22% (154)	4.9	4.50 (3.43, 5.92)	3.89 (2.93, 5.18)	0.0489
	Women	350	74% (259)	18.7	725	22% (163)	3.8	4.85 (3.43, 6.87)	2.38 (1.58, 3.59)	

[a] To prevent backwards calculation of small patient groups the number of patients has been reported rounded to 5.
[b] Percentage (number) of patients admitted for at least one night in the year after diagnosis.
[c] Adjusted for age at diagnosis, year of diagnosis, IMD score, comorbidity score six months (180 days) prior to diagnosis and diagnosis source. Note that p-value indicates difference between genders.
[d] Note that p-values relate to the null hypothesis that there was no difference in the adjusted RR between men and women tested using likelihood ratio tests.

Condition	Gender	Emergency diagnosed			Non-emergency diagnosed			Crude RR (95% CI)	Adjusted [c] RR (95% CI)	p-value [d]
		N patients [a]	Admitted, % (n) [b]	Mean admitted duration	N patients [a]	Admitted, % (n) [b]	Mean admitted duration			

CI, confidence interval; RR, rate ratio; CHD, coronary/ischaemic heart disease; COPD, chronic obstructive pulmonary disease; IBD, inflammatory bowel disease; PCOS, polycystic ovary syndrome; SBE, subacute bacterial endocarditis.

Sensitivity analysis 2: Adjusted hospitalisation risk

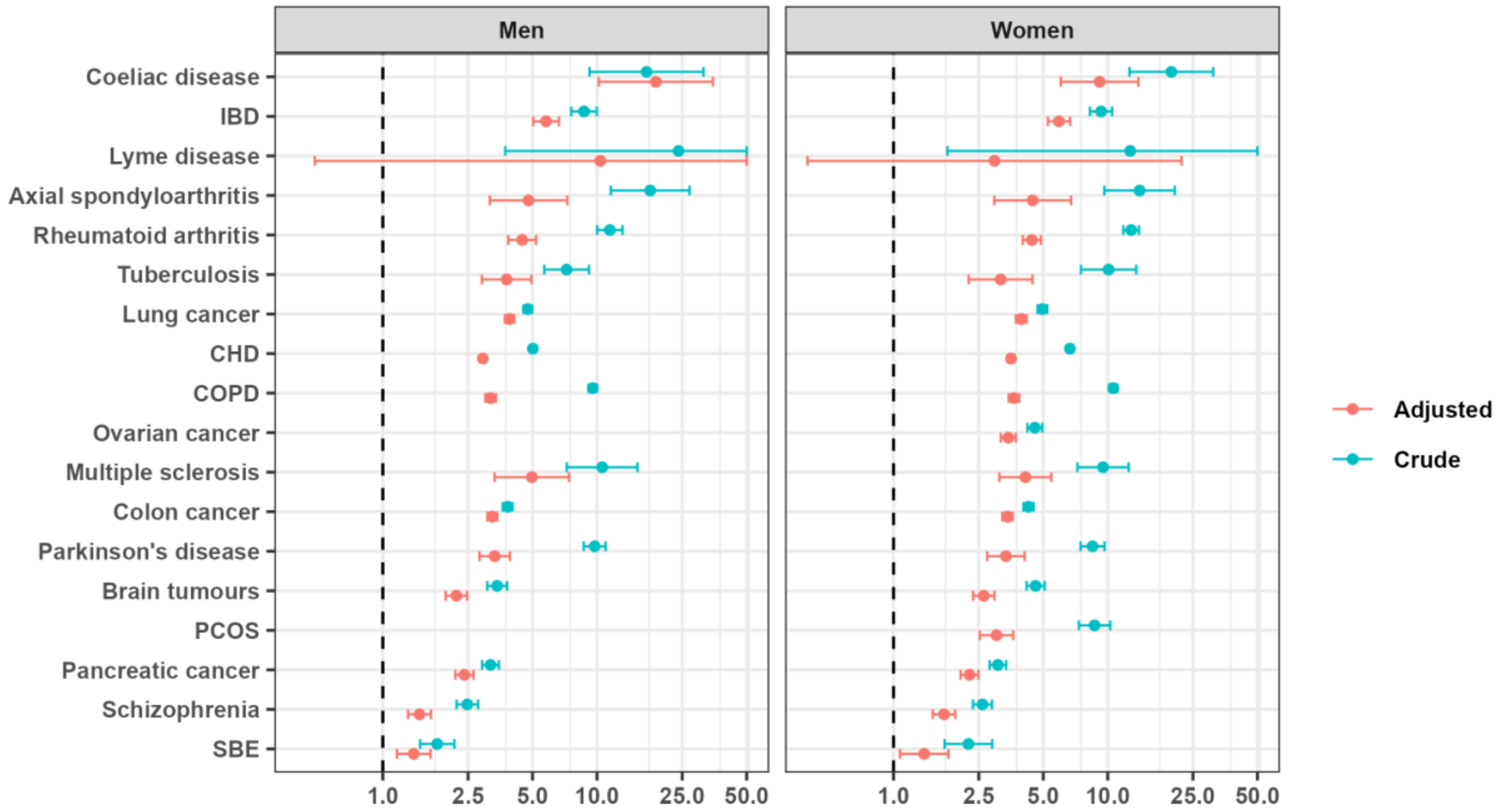

Figure 44. Forest plot of rate ratios for time in hospital whilst alive in the year post-diagnosis following an emergency diagnosis - GOLD. Rate ratios were adjusted for age at diagnosis, year of diagnosis, IMD score, comorbidity score six months (180 days) prior to diagnosis and diagnosis source and an offset for number of days alive in the year after diagnosis. N.B. Upper confidence intervals that exceed 50 have been reduced to 50 for visualisation purposes. CHD, coronary/ischaemic heart disease; COPD, chronic obstructive pulmonary disease; IBD, inflammatory bowel disease; PCOS, polycystic ovary syndrome; SBE, subacute bacterial endocarditis.

Table 46. Observed mean number of hospital inpatient nights whilst patients were alive in year after diagnosis and rate ratios (RRs) for duration of hospital inpatient nights in year after diagnosis in emergency diagnosed vs non-emergency diagnosed patients by condition, gender, and CPRD dataset.

Condition	Gender	ED			Non-ED			Crude RR (95% CI)	Adjusted [c] RR (95% CI)	p-value [d]
		N patients [a]	Admitted, % (n) [b]	Mean admitted duration	N patients [a]	Admitted, % (n) [b]	Mean admitted duration			
Aurum										
Axial spondyloarthritis	Men	1,265	78% (993)	21.9	5,600	16% (902)	2.4	17.8 (14.3, 22.1)	3.86 (3.05, 4.88)	0.0785
	Women	1,145	76% (869)	17.8	3,880	25% (973)	2.7	11.3 (9.36, 13.8)	5.03 (4.20, 6.03)	
Brain tumours	Men	8,845	93.6% (8,281)	27.3	4,620	62% (2,878)	13.8	3.38 (3.19, 3.58)	2.29 (2.15, 2.43)	0.0328
	Women	10,620	91.2% (9,689)	27.2	7,915	51% (4,063)	10.6	4.44 (4.21, 4.69)	2.51 (2.36, 2.67)	
Coeliac disease	Men	825	72% (596)	18.8	8,480	12% (1,025)	1.5	20.7 (15.0, 28.6)	11.0 (8.05, 15.0)	0.314
	Women	1,725	69% (1,193)	13.6	17,450	13% (2,248)	1.2	18.6 (15.1, 23.0)	9.17 (7.52, 11.2)	
CHD	Men	162,500	87% (140,818)	14.3	130,335	47% (61,077)	5.9	5.11 (5.03, 5.19)	3.21 (3.15, 3.26)	< 0.01
	Women	133,265	85% (113,712)	18.4	90,805	41% (37,238)	6.0	6.86 (6.74, 6.98)	4.08 (4.00, 4.16)	
Colon cancer	Men	12,435	94.7% (11,773)	24.2	26,500	85% (22,537)	14.4	4.10 (3.98, 4.22)	3.52 (3.41, 3.63)	0.0551
	Women	12,340	95.2% (11,743)	25.4	21,915	85% (18,737)	14.1	4.42 (4.29, 4.55)	3.67 (3.56, 3.79)	
COPD	Men	71,255	86% (61,566)	20.4	128,515	28% (35,489)	4.2	10.9 (10.6, 11.1)	3.61 (3.50, 3.72)	< 0.01
	Women	72,340	87% (62,651)	21.1	110,340	26% (28,437)	3.7	12.1 (11.8, 12.4)	4.04 (3.92, 4.16)	
IBD	Men	8,555	82% (7,029)	17.9	27,675	21% (5,942)	3.0	9.79 (9.10, 10.5)	6.89 (6.41, 7.40)	0.121
	Women	10,450	83% (8,719)	18.9	29,875	23% (6,974)	3.1	9.98 (9.37, 10.6)	6.39 (6.00, 6.81)	
Lung cancer	Men	25,735	92.2% (23,740)	19.3	31,990	73% (23,317)	11.3	4.93 (4.81, 5.05)	4.02 (3.91, 4.14)	< 0.01
	Women	20,980	92.6% (19,437)	20.1	25,760	72% (18,490)	11.0	5.30 (5.16, 5.45)	4.32 (4.18, 4.46)	
Lyme disease	Men	200	52% (104)	8.1	2,225	5.8% (128)	0.7	10.0 (4.03, 24.9)	4.53 (1.60, 12.8)	0.678
	Women	195	52% (101)	6.9	3,060	7.7% (235)	0.5	16.2 (7.87, 33.2)	5.87 (2.65, 13.0)	
Multiple sclerosis	Men	1,060	76% (808)	19.0	3,630	19% (690)	2.8	10.7 (8.59, 13.4)	4.63 (3.62, 5.92)	0.155
	Women	2,120	74% (1,566)	16.7	8,245	18% (1,505)	2.3	11.5 (9.78, 13.4)	3.73 (3.15, 4.41)	
Ovarian cancer	Women	7,435	94.6% (7,036)	22.3	11,120	81% (9,021)	10.7	5.28 (5.05, 5.52)	3.91 (3.74, 4.09)	
Pancreatic cancer	Men	7,125	93.5% (6,660)	19.7	5,275	76% (4,004)	14.2	3.23 (3.08, 3.39)	2.46 (2.34, 2.60)	0.296
	Women	6,995	93.8% (6,559)	20.9	4,825	73% (3,534)	13.7	3.36 (3.20, 3.54)	2.36 (2.23, 2.50)	
Parkinson's disease	Men	10,485	91.5% (9,599)	35.9	22,345	26% (5,703)	6.0	11.0 (10.3, 11.7)	3.17 (2.90, 3.47)	0.119
	Women	7,860	90.6% (7,124)	34.2	14,390	26% (3,693)	6.3	9.46 (8.83, 10.1)	3.55 (3.19, 3.95)	
PCOS	Women	7,120	59% (4,204)	4.5	81,530	16% (12,956)	0.7	7.26 (6.70, 7.86)	2.39 (2.20, 2.59)	
Rheumatoid arthritis	Men	8,840	80% (7,043)	18.2	23,715	26% (6,278)	3.3	9.95 (9.30, 10.7)	4.88 (4.53, 5.25)	0.0754
	Women	21,060	81% (17,027)	20.3	49,260	25% (12,465)	3.0	12.1 (11.6, 12.6)	4.51 (4.29, 4.73)	
SBE	Men	4,100	98.2% (4,026)	45.6	730	80% (587)	38.0	1.56 (1.42, 1.70)	1.22 (1.12, 1.34)	0.182
	Women	1,980	97.0% (1,921)	47.5	385	77% (297)	37.2	1.79 (1.57, 2.04)	1.36 (1.19, 1.55)	
Schizophrenia	Men	13,230	82% (10,786)	40.6	18,150	31% (5,675)	19.3	2.55 (2.41, 2.70)	1.40 (1.32, 1.49)	< 0.01
	Women	12,425	87% (10,826)	44.2	16,115	38% (6,075)	17.5	3.06 (2.90, 3.22)	1.82 (1.72, 1.94)	
Tuberculosis	Men	3,475	85% (2,941)	22.6	6,225	23% (1,441)	4.8	7.49 (6.68, 8.40)	3.54 (3.13, 4.01)	0.0304
	Women	2,170	85% (1,840)	22.2	5,560	22% (1,247)	3.5	9.55 (8.29, 11.0)	4.41 (3.75, 5.19)	

Condition	Gender	ED			Non-ED			Crude RR (95% CI)	Adjusted [c] RR (95% CI)	p-value [d]
		N patients [a]	Admitted, % (n) [b]	Mean admitted duration	N patients [a]	Admitted, % (n) [b]	Mean admitted duration			
GOLD										
Axial spondyloarthritis	Men	325	85% (277)	21.1	1,800	16% (289)	2.1	17.7 (11.6, 27.0)	4.79 (3.16, 7.27)	0.822
	Women	305	85% (258)	22.9	1,435	23% (331)	2.9	14.1 (9.64, 20.6)	4.46 (2.95, 6.75)	
Brain tumours	Men	2,490	94.6% (2,355)	28.6	1,255	64% (807)	14.0	3.42 (3.08, 3.80)	2.20 (1.96, 2.47)	0.0287
	Women	2,860	93.9% (2,686)	29.5	1,990	58% (1,151)	12.3	4.61 (4.18, 5.08)	2.64 (2.35, 2.96)	
Coeliac disease	Men	220	79% (174)	21.2	2,400	13% (309)	1.6	17.0 (9.23, 31.4)	18.8 (10.2, 34.8)	0.0523
	Women	400	72% (289)	16.4	4,570	12% (571)	1.3	19.8 (12.6, 31.1)	9.16 (6.04, 13.9)	
CHD	Men	47,795	87% (41,781)	14.3	38,225	47% (17,790)	5.9	5.02 (4.88, 5.16)	2.93 (2.84, 3.02)	< 0.01
	Women	38,970	86% (33,550)	18.4	27,485	40% (10,902)	6.1	6.66 (6.44, 6.88)	3.54 (3.41, 3.67)	
Colon cancer	Men	3,845	96.2% (3,698)	24.5	8,165	86% (7,008)	15.3	3.83 (3.63, 4.03)	3.25 (3.08, 3.42)	0.220
	Women	3,830	95.6% (3,661)	26.0	6,955	85% (5,900)	15.0	4.27 (4.04, 4.50)	3.40 (3.22, 3.60)	
COPD	Men	19,795	87% (17,318)	20.7	33,015	30% (9,936)	4.7	9.55 (9.14, 9.99)	3.19 (3.01, 3.37)	< 0.01
	Women	20,080	88% (17,666)	21.4	28,800	28% (7,930)	4.2	10.6 (10.2, 11.1)	3.65 (3.45, 3.86)	
IBD	Men	2,395	85% (2,039)	19.7	8,160	23% (1,870)	3.5	8.70 (7.59, 9.98)	5.79 (5.04, 6.64)	0.814
	Women	3,040	84% (2,552)	19.5	9,255	24% (2,184)	3.2	9.31 (8.27, 10.5)	5.92 (5.26, 6.67)	
Lung cancer	Men	7,800	92.3% (7,198)	19.2	9,585	74% (7,058)	11.5	4.75 (4.54, 4.97)	3.91 (3.72, 4.10)	0.781
	Women	6,155	93.0% (5,718)	20.3	7,290	72% (5,252)	11.5	4.95 (4.70, 5.21)	3.95 (3.72, 4.18)	
Lyme disease	Men	30	57% (17)	16.4	370	9.6% (35)	0.9	24.0 (3.73, 155)	10.4 (0.482, 223)	0.534
	Women	30	67% (20)	8.8	430	7.7% (33)	0.9	12.7 (1.79, 90.6)	2.96 (0.397, 22.1)	
Multiple sclerosis	Men	320	79% (252)	22.0	1,020	20% (205)	2.8	10.6 (7.22, 15.5)	4.96 (3.33, 7.41)	0.462
	Women	620	77% (479)	18.9	2,450	21% (515)	2.6	9.51 (7.22, 12.5)	4.13 (3.12, 5.46)	
Ovarian cancer	Women	2,420	95.2% (2,304)	23.2	3,525	79% (2,791)	11.2	4.57 (4.22, 4.95)	3.43 (3.17, 3.72)	
Pancreatic cancer	Men	2,015	93.9% (1,892)	20.3	1,430	77% (1,108)	14.5	3.18 (2.91, 3.48)	2.41 (2.18, 2.65)	0.385
	Women	2,140	95.3% (2,040)	21.8	1,455	74% (1,075)	14.4	3.07 (2.81, 3.36)	2.26 (2.06, 2.49)	
Parkinson's disease	Men	2,935	92.4% (2,713)	36.2	6,760	26% (1,745)	6.8	9.75 (8.68, 11.0)	3.33 (2.83, 3.92)	0.973
	Women	2,325	90.7% (2,109)	35.3	4,600	26% (1,210)	7.3	8.49 (7.47, 9.66)	3.35 (2.74, 4.09)	
PCOS	Women	1,600	63% (1,002)	5.2	18,835	15% (2,879)	0.7	8.68 (7.33, 10.3)	3.02 (2.53, 3.62)	
Rheumatoid arthritis	Men	2,290	82% (1,875)	20.1	6,900	25% (1,747)	3.2	11.5 (10.0, 13.2)	4.47 (3.85, 5.20)	0.903
	Women	5,690	83% (4,727)	22.0	14,690	25% (3,612)	3.2	12.9 (11.8, 14.0)	4.42 (4.01, 4.88)	
SBE	Men	1,040	97.2% (1,011)	45.0	200	74% (147)	28.2	1.79 (1.49, 2.16)	1.39 (1.16, 1.67)	0.990
	Women	530	98.5% (522)	45.4	100	72% (72)	35.2	2.24 (1.73, 2.89)	1.39 (1.07, 1.80)	
Schizophrenia	Men	3,165	83% (2,620)	45.9	4,355	33% (1,435)	20.9	2.48 (2.21, 2.78)	1.48 (1.31, 1.68)	0.0937
	Women	3,140	89% (2,785)	47.9	3,995	41% (1,628)	20.6	2.60 (2.35, 2.88)	1.72 (1.52, 1.94)	
Tuberculosis	Men	750	86% (646)	25.1	1,205	26% (318)	5.8	7.21 (5.67, 9.17)	3.79 (2.90, 4.94)	0.414
	Women	485	86% (418)	25.0	1,240	25% (314)	4.0	10.1 (7.5, 13.6)	3.16 (2.25, 4.45)	

[a] To prevent backwards calculation of small patient groups the number of patients has been reported rounded to 5.
[b] Percentage (number) of patients admitted for at least one night in the year after diagnosis.
[c] Adjusted for age at diagnosis, year of diagnosis, IMD score, comorbidity score six months (180 days) prior to diagnosis, diagnosis source and an offset for number of days alive in the year after diagnosis
[d] Note that p-values relate to the null hypothesis that there was no difference in the adjusted RR between men and women tested using likelihood ratio tests.

Condition	Gender	ED			Non-ED			Crude RR (95% CI)	Adjusted [c] RR (95% CI)	p-value [d]
		N patients [a]	Admitted, % (n) [b]	Mean admitted duration	N patients [a]	Admitted, % (n) [b]	Mean admitted duration			

CI, confidence interval; RR, rate ratio; CHD, coronary/ischaemic heart disease; COPD, chronic obstructive pulmonary disease; IBD, inflammatory bowel disease; PCOS, polycystic ovary syndrome; SBE, subacute bacterial endocarditis.

Sensitivity Analysis 3: COVID-19 impact

Table 47. Observed 1-year mortality and odds ratios (ORs) by emergency diagnosis status (emergency hospitalisation), condition, gender, and CPRD dataset excluding patients diagnosed in 2019.

Condition	Gender	Emergency diagnosed		Non-emergency diagnosed		Crude OR (95% CI)	Adjusted [b] OR (95% CI)	p-value [c]
		N patients [a]	1-year mortality, % (n)	N patients [a]	1-year mortality, % (n)			
Aurum								
Axial spondyloarthritis	Men	1,145	21% (237)	5,175	-			
	Women	1,025	14% (146)	3,460	-			
Brain tumours	Men	8,180	56% (4,585)	4,285	25% (1,063)	3.87 (3.56, 4.20)	3.23 (2.91, 3.59)	< 0.01
	Women	9,675	46% (4,478)	7,210	14% (987)	5.43 (5.03, 5.88)	4.00 (3.61, 4.44)	
Coeliac disease	Men	750	-	7,785	1.6% (125)			
	Women	1,510	11% (164)	15,880	-			
CHD	Men	151,985	20% (29,662)	122,140	4.9% (6,019)	4.68 (4.55, 4.82)	3.35 (3.24, 3.46)	< 0.01
	Women	125,690	27% (33,548)	85,355	5.1% (4,365)	6.76 (6.54, 6.98)	3.96 (3.82, 4.12)	
Colon cancer	Men	11,655	51% (5,929)	24,695	19% (4,692)	4.41 (4.20, 4.63)	4.38 (4.16, 4.61)	0.0624
	Women	11,580	53% (6,168)	20,415	19% (3,867)	4.88 (4.64, 5.13)	4.71 (4.45, 4.97)	
COPD	Men	65,920	34% (22,160)	120,250	6.3% (7,555)	7.55 (7.34, 7.77)	2.95 (2.84, 3.07)	< 0.01
	Women	67,300	30% (19,898)	102,930	4.6% (4,750)	8.68 (8.39, 8.97)	3.51 (3.35, 3.68)	
IBD	Men	7,835	13% (1,024)	25,355	1.7% (436)	8.59 (7.67, 9.65)	5.92 (5.21, 6.73)	0.0458
	Women	9,565	13% (1,285)	27,395	1.6% (437)	9.57 (8.57, 10.7)	4.94 (4.36, 5.59)	
Lung cancer	Men	24,200	85% (20,578)	29,990	54% (16,307)	4.77 (4.57, 4.97)	4.70 (4.50, 4.92)	0.142
	Women	19,650	82% (16,061)	23,900	47% (11,207)	5.07 (4.85, 5.30)	4.94 (4.71, 5.19)	
Lyme disease	Men	165	-	1,790	-			
	Women	165	-	2,385	-			
Multiple sclerosis	Men	970	14% (140)	3,380	-			
	Women	1,935	10% (203)	7,690	-			
Ovarian cancer	Women	7,000	53% (3,714)	10,460	18% (1,905)	5.08 (4.74, 5.44)	4.41 (4.09, 4.76)	
Pancreatic cancer	Men	6,610	83% (5,515)	4,895	65% (3,192)	2.68 (2.46, 2.93)	2.59 (2.34, 2.85)	0.39
	Women	6,515	85% (5,548)	4,490	65% (2,922)	3.08 (2.81, 3.38)	2.75 (2.48, 3.06)	
Parkinson's disease	Men	9,750	40% (3,886)	20,760	6.5% (1,358)	9.46 (8.84, 10.1)	2.82 (2.52, 3.17)	0.712
	Women	7,365	30% (2,244)	13,460	5.3% (710)	7.87 (7.19, 8.62)	2.72 (2.33, 3.19)	
PCOS	Women	6,325	-	74,455	-			
Rheumatoid arthritis	Men	8,050	20% (1,629)	21,880	3.2% (690)	7.79 (7.10, 8.56)	3.56 (3.17, 4.01)	0.118
	Women	19,345	20% (3,864)	45,615	2.1% (955)	11.7 (10.9, 12.6)	4.02 (3.66, 4.42)	
SBE	Men	3,745	31% (1,153)	675	22% (146)	1.62 (1.33, 1.97)	1.02 (0.812, 1.28)	
	Women	1,800	40% (711)	360	-			
Schizophrenia	Men	12,165	11% (1,279)	16,715	3.2% (532)	3.57 (3.22, 3.97)	1.79 (1.54, 2.10)	0.499
	Women	11,370	13% (1,472)	14,885	4.8% (711)	2.97 (2.70, 3.26)	1.67 (1.44, 1.93)	

Condition	Gender	Emergency diagnosed		Non-emergency diagnosed		Crude OR (95% CI)	Adjusted [b] OR (95% CI)	p-value [c]
		N patients [a]	1-year mortality, % (n)	N patients [a]	1-year mortality, % (n)			
Tuberculosis	Men	3,300	18% (578)	5,860	4.2% (244)	4.89 (4.19, 5.72)	3.04 (2.44, 3.79)	
	Women	2,080	-	5,255	-			
GOLD								
Axial spondyloarthritis	Men	-	-	1,775	1.7% (31)			
	Women	295	-	1,405	1.6% (22)			
Brain tumours	Men	2,435	62% (1,503)	1,225	-			
	Women	2,795	50% (1,403)	1,955	-			
Coeliac disease	Men	-	-	2,350	2.0% (48)			
	Women	390	12% (47)	4,475	0.94% (42)	14.5 (9.40, 22.3)	5.41 (3.17, 9.33)	
CHD	Men	47,110	20% (9,340)	37,710	5.6% (2,111)	4.17 (3.97, 4.38)	2.91 (2.74, 3.09)	
	Women	38,465	28% (10,580)	27,160	-			
Colon cancer	Men	3,785	50% (1,885)	8,045	20% (1,598)	4.00 (3.68, 4.36)	3.88 (3.54, 4.24)	0.0997
	Women	3,775	53% (2,010)	6,860	19% (1,335)	4.71 (4.32, 5.15)	4.33 (3.94, 4.76)	
COPD	Men	19,400	35% (6,869)	32,610	7.7% (2,506)	6.59 (6.26, 6.93)	2.83 (2.64, 3.04)	0.513
	Women	19,715	31% (6,049)	28,375	5.8% (1,659)	7.13 (6.73, 7.56)	2.93 (2.70, 3.19)	
IBD	Men	2,345	-	8,025	-			
	Women	2,980	-	9,070	-			
Lung cancer	Men	7,710	87% (6,679)	9,435	58% (5,516)	4.61 (4.27, 4.98)	4.50 (4.15, 4.89)	0.107
	Women	6,045	84% (5,094)	7,160	51% (3,654)	5.14 (4.73, 5.59)	4.98 (4.55, 5.45)	
Lyme disease	Men	-	-	360	-			
	Women	-	-	-	-			
Multiple sclerosis	Men	-	-	1,000	1.3% (13)			
	Women	605	-	2,410	1.3% (31)			
Ovarian cancer	Women	2,380	52% (1,226)	3,480	-			
Pancreatic cancer	Men	1,990	84% (1,681)	1,410	66% (935)	2.75 (2.33, 3.24)	2.78 (2.32, 3.34)	0.38
	Women	2,115	86% (1,814)	1,430	68% (969)	2.87 (2.44, 3.39)	2.48 (2.07, 2.97)	
Parkinson's disease	Men	2,880	41% (1,179)	6,670	-			
	Women	2,285	33% (750)	4,540	-			
PCOS	Women	1,530	0.78% (12)	18,400	-			
Rheumatoid arthritis	Men	2,220	22% (485)	6,800	-			
	Women	5,550	21% (1,183)	14,475	-			
SBE	Men	1,010	-	-	-			
	Women	520	-	-	-			
Schizophrenia	Men	3,085	-	4,290	3.9% (169)			
	Women	3,080	-	3,935	-			
Tuberculosis	Men	735	-	1,185	-			
	Women	475	-	1,225	3.8% (46)			

[a] To prevent backwards calculation of small patient groups the number of patients has been reported rounded to 5.
[b] Adjusted for age at diagnosis, year of diagnosis, IMD score, comorbidity score six months (180 days) prior to diagnosis and diagnosis source. Note that p-value indicates difference between genders.
[c] Note that p-values relate to the null hypothesis that there was no difference in the adjusted OR between men and women tested using likelihood ratio tests.

Condition	Gender	Emergency diagnosed		Non-emergency diagnosed		Crude OR (95% CI)	Adjusted [b] OR (95% CI)	p-value [c]
		N patients [a]	1-year mortality, % (n)	N patients [a]	1-year mortality, % (n)			

"–" indicates that fewer than 10 patients died within 1 year of diagnosis, or that results have not been calculated to prevent backwards calculation of small patient groups. Odds ratios were not calculated for these conditions as the results are unlikely to be reliable.
CI, confidence interval; OR, odds ratio; CHD, coronary/ischaemic heart disease; COPD, chronic obstructive pulmonary disease; IBD, inflammatory bowel disease; PCOS, polycystic ovary syndrome; SBE, subacute bacterial endocarditis.

Table 48. Observed mean number of hospital inpatient nights whilst patients were alive in year after diagnosis and rate ratios (RRs) for duration of hospital inpatient nights in year after diagnosis in emergency diagnosed vs non-emergency diagnosed patients by condition, gender, and CPRD dataset excluding patients diagnosed in 2019.

Condition	Gender	ED			Non-ED			Crude RR (95% CI)	Adjusted [c] RR (95% CI)	p-value [d]
		N patients [a]	Admitted, % (n) [b]	Mean admitted duration	N patients [a]	Admitted, % (n) [b]	Mean admitted duration			
Aurum										
Axial spondyloarthritis	Men	1,145	79% (906)	22.4	5,175	17% (855)	2.5	8.94 (7.23, 11.1)	2.78 (2.20, 3.52)	0.0137
	Women	1,025	76% (782)	18.7	3,460	25% (866)	2.7	6.83 (5.66, 8.24)	4.00 (3.35, 4.78)	
Brain tumours	Men	8,180	93.9% (7,682)	27.6	4,285	63% (2,692)	14.0	1.97 (1.88, 2.08)	1.57 (1.48, 1.66)	< 0.01
	Women	9,675	91.7% (8,870)	27.7	7,210	52% (3,772)	11.1	2.49 (2.37, 2.61)	1.80 (1.70, 1.90)	
Coeliac disease	Men	750	72% (541)	19.4	7,785	12% (962)	1.6	12.5 (9.07, 17.2)	9.42 (6.92, 12.8)	0.0469
	Women	1,510	71% (1,066)	14.0	15,880	13% (2,044)	1.3	11.2 (9.00, 13.8)	6.53 (5.31, 8.02)	
CHD	Men	151,985	87% (131,919)	14.4	122,140	47% (57,911)	5.9	2.43 (2.40, 2.46)	2.07 (2.04, 2.10)	< 0.01
	Women	125,690	86% (107,495)	18.7	85,355	42% (35,444)	6.1	3.06 (3.02, 3.11)	2.47 (2.43, 2.51)	
Colon cancer	Men	11,655	94.8% (11,051)	24.4	24,695	85% (21,051)	14.7	1.67 (1.62, 1.71)	1.56 (1.52, 1.60)	< 0.01
	Women	11,580	95.3% (11,034)	25.9	20,415	86% (17,478)	14.4	1.79 (1.75, 1.84)	1.69 (1.65, 1.74)	
COPD	Men	65,920	87% (57,169)	20.6	120,250	28% (33,434)	4.2	4.86 (4.76, 4.97)	2.15 (2.09, 2.20)	< 0.01
	Women	67,300	87% (58,517)	21.4	102,930	26% (26,718)	3.8	5.64 (5.52, 5.76)	2.49 (2.42, 2.55)	
IBD	Men	7,835	83% (6,480)	18.2	25,355	22% (5,550)	3.1	5.91 (5.51, 6.33)	5.04 (4.70, 5.41)	0.213
	Women	9,565	84% (8,036)	19.3	27,395	24% (6,499)	3.2	5.96 (5.61, 6.34)	4.75 (4.47, 5.05)	
Lung cancer	Men	24,200	92.4% (22,352)	19.5	29,990	73% (21,918)	11.4	1.71 (1.67, 1.75)	1.56 (1.53, 1.60)	< 0.01
	Women	19,650	92.9% (18,252)	20.3	23,900	72% (17,198)	11.1	1.83 (1.79, 1.88)	1.68 (1.63, 1.72)	
Lyme disease	Men	165	53% (88)	6.8	1,790	6.2% (111)	0.7	9.17 (3.75, 22.4)	3.07 (1.10, 8.53)	0.285
	Women	165	55% (90)	7.8	2,385	8.3% (198)	0.5	14.6 (6.89, 31.0)	6.16 (2.70, 14.0)	
Multiple sclerosis	Men	970	76% (742)	19.5	3,380	19% (658)	2.9	6.75 (5.42, 8.40)	3.78 (2.96, 4.84)	0.162
	Women	1,935	74% (1,440)	17.1	7,690	19% (1431)	2.4	7.09 (6.06, 8.30)	3.06 (2.59, 3.61)	
Ovarian cancer	Women	7,000	94.8% (6,630)	22.6	10,460	81% (8515)	10.9	2.07 (2.00, 2.15)	1.90 (1.83, 1.97)	
Pancreatic cancer	Men	6,610	93.7% (6,191)	20.0	4,895	76% (3,738)	14.5	1.38 (1.32, 1.44)	1.27 (1.21, 1.33)	0.0359
	Women	6,515	94.0% (6,124)	21.0	4,490	74% (3,303)	13.9	1.51 (1.44, 1.58)	1.37 (1.30, 1.44)	
Parkinson's disease	Men	9,750	91.8% (8,949)	36.4	20,760	26% (5,369)	6.2	5.85 (5.52, 6.20)	2.38 (2.18, 2.59)	0.0492
	Women	7,365	90.8% (6,689)	34.9	13,460	26% (3,505)	6.5	5.36 (5.01, 5.73)	2.72 (2.46, 3.01)	
PCOS	Women	6,325	60% (3,787)	4.6	74,455	16% (11,781)	0.7	6.87 (6.32, 7.47)	2.45 (2.25, 2.68)	

Condition	Gender	ED: N patients [a]	ED: Admitted, % (n) [b]	ED: Mean admitted duration	Non-ED: N patients [a]	Non-ED: Admitted, % (n) [b]	Non-ED: Mean admitted duration	Crude RR (95% CI)	Adjusted [c] RR (95% CI)	p-value [d]
Rheumatoid arthritis	Men	8,050	80% (6,459)	18.5	21,880	27% (5,818)	3.4	5.51 (5.16, 5.88)	3.07 (2.87, 3.30)	0.74
	Women	19,345	81% (15,740)	20.8	45,615	25% (11,612)	3.1	6.72 (6.45, 7.01)	3.03 (2.89, 3.17)	
SBE	Men	3,745	98.2% (3,677)	46.0	675	81% (550)	38.7	1.19 (1.10, 1.28)	1.14 (1.05, 1.24)	0.503
	Women	1,800	97.1% (1,747)	47.9	360	78% (279)	38.0	1.26 (1.12, 1.42)	1.20 (1.06, 1.35)	
Schizophrenia	Men	12,165	82% (9,966)	41.5	16,715	32% (5,349)	20.2	2.05 (1.94, 2.18)	1.28 (1.20, 1.36)	< 0.01
	Women	11,370	87% (9,945)	45.4	14,885	38% (5,697)	18.2	2.50 (2.37, 2.63)	1.65 (1.55, 1.76)	
Tuberculosis	Men	3,300	85% (2,802)	22.4	5,860	23% (1,375)	4.8	4.69 (4.23, 5.21)	3.01 (2.68, 3.39)	< 0.01
	Women	2,080	85% (1,767)	22.3	5,255	23% (1,186)	3.6	6.20 (5.43, 7.07)	4.01 (3.44, 4.68)	
GOLD										
Axial spondyloarthritis	Men	-	-		1,775	-				
	Women	295	-		1,405	-				
Brain tumours	Men	2,435	94.7% (2,305)	28.8	1,225	65% (794)	14.2	2.02 (1.85, 2.21)	1.57 (1.41, 1.73)	0.15
	Women	2,795	94.0% (2,628)	29.6	1,955	58% (1,134)	12.4	2.38 (2.19, 2.59)	1.74 (1.57, 1.94)	
Coeliac disease	Men	-	-		2,350	-				
	Women	390	-		4,475	13% (559)	1.3			
CHD	Men	47,110	87% (41,190)	14.3	37,710	47% (17,592)	6.0	2.40 (2.35, 2.46)	1.96 (1.91, 2.02)	< 0.01
	Women	38,465	86% (33,126)	18.5	27,160	40% (10,803)	6.1	3.02 (2.93, 3.10)	2.29 (2.21, 2.36)	
Colon cancer	Men	3,785	96.2% (3,643)	24.6	8,045	86% (6,902)	15.4	1.60 (1.53, 1.67)	1.49 (1.43, 1.56)	0.0404
	Women	3,775	95.5% (3,605)	26.1	6,860	85% (5,825)	15.1	1.73 (1.66, 1.81)	1.59 (1.52, 1.66)	
COPD	Men	19,400	88% (16,991)	20.8	32,610	30% (9,804)	4.7	4.42 (4.26, 4.60)	2.02 (1.92, 2.12)	< 0.01
	Women	19,715	88% (17,371)	21.5	28,375	28% (7,806)	4.2	5.07 (4.88, 5.27)	2.31 (2.20, 2.43)	
IBD	Men	2,345	85% (1,997)	19.7	8,025	23% (1,845)	3.5	5.60 (4.93, 6.36)	4.50 (3.95, 5.13)	0.722
	Women	2,980	84% (2,503)	19.6	9,070	24% (2,154)	3.3	5.96 (5.35, 6.65)	4.64 (4.15, 5.19)	
Lung cancer	Men	7,710	92.3% (7,118)	19.2	9,435	74% (6,962)	11.5	1.67 (1.60, 1.73)	1.55 (1.49, 1.62)	0.0992
	Women	6,045	93.1% (5,629)	20.4	7,160	72% (5,162)	11.4	1.78 (1.71, 1.86)	1.64 (1.56, 1.72)	
Lyme disease	Men	-	-		360	9.9% (35)	0.9			
	Women	-	-		-					
Multiple sclerosis	Men	-	-		1,000	-				
	Women	605	77% (467)	19.2	2,410	-				
Ovarian cancer	Women	2,380	95.4% (2,271)	23.3	3,480	79% (2,752)	11.2	2.08 (1.95, 2.21)	1.87 (1.75, 1.99)	
Pancreatic cancer	Men	1,990	94.0% (1,870)	20.3	1,410	78% (1,093)	14.6	1.39 (1.28, 1.50)	1.25 (1.15, 1.36)	0.0508
	Women	2,115	95.3% (2,016)	21.9	1,430	74% (1,059)	14.5	1.51 (1.40, 1.63)	1.40 (1.29, 1.53)	
Parkinson's disease	Men	2,880	92.5% (2,663)	36.4	6,670	26% (1,724)	6.9	5.28 (4.73, 5.89)	2.37 (2.03, 2.76)	0.666
	Women	2,285	90.8% (2,074)	35.5	4,540	26% (1,198)	7.4	4.83 (4.27, 5.46)	2.49 (2.09, 2.98)	
PCOS	Women	1,530	63% (965)	5.0	18,400	15% (2,770)	0.7	7.61 (6.41, 9.03)	2.80 (2.33, 3.36)	
Rheumatoid arthritis	Men	2,220	82% (1,820)	20.2	6,800	25% (1,721)	3.2	6.23 (5.50, 7.06)	3.00 (2.60, 3.46)	0.646
	Women	5,550	83% (4,613)	22.2	14,475	25% (3,564)	3.2	6.82 (6.31, 7.38)	2.89 (2.64, 3.16)	

Condition	Gender	ED			Non-ED			Crude RR (95% CI)	Adjusted [c] RR (95% CI)	p-value [d]
		N patients [a]	Admitted, % (n) [b]	Mean admitted duration	N patients [a]	Admitted, % (n) [b]	Mean admitted duration			
SBE	Men	1,010	97.0% (980)	45.2	-	-				
	Women	520	-		-	-				
Schizophrenia	Men	3,085	83% (2,564)	46.7	4,290	33% (1,422)	21.0	2.22 (1.98, 2.49)	1.41 (1.25, 1.60)	0.115
	Women	3,080	89% (2,734)	48.4	3,935	41% (1,608)	20.8	2.33 (2.11, 2.58)	1.62 (1.44, 1.82)	
Tuberculosis	Men	735	86% (633)	25.1	1,185	-				
	Women	475	-		1,225	-				

[a] To prevent backwards calculation of small patient groups the number of patients has been reported rounded to 5.

[b] Percentage (number) of patients admitted for at least one night in the year after diagnosis.

[c] Adjusted for age at diagnosis, year of diagnosis, IMD score, comorbidity score six months (180 days) prior to diagnosis and diagnosis source.

[d] Note that p-values relate to the null hypothesis that there was no difference in the adjusted RR between men and women tested using likelihood ratio tests.

"–" indicates that results have not been calculated to prevent backwards calculation of small patient groups. CI, confidence interval; RR, rate ratio; CHD, coronary/ischaemic heart disease; COPD, chronic obstructive pulmonary disease; IBD, inflammatory bowel disease; PCOS, polycystic ovary syndrome; SBE, subacute bacterial endocarditis.
