# Supplementary material for: Frequency and prognostic outcomes of emergency diagnosis in 13 non-neoplastic conditions in England: A population-based cohort study using linked electronic health records of 1.7 million patients
Source: PLoS Med. 2026 Aug 25;23(8):e1005182. doi: 10.1371/journal.pmed.1005182 (PMC13505939; doi:10.1371/journal.pmed.1005182)
Supplement: Supplementary file 3 [file pmed.1005182.s003.pdf]

Frequency and prognostic outcomes of emergency diagnosis in 13 non-neoplastic conditions in England: a population-based cohort study using linked electronic health records of 1.7 million patients – S1 File

Data extract protocol

Whitfield E et al.

**CPRD Protocol number**
22_002264
**Study Title**
Evaluating diagnostic window presence and length for 'hard-to-suspect' conditions using linked longitudinal health records
**Outcomes to be Measured**
Pre-diagnostic healthcare utilisation; Diagnostic window presence; Diagnostic window length; Pre-diagnostic emergency presentations
**Objectives, Specific Aims and Rationale**
The main objective of this work is to produce evidence of the potential for earlier diagnosis of a range of different conditions (colon cancer, ovarian cancer, pancreatic cancer, lung cancer, brain tumours, Parkinson's disease, schizophrenia, celiac disease, inflammatory bowel disease, rheumatoid arthritis, ankylosing spondylitis, multiple sclerosis, chronic obstructive pulmonary disease, tuberculosis, Lyme disease, subacute bacterial endocarditis, coronary/ischaemic heart disease, polycystic ovary syndrome). Achieving this objective will indirectly improve patient care by informing future research objectives (which conditions are most pressing for future diagnostic quality and safety research?) and improvement efforts.<br><br>The primary hypothesis to be tested is that diagnostic windows exist for many conditions and may be of considerable length pre-diagnosis.<br><br>The primary aim is to evaluate diagnostic window presence and length for a range of different conditions using pre-diagnostic healthcare utilisation.<br><br>Secondary aims are to document the frequency and prognostic implications of emergency presentations before the diagnosis of selected conditions and explore best methods for measuring diagnostic windows. Achieving these aims will produce the first available estimates of diagnostic window presence and length for many of the conditions we include. It will also describe groups of patients who may benefit most from earlier diagnosis. This will allow us to understand which of these conditions have the potential to be diagnosed earlier than at present.

Study Background

The study of diagnostic quality and safety, and particularly that of diagnostic windows, has largely developed in the cancer literature (1). For many cancer sites, diagnostic windows have been shown to exist and (using primary care consultations as the measure of healthcare utilisation) range in length from several months to a few years (2,3).

Current evidence on diagnostic windows for non-neoplastic diseases is limited; to our knowledge only four studies directly estimating diagnostic windows for non-neoplastic diseases exist: Miller et al. estimate diagnostic windows of 1 month, 3 months and 4 months for herpes simplex encephalitis, histoplasmosis and tuberculosis, respectively (4–6), while Erickson et al. estimate a diagnostic window of 1 month for necrotizing soft tissue infections of the genitalia (7).

A broader evidence base exists regarding patterns of pre-diagnostic healthcare use in patients subsequently diagnosed with non-neoplastic diseases: Parkinson's disease patients have consistently higher rates of healthcare encounters than controls for symptoms relevant to Parkinson's disease during the 10 years prior to diagnosis (8), while Inflammatory Bowel Disease patients report excess gastrointestinal symptoms in each of the 10 years prior to diagnosis (9) (ISAC protocol 15_081R). These studies suggest that diagnostic windows for these conditions could extend to more than 10 years. However, as differences in healthcare utilisation were observed for the entire study duration, there is insufficient evidence to make conclusions on the diagnostic window length. Additionally, it may be that patients with these conditions are inherently higher lifetime users of healthcare, and a case-only historical baseline may be a more appropriate comparator for healthcare utilisation. Similar conclusions can be drawn from studies on diseases such as COPD, Psychosis, Schizophrenia, Childhood Type 1 Diabetes Mellitus, Granulomatosis with Polyangiitis, Rheumatoid Arthritis and Multiple Sclerosis (10–17) (ISAC protocols 15_150, 15_208). Whilst the current literature provides evidence that diagnostic windows are likely to exist, it is insufficient to prove that they exist or measure their length. Thus current studies containing evidence of diagnostic windows lack in terms of (a) granularity (the length of the diagnostic window is not measured) (b) study duration (follow-up is insufficient to evaluate inflection points) and (c) linkage with hospitalization data (1).

Furthermore, existing diagnostic window literature is yet to reach a consensus on the most appropriate method for evaluating diagnostic window presence and length. Both case-only and case-control study designs have been used to compare healthcare utilisation (3,18) (ISAC protocol 17_107) with no clear determination of which, if either, is preferable. Additionally, a variety of approaches to identifying the inflection point in healthcare utilisation (start of the diagnostic window) have been used; this includes visual identification (19), statistical identification of the first time-period where healthcare utilisation differs from a baseline (20), and maximum likelihood estimation of the inflection point (18) (ISAC protocol 17_107). These methods have only been applied in the setting of cancer where it is reasonable to believe that diagnostic windows are unlikely to be longer than a few years. It is unclear how well existing methods will extend to situations such as Parkinson's disease, where diagnostic windows are likely to be much longer and changes in healthcare utilisation may be much more gradual. New techniques or extensions of existing techniques that can account for changes in healthcare utilisation due to aging may be required in these circumstances.

Additionally, emergency presentations prior to diagnosis of cancer are increasingly well-documented (21) and are associated with worse prognosis and patient satisfaction with care (22,23). As with diagnostic windows, there is a lack of documented evidence on the frequency of emergency presentations before diagnosis of non-neoplastic conditions in particular. Nonetheless, evidence from cancer population health studies indicates the concept is likely to be highly revealing.

Finally, for most of the conditions we include it is unclear what proportion of patients are only diagnosed upon their death (24–26). As with emergency presentations, documenting the frequency of these occurrences will aid interpretation of diagnostic window results.

Study Type

This study will be exploratory.

Study Design

This is a nested case-control study (although depending on results of initial methodological tests only cases may be required for some conditions). For each condition we include there will be a specific case group and a specific control group and the analyses for each condition will be carried out independently.

Planned use of linked data (if applicable):
We would require three linkages, between CPRD and other data sources (ONS death data, Hospital Episode Statistics (APC, OP, and A&E) data, IMD patient-level data).
Linkage to HES APC and OP data will allow us to identify diagnoses with additional accuracy, particularly when specialist input is typically needed to confirm diagnosis (30,31), although we acknowledge that diagnoses are coded less frequently in HES OP. Inclusion of cases identified on the basis of a diagnosis in HES OP will be considered carefully. Furthermore, linkage to HES A&E and HES APC will enable us to ascertain emergency presentations prior to diagnosis of selected conditions which is a secondary outcome of our study. Finally, linkage to ONS death data will allow us to determine how many patients, if any, are only diagnosed after death.

Linkage to HES APC, OP, and A&E datasets can be used to evaluate pre-diagnostic secondary healthcare utilisation for relevant conditions. For example, symptomatic presentations in secondary care, consultations with relevant specialties, and utilisation of emergency care prior to diagnosis.

Linkage to HES APC, HES A&E and ONS data will enable us to evaluate associations of emergency presentations with prognosis (measured in terms of risk of hospitalization or death in the year after diagnosis).

Finally, patient-level IMD data will allow us to control for deprivation when assessing healthcare utilisation rates and investigate differences in outcomes due to socio-economic status.

The main outputs of this study will benefit patients indirectly by providing new insights into conditions that could be diagnosed earlier, and estimates of how much earlier these diagnoses may theoretically be possible. This information can be used to inform future research and target diagnostic improvement efforts.

We recognise that these datasets are available for different periods of time. The main datasets that will be used for determining our cohort and carrying out the main analyses are all available for the period 1/1/1999-31/12/2019. As this is an exploratory study, supplementary analyses examining emergency presentations, or diagnostic windows using specific healthcare use events will be restricted to appropriate time periods as necessary.

Definition of the Study population

Conditions to be included in this study were determined by the following considerations:

- Most existing diagnostic window estimates are for neoplastic conditions (1). Including a selection of different cancer sites will (a) allow for comparisons to be made with existing non-UK evidence and (b) provide 'benchmarks' against which the diagnostic windows of non-neoplastic conditions can be compared. We include 5 cancer sites (colon cancer, ovarian cancer, pancreatic cancer, lung cancer, brain tumours)
- We carried out a literature review (manuscript soon to be submitted) on pre-diagnostic healthcare utilisation for non-neoplastic conditions to explore the possibility of diagnostic windows outside cancer. Evidence of changing healthcare use before diagnosis existed for many non-neoplastic conditions, indicating that diagnostic windows were likely; however as the majority of studies were not aiming to measure diagnostic windows further research is needed to accurately estimate diagnostic window presence and length. The literature review identified studies of pre-diagnostic healthcare use for 17 conditions; we include 11 of these (merged into 9 distinct conditions for this study; we include clinically isolated syndrome within our phenotypic definition of multiple sclerosis and psychosis within our phenotypic definition of schizophrenia and other chronic psychoses; acute myocardial infarction is included as an emergency presentation of coronary/ischaemic heart disease). Conditions identified in the review that are not included here are:
  - Childhood type 1 diabetes mellitus – We exclude paediatric forms of conditions a priori
  - Common variable immunodeficiency, granulomatosis with polyangiitis, herpes simplex encephalitis – These conditions are very rare and we anticipated sample sizes would be too small
  - Stroke – A stroke is an 'emergency presentation' of a number of possible underlying chronic conditions, ranging from primary hypertension to atrial fibrillation, for example. However, these conditions are too varied to be included under a single phenotype
  - Dementia – Dementia is sometimes considered to be under-coded in clinical practice
- Many of the studies identified in the literature review focused on autoimmune conditions. To test the hypothesis that diagnostic windows exist for different types of conditions we include two infections: Lyme disease and subacute bacterial endocarditis. From the literature review we also include tuberculosis.
- Ankylosing spondylitis and rheumatoid arthritis are both auto-immune conditions affecting joints, but dominant symptoms and patient populations differ. We include both to enable a comparison of their diagnostic windows.
- Finally, female-specific health conditions have historically been under-researched and there is evidence of widespread gender bias in healthcare, including in diagnosis. For these reasons, we will estimate diagnostic windows in men and women for each condition, and we include polycystic ovary syndrome in our study.

Possible diagnostic windows identified in the literature review ranged in length from 4 weeks for an acute condition, to 9 years for a chronic condition.

Given the above considerations, the final list of conditions included is

- Colon cancer
- Ovarian cancer
- Pancreatic cancer
- Lung cancer
- Brain tumours
- Parkinson's disease
- Schizophrenia, and other chronic psychoses
- Coeliac disease
- Inflammatory Bowel Disease (IBD)
- Rheumatoid Arthritis
- Ankylosing Spondylitis
- Multiple Sclerosis (MS)
- Chronic Obstructive Pulmonary Disease (COPD)
- Tuberculosis
- Lyme disease
- Subacute Bacterial Endocarditis (SBE)
- Coronary Heart Disease/Ischaemic Heart Disease (CHD)
- Polycystic Ovary Syndrome (PCOS)

Estimating diagnostic window presence and length for these conditions will produce evidence that diagnostic windows exist across non-neoplastic conditions of different types and aetiologies. It will also allow us to explore the best methods for

measuring diagnostic windows in a variety of different conditions. We believe this is a reasonable number of conditions to explore at this early stage, and that – dependent on the success of this study – this work could be scaled up to include more conditions in the future.

For each condition of interest, we will include all patients that satisfy all of the following criteria:
• Meet reasonable criteria describing the population at risk of the condition (e.g. right age group, see below)
• Have a first ever record of the condition of interest in a CPRD, HES or ONS table (as appropriate for condition of interest) between 1999 and 2019
• Have up-to-standard CPRD records at the date of diagnosis
• Have a minimum of 1 year between CRD/UTS and the date of diagnosis
• Are registered with practices that have consented to data linkage

We will exclude patients who do not meet reasonable criteria describing the population at risk of the condition. We a priori exclude juvenile or paediatric forms of the included conditions. Criteria for each condition are
• Colon cancer: We include all patients over 30 at the index date. We do not include cancer sites that fall under the ICD10 codes C19-21.
• Ovarian cancer: We include all women over 30 at the index date.
• Pancreatic cancer: We include all patients over 30 at the index date.
• Lung cancer: We include all patients over 30 at the index date.
• Brain tumours: We include all patients over 13 at the index date.
• Parkinson's disease: We include all patients over 30 at the index date.
• Schizophrenia, and other chronic psychoses: We include all patients over 16 at the index date. We include conditions that fall under the ICD10 codes F20, F21, F22, F25, F28, F29.
• Coeliac disease: We include all patients over 13 at the index date
• Inflammatory Bowel Disease (IBD): We include all patients over 13 at the index date. We include both Crohn's disease and ulcerative colitis.
• Rheumatoid Arthritis: We include all patients over 16 at the index date. • Ankylosing Spondylitis: We include all patients over 16 at the index date.
• Multiple Sclerosis (MS): We include all patients over 16 at the index date.
• Chronic Obstructive Pulmonary Disease (COPD): We include all patients over 30 at the index date
• Tuberculosis: We include all patients over 16 at the index date. We include primary, pulmonary, respiratory or miliary tuberculosis.
• Lyme disease: We include all patients over 16 at the index date.
• Subacute Bacterial Endocarditis (SBE): We include all patients over 16 at the index date.
• Coronary Heart Disease/Ischaemic Heart Disease (CHD): We include all patients over 30 at the index date
• Polycystic Ovary Syndrome (PCOS): We include all women over 13 at the index date.

The index date will be defined as the date of the first (non-prevalent) record of the condition of interest within the CPRD, HES and ONS datasets after a minimum period of 1-year during which the condition was not recorded and the patient was registered and up-to-standard (where UTS flag is available). A patient's follow-up will begin when they are registered at an up-to-standard CPRD practice and end 1 year after their index date (or at their transfer out date or deathdate, if earlier).

Selection of comparison group(s) or controls

Up to 5 individually-matched controls will be selected for each case, for each condition of interest. Controls will be assigned the same index date as their matched case, and each control will be matched to at most one case within each sample defined by the condition of interest. Controls will be disease free at the end-of-follow-up, i.e., should have no record of the condition of interest in CPRD clinical or referral (GOLD) or CPRD observation (Aurum), HES or ONS, but may have associated conditions. Controls will be matched on age, sex, and practice at the index date, and must meet reasonable criteria describing the population at risk of the condition (see below). 5:1 exact matching will be used, with patients judged to be matches if they have the same 5-year age-group, sex and practice at the index date.

Criteria for each condition are

- Colon cancer: We include all patients over 30 at the index date.
- Ovarian cancer: We include all women over 30 at the index date.
- Pancreatic cancer: We include all patients over 30 at the index date.
- Lung cancer: We include all patients over 30 at the index date.
- Brain tumours: We include all patients over 13 at the index date.
- Parkinson's disease: We include all patients over 30 at the index date.
- Schizophrenia, and other chronic psychoses: We include all patients over 16 at the index date.
- Coeliac disease: We include all patients over 13 at the index date
- Inflammatory Bowel Disease (IBD): We include all patients over 13 at the index date.
- Rheumatoid Arthritis: We include all patients over 16 at the index date. We exclude any patients with a prior diagnosis of Ankylosing Spondylitis.
- Ankylosing Spondylitis: We include all patients over 16 at the index date.
- Multiple Sclerosis (MS): We include all patients over 16 at the index date.
- Chronic Obstructive Pulmonary Disease (COPD): We include all patients over 30 at the index date
- Tuberculosis: We include all patients over 16 at the index date.
- Lyme disease: We include all patients over 16 at the index date.
- Subacute Bacterial Endocarditis (SBE): We include all patients over 16 at the index date.
- Coronary Heart Disease/Ischaemic Heart Disease (CHD): We include all patients over 30 at the index date
- Polycystic Ovary Syndrome (PCOS): We include all women over 13 at the index date.

The matching will produce separate case-control studies for each of the conditions of interest. Cases and controls for disease A are eligible for inclusion as either cases or controls for disease B.

We request 5 controls to increase the likelihood of each case having at least one control with the same amount of follow-up.

Exposures, Outcomes and Covariates

The main exposures of interest will be the diagnoses recorded in patients' CPRD, HES or ONS records (as appropriate). These diagnoses will be defined by a record of a diagnosis code of a condition of interest documented in the patient record. Conditions of interest have been selected to include a range of neoplastic and non-neoplastic, common and rare, acute and chronic diseases. Read v2, SNOMED and ICD-10 codelists to identify these conditions in primary care, secondary care and death data have been developed. Where possible these codelists have been developed from existing codelists from previous CPRD studies (33,34) (ISAC protocols 16_022, 19_173) and other disease-specific studies (27,35–44) (ISAC protocols 13_210R, 17_048, 13_126, 18_288, 08_031R, 17_168). When necessary, these codelists have been updated to include SNOMED codes for use in CPRD Aurum by manually searching SNOMED code browsers.

The main outcome will be healthcare utilisation as documented in the patient record prior to the diagnosis of a condition of interest and whilst the patient is registered at an up-to-standard CPRD practice. We include a patient's entire CPRD record prior to diagnosis as there is not currently any conclusive evidence for determining appropriate study duration for diagnostic window studies and many of the conditions of interest may have diagnostic windows lasting several years. We will consider several different types of healthcare utilisation events:

- All-cause primary care utilisation (all primary care encounters)
- Face-to-face primary care utilisation (face-to-face primary care encounters)
- Telephone or digital primary care utilisation (telephone or video primary care encounters, as determined from the CPRD consultation table (although we anticipate that this may not be possible with data currently available))
- All-cause secondary care utilisation (all encounters/stays as an inpatient, outpatient, or presentations to A&E)
- Inpatient secondary care utilisation (all inpatient encounters and stays)
- Outpatient secondary care utilisation (all outpatient encounters)
- Emergency care utilisation (all A&E encounters)

When measuring these types of healthcare utilisation we will first consider 'fact of consultation' (i.e. to measure all-cause primary care utilisation we will count each distinct interaction that a patient has had with primary care regardless of the content of that consultation). We will then consider measuring healthcare utilisation using features of consultations (for example, we will count each distinct primary care encounter where a specific symptom was recorded, each distinct primary care encounter where a certain blood test was ordered, each distinct outpatient encounter with a specific specialty, or each distinct secondary care encounter where a code from a specific ICD chapter was used).

Whilst outcomes being measured do not normally precede exposures, in diagnostic window studies this is appropriate (see (45)) due to the underlying assumption that any changes in pre-diagnostic healthcare utilisation are due to the as-yet-undiagnosed condition. Whilst the exposure can only be measured at diagnosis, and therefore after the outcome, cases are assumed to be exposed and controls unexposed for the duration when the outcomes are measured.

Secondary outcomes will be emergency presentations as recorded in HES A&E or HES APC (admissions from A&E) prior to the diagnosis of a condition of interest and whilst the patient is registered at an up-to-standard CPRD practice, and deaths as recorded in ONS.

We will include as covariates patient factors known to potentially impact the timeliness of diagnosis: age at diagnosis, sex, deprivation, Cambridge Multimorbidity score (46) and year of diagnosis as derived from CPRD, HES, ONS and IMD records. We will examine whether diagnostic window presence and length varies by any of these factors by either (i) restricting the population of interest to only include patients with a certain characteristic or (ii) including these factors in our modelling of primary care consultation rates depending on the choice of analytic method.

Data/ Statistical Analysis
As described above, there is currently no consensus on the optimal method for measuring diagnostic windows or any understanding of whether different methods may result in different estimates. The first part of this study will apply different methods to 'exemplar' conditions to determine the optimal method(s) in our context. The exemplar conditions will include a neoplastic condition (to allow comparison with existing diagnostic window estimates), a condition likely to have a short diagnostic window, a condition whose definitive diagnosis typically requires input from secondary care specialists, and a condition that is likely to have a long diagnostic window.

This will allow us to examine how methods perform in different diseases. A good method should produce reliable estimates of diagnostic window length in all four scenarios. Possible methods for inclusion are:
• Comparison of monthly rates in cases and controls – what is the earliest month that rates differ?
• Comparison of monthly rates in cases to a baseline – what is the earliest month that the rate differs?
• Maximum Likelihood Estimation (MLE) case-only approach (18,47)
• MLE case-control approach
Other methods may also be included as appropriate.

The results of applying each method on each condition will be considered against the following criteria:
• Robustness: When given a slightly different sample from same population, how different is the dx window estimate?
• Clinical validity: Are the resulting estimates in line with reasonable clinical scenarios that would explain the data?
• Reliability: Does the method perform well for (true, underlying) diagnostic windows of different lengths as will be the case for different diseases? Does the method perform well for populations who consult at different rates naturally (e.g., older vs younger)?

This preliminary analysis will be used to determine the most appropriate method(s) to be used in the main analysis.
In the main analysis we will estimate the diagnostic window presence and length of each condition of interest by applying the appropriate method (as determined above) to the pre-diagnostic healthcare utilisation of the sample of patients with this condition and (as appropriate) that of their matched controls. For this main aim, multiple testing is not a concern.

Pre-diagnostic healthcare utilisation will be measured initially via primary care consultation rates (face-to-face and phone consultations) using Poisson regression or negative binomial regression, which will include consideration of other covariates (age, gender, deprivation). Diagnostic windows will be measured on a daily, weekly, monthly or yearly scale as deemed appropriate for each condition of interest and the method being applied. Diagnostic windows will be measured for the population of patients with each condition of interest, and for subgroups determined by covariates.

As the diagnostic window length for most of these conditions is unknown, it is unclear what duration of pre-diagnostic records will be required for each condition. To address this, for each condition we will iteratively apply the diagnostic window method to records of sequentially longer follow-up duration, until the diagnostic window estimate is stable over multiple periods, or the sample size becomes too small and the results will be deemed inconclusive. As an example, we will first apply the method to 1 year of records before diagnosis: either an estimate of a diagnostic window shorter than a year will be produced (which could be 0), or a difference will extend for the entire duration. We then apply the method to 2 years of records before diagnosis: if the same diagnostic window estimate is produced, we can stop; otherwise we continue for another year.

Due to the different coding systems used in CPRD GOLD and Aurum, there may be different recording practices for all, or for a subset of, the included conditions. It is possible that these recording practices may in turn result in different healthcare utilisation patterns. To understand this, where sample size allows, we will conduct a sensitivity analysis by stratifying patients on whether they come from a GOLD or Aurum practice and seeing how this affects diagnostic window presence and length. We will include practice region as a covariate to account for differences in the geographical distribution of GOLD and Aurum practices. Due to sample size constraints, this sensitivity analysis may only be possible for some conditions, but will help us evaluate whether recording practices impact diagnostic window presence and length.

To address our secondary aims we will produce descriptive statistics of emergency presentations prior to diagnosis of the included conditions. These will be identified as emergency inpatient admissions in HES APC and presentations to emergency settings from HES A&E. We will evaluate the association of emergency presentations with prognosis by considering the risk of hospital admission (as per HES) and death (as per ONS) in the year following diagnosis.

Finally, we will produce descriptive statistics of diagnoses that were only made in ONS death data. This will allow us to

consider whether there are certain subgroups of patients that may not be captured in our diagnostic windows analysis. This will not be applicable for every condition we include (e.g. ankylosing spondylitis).

Plan for addressing confounding

We will estimate diagnostic windows for the population of patients with each condition of interest, and for subgroups determined by covariates (as relevant for the condition of interest). It is unlikely that these subgroups will be small enough to significantly increase risk of re-identification.

Confounding is most likely to occur due to healthcare utilisation rates increasing with age and over time. To address this, we will use controls matched on age, use the same index date for cases and controls, and account for age and year in our modelling. Matching on follow-up length is not appropriate for this study as it would introduce bias into diagnostic window estimates.

Using matched cases and controls will also help address other confounding that could occur.

Plans for addressing missing data

Some diagnoses of conditions of interest may be missing or not have been recorded by the doctor (or be recorded as free text). Despite this, we expect that the majority of diagnoses considered to be clinically relevant by doctors will be recorded as coded entries, and thus will be available for analysis. To measure diagnostic windows, we are primarily interested in the fact of consultation, thus any missingness in recording of symptoms or tests will not be a concern. This will allow us to consider diagnostic windows for a range of conditions.

For covariates, we do not expect substantial missingness regarding age, sex, or deprivation. Any patients with missing age or sex will be excluded, whilst IMD will be imputed.

Whilst patient records prior to diagnosis are likely to widely differ in duration, we have imposed a minimum length of 1 year of records prior to diagnosis as part of our inclusion criteria. The number of patients for whom valid records are available at any given point prior to diagnosis will be accounted for in the modelling of primary care consultation rates (for example, as an offset). Additionally, we seek multiple controls per case to try to ensure that few cases will have unmatched periods due to left truncation.

Limitations of the study design, data sources, and analytic methods

Some conditions may be under-recorded in primary care data, however our request for linkage to HES data should mitigate some of this under-recording. Where conditions are under-recorded, or diagnoses are inaccurate, the estimates produced are still likely to represent the first available estimates of diagnostic windows for the condition of interest. These can be refined further in time with more in-depth studies using more advanced phenotypes to define cases.

Additionally, due to the different coding systems used in CPRD GOLD and Aurum, there may be different recording practices for all, or for a subset of, the included conditions. Where sample size allows, we will conduct a sensitivity analysis by stratifying patients on whether they come from a GOLD or Aurum practice.

Some GOLD practices may have changed software and become Aurum practices. We will request the ‘migrators’ file to identify these practices and remove duplicates from our sample.

For many of the conditions we include, our results will be the first available estimates of diagnostic windows. As such, we do not know how long these windows will be or how much follow-up will be required to measure them accurately. It is possible that for some conditions, insufficient follow-up will be available to produce accurate estimates. However, presenting these findings will provide evidence to support accurate estimates in the future.

References

1. White B, Renzi C, Rafiq M, Abel GA, Jensen H, Lyratzopoulos G. Does changing healthcare use signal opportunities for earlier detection of cancer? A review of studies using information from electronic patient records. Cancer Epidemiol. 2021 Dec 4;102072.
2. Ansell P, Johnston T, Simpson J, Crouch S, Roman E, Picton S. Brain tumor signs and symptoms: analysis of primary health care records from the UKCCS. Pediatrics [Internet]. 2010 Jan [cited 2022 Apr 11];125(1):112–9. Available from: https://pubmed.ncbi.nlm.nih.gov/20026498/
3. Ewing M, Naredi P, Nemes S, Zhang C, Månsson J. Increased consultation frequency in primary care, a risk marker for cancer: a case-control study. Scand J Prim Health Care [Internet]. 2016 Apr 2 [cited 2022 Apr 11];34(2):204–11. Available from: https://pubmed.ncbi.nlm.nih.gov/27189513/
4. Miller AC, Arakkal AT, Koeneman S, Cavanaugh JE, Gerke AK, Hornick DB, et al. Incidence, duration and risk factors associated with delayed and missed diagnostic opportunities related to tuberculosis: a population-based longitudinal study. BMJ Open [Internet]. 2021 Feb 1 [cited 2021 Sep 22];11(2):e045605. Available from: https://bmjopen.bmj.com/content/11/2/e045605
5. Miller AC, Koeneman SH, Arakkal AT, Cavanaugh JE, Polgreen PM. Incidence, Duration, and Risk Factors Associated With Missed Opportunities to Diagnose Herpes Simplex Encephalitis: A Population-Based Longitudinal Study. Open Forum Infect Dis [Internet]. 2021 Sep 1 [cited 2021 Sep 23];8(9). Available from: /pmc/articles/PMC8415533/
6. Miller AC, Arakkal AT, Koeneman SH, Cavanaugh JE, Thompson GR, Baddley JW, et al. Frequency and Duration of, and Risk Factors for, Diagnostic Delays Associated with Histoplasmosis. Journal of Fungi 2022, Vol 8, Page 438 [Internet]. 2022 Apr 23 [cited 2022 Jun 7];8(5):438. Available from: https://www.mdpi.com/2309-608X/8/5/438/htm
7. Erickson BA, Miller AC, Warner HL, Drobish JN, Koeneman SH, Cavanaugh JE, et al. Understanding the Prodrome of Necrotizing Soft Tissue Infections of the Genitalia (Fournier's Gangrene) and the Incidence, Duration, and Risk Factors Associated with Potential Missed Opportunities for an Earlier Diagnosis: A Population-Based Longitudinal Study. J Urol [Internet]. 2022 Aug 25 [cited 2022 Aug 30]; Available from: https://pubmed.ncbi.nlm.nih.gov/36006046/
8. Schrag A, Horsfall L, Walters K, Noyce A, Petersen I. Prediagnostic presentations of Parkinson's disease in primary care: a case-control study. Lancet Neurol [Internet]. 2015 Jan 1 [cited 2021 Sep 22];14(1):57–64. Available from: http://www.thelancet.com/article/S147444221470287X/fulltext
9. Blackwell J, Saxena S, Jayasooriya N, Bottle A, Petersen I, Hotopf M, et al. Prevalence and Duration of Gastrointestinal Symptoms Before Diagnosis of Inflammatory Bowel Disease and Predictors of Timely Specialist Review: A Population-Based Study. J Crohns Colitis [Internet]. 2021 Feb 1 [cited 2021 Sep 22];15(2):203–11. Available from: https://academic.oup.com/ecco-jcc/article/15/2/203/5871943
10. Johnson KM, Khakban A, Bryan S, Sin DD, Sadatsafavi M. Healthcare system encounters before COPD diagnosis: a registry-based longitudinal cohort study. Thorax [Internet]. 2020 Feb 1 [cited 2021 Oct 8];75(2):108–15. Available from: https://thorax.bmj.com/content/75/2/108
11. Sullivan SA, Hamilton W, Tilling K, Redaniel T, Moran P, Lewis G. Association of Primary Care Consultation Patterns With Early Signs and Symptoms of Psychosis. JAMA Netw Open [Internet]. 2018 Nov 2 [cited 2021 Oct 8];1(7):e185174. Available from: /pmc/articles/PMC6324409/
12. Nørgaard HCB, Pedersen HS, Fenger-Grøn M, Mors O, Nordentoft M, Vestergaard M, et al. Increased use of primary care during 6 years of prodromal schizophrenia. Acta Psychiatr Scand [Internet]. 2016 Sep 1 [cited 2021 Oct 8];134(3):225–33. Available from: https://onlinelibrary.wiley.com/doi/full/10.1111/acps.12600
13. Lee JJ, Thompson MJ, Usher-Smith JA, Koshiaris C, Bruel A van den. Opportunities for earlier diagnosis of type 1 diabetes in children: A case-control study using routinely collected primary care records. Prim Care Diabetes [Internet]. 2018 Jun 1 [cited 2021 Oct 8];12(3):254–64. Available from: http://www.primary-care-diabetes.com/article/S1751991818300111/fulltext
14. Townson J, Cannings-John R, Francis N, Thayer D, Gregory JW. Presentation to primary care during the prodrome of type 1 diabetes in childhood: A case-control study using record data linkage.

Pediatr Diabetes [Internet]. 2019 May 1 [cited 2021 Oct 8];20(3):330–8. Available from: https://onlinelibrary.wiley.com/doi/full/10.1111/pedi.12829
15. Pearce FA, Hubbard RB, Grainge MJ, Watts RA, Abhishek A, Lanyon PC. Can granulomatosis with polyangiitis be diagnosed earlier in primary care? A case–control study. QJM: An International Journal of Medicine [Internet]. 2018 Jan 1 [cited 2021 Oct 8];111(1):39–45. Available from: https://academic.oup.com/qjmed/article/111/1/39/4381907
16. Disanto G, Zecca C, MacLachlan S, Sacco R, Handunnetthi L, Meier UC, et al. Prodromal symptoms of multiple sclerosis in primary care. Ann Neurol [Internet]. 2018 Jun 1 [cited 2021 Oct 8];83(6):1162–73. Available from: https://onlinelibrary.wiley.com/doi/full/10.1002/ana.25247
17. de Thurah A, Jensen H, Maribo T, Jensen MB, Sandbaek A, Hauge EM, et al. Use of primary health care and radiological imaging preceding a diagnosis of rheumatoid arthritis: a nationwide cohort study in Denmark. Rheumatology [Internet]. 2022 Jun 17 [cited 2022 Jul 27]; Available from: https://academic.oup.com/rheumatology/advance-article/doi/10.1093/rheumatology/keac306/6609759
18. Zhou Y, Abel GA, Hamilton W, Singh H, Walter FM, Lyratzopoulos G. Imaging activity possibly signalling missed diagnostic opportunities in bladder and kidney cancer: A longitudinal data-linkage study using primary care electronic health records. Cancer Epidemiol [Internet]. 2020 Jun 1 [cited 2022 Apr 11];66. Available from: https://pubmed.ncbi.nlm.nih.gov/32334389/
19. Christensen KG, Fenger-Grøn M, Flarup KR, Vedsted P. Use of general practice, diagnostic investigations and hospital services before and after cancer diagnosis - a population-based nationwide registry study of 127,000 incident adult cancer patients. BMC Health Serv Res [Internet]. 2012 [cited 2022 May 13];12:224. Available from: /pmc/articles/PMC3507912/
20. Ahrensberg JM, Fenger-Grøn M, Vedsted P. Use of Primary Care during the Year before Childhood Cancer Diagnosis: A Nationwide Population-Based Matched Comparative Study. PLoS One [Internet]. 2013 Mar 12 [cited 2022 May 13];8(3). Available from: /pmc/articles/PMC3595276/
21. McPhail S, Swann R, Johnson SA, Barclay ME, Elkader HA, Alvi R, et al. Risk factors and prognostic implications of diagnosis of cancer within 30 days after an emergency hospital admission (emergency presentation): an International Cancer Benchmarking Partnership (ICBP) population-based study. Lancet Oncol [Internet]. 2022 May 1 [cited 2022 May 11];23(5):587–600. Available from: http://www.thelancet.com/article/S1470204522001279/fulltext
22. Danckert B, Falborg AZ, Christensen NL, Frederiksen H, Lyratzopoulos G, McPhail S, et al. Routes to diagnosis and the association with the prognosis in patients with cancer - A nationwide register-based cohort study in Denmark. Cancer Epidemiol [Internet]. 2021 Oct 1 [cited 2022 May 11];74. Available from: https://pubmed.ncbi.nlm.nih.gov/34352658/
23. Pham TM, Gomez-Cano M, Salika T, Jardel D, Abel GA, Lyratzopoulos G. Diagnostic route is associated with care satisfaction independently of tumour stage: Evidence from linked English Cancer Patient Experience Survey and cancer registration data. Cancer Epidemiol [Internet]. 2019 Aug 1 [cited 2022 May 11];61:70–8. Available from: https://pubmed.ncbi.nlm.nih.gov/31153049/
24. Nakhleh RE, Nosé V, Colasacco C, Fatheree LA, Lillemoe TJ, McCrory DC, et al. Overview of Diagnostic Error in Health Care. Arch Pathol Lab Med [Internet]. 2015 Dec 29 [cited 2022 Sep 9];140(1):29–40. Available from: https://www.ncbi.nlm.nih.gov/books/NBK338594/
25. Graber ML. The incidence of diagnostic error in medicine. [cited 2022 Sep 9]; Available from: http://dx.doi.org/10.1136/bmjqs-2012-001615
26. Danckert B, Christensen NL, Falborg AZ, Frederiksen H, Lyratzopoulos G, McPhail S, et al. Assessing how routes to diagnosis vary by the age of patients with cancer: a nationwide register-based cohort study in Denmark. BMC Cancer [Internet]. 2022 Aug 19 [cited 2022 Sep 9];22(1). Available from: https://pubmed.ncbi.nlm.nih.gov/35986279/
27. Jessen NH, Jensen H, Falborg AZ, Glerup H, Gronbaek H, Vedsted P. Contacts to general practice in the 12 months preceding a diagnosis of an abdominal cancer: a national register-based cohort study. Scand J Prim Health Care [Internet]. 2022 [cited 2022 Jun 20];40(1):148–56. Available from: https://www.tandfonline.com/doi/abs/10.1080/02813432.2022.2057054

28. Wang Y, Hunt K, Nazareth I, Freemantle N, Petersen I. Do men consult less than women? An analysis of routinely collected UK general practice data. BMJ Open [Internet]. 2013 Aug 1 [cited 2022 Jul 26];3(8):e003320. Available from: https://bmjopen.bmj.com/content/3/8/e003320
29. Morris S, Sutton M, Gravelle H. Inequity and inequality in the use of health care in England: an empirical investigation. Soc Sci Med. 2005 Mar 1;60(6):1251–66.
30. Herrett E, Gallagher AM, Bhaskaran K, Forbes H, Mathur R, Staa T van, et al. Data Resource Profile: Clinical Practice Research Datalink (CPRD). Int J Epidemiol [Internet]. 2015 Jun 1 [cited 2022 May 11];44(3):827–36. Available from: https://pubmed.ncbi.nlm.nih.gov/26050254/
31. Persson R, Sponholtz T, Vasilakis-Scaramozza C, Hagberg KW, Williams T, Kotecha D, et al. Quality and Completeness of Myocardial Infarction Recording in Clinical Practice Research Datalink Aurum. Clin Epidemiol [Internet]. 2021 [cited 2022 May 11];13:745–53. Available from: https://pubmed.ncbi.nlm.nih.gov/34471387/
32. Schneeweiss S, Rassen JA, Brown JS, Rothman KJ, Happe L, Arlett P, et al. Graphical depiction of longitudinal study designs in health care databases. Ann Intern Med [Internet]. 2019 [cited 2022 May 13];170(6):398–406. Available from: www.repeatinitiative.org/projects.html.
33. Kuan V, Denaxas S, Gonzalez-Izquierdo A, Direk K, Bhatti O, Husain S, et al. A chronological map of 308 physical and mental health conditions from 4 million individuals in the English National Health Service. Lancet Digit Health [Internet]. 2019 [cited 2021 Mar 3];1:e63–77. Available from: www.thelancet.com/
34. Head A, Fleming K, Kypridemos C, Schofield P, Pearson-Stuttard J, O'Flaherty M. Inequalities in incident and prevalent multimorbidity in England, 2004–19: a population-based, descriptive study. Lancet Healthy Longev. 2021 Aug 1;2(8):e489–97.
35. Renzi C, Lyratzopoulos G, Hamilton W, Maringe C, Rachet B. Contrasting effects of comorbidities on emergency colon cancer diagnosis: A longitudinal data-linkage study in England. BMC Health Serv Res [Internet]. 2019 May 15 [cited 2022 Jul 13];19(1):1–14. Available from: https://bmchealthservres.biomedcentral.com/articles/10.1186/s12913-019-4075-4
36. Nygaard C, Jensen H, Christensen J, Vedsted P. Health care use before a diagnosis of primary intracranial tumor: a Danish nationwide register study. Clin Epidemiol [Internet]. 2018 Jul 12 [cited 2022 Jul 13];10:809–29. Available from: https://www.dovepress.com/health-care-use-before-a-diagnosis-of-primary-intracranial-tumor-a-dan-peer-reviewed-fulltext-article-CLEP
37. Launders N, Hayes JF, Price G, Osborn DPJ. Clustering of physical health multimorbidity in people with severe mental illness: An accumulated prevalence analysis of United Kingdom primary care data. PLoS Med [Internet]. 2022 Apr 1 [cited 2022 Jul 4];19(4):e1003976. Available from: https://journals.plos.org/plosmedicine/article?id=10.1371/journal.pmed.1003976
38. Freeman K, Ryan R, Parsons N, Taylor-Phillips S, Willis BH, Clarke A. The incidence and prevalence of inflammatory bowel disease in UK primary care: a retrospective cohort study of the IQVIA Medical Research Database. BMC Gastroenterol [Internet]. 2021 Dec 1 [cited 2022 Jul 4];21(1):1–7. Available from: https://bmcgastroenterol.biomedcentral.com/articles/10.1186/s12876-021-01716-6
39. Muller S, Hider SL, Raza K, Stack RJ, Hayward RA, Mallen CD. An algorithm to identify rheumatoid arthritis in primary care: a Clinical Practice Research Datalink study. BMJ Open [Internet]. 2015 Dec 1 [cited 2022 Jul 4];5(12):e009309. Available from: https://bmjopen.bmj.com/content/5/12/e009309
40. Rockenschaub P, Jhass A, Freemantle N, Aryee A, Rafiq M, Hayward A, et al. Opportunities to reduce antibiotic prescribing for patients with COPD in primary care: a cohort study using electronic health records from the Clinical Practice Research Datalink (CPRD). Journal of Antimicrobial Chemotherapy [Internet]. 2020 Jan 1 [cited 2022 Jul 5];75(1):243–51. Available from: https://academic.oup.com/jac/article/75/1/243/5584403
41. Cairns V, Wallenhorst C, Rietbrock S, Martinez C. Incidence of Lyme disease in the UK: a population-based cohort study. BMJ Open [Internet]. 2019 Jul 1 [cited 2022 Jul 4];9(7):e025916. Available from: https://bmjopen.bmj.com/content/9/7/e025916
42. Zghebi SS, Mamas MA, Ashcroft DM, Rutter MK, Vanmarwijk H, Salisbury C, et al. Assessing the severity of cardiovascular disease in 213 088 patients with coronary heart disease: a retrospective

cohort study. Open Heart [Internet]. 2021 Apr 1 [cited 2022 Jul 8];8(1):e001498. Available from: https://openheart.bmj.com/content/8/1/e001498

43. Rees DA, Jenkins-Jones S, Morgan CL. Contemporary Reproductive Outcomes for Patients With Polycystic Ovary Syndrome: A Retrospective Observational Study. J Clin Endocrinol Metab [Internet]. 2016 Apr 1 [cited 2022 Jul 5];101(4):1664–72. Available from: https://academic.oup.com/jcem/article/101/4/1664/2804548

44. Iwundu C, MacRae C, Vasileiou E. Phenotype Library | phenotype: Tuberculosis Primary care [Internet]. 2022 [cited 2022 Jul 27]. Available from: https://phenotypes.healthdatagateway.org/phenotypes/PH818/version/1715/detail/

45. Ahrensberg JM, Fenger-Grøn M, Vedsted P. Use of primary care during the year before childhood cancer diagnosis: a nationwide population-based matched comparative study. PLoS One [Internet]. 2013 Mar 12 [cited 2022 May 18];8(3). Available from: https://pubmed.ncbi.nlm.nih.gov/23554980/

46. Payne RA, Mendonca SC, Elliott MN, Saunders CL, Edwards DA, Marshall M, et al. Development and validation of the Cambridge Multimorbidity Score. CMAJ [Internet]. 2020 Feb 3 [cited 2022 May 18];192(5):E107–14. Available from: https://pubmed.ncbi.nlm.nih.gov/32015079/

47. Joinpoint Regression Program [Internet]. [cited 2022 Apr 11]. Available from: https://surveillance.cancer.gov/joinpoint/
